# SRA-Domain Proteins Required for DRM2-Mediated De Novo DNA Methylation

**DOI:** 10.1371/journal.pgen.1000280

**Published:** 2008-11-28

**Authors:** Lianna M. Johnson, Julie A. Law, Anuj Khattar, Ian R. Henderson, Steven E. Jacobsen

**Affiliations:** 1 Life Sciences Core Curriculum, University of California Los Angeles, Los Angeles, California, United States of America; 2 Department of Molecular, Cell, and Developmental Biology, University of California Los Angeles, Los Angeles, California, United States of America; 3 Howard Hughes Medical Institute, University of California Los Angeles, Los Angeles, California, United States of America; 4 Department of Plant Sciences, University of Cambridge, Cambridge, United Kingdom; National Institute of Genetics, Japan

## Abstract

De novo DNA methylation and the maintenance of DNA methylation in asymmetrical sequence contexts is catalyzed by homologous proteins in plants (DRM2) and animals (DNMT3a/b). In plants, targeting of DRM2 depends on small interfering RNAs (siRNAs), although the molecular details are still unclear. Here, we show that two SRA-domain proteins (SUVH9 and SUVH2) are also essential for DRM2-mediated de novo and maintenance DNA methylation in *Arabidopsis thaliana*. At some loci, SUVH9 and SUVH2 act redundantly, while at other loci only SUVH2 is required, and this locus specificity correlates with the differing DNA-binding affinity of the SRA domains within SUVH9 and SUVH2. Specifically, SUVH9 preferentially binds methylated asymmetric sites, while SUVH2 preferentially binds methylated CG sites. The *suvh9* and *suvh2* mutations do not eliminate siRNAs, suggesting a role for SUVH9 and SUVH2 late in the RNA-directed DNA methylation pathway. With these new results, it is clear that SRA-domain proteins are involved in each of the three pathways leading to DNA methylation in Arabidopsis.

## Author Summary

Our genetic heritage plays an important role in determining who we are and the characteristics we possess. However, in the past decade it has become increasingly clear that in addition to the genes we inherit, a second level of information is critical for expression of these genes. This information takes the form of modifications to either the DNA (DNA methylation) or the proteins that package the DNA (histones). These modifications can determine whether a gene is expressed or silenced. In this paper, we identify two new genes that are part of a DNA methylation–targeting pathway in the model plant *A. thaliana*. Disruption of these two closely related genes prevents DNA methylation by one of the cellular DNA methyltransferases. However, these genes are not simply redundant. They are both capable of binding methylated DNA, but differ in their preference for specific sequences in the genome. This ability to bind to methylated DNA suggests that these proteins help target or retain the modification apparatus at particular regions of the genome. These results are important in that they identify two new players in this vital cellular process and bring us closer to understanding how epigenetic modifications can be targeted to specific genes.

## Introduction

Cytosine methylation is found in the genomes of most eukaryotes and plays a critical role in repression of transposable elements as well as in the epigenetic regulation of select genes [1]–[4]. In *Arabidopsis thaliana* cytosine methylation occurs in all sequence contexts, with the highest frequency occurring at CG, followed by CHG and then CHH sequences (where H is any base other than a G) [5]. Maintenance of CG methylation is catalyzed by MET1, a homolog of mammalian DNMT1 [6],[7]. DNMT1 functions at the replication fork, prefers hemi-methylated DNA as a template, and interacts with PCNA in vivo [8]. Recent studies have shown that an SRA-domain protein is required to efficiently target DNMT1 to the replication fork and to maintain high levels of CG methylation [9]–[11]. A key to this targeting in mammals was shown to involve preferential binding of the UHRF1 SRA domain to hemi-methylated CG sites [10], which are the physiological substrates for DNMT1 that are generated after replication of methylated DNA. A plant homolog of UHRF1, VIM1/ORTH2, also binds methylated CG sites and is required for maintenance of DNA methylation in Arabidopsis [12],[13], suggesting that this pathway is widely conserved in eukaryotes.

In Arabidopsis CHG methylation is primarily catalyzed by CHROMOMETHYLASE 3 (CMT3), which is also dependent on an SRA-domain protein, KRYPTONITE (KYP/SUVH4) [14],[15]. In this case, KYP is the main enzyme catalyzing methylation of histone H3 lysine 9, providing a binding site for CMT3 through its chromodomain [16]. Two other SRA-SET proteins (SUVH5 and SUVH6), which also methylate H3 lysine 9, contribute to this pathway as well [17]–[19]. Just as CMT3 binds to the mark put on by KYP, KYP and SUVH6 have been shown to bind directly to DNA methylated at CHG sites through their SRA domains, leading to a reinforcing loop between histone lysine 9 methylation and CHG methylation [12].

The de novo DNA methyltransferase DOMAINS REARRANGED METHYLTRANSFERASE (DRM2), a homolog of mammalian DNMT3 enzymes, is required for maintenance of non-CG methylation at some loci and initiation of DNA methylation in all contexts [20],[21]. Targeting of DRM2 to specific loci is dependent on several proteins involved in siRNA biosynthesis (RNA polymerase IVa: NRPD1a and NRPD2; RNA polymerase IVb: NRPD1b and NRPD2; RNA-dependent RNA polymerase: RDR2; Dicer-like3: DCL3) along with an argonaute protein (AGO4) and two SNF-related genes (DRD1 and CLASSY)[22]–[26]. We show here that two SRA-domain containing proteins, SUVH9 and SUVH2, are also essential to the DRM2 pathway. Furthermore, we show that the SRA domains of SUVH9 and SUVH2 bind directly to methylated DNA and that mutations in the SRA domains reduce DRM2 function in vivo. Thus, SRA domain proteins play critical roles in all three of the major DNA methylation pathways in Arabidopsis controlled by the MET1, CMT3 and DRM2 methyltransferases.

## Results

### SDC Gene Expression Is Activated in *suvh9 suvh2 kyp* Triple Mutants

**Figure 1:**
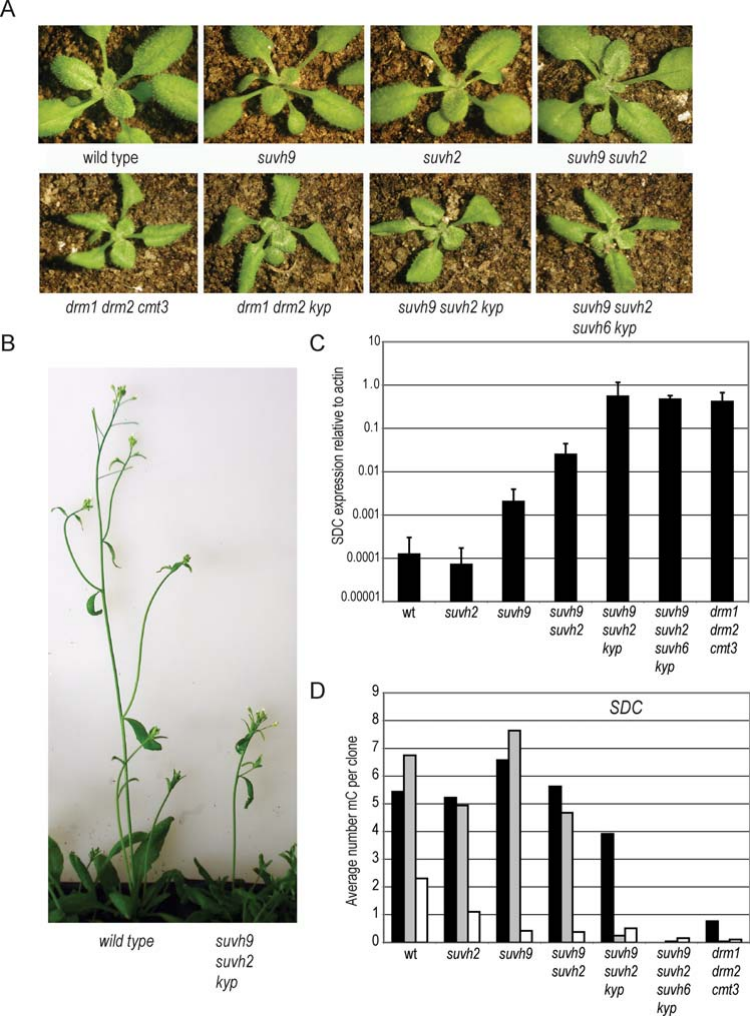
*suvh9 suvh2 kyp* triple mutants show a similar phenotype as *drm1 drm2 cmt3* triple mutants. A. Curled-leaf phenotype of young seedlings. Wild-type line is Columbia accession and all mutants are homozygous for T-DNA insertions in the Columbia background. The developmental phenotype appears in the first generation. B. Short stature phenotype observed in *suvh9 suvh2 kyp* plants. C. Quantitative RT-PCR of *SDC* gene expression compared to *ACTIN* plotted on log scale. D. Bisulfite sequence results of the *SDC* tandem repeats region. Between 13–21 independent clones were sequenced and the results are shown as the average number of methyl cytosines per clone. See Figure S2 for methylation expressed as percentage of methyl cytosine and Figure S3 for alignments. Black bar represents CG, gray CHG, and white is CHH methylation.

The importance of SRA-domain proteins in both the MET1 and CMT3 pathways led us to investigate other members of these families in Arabidopsis. There are nine SRA-SET proteins (SUVH1-9), six SRA-RING proteins (VIM1-6) and two proteins that contain only an SRA domain [12]. SUVH9 and SUVH2 are closely related to each other, but divergent from other members of the SUVH group (Figure S1A). KYP, SUVH5 and SUVH6 form a second clade, while SUVH1, SUVH3, SUVH7 and SUVH8 form a third clade. We obtained T-DNA insertion lines in each of the SUVH genes [27],[28] and constructed a series of single, double, triple, and quadruple homozygous mutant combinations. While none of the single T-DNA mutants had any obvious phenotype, a *suvh9 suvh2 kyp* triple mutant displayed morphological differences from wild type. These morphological defects are indistinguishable from those displayed by a DNA methyltransferase triple mutant *drm1 drm2 cmt3* (Figure 1A–B; *DRM1* and *DRM2* are tightly linked genes and in all cases the double mutant is examined though no activity has been ascribed to *DRM1*) [20],[29]. This phenotype, which consists of curling of the leaves and short stature, has recently been shown to be caused by the ectopic expression of an F-box gene, *SUPPRESSOR of DRM1 DRM2 CMT3 (SDC)*, which is silenced by non-CG DNA methylation occurring at tandem repeats found in its promoter [30]. Simultaneous disabling of both the CMT3 pathway and the DRM2 pathway is required for activation of *SDC* and the appearance of this developmental phenotype. In this respect, mutations in siRNA biosynthesis pathway genes can substitute for *drm2* mutations, and *kyp* mutations can substitute for *cmt3* mutations. For example, the *SDC* overexpression developmental phenotype can also be seen in the following triple mutants: *drm1 drm2 kyp*, *nrpd2a nrpd2b kyp*, and *nrpd2a nrpd2b cmt3* [29],[30]. Based on the morphological defect of the *suvh9 suvh2 kyp* triple mutant, we therefore reasoned that these mutations must also be blocking both the DRM2 and the CMT3 pathways. Furthermore, since KYP has clearly been shown to be required for CMT3 but not DRM2 activity [14],[15], *suvh9 suvh2* is likely preventing DRM2 function. To confirm that the *suvh9 suvh2 kyp* phenotype was indeed due to reactivation of *SDC*, RNA levels were measured using reverse transcription quantitative PCR (RT-qPCR; Figure 1C). We found SDC expression in *suvh9 suvh2 kyp* was elevated 10,000-fold over wild-type expression levels (normalized to *ACTIN*). This is similar to what is observed in the *drm1 drm2 cmt3* mutant (Figure 1C). The *suvh2* mutant alone had no effect on SDC expression and *suvh9* had a weak affect (10 fold above wild type control). The double mutant *suvh9 suvh2* increased expression 100 fold above the control, but was still 100 fold lower than the triple mutant *suvh9 suvh2 kyp*. The level of expression in *suvh9suvh2* is below what is required to observe a full morphological phenotype.

Since expression of *SDC* is inhibited by non-CG promoter DNA methylation, the extent of methylation at the *SDC* repeats was examined in the *suvh* mutants using bisulfite sequencing. We found that disruption of either *suvh2* or *suvh9* or both resulted in a reduction of the CHH methylation, with little change in other types of methylation (Figure 1D and Figure S2). This is comparable to what has been previously observed in a *drm1 drm2* mutant (Figure S2 and [30]). While the *suvh9 suvh2 kyp* triple mutant shows a loss of both CHG and CHH methylation, it has little effect on CG methylation (Figure 1D). This differs from *drm1 drm2 cmt3* which shows almost complete loss of all methylation (Figure 1D). We hypothesized that the residual methylation is *suvh9 suvh2 kyp* might be due to CMT3 activity, because it is known that SUVH6 functions redundantly at some loci to control CMT3 action [18]. We therefore analyzed the *suvh9 suvh2 suvh6 kyp* quadruple mutant and found a complete loss of all DNA methylation (Figure 1D).These results suggest that the combination of these four SRA-SET mutants efficiently blocks both the DRM2 and CMT3 pathways and reinforces our hypothesis that SUVH9 and SUVH2 act in the DRM2 pathway.

### Maintenance of DRM2-Dependent Non-CG Methylation Requires SUVH9 or SUVH2 in a Locus-Specific Manner

**Figure 2:**
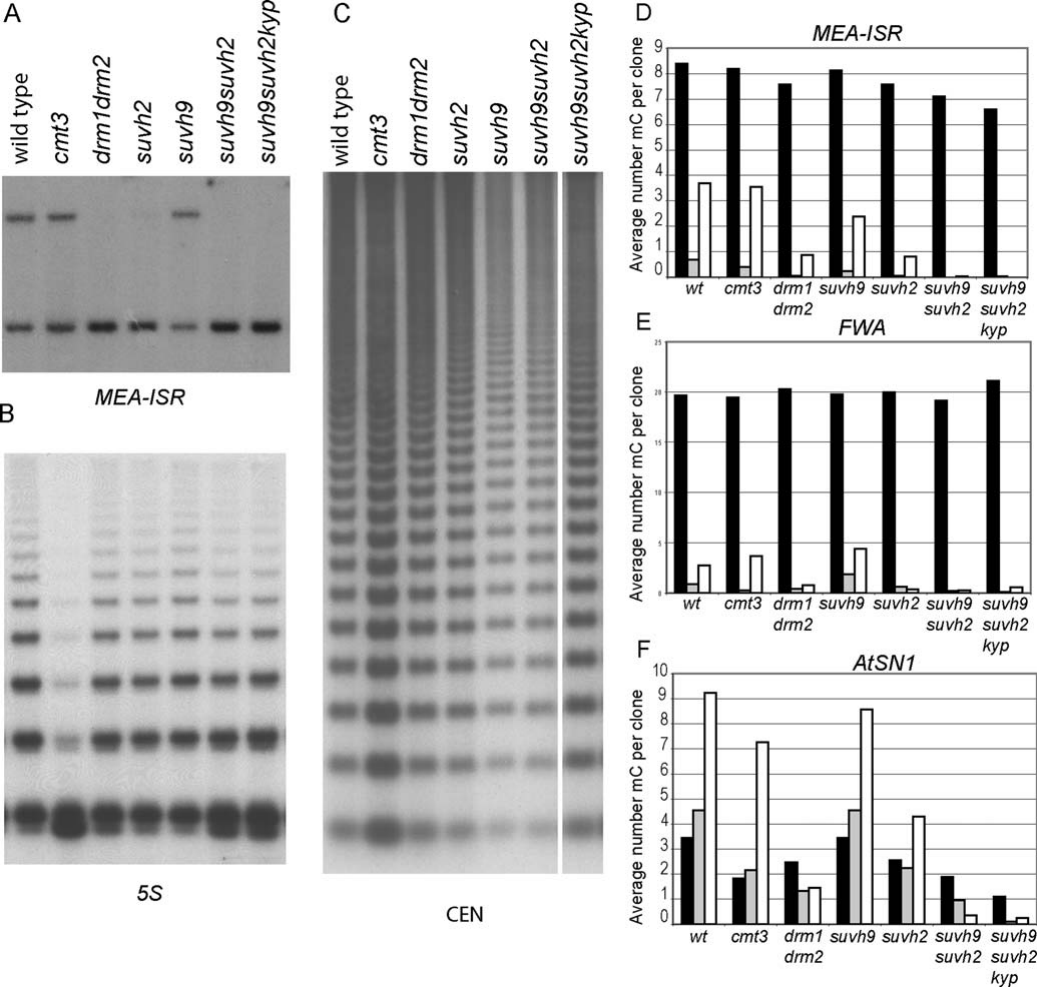
SUVH9 and/or SUVH2 are required for maintenance of non-CG methylation. A. Southern blot of MspI digested DNA using *MEA-ISR* probe. Upper band represents methylated DNA and lower band represents unmethylated DNA. B. Same blot as in A reprobed with 5S DNA probe. C. Similar blot probed with *CEN180* repeats. D. Bisulfite sequencing results of the *MEA-ISR* region expressed as the average number of methyl cytosines per clone (approximately 20 clones analyzed). Black (mCG), gray (mCHG), white (mCHH). E. Bisulfite results at *FWA*. F. Bisulfite results at *AtSN1*. See Figure S3 for alignments of all bisulfite sequence data.

Maintenance of non-CG methylation is performed by both the CMT3 pathway and the DRM2 pathway in a locus specific manner, i.e. some loci require just DRM2 or CMT3 and others require both enzymes to maintain non-CG methylation [20]. To determine the specificity of the SUVH9 and SUVH2 proteins, we assessed the level of methylation at several well-characterized loci. In contrast to the *SDC* locus where SUVH9 and SUVH2 act redundantly, we found that at two DRM2-dependent loci, *MEA-ISR* (tandem repeats near the *MEA* gene)[20] and FWA [21], non-CG methylation was mostly dependent on SUVH2 (compare Figure 2A, 2D and 2E with Figure 1D; see also Figure S2). At two loci whose non-CG methylation mostly depends on CMT3, the 180 base pair *CEN* repeats (Figure 2C) and the retrotransposon *Ta3* (data not shown), we did not observe an effect on either CG methylation or CHG methylation, further suggesting that SUVH9 and SUVH2 are specific to the DRM pathway. Methylation of *Ta3* and the *CEN* repeats is also strongly dependent on the MET1 pathway, indicating the SUVH9 and SUVH2 do not affect this pathway either. At the single copy SINE element, *AtSN1*, which is methylated by both DRM2 and CMT3, *suvh9* again had no effect while *suvh2* reduced non-CG methylation by 50%. At this locus *suvh9 suvh2* had an effect similar to *drm1 drm2*, and *suvh9 suvh2 kyp* reduced the CG and CHG methylation even further, to a similar level as observed in *drm1 drm2 cmt3* (Figure 2F and Figure S2). CHG methylation at the repetitive *5S* locus is strongly reduced in *cmt3* and slightly reduced in *drm1 drm2*, and we also observed a slight reduction in *suvh9 suvh2* and *suvh9 suvh2 kyp* mutants (Figure 2B). Together, these results are consistent with SUVH9 and SUVH2 functioning solely in the DRM2 pathway, where they act redundantly at some sites (*AtSN1*, *SDC*) and at other sites depend only on SUVH2 (*MEA-ISR*, *FWA*). Our results are not consistent with a previous report of a role of SUVH2 in control of MET1-dependent CG methylation [31].

### Expression of Epitope-Tagged SUVH9 and SUVH2 in Complementing Transgenic Plants

**Figure 3:**
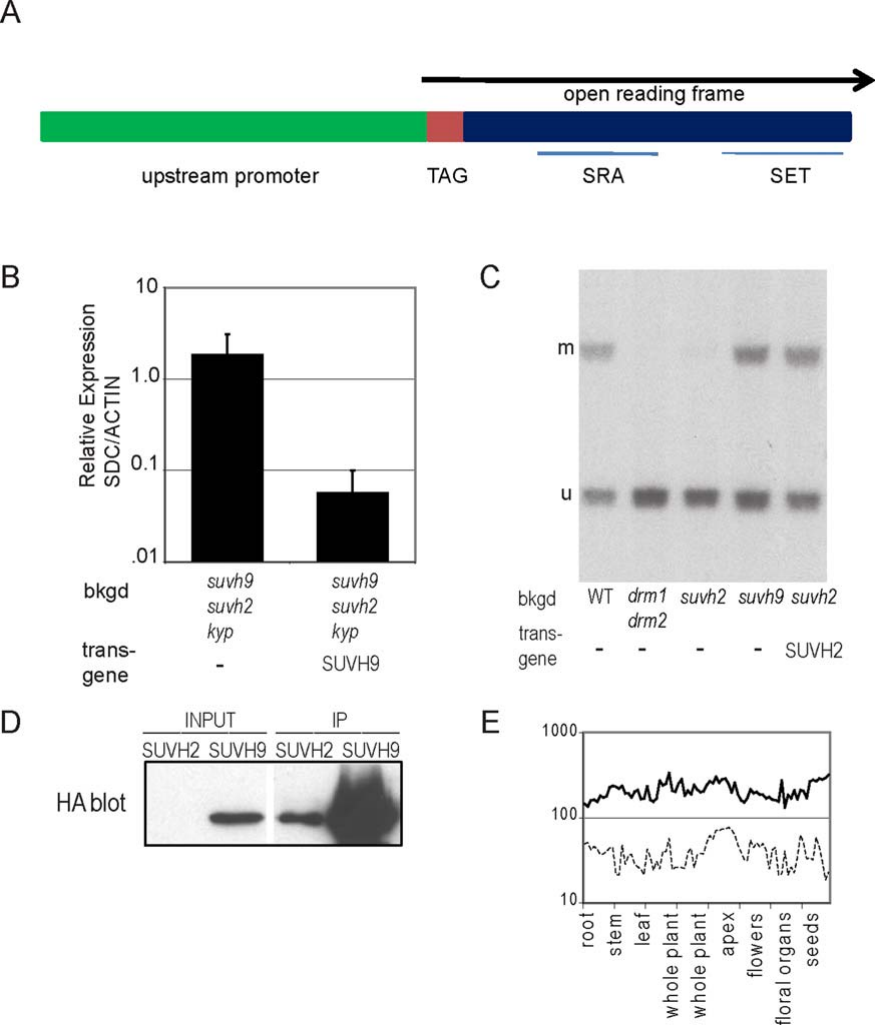
Epitope tagged SUVH9 and SUVH2 complement the *suvh9* and *suvh2* mutations. A. Diagram of epitope-tagged SUVH9 and SUVH2. The TAG is either 3xHA or 9xMyc as described in Material and Methods. B. Quantitative RT-PCR of *SDC* gene relative to *ACTIN* in *suvh9 suvh2 kyp* untransformed and transformed with myc-tagged SUVH9. C. Complementation of *suvh2* was tested by *MEA-ISR* southern blot using MspI digested DNA isolated from Columbia (wt), *drm1drm2*, *suvh2*, *suvh9*, and *suvh2* transformed with HA-tagged SUVH2. D. Western blot of HA- tagged SUVH2 and SUVH9 under control of their endogenous promoters in transgenic plants. Input refers to leaf extract before immunoprecipitation, IP after immunoprecipitation. E. Expression levels of SUVH9 and SUVH2 from different tissues as measured on microarrays.

In order to confirm that the phenotypes we observed were indeed caused by disruption of SUVH9 and SUVH2, we transformed plants with genomic clones containing amino-terminally tagged SUVH9 or SUVH2 under the control of their endogenous promoters (Figure 3A). To test for complementation of *suvh9*, myc-tagged *SUVH9* was transformed into *suvh9 suvh2 kyp* and expression of *SDC* was analyzed by RT-qPCR (Figure 3B). We observed a 30× reduction in *SDC* expression, indicating the tagged SUVH9 transgene was able to complement the mutant phenotype and allow resilencing of *SDC*. For *suvh2* complementation, we transformed HA-tagged *SUVH2* into a *suvh2* mutant and tested for DNA methylation at *MEA-ISR* (Figure 3C). Efficient complementation was observed as the reappearance of DRM2-dependent non-CG DNA methylation.

The epitope-tagged complementing transgenes also allowed us to evaluate the expression level of the SUVH9 and SUVH2 proteins in vivo. Expression of either myc-SUVH9 or HA-SUVH9 could be easily detected in leaves or flowers by western blot, but the level of expression of HA-SUVH2 was much lower and required immunoprecipitation in order to detect (Figure 3D). This is consistent with mRNA expression data from publicly available microarray experiments from 79 Arabidopsis tissues [32] that showed both SUVH9 and SUVH2 to be ubiquitously expressed, with SUVH9 showing a more than five-fold higher mRNA expression value than SUVH2 (Figure 3E). Although the microarray data is not particularly quantitative (because of noise introduced by the potentially different efficiencies of the probe sets for the two different genes) the large difference in signal suggests that SUVH9 is expressed more highly than SUVH2. These results suggest that the stronger effect of the *suvh2* single mutant as compared to the *suvh9* single mutant at loci such as *MEA-ISR* and *FWA* (Figure 2) cannot be explained simply by relative expression levels of the SUVH2 and SUVH9 and instead suggests that these proteins are functionally different.

### SUVH9 and SUVH2 Are Required for De Novo Methylation of the *FWA* Gene

**Figure 4:**
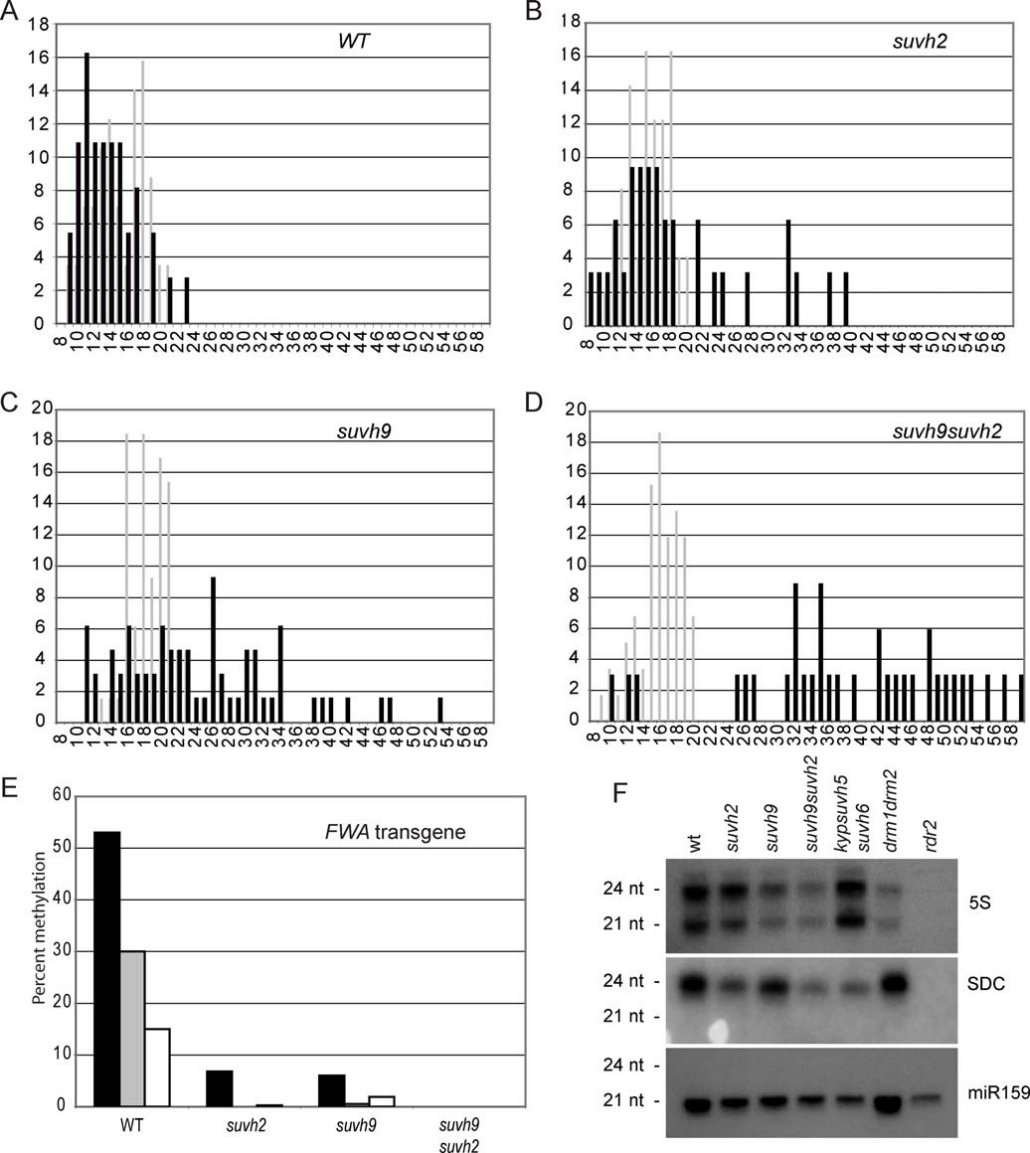
SUVH9 and SUVH2 are required for establishment of DNA methylation and silencing at *FWA*. A–D. Flowering time distributions shown as total leaf number of T1 populations of untransformed (light gray) or *FWA* transformed (black) plants of the indicated genotype. X-axes show total number of leaves at flowering time, and the Y-axes show the percentage of plants with the given leaf number. E. Bisulfite sequencing results of the *FWA* transgenes of late-flowering T1 plants. Black, CG methylation; gray, CHG methylation; white, CHH methylation. F. siRNA accumulation in various mutant backgrounds. MicroRNA 159 (mi159) was used as a loading control for SDC (siRNAs hybridizing to the tandem repeats) and 5S siRNAs.

A well-established method for assaying de novo methylation in Arabidopsis involves transforming plants with an unmethylated *FWA* transgene using Agrobacterium. The promoter region of the *FWA* gene contains two large and two small repeat sequences that are methylated upon integration into the plant genome, silencing the *FWA* gene and allowing for normal flowering time [21],[33]. In a *drm1 drm2* mutant background, the *FWA* transgene does not become methylated, allowing ectopic expression and causing late flowering. To investigate the role of SUVH9 and SUVH2 in the establishment of methylation, we assayed for de novo methylation using the *FWA* transformation assay and measured flowering time by counting the number of leaves produced before the transition to flowering. In the wild-type line (Columbia), flowering time changed very little upon transformation indicating efficient establishment of methylation on the *FWA* transgene (Figure 4A). In *suvh2*, most transformants flowered at the same time as untransformed plants, but 24% of the plants flowered later than the latest flowering plant in the untransformed control (Figure 4B), presumably due to an inability to methylate and silence the FWA transgene. In *suvh9*, 50% of the transformants flowered later than the untransformed control (Figure 4C) and in *suvh9 suvh2*, 91% of the transformants were late-flowering (Figure 4D).

To confirm the defect in *FWA* de novo DNA methylation, DNA from late-flowering T1 plants was isolated, and the methylation of the *FWA* transgene was analyzed by bisulfite sequencing. We found a significant reduction of DNA methylation in the *FWA* transgene from both a late-flowering *suvh2* T1 plant (flowered at 32 leaves) and a late-flowering *suvh9* T1 plant (flowered at 36 leaves), compared to the wild-type control (Figure 4E). The *suvh9 suvh2* late-flowering T1 plant had no detectable DNA methylation in the *FWA* transgene. These results suggest SUVH9 and SUVH2 act redundantly in the DRM2 pathway during establishment of methylation at *FWA*. Notably, these results are distinct from those obtained when assessing maintenance of non-CG methylation at *FWA*, which we find is dependent exclusively on SUVH2 (Figure 2E).

### SUVH9 and SUVH2 Function after siRNA Biosynthesis

Previous studies have shown that RNA Pol IVa, RDR2 and DCL3 are required to generate siRNAs and thus act upstream from RNA Pol IVb, AGO4, DRD1 and DRM2, which have only modest effects on siRNA abundance [25] We investigated whether SUVH9 and SUVH2 function before or after siRNA synthesis by analyzing their effect on siRNA levels at DRM2-dependent loci. Using siRNA blot analysis we analyzed the levels of siRNAs from two representative loci, *5S* and *SDC*. While the levels of siRNAs were somewhat reduced in some mutant backgrounds, they were not eliminated in *suvh9 suvh2* double mutants (Figure 4F). This was similar to what was observed with the *drm1 drm2* mutant and in contrast to the complete loss of siRNAs observed in *rdr2* (Figure 4F). These results indicate that SUVH9 and SUVH2 function at a point in the RNA-directed DRM2 DNA methylation pathway downstream of the initial biosynthesis of siRNAs. However, the modest reduction of siRNA levels in *suvh2 suvh9* may indicate a role in feedback between DNA methylation and the siRNA machinery, as has been suggested for other DRM2 pathway mutants [23].

### Histone Methylation in Heterochromatin Does Not Change in *suvh9 suvh2*

**Figure 5:**
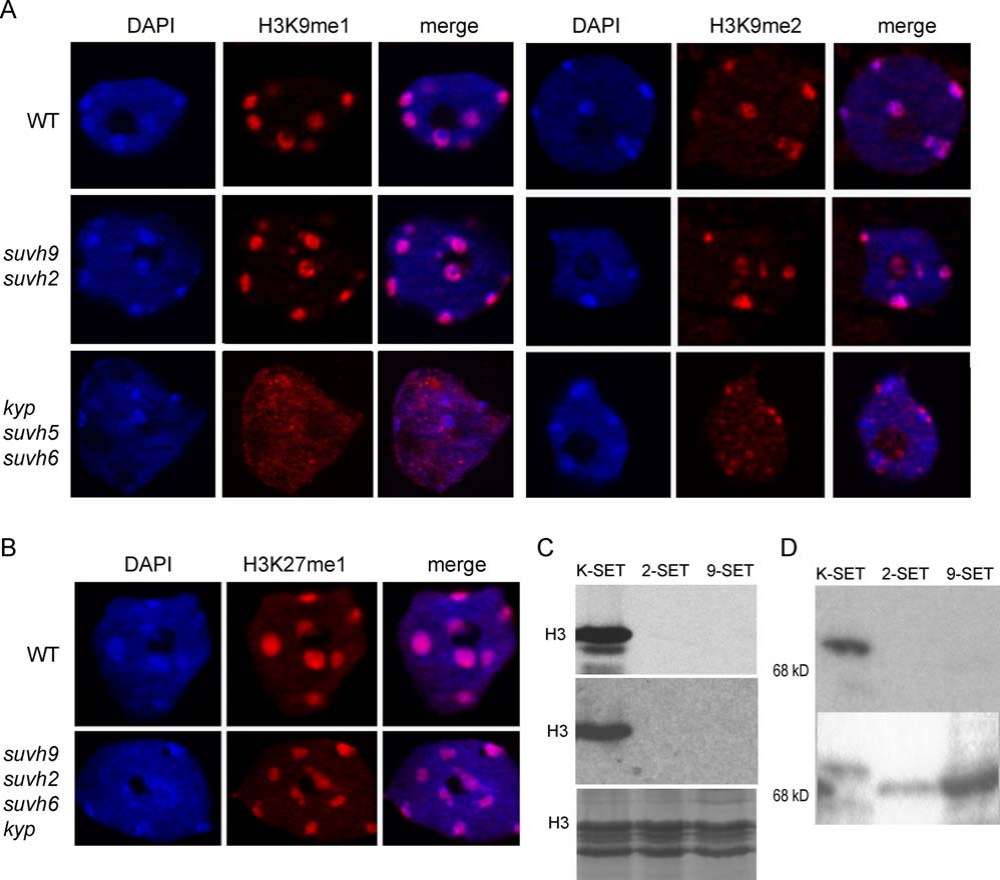
SUVH9 and SUVH2 may be inactive for histone methyltransferase activity. A–B. Immunofluorescence of H3K9me1, H3K9me2 and H3K27me1 compared to DAPI in nuclei isolated from Columbia (wt), *suvh9 suvh2*, *kyp suvh5 suvh6* and *suvh9 suvh2 suvh6 kyp*. C. Histone methylation assays using GST fusion proteins and either calf thymus histones (top panel, autoradiogram) or Arabidopsis nucleosomes (middle panel, autoradiogram) as substrates. Calf thymus histones are shown in bottom panel (protein stain). D. ^3^H-AdoMet crosslinking to GST fusion proteins. Top panel autoradiogram, bottom panel stained proteins.

The SET domains of SUVH9 and SUVH2 align closest to the H3K9 methyltransferases and yet are highly divergent (Figure S1B), suggesting that SUVH9 and SUVH2 could have evolved a function different from the other H3K9 methyltransferases. To determine the specificity of SUVH9 and SUVH2 SET domains, histone methylation marks associated with heterochromatin and gene silencing were examined by immunostaining of nuclei using well-characterized antibodies. Previous studies reported a decrease in H3K9me1, H3K9me2, H3K27me1, H3K27me2 and H4K20me1 in *suvh2* background [31]. However, we could not find any significant differences using well-characterized antibodies to H3K9me1 or H3K9me2 in *suvh2*, *suvh9* (data not shown), or in *suvh9 suvh2* nuclei compared to the wild-type Columbia line (Figure 5A). Consistent with previous reports showing that the KYP, SUVH5, and SUVH6 proteins add one or two methyl groups to H3K9 in vitro and all play a role in targeting CMT3 [17],[19], H3K9me1 and H3K9me2 staining of chromocenters in *kyp suvh5 suvh6* nuclei was significantly reduced (Figure 5A). We next tested H3K27me1, a mark found in heterochromatin, in the quadruple mutant *suvh9 suvh2 suvh6 kyp* and found no difference compared to wild-type Columbia (Figure 5B). H3K27me2 is found only in euchromatin (data not shown) and H4K20me has not been detected by mass spectrometry [34] nor could we detect a signal by immunofluorescence (data not shown). Thus, SUVH9 and SUVH2 do not appear to be required for the overall presence of the main histone methylation marks associated with gene silencing and may act either in a more locus-specific manner or on non-histone substrates.

To further explore the possibility that SUVH9 and SUVH2 are histone methyltransferases, various in vitro histone methylation assays were conducted. Amino-terminal glutathione S-transferase fusions with SUVH9 and SUVH2 containing the Pre-SET and SET domains were constructed and purified from *E. coli* along with KYP-SET as a positive control. With KYP-SET we readily detected methylation of H3 using either calf thymus histones or Arabidopsis nucleosomes (Figure 5C). However, using a variety of different buffers we did not detect activity with either SUVH9-SET or SUVH2-SET (Figure 5C). Since it is possible that SUVH9 or SUVH2 must be in a complex for activity, affinity-tagged SUVH9 and SUVH2 were immunoprecipitated from Arabidopsis extracts prepared from complementing transgenic lines, and immunoprecipitates were assayed for histone methylation activity using either calf thymus histones or Arabidopsis nucleosomes, but no activity above background levels was observed (data not shown).

Finally we tested the ability of SUVH9 or SUVH2 to bind S-adenosylmethionine (AdoMet), which would indicate they have the potential to be active methyltransferases. AdoMet binding to SET domains can be detected by crosslinking with ultraviolet light using ^3^H-AdoMet [35]. While binding of AdoMet to KYP-SET was easily detected, binding to either SUVH9-SET or SUVH2-SET was undetectable (Figure 5D). The lack of binding to AdoMet suggests the possibility that SUVH9 and SUVH2 may not be active methyltransferases, or may require other factors to be active.

### SUVH9 and SUVH2 Are Distinguished by Their DNA-Binding Specificity

**Figure 6:**
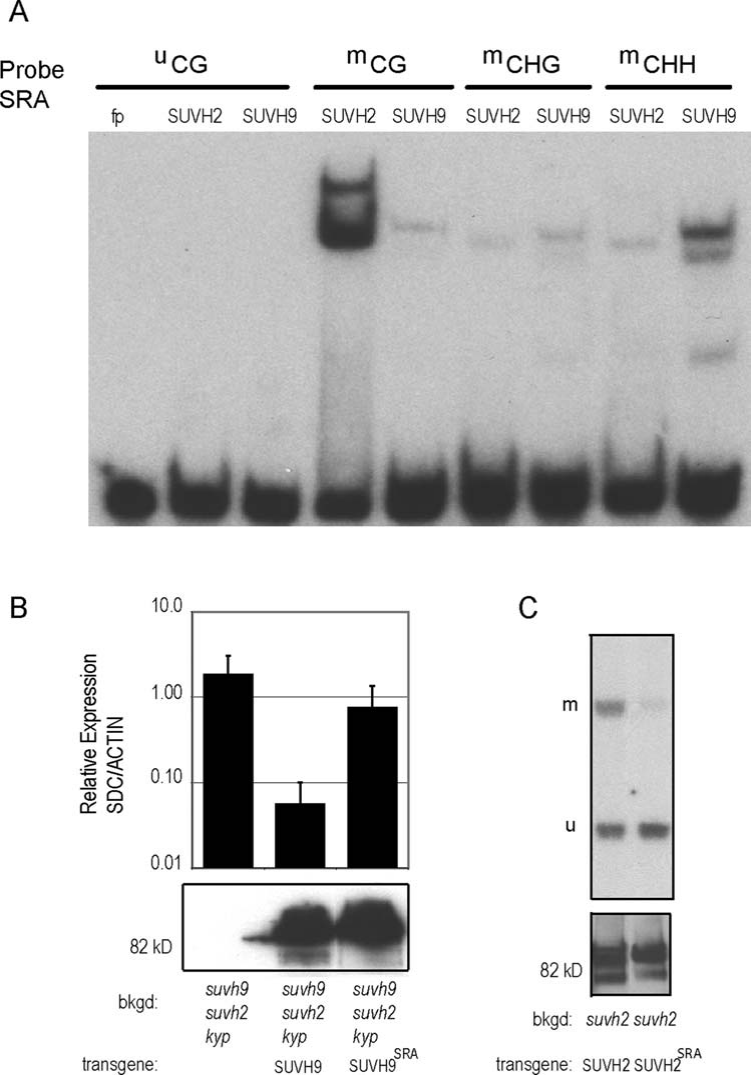
SRA domains of SUVH9 and SUVH2 bind to methylated CHH and CG sequences, respectively. A. Mobility shift assays using either GST-SUVH2-SRA (SUVH2) or GST-SUVH9-SRA (SUVH9) and either unmethylated CG oligonucleotide (^u^CG), methylated CG (^m^CG), methylated CHG (^m^CHG) or methylated CHH (^m^CHH) as probe. B. Upper panel: Quantitative RT-PCR of the *SDC* gene relative to *ACTIN* was measured in *suvh9 suvh2 kyp* lines containing the following stable transgenes: myc-tagged SUVH9 (SUVH9) or myc-tagged SUVH9-SRA mutant (S252F; SUVH9^SRA^). Lower panel: Western blot of transgenic plant extracts probed with myc antibody. C. Upper panel: Complementation of *suvh2* was tested by *MEA-ISR* Southern blot using MspI digested DNA isolated from *suvh2* lines containing the following stable transgenes: HA-tagged SUVH2 or HA-tagged SUVH2-SRA mutant (E262K; SUVH2^SRA^). Lower Panel: Western blot of immunoprecipitated protein from *suvh2* transgenic plants using HA antibody as probe.

In addition to the SET domain, SUVH9 and SUVH2 also possess an SRA domain that could be important for its function in the DRM2 pathway, similar to what has been observed in the CMT3 and MET1 pathways. SRA-domains are methyl-cytosine binding domains that vary in their sequence specificity [9],[10],[12],[13]. To determine the sequence specificity for the SUVH9 and SUVH2 SRA domains, GST-SRA fusions were expressed and purified from bacteria. We measured binding to various double-stranded oligonucleotide substrates in the presence of 1000× molar excess of unmethylated competitor using mobility shift assays. The SUVH9 SRA showed a strong preference for methylated CHH over CHG or CG oligonucleotides with little affinity for hemi-methylated DNA, whereas no binding was detected to unmethylated DNA (Figure 6A and Figure S4). In contrast, the SUVH2 SRA showed strong binding to methylated CG sites, with a very low affinity for methylated CHG, CHH, hemi-methylated or unmethylated DNA (Figure 6A and Figure S4). These two SRA domains, therefore, have specificities that distinguish them from each other as well as from the other SRA-domains that have been characterized to date.

If the methyl DNA-binding activity of SUVH9 and SUVH2 is important for DRM2 activity, then their SRA domains should be necessary for successful genomic complementation. To test this, mutations analogous to those isolated in the KYP SRA-domain [12] were introduced into the otherwise complementing SUVH9 and SUVH2 epitope-tagged transgenes, and mutant transgenic lines were used in complementation experiments. RT-qPCR of the *SDC* gene revealed that the SRA mutation in SUVH9 (S252F: equivalent to KYP S200F) resulted in an increase of expression 10 fold above what was observed in *suv9 suvh2 kyp/SUVH9* (Figure 6B). A SUVH2 SRA mutant (E262K: equivalent to KYP E208K) also showed a loss of activity as measured by methylation of MEA-ISR (Figure 6C). In neither case did the mutation destabilize the protein in plants (Figure 6B and Figure 6C, lower panels). These results suggest that the SRA domains of SUVH9 and SUVH2 are critical to their function in DRM2-mediated DNA methylation.

## Discussion

DRM2 is the major enzyme responsible for de novo methylation and maintenance of non-CG methylation in Arabidopsis. siRNAs produced by RNA Pol IV, RDR2, and DCL3 and bound by AGO4 are necessary for targeting DRM2 to specific sequences resulting in DNA methylation [36],[37]. This process has also been shown to involve two SNF2 homologs, DRD1 and CLASSY, and a chromosome architectural protein (DMS3) homologous to the hinge region of SMC [37],[38]. We show here that SUVH9 and SUVH2 are also required for RNA-directed DNA methylation. Knocking out *SUVH9* and/or *SUVH2* blocks maintenance of non-CG methylation by DRM2 at multiple loci and prevents de novo methylation of the *FWA* transgene. Furthermore, we show that these two proteins function after the initial biosynthesis of siRNAs, suggesting they may be involved in a later step in the DRM2 pathway.

SUVH9 and SUVH2 have two notable domains: the SRA methyl-cytosine DNA binding domain and the SET methyltransferase domain. The SET domain aligns closest to the H3K9 methyltransferases, but one of the most conserved sequences in the carboxy-terminal region of the SET domain (RFxNHxCxPN) is highly diverged, replaced with a sequence that is highly conserved between SUVH9 and SUVH2 homologs in rice and poplar (CYxSHSxxPN; Figure S1). SUVH9, SUVH2, and their homologs are also missing the post-SET domain. This conservation suggests that this region is important functionally, but may have a distinct activity compared to the other SUVH proteins. Conflicting results have been reported regarding the histone methyltransferase activity of these enzymes. One report found no histone methyltransferase activity in SUVH9 and SUVH2 GST fusion proteins using calf thymus histones as substrates, whereas activity for SUVH4 (KYP), SUVH5 and SUVH6 fusions was easily observed [19]. In a second report, recombinant nucleosomes were used as a substrate and SUVH2 activity was detected on both H3 and H4 [31]. We repeated these assays for both SUVH2 and SUVH9 and did not observe activity in vitro using either histones or nucleosomes as a substrate. Another possibility is that SUVH9 and SUVH2 have diverged such that they methylate non-histone protein substrates [39]–[41]. One obvious candidate is DRM2, however in vitro assays using DRM2 as a substrate were also negative (data not shown). Furthermore, we could detect binding of AdoMet (the methyl donor for methyltransferase enzymes) to KYP but not to SUVH9 or SUVH2, suggesting that these two proteins may have lost methyltransferase activity.

It is possible that SUVH9 and SUVH2 require another protein for activity, or possibly dimerize in vivo to create an active site [42]–[45]. For instance, the Drosophila SU(VAR)3-9 is most active as a dimer and requires the amino terminus to dimerize. We immunoprecipitated SUVH9 and SUVH2 from plants and assayed their ability to methylate histones or nucleosomes, but again no activity was observed. Thus, while our results cannot rule out that SUVH9 and SUVH2 are active methyltransferases, they do raise the possibility that the SET domains are acting in a different manner. For instance, there are several examples in other systems where catalytically inactive homologs are important [46],[47].

The SUVH9 and SUVH2 proteins also contain SRA domains that are active in binding methylated DNA and differ from each other in their sequence specificity. SUVH9 prefers methylated CHH, binding with a higher affinity when a G residue is not located in the two adjacent positions. This differs from KYP and SUVH6 which preferentially bind to CHG [12]. Biologically this preference for CHH makes sense because DRM2 is essential for maintaining CHH residues. SUVH2 binding specificity for methylated CG sites was more surprising. DRM2 from tobacco is active in vitro, and preferentially methylates CHH and CHG and only methylates CG residues at a low frequency [48]. Hence, SUVH2 appears to be binding to a sequence not commonly maintained by DRM2. However, it has been previously shown that eliminating CG methylation in *met1* mutant lines results in loss of non-CG methylation at certain loci [20],[30],[49]. Thus, one possibility is that SUVH2 may function by linking CG methylation and non-CG methylation.

The difference in binding specificity of the SRA domains of SUVH2 and SUVH9 correlates well with the loci that are preferentially affected in the mutants. Both *MEA-ISR* and *FWA* are rich in methylated CG residues (8 mCGs:4 methylated non-CGs in *MEA-ISR*; 20 mCGs:4 methylated non-CGs in *FWA*; see Figure 2) and show a strong dependence on SUVH2 which preferentially binds to methylated CGs. On the other hand, *SDC* and *AtSN1* are more heavily CHH and CHG methylated (5 mCGs:9 methylated non-CGs at *SDC*; 3 mCGs:14 methylated non-CGs at *AtSN1*; see Figure 1D and Figure 2E) and only show a strong reduction of methylation in the *suvh9 suvh2* double mutant background, suggesting less of a dependence on SUVH2.

An attractive model to explain the role of SUVH9 and SUVH2 in de novo methylation may be that they function to retain DRM2 at methylated regions immediately after the establishment of methylation (even in initiation assays DNA methylation must be maintained through many rounds of replication and mitoses before the DNA methylation is analyzed). One possibility is that SUVH2 could retain DRM2 at sites rich in methylated CG and SUVH9 could do the same at sites rich in non-CG methylation. These SRA proteins would then provide a link between establishment of methylation and maintenance methylation. Alternatively, SUVH9 and SUVH2 may recruit or retain a component of the DRM2 pathway which is needed for DRM2 activity. It is also possible that SUVH2 and SUVH9 directly participate in the silencing of DNA methylated genes, and that some of the loss of DNA methylation observed in *suvh2* and *suvh9* mutants is due to secondary effects of the loss of gene silencing or other chromatin marks. Regardless of the specific mechanism involved, these results show that each of the major methylation systems in Arabidopsis require an SRA-domain protein for function. CG methylation by MET1 involves VIM1 (an SRA protein homologous to UHRF1 which specifically binds hemimethylated CG sites; [10],[13]) CMT3 is dependent on KYP, which specifically binds methylated CHG; and DRM2 requires SUVH9 and SUVH2, which bind to methylated CHH or methylated CG, respectively.

## Material and Methods

### Plant Materials

The *drm1 drm2 cmt3* and *drm1 drm2 kyp* triple mutants were generated in the Columbia background and have been described previously [29]. The *kyp* mutant is Salk T-DNA_041474 and was described previously [12]. SUVH5 mutant T DNA was obtained from GABI-Kat (line 263C05, [50]) and disrupts the open reading frame at amino acid 40. SUVH6 mutant T-DNA was obtained from Syngenta (Garlic_1244_F04.b.1a; [27]) and disrupts the open reading frame in the middle of the pre-SET domain. SUVH2 mutant T-DNA (Salk _079574.17.40) disrupts the open reading frame at amino acid 101 and has been previously characterized [31]. The SUVH9 mutant T-DNA (Salk_048033) disrupts the open reading frame at amino acid 43. Plants were grown under continuous light for scoring the SDC over-expression leaf-curling phenotype and under long days for measuring flowering time. *suvh9 suvh2 kyp* morphological phenotypes were examined over several generations of inbreeding and differences between generations were not observed.

### RNA Analysis

Total RNA was extracted from several pooled 3 week-old plants using Trizol reagent (Invitrogen) and analyzed by RT-qPCR. Two to three biological replicas were sampled and standard deviations determined. Primers for *SDC* amplification were JP3395 and JP3396 (primers are listed in Table S1) using SYBR green and *ACTIN* amplification was done using JP2452 and JP2453 using a Taqman probe (M17). Small RNAs were extracted from flowers and analyzed by Northern blotting as previously described [51].

### DNA Methylation

Approximately 0.5 to 1.0 µg of genomic DNA (from flowers and rosette leaves) was bisulfite treated using the EZ DNA Methylation-Gold kit (Zymo research cat. No. D5005). The *MEA-ISR*, *AtSN1*, and *SDC* loci were amplified using 1 µl of bisulfite treated DNA in a 50 ul PCR reaction using Ex Taq polymerase (Takara Cat. No. TAK RR001 A) and JP5392, JP5393 (*MEA-ISR*); JP1821, JP1822 (*AtSN1*); JP4039, JP4045 (*SDC*). FWA methylation was determined from DNA isolated from rosette leaves for bisulfite treatment and amplified with JP2004, JP4423. PCR products were TA cloned in to pCR2.1 (Invitrogen cat No. K4500-01) and approximately 20 individual clones were sequenced using the M13 reverse primer by the High Throughput Genomics Unit at the University of Washington. See Figure S3 for alignments.

For Southern blots, 3–5 µg of genomic DNA was run on 1% agarose gels, transferred to Hybond N+ membranes, blocked and washed according to manufacture instructions (GE Healthcare). Membranes where probed using PCR products radiolabeled with alpha ^32^P-dCTP using the Megaprime DNA Labeling System (GE cat. No. RPN1606). *MEA-ISR*, *Ta3* and *CEN180* probes were amplified as described previously [20],[52].

### SUVH9 and SUVH2 Constructs

GST fusions were made using either the Gateway cloning system from Invitrogen or the pGEX-4T1 plasmid from GE Healthcare. The GST fusion containing the KYP SET was described previously [12]. pLJ248 is pDEST15 containing amino acids 387–650 of SUVH9, the entire carboxy-terminal end of the protein (GST-preSET-SET construct: abbreviated 9-SET). pLJ205 is pDEST15 containing amino acids 387–651 of SUVH2 (GST-preSET-SET contruct: abbreviated 2-SET). pLJ176 is pDEST15 containing amino acids 137–356 of SUVH9 (GST-SRA: abbreviated 9-SRA) and pLJ242 is pGEX-4T1 with amino acids 201 to 400 of SUVH2 (GST-SRA: abbreviated 2-SRA). The GST fusion proteins were expressed in BL21 AI cells and purified as described previously except that the final buffer for the GST-SET proteins was 50 mM Tris-HCl pH 8.0, 50 mM NaCl, 1 mM DTT, 40% glycerol and the GST-SRA proteins were dialyzed into 50 mM Tris-HCl pH 6.8, 300 mM NaCl, 1 mM DTT, 40% glycerol.

Epitope-tagged protein constructs were made using a modified Gateway cloning system for expression in plants. Specifically, the biotin ligase gene (BirA) under the control of the ACTIN promoter was added into the single Sbf1 site of the pEarleyGate302 destination vector [53] and the C-terminal Flag tag was removed by site directed mutagenesis using JP 4225 and JP 4226 primers (JP746; Figure S5). 1.4 kb of genomic DNA upstream the SUVH9 ORF and the entire ORF was cloned into pENTR. A *Kpn* I restriction site was introduced at the ATG and either a 9xMyc epitope tag (pLJ217) or a 3xHA (pLJ214) epitope tag was introduced. Both of these tags also contain the biotin ligase recognition peptide (BLRP) and a 3C protease site. These tagged constructs were then recombined into JP746 and introduced into Agrobacterium strain AGLO.

pLJ213 contains 2.1 kb upstream of the SUVH2 ORF, the ORF, and 1 kb downstream of the ORF (SUVH2 contains an intron and an untranslated exon in the 3′ end) with the BLRP-3C-3xHA epitope tag inserted at the ATG via an introduced *Kpn* I site in vector JP746. Mutations in the SRA domain were introduced using QuikChange Kit (Stratagene). Plasmids were transformed into the Agrobacterium strain AGLO and then introduced into Arabidopsis using the floral dip method of transformation. Transformed lines were selected with Basta.

### Immunofluorescence

Nuclei were isolated and stained as described in [12]. The H3K9me1 and H3K9me2 antibodies used in this study were a gift from Thomas Jenuwein (lot #4858 and lot #4677, respectively). The H3K9me3 was obtained from Abcam (#8898-100). The H3K27me1 antibody was obtained from Upstate Biotechnology (#24439). Immuno staining was done as described previously with the additional use of a Zeiss ApoTome [53].

### In Vitro Assays

Histone methylation assays were done as described in [54]. Specifically, 8 ug GST fusion proteins purified from *E. coli* were incubated in 50 mM Tris pH 8.8, 20 mM KCl, 10 mM MgCl_2_, 10 mM β-mercaptoethanol, 250 mM sucrose and ^3^H-S-AdoMet (GE Healthcare, #TRK581) with either 10 ug of calf thymus histones or Arabidopsis nucleosomes [55]. Reaction mixtures were incubated at room temperature for 3 hours before separating proteins on 15% polyacrylamide gel. Incorporation of tritium was detected by autoradiography. AdoMet crosslinking was done as described in [35] using approximately 15 ug of purified GST-fusion proteins. The DNA probes used in the electrophoretic mobility shift assays were described previously [12]. GST-SUVH2-SRA (60 nM final concentration) or GST-SUVH9-SRA (0.4 nM final concentration) was incubated with ^32^P-labeled probe in the presence of 1000× molar excess of unmethylated DNA as competitor in buffer (25 mM Tris, pH 6.8, 10 mM MgCl_2_, 1 mM DTT, 5% glycerol, 0.4 mg/ml BSA) for 30 minutes. Samples were electrophoresed on an 8% polyacrylamide gel, which was then fixed in 5% acetic acid and dried. ^32^P-labeled DNA was detected by autoradiography. GST-fusion proteins isolated from *E. coli* vary in the amount of active protein, so it is unclear whether SUVH9 binds methylated DNA with a higher affinity than SUVH2.

## Supporting Information

Figure S1 A. Phylogenetic relationships between plant SUVH proteins. The tree was constructed using Unweighted Pair Group Metho with Arithmetic Means (UPGMA). Bootstrap valued were calculated from 1000 replicates. Protein sequences were obtained from the Plant Chromatin Database (www.chromdb.org): (*Arabidopsis thaliana*: A) SUVH1 (At5g04940), SUVH2 (At2g33290), SUVH3 (At1g73100), KYP (At5g13960), SUVH5 (At2g35160), SUVH6 (At2g22740), SUVH7 (At1g17770), SUVH8 (At2g24740), (*Populus trichocarpa*: P) SDG915, SDG949, SDG939, SDG940 (*Oryzae sativa*: R; numbers refer to Plant Chromatin Database ID) SDG2211, SDG726, SDG714, (*Homo sapiens*: H) G9a. Poplar and Rice homologs of SUVH1, SUVH3, SUVH7 and SUVH8 are not included. B. Sequence alignment of C-terminal region of SET domain. Non-conserved sequences are shaded in purple.
(0.64 MB TIF)

Figure S2 DNA methylation data derived from bisulfite sequencing expressed as percentage of methylation. Black bars represent CG methylation, gray bars represent CHG methylation and white bars represent CHH methylation. A. Data from Figure 1D. B. Data from Figure 2D. C. Data from Figure 2F. D. Data from Figure 2E.
(0.96 MB TIF)

Figure S3 Bisulfite sequence alignments. Data previously reported in Henderson and Jacobsen [30] was not included. Top sequence represents unconverted genomic sequence.
(13.9 MB PDF)

Figure S4 Mobility shift assays using either GST-SUVH2-SRA (2) or GST-SUVH9-SRA (9) and either unmethylated CHG oligonucleotide (uCHG), unmethylated CHH (uCHH), hemimethylated CG (hCG) or hemimethylated CHG (hCHG) as probe.
(0.53 MB TIF)

Figure S5 Map of binary vector JP746. LB (T-DNA left border), RB (T-DNA right border), BaR (basta resistance), attL2 and attR2 (attachment sites), ccdB (toxic gene), BirA (Biotin ligase gene), OCS (3′ end of the octopine synthase gene).
(0.08 MB TIF)

Table S1 Primer sequences.
(0.03 MB DOC)
