# Supplementary figures and images for: SRA-Domain Proteins Required for DRM2-Mediated De Novo DNA Methylation
Source: PLoS Genet. 2008 Nov 28;4(11):e1000280. doi: 10.1371/journal.pgen.1000280 (PMC2582956; doi:10.1371/journal.pgen.1000280)

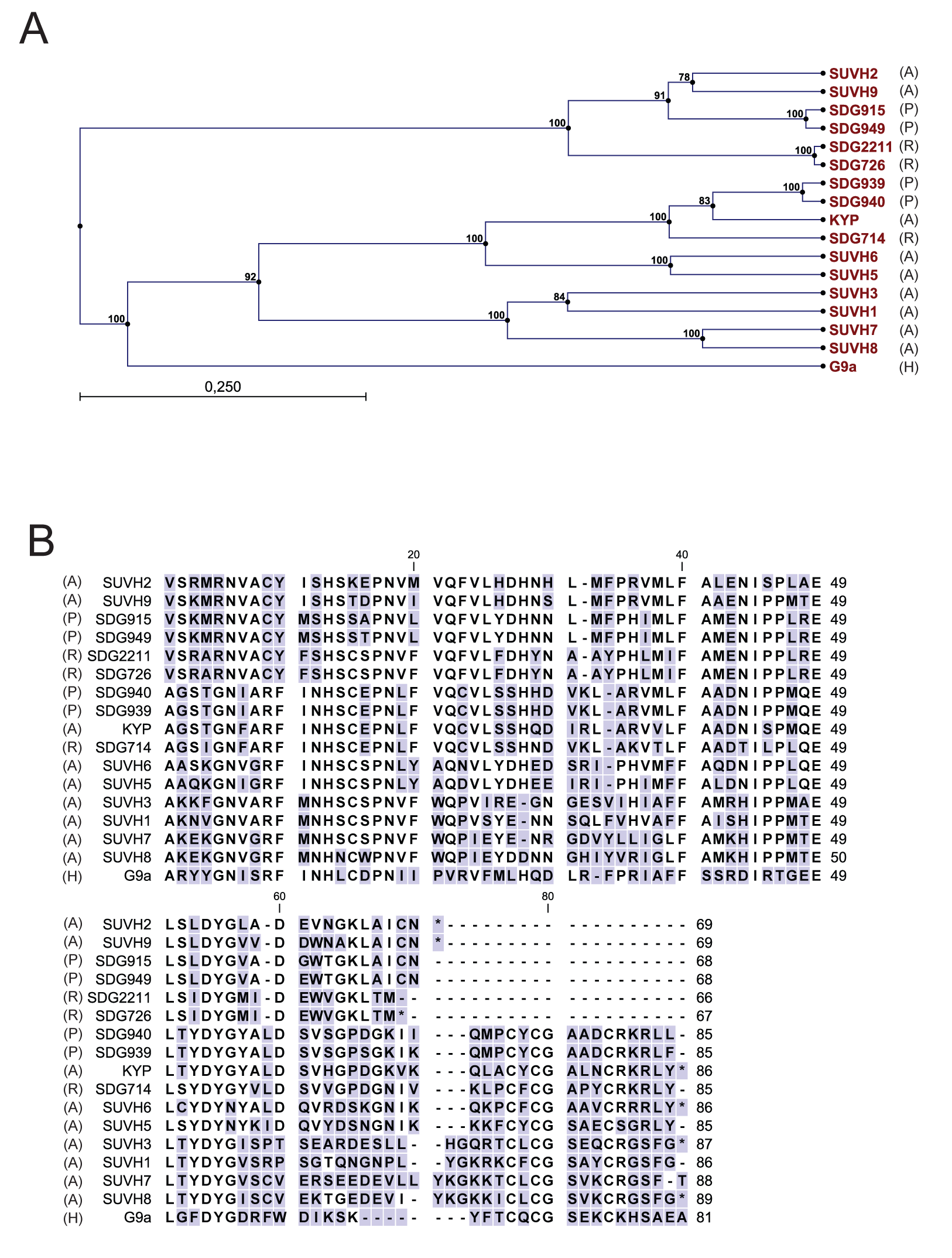

Supplement: Figure S1 — A. Phylogenetic relationships between plant SUVH proteins. The tree was constructed using Unweighted Pair Group Metho with Arithmetic Means (UPGMA). Bootstrap valued were calculated from 1000 replicates. Protein sequences were obtained from the Plant Chromatin Database (www.chromdb.org): (Arabidopsis thaliana: A) SUVH1 (At5g04940), SUVH2 (At2g33290), SUVH3 (At1g73100), KYP (At5g13960), SUVH5 (At2g35160), SUVH6 (At2g22740), SUVH7 (At1g17770), SUVH8 (At2g24740), (Populus trichocarpa: P) SDG915, SDG949, SDG939, SDG940 (Oryzae sativa: R; numbers refer to Plant Chromatin Database ID) SDG2211, SDG726, SDG714, (Homo sapiens: H) G9a. Poplar and Rice homologs of SUVH1, SUVH3, SUVH7 and SUVH8 are not included. B. Sequence alignment of C-terminal region of SET domain. Non-conserved sequences are shaded in purple. (0.64 MB TIF) [file pgen.1000280.s001.tif]

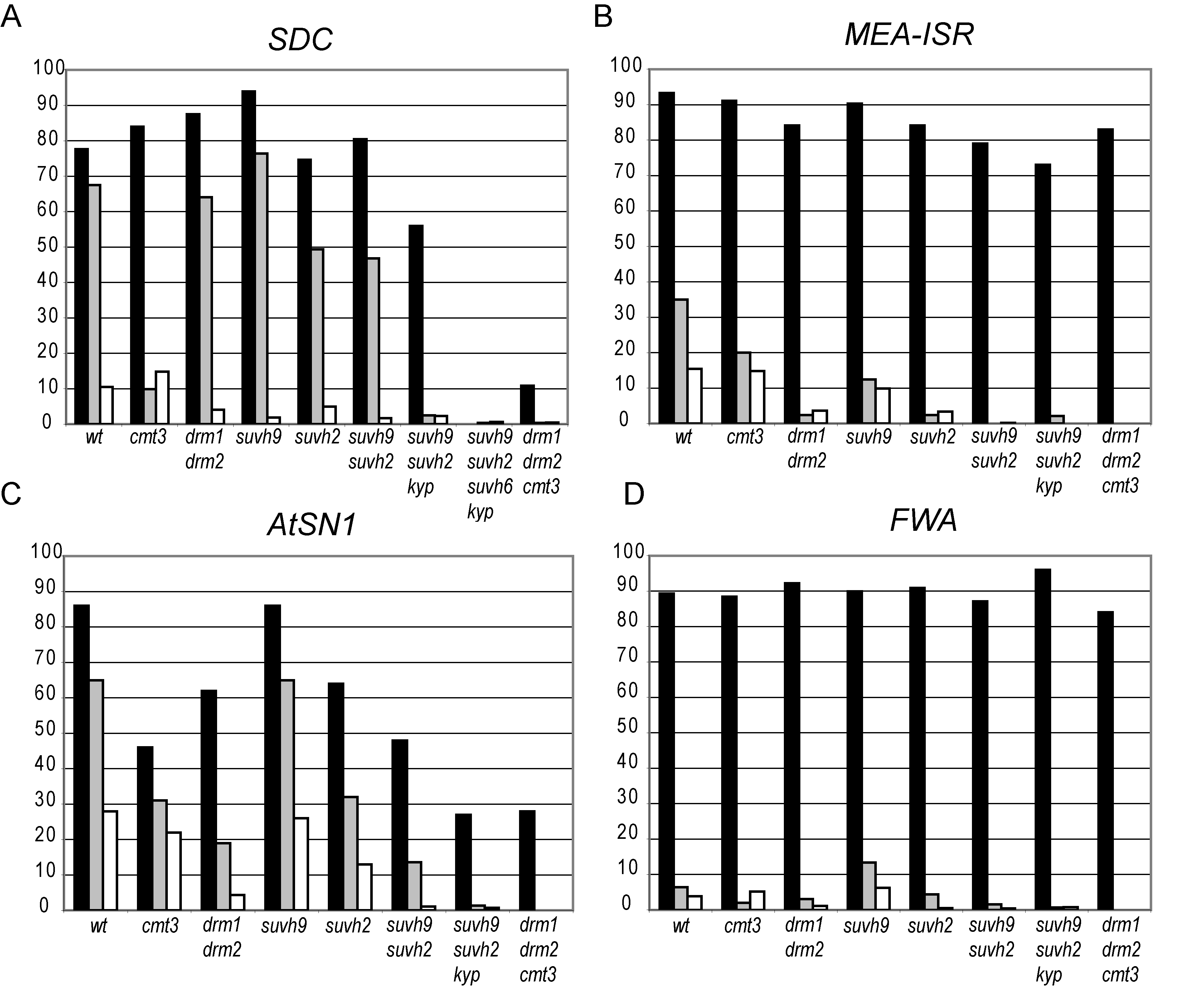

Supplement: Figure S2 — DNA methylation data derived from bisulfite sequencing expressed as percentage of methylation. Black bars represent CG methylation, gray bars represent CHG methylation and white bars represent CHH methylation. A. Data from Figure 1D. B. Data from Figure 2D. C. Data from Figure 2F. D. Data from Figure 2E. (0.96 MB TIF) [file pgen.1000280.s002.tif]

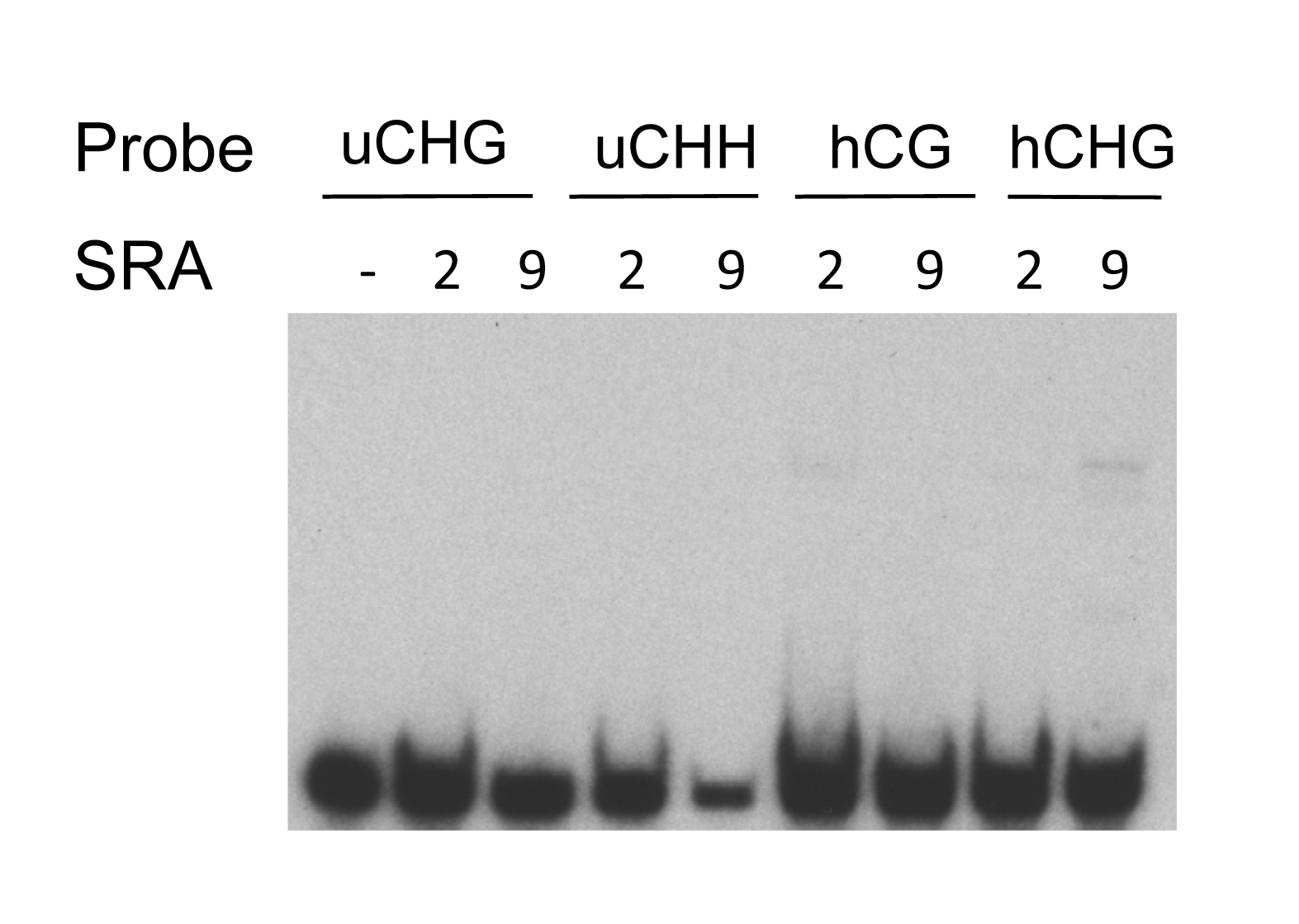

Supplement: Figure S4 — Mobility shift assays using either GST-SUVH2-SRA (2) or GST-SUVH9-SRA (9) and either unmethylated CHG oligonucleotide (uCHG), unmethylated CHH (uCHH), hemimethylated CG (hCG) or hemimethylated CHG (hCHG) as probe. (0.53 MB TIF) [file pgen.1000280.s004.tif]

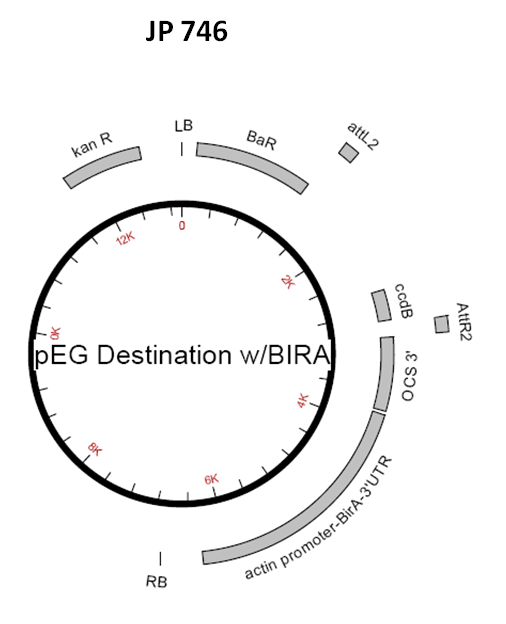

Supplement: Figure S5 — Map of binary vector JP746. LB (T-DNA left border), RB (T-DNA right border), BaR (basta resistance), attL2 and attR2 (attachment sites), ccdB (toxic gene), BirA (Biotin ligase gene), OCS (3′ end of the octopine synthase gene). (0.08 MB TIF) [file pgen.1000280.s005.tif]
