# Supplementary material for: SRA-Domain Proteins Required for DRM2-Mediated De Novo DNA Methylation
Source: PLoS Genet. 2008 Nov 28;4(11):e1000280. doi: 10.1371/journal.pgen.1000280 (PMC2582956; doi:10.1371/journal.pgen.1000280)
Supplement: Figure S3 — Bisulfite sequence alignments. Data previously reported in Henderson and Jacobsen [30] was not included. Top sequence represents unconverted genomic sequence. (13.9 MB PDF) [file pgen.1000280.s003.pdf]

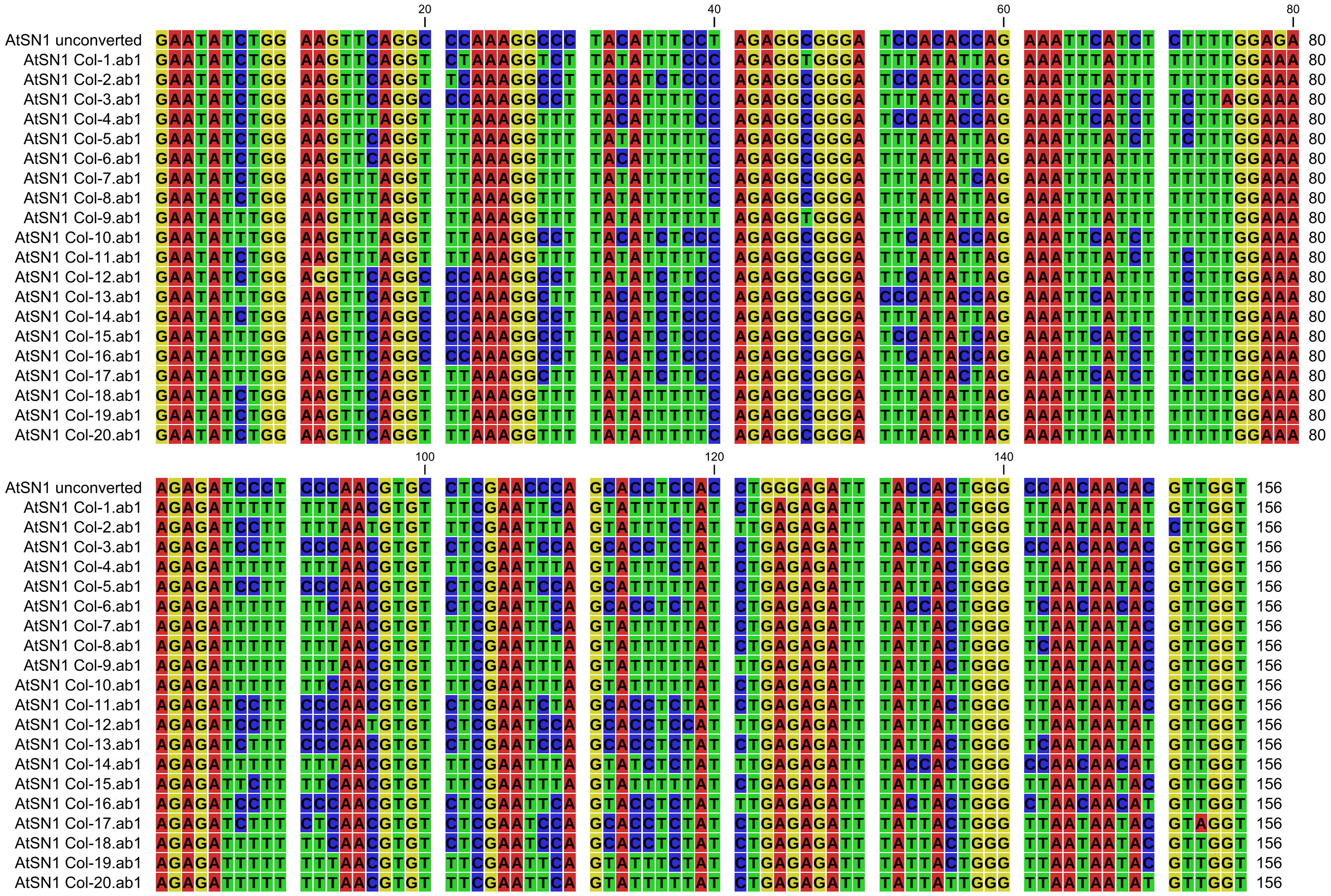

20
40
60
80
AtSN1 unconverted
AtSN1 Col-1.ab1
AtSN1 Col-2.ab1
AtSN1 Col-3.ab1
AtSN1 Col-4.ab1
AtSN1 Col-5.ab1
AtSN1 Col-6.ab1
AtSN1 Col-7.ab1
AtSN1 Col-8.ab1
AtSN1 Col-9.ab1
AtSN1 Col-10.ab1
AtSN1 Col-11.ab1
AtSN1 Col-12.ab1
AtSN1 Col-13.ab1
AtSN1 Col-14.ab1
AtSN1 Col-15.ab1
AtSN1 Col-16.ab1
AtSN1 Col-17.ab1
AtSN1 Col-18.ab1
AtSN1 Col-19.ab1
AtSN1 Col-20.ab1
100
120
140
156

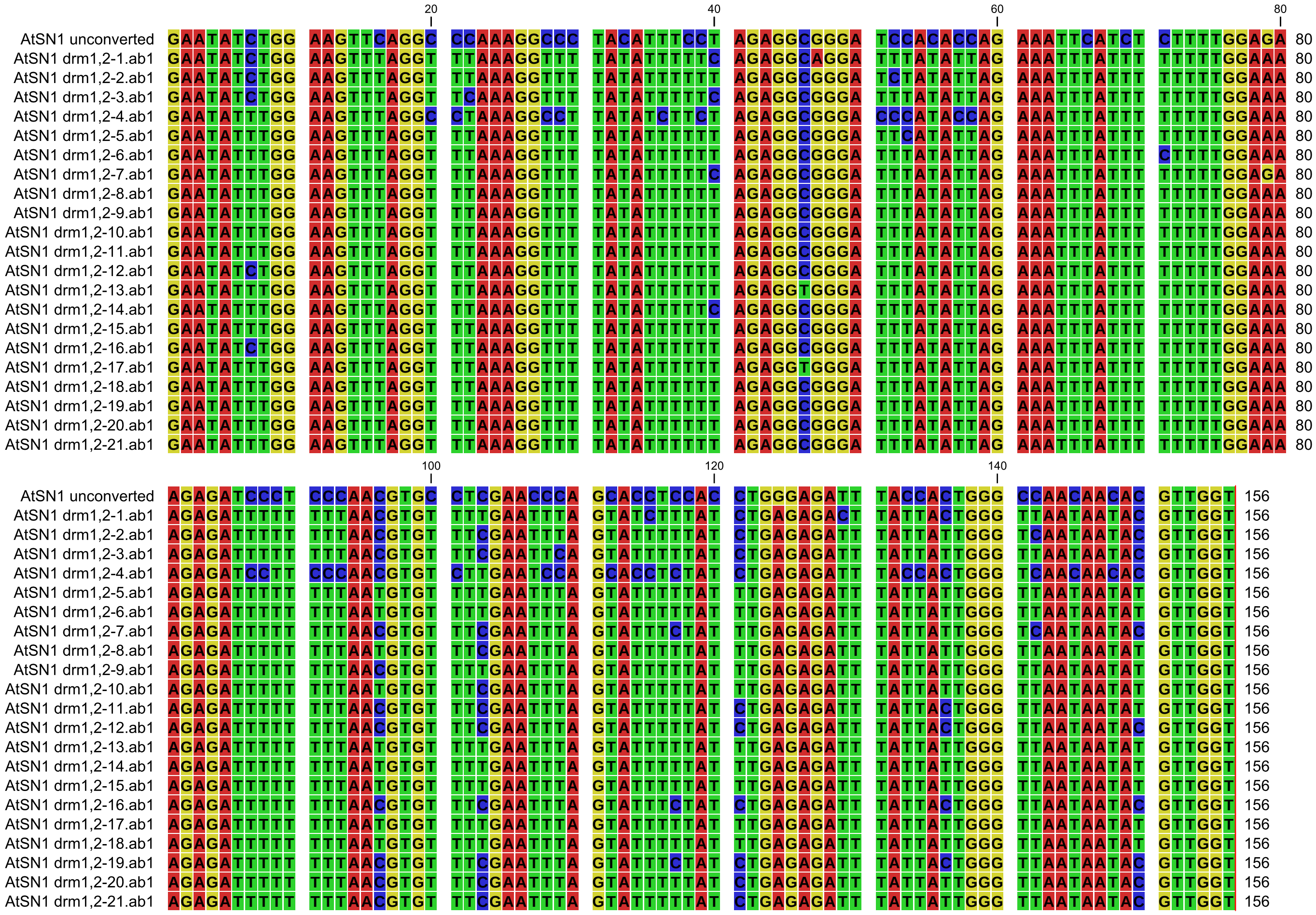

```
                                               20                    40                    60                    80
AtSN1 uncoverted  GAATATCTGG AAGTTCAGGC CCAAAGGCCC TACATTTCCT AGAGGCGGGA TCCACACCAG AAATTCATCT CTTTTGGAGA 80
AtSN1 cmt3-1.ab1  GAATATCTGG AAGTTTAGGT TTAAAGGTTT TATATCTTTT AGAGGTGGGA TTTATATTAG AAATTTATTT TTTTTGGAAA 80
AtSN1 cmt3-2.ab1  GAATATCTGG AAGTTCAGGT CCAAAGGCCT TACATCTCCC AGAGGTGGGA CTCATACCAG AAATTCAT-T TTTTTGGAAA 79
AtSN1 cmt3-3.ab1  GAATATCTGG AAGTTCAGGC CCAAAGGCCT TACATCTCTC AGAGGCGGGA CCCATACCAG AAATTCATCT TCTTTGGAAA 80
AtSN1 cmt3-4.ab1  GAATATCTGG AAGTTCAGGC CCAAAGGCCT TACATTTTTT AGAGGCGGGA TTTATATTAG AGATTCATCT TCTTTGGAAA 80
AtSN1 cmt3-5.ab1  GAATATTTGG AA-CCCGGGT TCAAAGGTTT TATTTTTTTT AGAGCCGGGA TTTGTTTTAG AAATCTA-CT TTTTTGGAAA 78
AtSN1 cmt3-6.ab1  GAATATTTGG AAGTCTAGGT CTAAAGCTTT TCTATTTCTT AGAGGTGGGA TTTCTATTAG AAATTTATTT TTTTTGGACA 80
AtSN1 cmt3-7.ab1  GAATATTTGG AAGTTTAGGT CTAAAGGTTT TATATTTTTT AGAGGTGGGA TTTATATTAG AAATTTATTT TTTTTGGAAA 80
AtSN1 cmt3-8.ab1  GAATATTTGG AAGTTTAGGT TTAAAGGTCT TATATTTTTT AGAGGCGGGA TTTATATTAG AAATTTATCT TTTTTGGAAA 80
AtSN1 cmt3-9.ab1  GAATATTTGG AAGTTTAGGT TTAAAGGTTT TATATTTTTT AGAGGCGGGA TTTATATTAG AAATTTATTT TTTTTGGAAA 80
AtSN1 cmt3-10.ab1 GAATATTTGG AAGTTTAGGT TTAAAGGTTT TATATTTTTT AGAGGTGGGA TTTATATTAG AAATTTATTT TTTTTGGAAA 80
AtSN1 cmt3-11.ab1 GAGGATGGGA AAGTTTCACC CCAAGATTTT TAT-TTTTTT AAAGGCGGGG TTTTTATTTG AAATTTTTTT TTTTAAGAAA 79
AtSN1 cmt3-12.ab1 GAATATTTGG AAGTTTAGGT TTAAAGGTTT TATATTTTTT AGAGGCGGGA TTTGTATTAG AAATCTATTT TTTT-GGAAA 79
AtSN1 cmt3-13.ab1 GAATATTTGG AAGTTTAGGT TTAAAGGTTT TATATTTTTT AGAGGCGGGA TTTATATTAG AAATTTATTT TTTTTGGAAA 80

                                              100                   120                   140
AtSN1 uncoverted  AGAGATCCCT CCCAACGTGC CTCGAACCCA GCACCTCCAC CTGGGAGATT TACCACTGGG CCAACAACAC GTTGGT 156
AtSN1 cmt3-1.ab1  AGAGATTTTT TTTAATGTGT TTTGAATTTA GTATTTTTAT CTGAGAGATT TATTATTGGG CCAACAATAC GTTGGT 156
AtSN1 cmt3-2.ab1  AGAGATCCTT CCCAATGTGT CTCGAATCCA GCACCTCTAT CTGAGAGATT TACCACTGGG CCAACAACAT GTTGGT 155
AtSN1 cmt3-3.ab1  AGAGATCCTT CCCAACGTGT CTCGAATCCA GCACCTCTAT CTGAGAGATT TACCACTGGG TCAACAACAC GTTGGT 156
AtSN1 cmt3-4.ab1  AGAGATCCTT CCCAATGTGT CTCGAATTCA GCATCTCTAT TTGAGAGATT TACCATTGGG CCAACAATAC GTTGGT 156
AtSN1 cmt3-5.ab1  AGAGATTTTT TTTAATGTGC TTTGAATCTA GTATTTTTAT TTGAGAGATT TACTATTGCG TTAATAACAT ACTGGT 154
AtSN1 cmt3-6.ab1  AGAGATTTTT TTTAATGTGT TTTGAATCCA GTATTTTTAT TTGAGAGATT TATTATTGGG TTAACAATAT GCTGGT 156
AtSN1 cmt3-7.ab1  AGAGATTTTT TTTAATGTGT TTTGAATTTA GTATTTTTAT TTGAGAGATT TATTATTGGG TTAATAATAT GTTGGT 156
AtSN1 cmt3-8.ab1  AGAGATCCTT CCCAATGTGT CTCGAATCCA GCACCTTTAT TTGAGAGATT TATTATTGGG TCAATAATAC GTTGGT 156
AtSN1 cmt3-9.ab1  AGAGACTTTT TTTAATGTGT TTCGAATTTA GTATTTTTAT TTGAGAGATT TATTATTGGG TTAATAATAC GTTGGT 156
AtSN1 cmt3-10.ab1 AGAGATTTTT TTTAACGTGT TTCGAATTTA GTATCTTTAT CTGAGAGATT TATTATTGGG TTAATAATAC GTTGGT 156
AtSN1 cmt3-11.ab1 GAAGTTTTTT TTAAATGGTT TTAAGATTTC GTATTTTT-T TTGAGAGAAT TTTGTCTGGA TTAAAACTAG GTAGAG 154
AtSN1 cmt3-12.ab1 AGAGATTTTT TTTAATGTGT TTTGAATTTA GTATTTTTAT TTGAGAGATT TATTATTGGG TTAATAATAT GTTGGT 155
AtSN1 cmt3-13.ab1 AGAGATTTTT TTTAACGTGT TTCGAATTTA GTATTTTTAT CTGAGAGATT TATTACTGGG TTAATAATAT GTTGGT 156
```

```
                                                20                   40                   60                   80
AtSN1 unconverted  GAATATCTGG AAGTTCAGGC CCAAAGGCCC TACATTTCCT AGAGGCGGGA TCCACACCAG AAATTCATCT CTTTTGGAGA 80
AtSN1 2-1.ab1      GAATATCTGG AAGTTCAGGT TTAAAGGTTT TATATTTTTC AGAGGCGGGA TTTATATCAG AAATTTATTT TTTTTGGAAA 80
AtSN1 2-2.ab1      GA-TATCTGG AAGTTCAGGT CCAAAGGTCT TATATTTTTT AGAGGCGGGA TTTATATTAG AAATTTATTT TTTTTGGAAA 79
AtSN1 2-3.ab1      GAATATCTGG AAGTTCAGGT TTAAAGGTTT TATATTTTCC AGAGGCGGGA TTTATATTAG AAATTTATCT TTTTTGGAAA 80
AtSN1 2-4.ab1      GAATATCTGG AAGTTCAGGT TTAAAGGTTT TATATTTTTT AGAGGCGGGA TTTATATTAG AAATTTATTT TTTTTGGAAA 80
AtSN1 2-5.ab1      GAATATCTGG AAGTTCAGGT TTAAAGGTCT TACATCTCCC AGAGGCGGGA TTTATATTAG AAATTTATTT TTTTTGGAAA 80
AtSN1 2-6.ab1      GAATATCTGG AAGTTCAGGT TCAAAGGCCT TATATTTTTT AGAGGCGGGA TTTATATTAG AAATTTATTT TTTTTGGAAA 80
AtSN1 2-7.ab1      GAATATTTGG AAGTTCAGGT CCAAAGGCCT TACATCTCCC AGAGGCGGGA CCCATATTAG AAATTCATCT TCTTTGGAAA 80
AtSN1 2-8.ab1      GAATATTTGG AAGTTTAGGT TCAAAGGTCT TACATCTCCC AGAGGCGGGA TCCATATTAG AAATTTATTT TTTTTGGAAA 80
AtSN1 2-9.ab1      GAATATTTGG AAGTTTAGGT TTAAAGGCCT TACATCTCCC AGAGGCGGGA CCCATACCAG AAATTTATTT TTTTTGGAAA 80
AtSN1 2-10.ab1     GAATATTTGG AAGTTTAGGT TTAAAGGTCT TATATCTCCC AGAGGCGGGA TTTATATTAG AAATTTATTT TCTTTGGAAA 80
AtSN1 2-11.ab1     GAATATTTGG AAGTTTAGGT TTAAAGGTTT TATATTTTTT AGAGGTGGGA TTTATATTAG AAATTTATTT TTTTTGGAAA 80
AtSN1 2-12.ab1     GAATATCTGG AAGTTTAGGT TTAAAGGTTT TATATTTTTT AGAGGCGGGA TTTATATTAG AAATTTATTT TTTTTGGAAA 80
AtSN1 2-13.ab1     GATTTTTTGG A-GTTTTGGC CTAAAGTTTT TTTTTTTTTA AAGGGCGGGA TTTA-ATTAG AAATTTTTTT TTTTTGGAAA 78
AtSN1 2-14.ab1     GAATATCTGG AAGTTTAGGT TTAAAGGTTT TATATTTTTT AGAGGCGGGA TTTATATTAG AAATTTATTT TTTTTGGAAA 80
AtSN1 2-15.ab1     GAATATTTGG AAGTTTAGGT TTAAAGGTTT TATATTTTTT AGAGGCGGGA TTTATATTAG AAATTTATTT TTTTTGGAAA 80
AtSN1 2-16.ab1     GAATATTTGG AAGTTTAGGT TTAAAGGTTT TATATTTTTC AGAGGCGGGA TTTATATTAG AAATTTATTT TTTTTGGAAA 80
AtSN1 2-17.ab1     GAATATTTGG AAGTTTAGGT TTAAAGGTTT TATATTTTTT AGAGGCGGGA TTTATATTAG AAATTTATTT TTTT-GGAAA 79
AtSN1 2-18.ab1     GAATATTTGG AAGTTTAGGC CTAAAGGTTT TATATTTTTT AGAGGCGGGA TTTATATTAG AAATTTATTT TTTTTGGAAA 80

                                               100                  120                  140
AtSN1 unconverted  AGAGATCCCT CCCAACGTGC CTCGAACCCA GCACCTCCAC CTGGGAGATT TACCACTGGG CCAACAACAC GTTGGT 156
AtSN1 2-1.ab1      AGAGATTTTT TTTAACGTGT TTTGAATTTA GTATTTCTAT CTGAGAGATT TATTATTGGG TTAATAATAC GTTGGT 156
AtSN1 2-2.ab1      AGAGATCCTT TTTAACGTGT TTCGAATTTA GTATTTTTAT TTGAGAGATT TATTATTGGG TTAATAATAT GTTGGT 155
AtSN1 2-3.ab1      AGAGATTTTT TT-AATGTGT CTCGAATCCA GTATCTCTAT CTGAGAGATT TATTACTGGG TTAATAATAT GTTGGT 155
AtSN1 2-4.ab1      AGAGATTTTT TTTAACGTGT TTCGAATTTA GTATTTTTAT TTGAGAGATT TATTATTGGG TTAATAATAT GTTGGT 156
AtSN1 2-5.ab1      AGAGATTCTT TTCAACGTGT CTCGAATCCA GCACCTCTAT CTGAGAGATT TATTATTGGG TTAATAATAC GTTGGT 156
AtSN1 2-6.ab1      AGAGATTTTT TTTAACGTGT TTCGAATTTA GTACTTTTAT CTGAGAGATT TATTACTGGG TTAATAATAC GTTGGT 156
AtSN1 2-7.ab1      AGAGATTCTT CCCAACGTGT CTCGAATCCA GCACCTCTAT CTGAGAGATT TATCACTGGG CCAACAACAC GTTGGT 156
AtSN1 2-8.ab1      AGAGATTTTT TTTAATGTGT TTTGAATTTA GTATTTTTAT TTGAGAGATT TATCATTGGG TTAATAATAT GTTGGT 156
AtSN1 2-9.ab1      AGAGATTTTT TTCAATGTGT TTCGAATTTA GTATTTTTAT TTGAGAGATT TATCATTGGG TTAATAATAT GTTGGT 156
AtSN1 2-10.ab1     AGAGATCCTT CCCAACGTGT CTCGAATTCA GCATTTTTAT TTGAGAGATT TATTACTGGG TTAATAATAC GTTGGT 156
AtSN1 2-11.ab1     AGAGATTTTT TTTAATGTGT TTTGAATTTA GTATTTTTAT TTGAGAGATT TATTATTGGG TTAATAATAT GTTGGT 156
AtSN1 2-12.ab1     AGAGATTTTT TTTAACGTGT TTCGAATTTA GTATTTCTAT TTGAGAGATT TATTATTGGG TTAATAATAT GTTGGT 156
AtSN1 2-13.ab1     AGAGATTTTT TTTATTGT-T TTTGAAACTA GTATTTTTAT T-GAGACATT AATTACCCAG TCAAGAA-AG GTTGGT 151
AtSN1 2-14.ab1     AGAGATTTTT TTTAATGTGT TTTGAATTTA GTATTTCTAT CTGAGAGATT TATTATTGGG TTAATAATAC GTTGGT 156
AtSN1 2-15.ab1     AGAGATTTTT TTTAACGTGT TTCGAATTCA GTATTTTTAT TTGAGAGATT TATTATTGGG TTAATAATAT GTTGGT 156
AtSN1 2-16.ab1     AGAGATTTTT TTTAACGTGT TTCGAATTTA GTATTTTTAT CTGAGAGATT TATTATTGGG TTAATAATAC GTTGGT 156
AtSN1 2-17.ab1     AGAGATTTTT TTTAATGTGT TTTGAATTTA GTATTTTTAT TTGAGAGATT TATTACTGGG TTAATAATAC GTTGGT 155
AtSN1 2-18.ab1     AGAGATTTTT TTTAATGTGT TTTGAATTTA GTATTTTTAT TTGAGAGATT TATTATTGGG TTAATAATAT GTTGGT 156
```

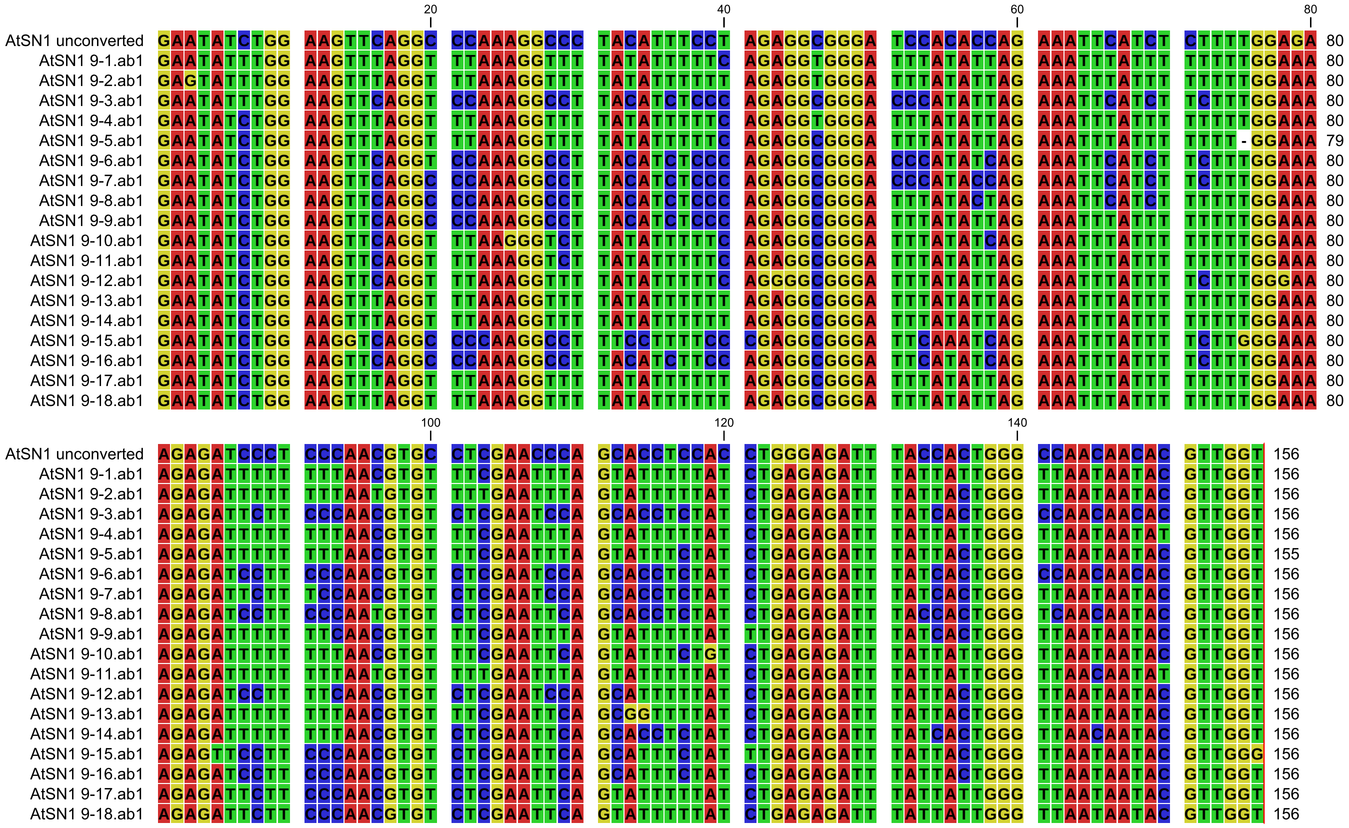
20 40 60 80
AtSN1 unconverted GAATATCTGG AAGTTCAGGC CCAAAGGCCC TACATTTCCT AGAGGCGGGA TCCACACCAG AAATTCATCT CTTTTGGAGA 80
AtSN1 9-1.ab1 GAATATTTGG AAGTTTAGGT TTAAAGGTTT TATATTTTTC AGAGGTGGGA TTTATATTAG AAATTTATTT TTTTTGGAAA 80
AtSN1 9-2.ab1 GAGTATTTGG AAGTTTAGGT TTAAAGGTTT TATATTTTTT AGAGGTGGGA TTTATATTAG AAATTTATTT TTTTTGGAAA 80
AtSN1 9-3.ab1 GAATATTTGG AAGTTCAGGT CCAAAGGCCT TACATCTCCC AGAGGCGGGA CCCATATTAG AAATTCATCT TCTTTGGAAA 80
AtSN1 9-4.ab1 GAATATCTGG AAGTTTAGGT TTAAAGGTTT TATATTTTTC AGAGGTGGGA TTTATATTAG AAATTTATTT TTTTTGGAAA 80
AtSN1 9-5.ab1 GAATATCTGG AAGTTTAGGT TTAAAGGTTT TATATTTTTC AGAGGCGGGA TTTATATTAG AAATTTATTT TTTT-GGAAA 79
AtSN1 9-6.ab1 GAATATCTGG AAGTTCAGGT CCAAAGGCCT TACATCTCCC AGAGGCGGGA CCCATATCAG AAATTCATCT TCTTTGGAAA 80
AtSN1 9-7.ab1 GAATATCTGG AAGTTCAGGC CCAAAGGCCT TACATCTCCC AGAGGCGGGA CCCATACCAG AAATTCATCT TCTTTGGAAA 80
AtSN1 9-8.ab1 GAATATCTGG AAGTTCAGGC CCAAAGGCCT TACATCTCCC AGAGGCGGGA TTTATACTAG AAATTCATCT TTTTTGGAAA 80
AtSN1 9-9.ab1 GAATATCTGG AAGTTCAGGC CCAAAGGCCT TACATCTCCC AGAGGCGGGA TTTATATTAG AAATTTATTT TTTTTGGAAA 80
AtSN1 9-10.ab1 GAATATCTGG AAGTTCAGGT TTAAGGGTCT TATATTTTTC AGAGGCGGGA TTTATATCAG AAATTTATTT TTTTTGGAAA 80
AtSN1 9-11.ab1 GAATATCTGG AAGTTCAGGT TTAAAGGTCT TATATTTTTC AGAGGCGGGA TTTATATTAG AAATTTATTT TTTTTGGAAA 80
AtSN1 9-12.ab1 GAATATCTGG AAGTTCAGGT TTAAAGGTTT TATATTTTTC AGGGGCGGGA TTTATATTAG AAATTTATTT TCTTTGGAAA 80
AtSN1 9-13.ab1 GAATATCTGG AAGTTTAGGT TTAAAGGTTT TATATTTTTT AGAGGCGGGA TTTATATTAG AAATTTATTT TTTTTGGAAA 80
AtSN1 9-14.ab1 GAATATCTGG AAGTTTAGGT TTAAAGGTTT TATATTTTTT AGAGGCGGGA TTTATATTAG AAATTTATTT TTTTTGGAAA 80
AtSN1 9-15.ab1 GAATATCTGG AAGGTCAGGC CCCAAGGCCT TTCCTTTTCC CGAGGCGGGA TTCAAATCAG AAATTTATTT TCTTGGGAAA 80
AtSN1 9-16.ab1 GAATATCTGG AAGTTCAGGC CCAAAGGCCT TACATCTTCC AGAGGCGGGA TTCATATCAG AAATTTATTT TCTTTGGAAA 80
AtSN1 9-17.ab1 GAATATCTGG AAGTTTAGGT TTAAAGGTTT TATATTTTTT AGAGGCGGGA TTTATATTAG AAATTTATTT TTTTTGGAAA 80
AtSN1 9-18.ab1 GAATATCTGG AAGTTTAGGT TTAAAGGTTT TATATTTTTT AGAGGCGGGA TTTATATTAG AAATTTATTT TTTTTGGAAA 80
100 120 140
AtSN1 unconverted AGAGATCCCT CCCAACGTGC CTCGAACCCA GCACCTCCAC CTGGGAGATT TACCACTGGG CCAACAACAC GTTGGT 156
AtSN1 9-1.ab1 AGAGATTTTT TTTAACGTGT TTCGAATTTA GTATTTTTAT CTGAGAGATT TATTATTGGG TTAATAATAC GTTGGT 156
AtSN1 9-2.ab1 AGAGATTTTT TTTAATGTGT TTTGAATTTA GTATTTTTAT CTGAGAGATT TATTACTGGG TTAATAATAC GTTGGT 156
AtSN1 9-3.ab1 AGAGATTCTT CCCAACGTGT CTCGAATCCA GCACCTCTAT CTGAGAGATT TATCACTGGG CCAACAACAC GTTGGT 156
AtSN1 9-4.ab1 AGAGATTTTT TTTAACGTGT TTCGAATTTA GTATTTTTAT CTGAGAGATT TATTATTGGG TTAATAATAT GTTGGT 156
AtSN1 9-5.ab1 AGAGATTTTT TTTAACGTGT TTCGAATTTA GTATTTCTAT CTGAGAGATT TATTACTGGG TTAATAATAC GTTGGT 155
AtSN1 9-6.ab1 AGAGATCCTT CCCAACGTGT CTCGAATCCA GCACCTCTAT CTGAGAGATT TATCACTGGG CCAACAACAC GTTGGT 156
AtSN1 9-7.ab1 AGAGATTCTT TCCAACGTGT CTCGAATCCA GCACCTCTAT CTGAGAGATT TATCACTGGG TTAATAATAC GTTGGT 156
AtSN1 9-8.ab1 AGAGATCCTT CCCAATGTGT CTCGAATTCA GCACCTCTAT CTGAGAGATT TACCACTGGG TCAACAATAC GTTGGT 156
AtSN1 9-9.ab1 AGAGATTTTT TTTAACGTGT TTCGAATTTA GTATTTTTAT TTGAGAGATT TATCACTGGG TTAATAATAC GTTGGT 156
AtSN1 9-10.ab1 AGAGATTTTT TTTAACGTGT TTCGAATTCA GTATTTCTGT CTGAGAGATT TATTATTGGG TTAATAATAC GTTGGT 156
AtSN1 9-11.ab1 AGAGATTTTT TTTAATGTGT TTTGAATTTA GTATTTTTAT CTGAGAGATT TATTATTGGG TTAACAATAT GTTGGT 156
AtSN1 9-12.ab1 AGAGATCCTT TTCAACGTGT CTCGAATCCA GCATTTTTAT CTGAGAGATT TATTACTGGG TTAATAATAC GTTGGT 156
AtSN1 9-13.ab1 AGAGATTTTT TTTAACGTGT TTCGAATTCA GCGGTTTTAT CTGAGAGATT TATTACTGGG TTAATAATAC GTTGGT 156
AtSN1 9-14.ab1 AGAGATTTTT TTTAACGTGT CTCGAATTCA GCACCTCTAT CTGAGAGATT TATCACTGGG TTAACAATAC GTTGGT 156
AtSN1 9-15.ab1 AGAGTTCCTT CCCAACGTGT CTCGAATTCA GCATTTCTAT TTGAGAGATT TATTACTGGG TTAATAATAC GTTGGG 156
AtSN1 9-16.ab1 AGAGATCCTT CCCAACGTGT CTCGAATTCA GCATTTCTAT CTGAGAGATT TATTACTGGG TTAATAATAC GTTGGT 156
AtSN1 9-17.ab1 AGAGATTCTT CCCAACGTGT CTCGAATTCA GTATTTTTAT CTGAGAGATT TATTATTGGG TTAATAATAC GTTGGT 156
AtSN1 9-18.ab1 AGAGATTCTT CCCAACGTGT CTCGAATTCA GTATTTTTAT CTGAGAGATT TATTATTGGG TTAATAATAC GTTGGT 156

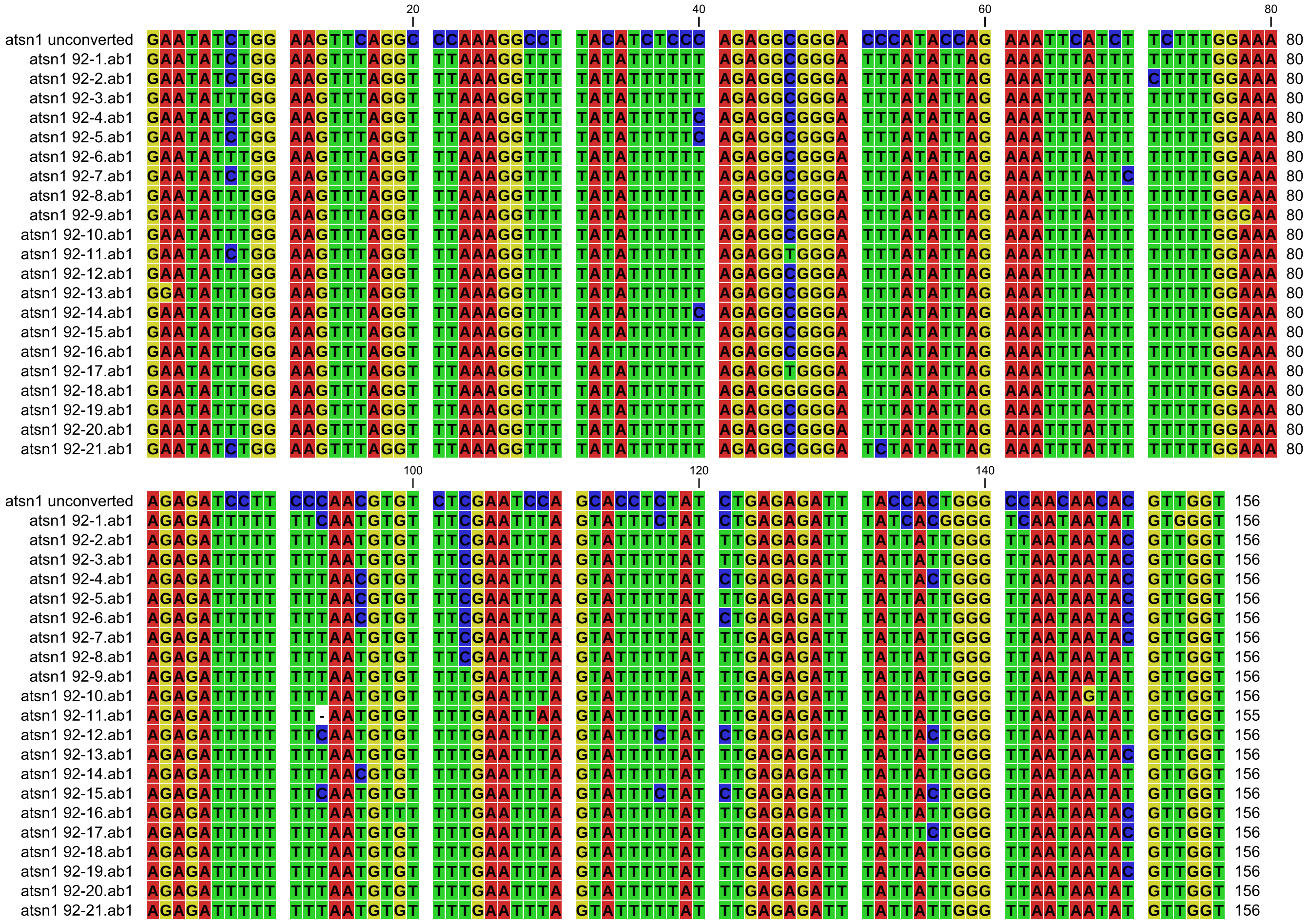
atsn1 unconverted
atsn1 92-1.ab1
atsn1 92-2.ab1
atsn1 92-3.ab1
atsn1 92-4.ab1
atsn1 92-5.ab1
atsn1 92-6.ab1
atsn1 92-7.ab1
atsn1 92-8.ab1
atsn1 92-9.ab1
atsn1 92-10.ab1
atsn1 92-11.ab1
atsn1 92-12.ab1
atsn1 92-13.ab1
atsn1 92-14.ab1
atsn1 92-15.ab1
atsn1 92-16.ab1
atsn1 92-17.ab1
atsn1 92-18.ab1
atsn1 92-19.ab1
atsn1 92-20.ab1
atsn1 92-21.ab1

```
                                             20                                          40                                          60                                          80
                                              |                                           |                                           |                                           |
           Atsn1  GAATATCTGG  AAGTTCAGGC  CCAAAGGCCT  TACATCTCCC  AGAGGCGGGA  CCCATACCAG  AAATTCATCT  TCTTTGGAAA  80
 Atsn1 492-1.ab1  GAATATTTGG  AAGTTCAGGT  TTAAAGGTTT  TATATTTTCT  AGAGGCGGGA  TTTATATTAG  AAATTTATTT  TTTTTGGAAA  80
 Atsn1 492-2.ab1  GAATATTTGG  AAGTTTAGGT  TTAAAGGTTT  TATATTTTTT  AGAGGCGGGA  TTTATATTAG  AAATTTATTT  TTTTTGGAAA  80
 Atsn1 492-3.ab1  GAATATTTGG  AAGTTTAGGT  TTAAAGGTTT  TATATTTTTT  AGAGGTGGGA  TTTATATTAG  AAATTTATTT  TTTTTGGAAA  80
 Atsn1 492-4.ab1  GAATATTTGG  AAGTTTAGGT  TTAAAGGTTT  TATATTTTTT  AGAGGTGGGA  TTTATATCAG  AAATTTATTT  TTTTTGGAAA  80
 Atsn1 492-5.ab1  GAATATTTGG  AAGTTTAGGT  TTAAAGGTTT  TATATTTTTT  AGAGGCGGGA  TTTATATTAG  AAATTTATTT  TTTTTGGAAA  80
 Atsn1 492-6.ab1  GAATATTTGG  AAGTTTAGGT  TTAAAGGTTT  TATATTTTTT  AGAGGCGGGA  TTTATATTAG  AAATTTATTT  TCTTTGGAAA  80
 Atsn1 492-7.ab1  GAATATTTGG  AAGTTTAGGT  TTAAAGGTTT  TATATTTTTT  AGAGGTGGGA  TTTATATTAG  AAATTTATTT  TTTTTGGAAA  80
 Atsn1 492-8.ab1  GAATATTTGG  AAGTTTAGGT  TTAAAGGTTT  TTTTTTTTTT  TTAGGCGGGA  TTTATATTAG  AAATTTATTT  TTTTTGGAAA  80
 Atsn1 492-9.ab1  GAATATTTGG  AAGTTTAGGT  TTAAAGGTTT  TATATTTTTT  AGAGGCGGGA  TTTATATTAG  AAATTTATTT  TCTTTGGAAA  80
Atsn1 492-10.ab1  GAATATTTGG  AAGTTTAGGT  TTAAAGGTTT  TATATTTTTT  AGAGGCGGGA  TCTATATTAG  AAATTTATTT  TTTTTGGAAA  80
Atsn1 492-11.ab1  GAATATTTGG  GAGTTTAGGT  TTAAAGGTTT  TATATTTTTT  AGAGGTGGGA  TTTATATTAG  AAATTTATTT  TTTTTGGAAA  80
Atsn1 492-12.ab1  GAATATTTGG  AAGTTTAGGT  TTAAAGGTTT  TATATTTTTT  AGAGGCGGGA  TTTATATTAG  AAATTTATTT  TTTTTGGAAA  80
Atsn1 492-13.ab1  GAATATTTGG  AAGTTTAGGT  TTAAAGGTTT  TATATTTTTT  AGAGGCGGGA  TTTATATTAG  AAATTTATTT  TTTTTGGAAA  80
Atsn1 492-14.ab1  GAATATTTGG  AAGTTTAGGT  TTAAAGGTTT  TATATTTTTT  AGAGGCGGGA  TTTATATTAG  AAATTTATTT  TTTTTGGAAA  80
Atsn1 492-15.ab1  GAATATTTGG  AAGTTTAGGT  TTAAAGGTTT  TATATTTTTT  AGAGGCGGGA  TTTATATTAG  AAATTTATTT  TTTTTGGAAA  80
Atsn1 492-16.ab1  GAATATTTGG  AAGTTTAGGT  TTAAAGGTTT  TATATTTTTT  AGAGGTGGGA  TTTATATTAG  AAATTTATTT  TTTTTGGAAA  80
Atsn1 492-17.ab1  GAATATTTGG  AAGTTTAGGT  TTAAAGGTTT  TATATTTTTT  AGAGGTGGGA  TTTATATTAG  AAATTTATTT  TTTTTGGAAA  80
Atsn1 492-18.ab1  GAATATTTGG  AAGTTTAGGT  TTAAAGGTTT  TATATTTTTT  AGAGGCGGGA  TTTATATTAG  AAATTTATTT  TTTTTGGAAA  80
Atsn1 492-19.ab1  GAATATTTGG  AAGTTTAGGT  TTAAAGGTTT  TATATTTTTT  AGAGGCGGGA  TTTATATTAG  AAATTTATTT  TTTTTGGAAA  80
Atsn1 492-20.ab1  GAATATTTGG  AAGTTTAGGT  TTAAAGGTTT  TATATTTTTT  AGAGGCGGGA  TTTATATTAG  AAATTTATTT  TTTTTGGAAA  80

                                            100                                         120                                         140
                                              |                                           |                                           |
           Atsn1  AGAGATCCTT  CCCAACGTGT  CTCGAATCCA  GCACCTCTAT  CTGAGAGATT  TACCACTGGG  CCAACAACAC  GTTGGTC  157
 Atsn1 492-1.ab1  AGAGATTTTT  TTTAATGTGT  TTCGAATTTA  GTATTTTTAT  TTGAGAGATT  TATTATTGGG  TTAATAATAT  GTTGGTG  157
 Atsn1 492-2.ab1  AGGGATTTTT  TTTAATGTGT  TTTGAATTTA  GTATTTTTAT  TTGAGAGATT  TATTATTGGG  TTAATAATAT  GTTGGTG  157
 Atsn1 492-3.ab1  AGAGATTTTT  TTTAATGTGT  TTTGAATTTA  GTATTTTTAT  TTGAGAGATT  TATTATTGGG  TTAATAATAT  GTTGGTG  157
 Atsn1 492-4.ab1  AGAGATTTTT  TTTAATGTGT  TTTGAATTTA  GTATTTTTAT  TTGAGAGATT  TATTATTGGG  TTAATAATAT  GTTGGTG  157
 Atsn1 492-5.ab1  AGAGATTTTT  TTTAATGTGT  TTTGAATTTA  GTATTTTTAT  TTGAGAGATT  TATTATTGGG  TTAATAATAT  GTTGGTG  157
 Atsn1 492-6.ab1  AGAGATTTTT  TTTAATGTGT  TTTGAATTTA  GTATTTTTAT  TTGAGAGATT  TATTATTGGG  TTAATAATAT  GTTGGTG  157
 Atsn1 492-7.ab1  AGAGATTTTT  TTTAATGTGT  TTTGAATTTA  GTATTTTTAT  TTGAGAGATT  TATTATTGGG  TTAATAATAT  GTTGGTG  157
 Atsn1 492-8.ab1  AGAGATTTAT  TTAAATGTGT  TTTGAATAAA  GTATTTTTAT  TTGAGAGATT  TATTATTGGG  TTAATAATAT  GTTGGGG  157
 Atsn1 492-9.ab1  AGAGATTTTT  TTTAATGTGT  TTTGAATTTA  GTATTTTTAT  TTGAGAGATT  TATTATTGGG  TTAATAATAT  GTTGGTG  157
Atsn1 492-10.ab1  AGAGATTTTT  TTTAATGTGT  TTCGAATTTA  GTATTTTTAT  TTGAGAGATT  TATTATTGGG  TTAATAATAC  GTTGGTG  157
Atsn1 492-11.ab1  AGAGATTTTT  TTTAATGTGT  TTTGAATTTA  GTATTTTTAT  TTGAGAGATT  TATTATTGGG  TTAATAATAT  GTTGGTG  157
Atsn1 492-12.ab1  AGAGAATTTT  TTTAATGTGT  TTTGAATTTA  GTATTTTTAT  TTGAGAGATT  TATTATTGGG  TTAATAATAT  GTTGGTG  157
Atsn1 492-13.ab1  AGAGATTTTT  TTTAATGTGT  TTTGAATTTA  GTATTTTTAT  TTGAGAGATT  TATTATTGGG  TTAATAATAT  GTTGGTG  157
Atsn1 492-14.ab1  AGAGATTTTT  TTTAATGTGT  TTTGAATTTA  GTATCTTTAT  TTGAGAGATT  TATTATTGGG  TTAATAATAC  GTTGGTG  157
Atsn1 492-15.ab1  AGAGATTTTT  TTTAATGTGT  TTTGAATTTA  GTATTTTTAT  TTGAGAGATT  TATTATTGGG  TTAATAATAT  GTTGGTG  157
Atsn1 492-16.ab1  AGAGATTTTT  TTTAATGTGT  TTTGAATTTA  GTATTTTTAT  TTGAGAGATT  TATTATTGGG  TTAATAATAT  GTTGGTG  157
Atsn1 492-17.ab1  AGAGATTTTT  TTTAACGTGT  TTTGAATTTA  GTATTTTTAT  TTGAGAGATT  TATTATTGGG  TTAATAATAT  GTTGGTG  157
Atsn1 492-18.ab1  AGAGATTTTT  TTTAATGTGT  TTTGAATTTA  GTATTTTTAT  TTGAGAGATT  TATTATTGGG  TTAATAATAT  GTTGGTG  157
Atsn1 492-19.ab1  AGAGATTTTT  TTTAACGTGT  TTCGAATTTA  GTATTTTTAT  TTGAGAGATT  TATTATTGGG  TTAATAATAC  GTTGGTG  157
Atsn1 492-20.ab1  AGAGATTTTT  TTTAATGTGT  TTTGTATTTA  GTATTTTTAT  TTGAGAGATT  TATTATTGGG  TTAATAATAT  GTTGGTG  157
```

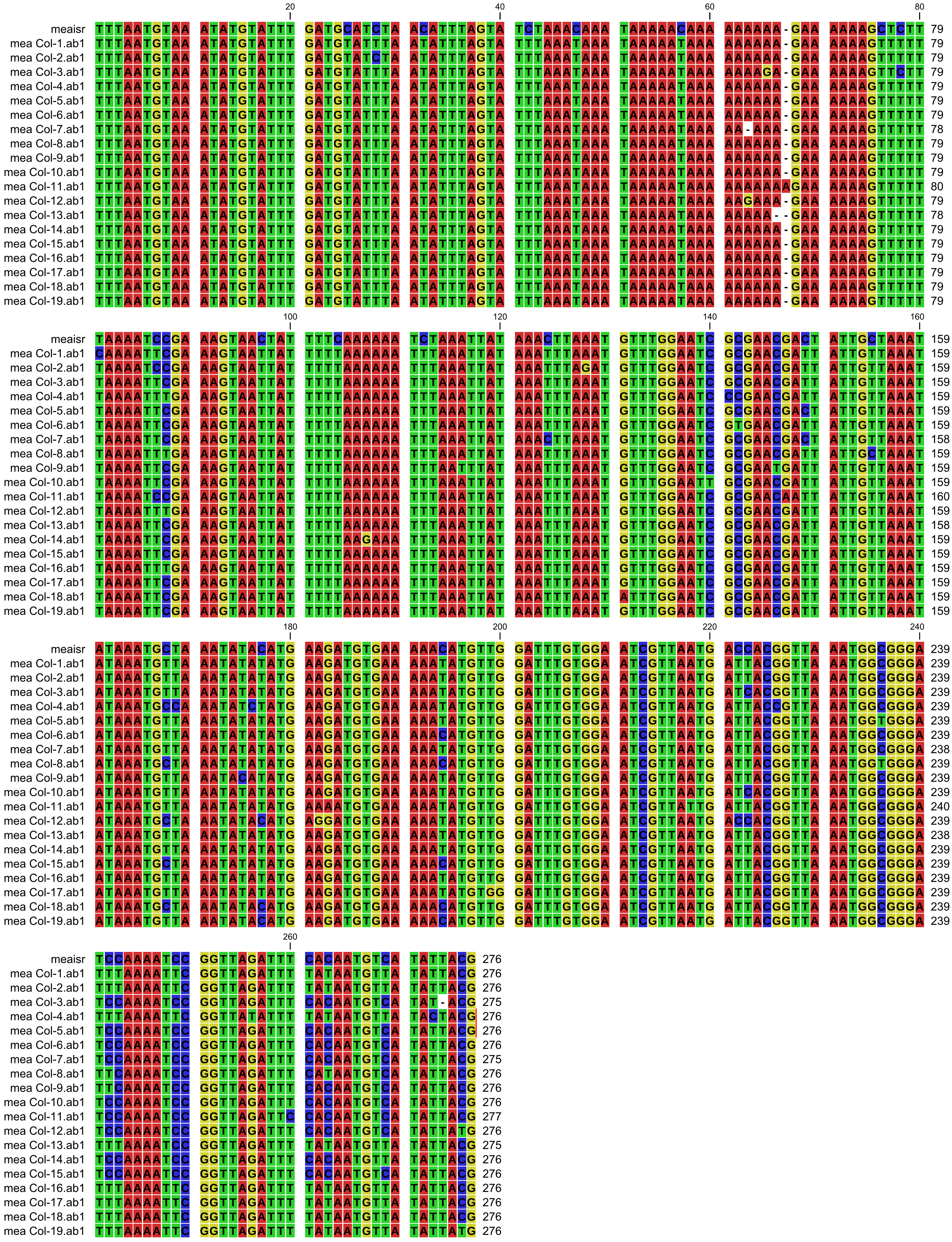
meaisr
mea Col-1.ab1
mea Col-2.ab1
mea Col-3.ab1
mea Col-4.ab1
mea Col-5.ab1
mea Col-6.ab1
mea Col-7.ab1
mea Col-8.ab1
mea Col-9.ab1
mea Col-10.ab1
mea Col-11.ab1
mea Col-12.ab1
mea Col-13.ab1
mea Col-14.ab1
mea Col-15.ab1
mea Col-16.ab1
mea Col-17.ab1
mea Col-18.ab1
mea Col-19.ab1

```
                          20         40         60         80
meaisr             TTTAATGTAA ATATGTATTT GATGCATCTA ACATTTAGTA TCTAAACAAA TAAAAACAAA AAAAAA-GAA AAAAGCTCTT 79
mea drm1,2-1.ab1   TTTAATGTAA ATATGTATTT GATGTATTTA ATATTTAGTA TTTAAATAAA TAGAAATAAA AAAAAA-GAA AAAAGCTTTT 79
mea drm1,2-2.ab1   TTTAATGTAA ATATGTATTT GATGTATTTA ATATTTAGTA TTTAAATAAA TAAAAATAAA AAAAAA-GAA AAAAGTTCTT 79
mea drm1,2-3.ab1   TTTAATGTAA ATATGTATTT GATGTATTTA ATATTTAGTA TTTAAATAAA TAAAAATAAA AAAAAA-GAA AAAAGTTTTT 79
mea drm1,2-4.ab1   TTTAATGTAA ATATGTATTT GATGTATTTA ATATTTAGTA TTTAAATAAA TAAAAATAAA AAAAAA-GAA AAAAGTTTTT 79
mea drm1,2-5.ab1   TTTAATGTAA ATATGTATTT AATGTATTTA ATATTTAGTA TTTAAATAAA TAAAAATAAA AAAAAA-GAA AAAAGTTTTT 79
mea drm1,2-6.ab1   TTTAATGTAA ATATGTATTT GATGTATTTA ATATTTAGTA TTTAAATAAA TAAAAATAAA AAAAAA-GAA AAAAGTTTTT 79
mea drm1,2-7.ab1   TTTAATGTAA ATATGTATTT GATGTATTTA ATATTTAGTA TTTAAATAAA TAAAAATAAG AAAAAA-GAA AAAAGTTTTT 79
mea drm1,2-8.ab1   TTTAATGTAA ATATGTATTT GATGTATTTA ATATTTAGTA TTTAAATAAA TAAAAATAAA AAAAAA-GAA AAAAGTTTTT 79
mea drm1,2-9.ab1   TTTAATGTAA ATATGTATTT GATGTATTTA ATATTTAGTA TTTAAATAAA TAAAAATAAA AAAAAA-GAA AAAAGTTTTT 79
mea drm1,2-10.ab1  TTTAATGTAA ATATGTATTT GATGTATTTA ATATTTAGTA TTTAAATAAA TAAAAATAAA AAAAA--GAA AAAAGTTTTT 78
mea drm1,2-11.ab1  TTTAATGTAA ATATGTATTT GATGTATTTA ATATTTAGTA TTTAAATAAA TAAAAATAAA AAAAAA-GAA AAGAGTTTTT 79
mea drm1,2-12.ab1  TTTAATGTAA ATATGTATTT GATGTATTTA ATATTTAGTA TTTAAATAAA TAAAAATAAA AAAAAAAGAA AAAAGTTTTT 80
mea drm1,2-13.ab1  TTTAATGTAA ATATGTATTT GATGTATTTA ATATTTAGTA TTTAAATAAA TAAAAATAAA AAAAAA-GAA AAAAGTTTTT 79
mea drm1,2-14.ab1  TTTAATGTAA ATATGTATTT GATGTATTTA ATATTTAGTA TTTAAATAAA TAAAAATAAA AAAAAA-GAA AAAAGTTTTT 79
mea drm1,2-15.ab1  TTTAATGTAA ATATGTATTT GATGTATTTA ATATTTAGTA TTTAAATAAA TAAAAATAAA AAAAAA-GAA AAAAGTTTTT 79
mea drm1,2-16.ab1  TTTAATGTAA ATATGTATTT GATGTATTTA ATATTTAGTA TTTAAATAAA TAAAAATAAA AAAAAA-GAA AAAAGTTTTT 79
mea drm1,2-17.ab1  TTTAATGTAA ATATGTATTT GATGTATTTA ATATTTAGTA TTTAAATAAA TAAAAATAAA AAAAAA-GAA AAAAGTTTTT 79
mea drm1,2-18.ab1  TTTAATGTAA ATATGTATTT GATGTATTTA ATATTTAGTA TTTAAATAAA TAAAAATAAA AAAAAA-GAA AAAAGTTTTT 79

                          100        120        140        160
meaisr             TAAAATCCGA AAGTAACTAT TTTCAAAAAA TCTAAATTAT AAACTTAAAT GTTTGGAATC GCGAACGACT ATTGCTAAAT 159
mea drm1,2-1.ab1   TAAAATTTGA AAGTAATTAT TTTTAAAAAA TTTAAATTAT AAATTTAAAT GTTTGGAATC GCGAACGATT ATTGTTAAAT 159
mea drm1,2-2.ab1   TAAAATTTGA AAGTAATTAT TTTTAAAAAA TTTAAATTAT AAATTTAAAT GTTTGGAATT GTGAATGATT ATTGTTAAAT 159
mea drm1,2-3.ab1   TAAAATTTGA AAGTAATTAT TTTTAAAAAA TTTAAATTAT AAATTTAAAC GTTTGGAATC GCGAACGATT ATTGTTAAAT 159
mea drm1,2-4.ab1   TAAAATTTGA AAGTAATTAT TTTTAAAAAA TTTAAATTAT AAGTTTAAAT GTTTGGAATC GCGAACGATT ATTGCTAAAT 159
mea drm1,2-5.ab1   TAAAATTTGA AAGTAATTAT TTTTAAAAAA TTTAAATTAT AAATTTAAAT GTTTGGAATC GCGAACGATT ATTGTTAAAT 159
mea drm1,2-6.ab1   TAAAATTTGA AAGTAATTAT TTTTAAAAAA TTTAAATTAT AAATTTAAAT GTTTGGAATC GCGAGCGATT ATTGTTAAAT 159
mea drm1,2-7.ab1   TAAAATTTGA AAGTAATTAT TTTTAAAAAA TTTAAATTAT GAATTTAAAT GTTTGGAATC GCGAACGATT ATTGTTAAAT 159
mea drm1,2-8.ab1   TAAAATTTGA AAGTAATTAT TTTTAAAAAA TTTAAATTAT AAATTTAAAT GTTTGGAATC GCGAACGATT ATTGTTAAAT 159
mea drm1,2-9.ab1   TAAAATTTGA AAGTAATTAT TTTTAAAAAA TTTAAATTAT ATATTTAAAT GTTTGGAATC GCGAACGATT ATTGTTAAAT 159
mea drm1,2-10.ab1  TAAAATTTGA AAGTAATTAT TTTTAAAAAA TTTAAATTAT AAATTTAAAT GTTTGGAATC GCGAACGATT ATTGTTAAAT 158
mea drm1,2-11.ab1  TAAAATTTGA AAGTAATTAT TTTTAAAAAA TTTAAATTAT AAATTTAAAT GTTTGGAATC GCGAACGATT ATTGTTAAAT 159
mea drm1,2-12.ab1  TAAAATTTGA AAGTAATTAT TTTTAAAAAA TTTAAATTAT AAATTTAAAT GTTTGGAATC GCGAACGATT ATTGTTAAAT 160
mea drm1,2-13.ab1  TAAAATTTGA AAGTAATTAT TTTTAAAAAA TTTAAATTAT AAATTTAAAT GTTTGGAATC GCAAACGATT ATTGTTAAAT 159
mea drm1,2-14.ab1  TAAAATTTGA AGGTAATTAT TTTTAAAAAA TTTAAATTAT AAATTTAAAT GTTTGGAATC GCGAACGATT ATTGTTAAAT 159
mea drm1,2-15.ab1  TAAAATTTGA AAGTAATTAT TTTTAAAAAA TTTAAATTAT AAATTTAAAT GTTTGGAATC GCGAACGATT ATTGTTAAAT 159
mea drm1,2-16.ab1  TAAAATTTGA AAGTAATTAT TTTTAAAAAA TTTAAATTAT AAATTTAAAT GTTTGGAATC GCGAACGATT ATTGTTAAAT 159
mea drm1,2-17.ab1  TAAAATTTGA AAGTAATTAT TTTTAAAAAA TTTAAATTAT AAATTTAAAT GTTTGGAATC GCGAACGATT ATTGTTAAAT 159
mea drm1,2-18.ab1  TAAAATTTGA AAGTAATTAT TTTTAAAAAA TTTAAATTAT AAATTTAAAT GTTTGGAATC GCGAACGATT ATTGTTAAAT 159

                          180        200        220        240
meaisr             ATAAATGCTA AATATACATG AAGATGTGAA AAACATGTTG GATTTGTGGA ATCGTTAATG ACCACGGTTA AATGGCGGGA 239
mea drm1,2-1.ab1   ATAAATGTTA AATATATATG AAGATGTGAA AAGTATGTTG GATTTGTGGA ATCGTTAATG ATTACGGTTA AATGGCGGGA 239
mea drm1,2-2.ab1   ATAAATGTTA AATATATATG AAGATGTGAA AAATATGTTG GATTTGTGGA ATCGTTAATG ATTATGGTTA AATGGTGGGA 239
mea drm1,2-3.ab1   ATAAATGTTA AATATATATG AAGATGTGAA AAATATGTTG GATTTGTGGA ATCGTTAATG ATTACGGTTA AATGGCGGGA 239
mea drm1,2-4.ab1   ATAAATGTTA AATATACATG AAGATGTGAA AAACATGTTG GATTTGTGGA ATCGTTAATG ACCACGGTTA AATGGCGGGA 239
mea drm1,2-5.ab1   ATAAATGTTA AATATATATG AAGATGTGAA AAATATGTTG GATTTGTGGA ATCGTTAATG ATTACGGTTA AATGGCGGGA 239
mea drm1,2-6.ab1   ATAAATGTTA AATATATATG AAGATGTGAA AAATATGTTG GATTTGTGGA ATCGTTAATG ATTACGGTTA AATGGTGGGA 239
mea drm1,2-7.ab1   ATAAATGTTA AATATATATG AAGATGTGAA AAATATGTTG GATTTGTGGA ATTGTTAATG ATTACGGTTA AATGGCGGGA 239
mea drm1,2-8.ab1   ATAAATGTTA AATATATATG AAGATGTGAA AAATATGTTG GATTTGTGGA ATCGTTAATG ATTACGGTTA AATGGCGGGA 239
mea drm1,2-9.ab1   ATAAATGTTA AATATATATG AAGATGTGAA AAATATGTTG GATTTGTGGA ATCGTTAATG ATTACGGTTA AATGGCGGGA 239
mea drm1,2-10.ab1  ATAAATGTTA AATATATATG AAGATGTGAA AAATATGTTG GATTTGTGGA ATCGTTAATG ATTACGGTTA AATGGCGGGA 238
mea drm1,2-11.ab1  ATAAATGTTA AATATATATG AAGATGTGAA AAATATGTTG GATTTGTGGA ATCGTTAATG ATTACGGTTA AATGGCGGGA 239
mea drm1,2-12.ab1  ATAAATGTTA AATATATATG AAGATGTGAA AAATATGTTG GATTTGTGGA ATCGTTAATG ATTACGGTTA AATGGCGGGA 240
mea drm1,2-13.ab1  ATAAATGTTA AATATATATG AAGATGTGAA AAATATGTTG GATTTGTGGA ATCGTTAATG ATTACGGTTA AATGGCGGGA 239
mea drm1,2-14.ab1  ATAAATGTTA AATATATATG AAGATGTGAA AAATATGTTG GATTTGTGGA ATCGTTAATG ATTACGGTTA AATGGCGGGA 239
mea drm1,2-15.ab1  ATAAATGTTA AATATGTATG AAGATGTGAA AAATATGTTG GATTTGTGGA ATCGTTAATG ATTACGGTTA AATGGCGGGA 239
mea drm1,2-16.ab1  ATAAATGTTA AATATATATG AAGATGTGAA AAATATGTTG GATTTGTGGA ATCGTTAATG ATTACGGTTA AATGGCGGGA 239
mea drm1,2-17.ab1  ATAAATGTTA AATATATATG AAGATGTGAA AAATATGTTG GATTTGTGGA ATCGTTAATG ATTACGGTTA AATGGCGGGA 239
mea drm1,2-18.ab1  ATAAATGTTA AATATATATG AAGATGTGAA AAATATGTTG GATTTGTGGA ATCGTTAATG ATTACGGTTA AATGGCGGGA 239

                          260
meaisr             TCCAAAATCC GGTTAGATTT CACAATGTCA TATTACG 276
mea drm1,2-1.ab1   TTTAAAATTC GGTTAGATTT TATAATGTTA TATTACG 276
mea drm1,2-2.ab1   TTTAAAATTC GGTTAGATTT TATAATGTTA TATTACG 276
mea drm1,2-3.ab1   TTTAAAATTC GGTTAGATTT TATAATGTTA TATTACG 276
mea drm1,2-4.ab1   TCCAAAATCC GGTTAGATTT CACAATGTCA TATTACG 276
mea drm1,2-5.ab1   TTTAAAATTC GGTTAGATTT TACAATGTCA TATTACG 276
mea drm1,2-6.ab1   TTTAAAATTC GGTTAGATTT TACAATGTCA TATTACG 276
mea drm1,2-7.ab1   TTTAAAATTC GGTTAGATTT TATAATGTTA TATTACG 276
mea drm1,2-8.ab1   TTCAAAATTC GGTTAGATTT TATAATGTTA TATTACG 276
mea drm1,2-9.ab1   TTTAAAATTC GGTTAGATTT TATAATGTTA TATCATG 276
mea drm1,2-10.ab1  TTTAAAATTC GGTTAGATTT TATAATGTTA TATTATG 275
mea drm1,2-11.ab1  TTTAAAATTC GGTTAGATTT TATAATGTTA TATTACG 276
mea drm1,2-12.ab1  TTTAAAATTC GGTTAGATTT TATAATGTTA TATTACG 277
mea drm1,2-13.ab1  TTTAAAATTC GGTTAGATTT TATAATGTTA TATTACG 276
mea drm1,2-14.ab1  TTTAAAATTC GGTTAGATTT TATAATGTTA TATTACG 276
mea drm1,2-15.ab1  TTTAAAATTC GGTTAGATTT TATAATGTTA TATTACG 276
mea drm1,2-16.ab1  TTTAAAATTC GGTTAGATTT TATAATGTTA TATTACG 276
mea drm1,2-17.ab1  TTTAAAATTC GGTTAGATTT TATAATGTTA TATTACG 276
mea drm1,2-18.ab1  TTTAAAATTC GGTTAGATTT TATAATGTTA TATTACG 276
```

```
                          20         40         60         80
meaisr            TTTAATGTAA ATATGTATTT GATGCATCTA ACATTTAGTA TCTAAACAAA TAAAAACAAA AAAAAA-GAA AAAAGCTCTT 79
mea cmt3-1.ab1    TTTAATGTAA ATATGTATTT GATGTATTTA ATATTTAGTA TTTAAATAAA TAAAAATAAA AAAAAA-GAA AAAAGTTTTT 79
mea cmt3-2.ab1    TTTAATGTAA ATATGTATTT GATGTATTTA ATATTTAGTA TTTAAATAAA TAAAAATAAA AAAAAA-GAA AAAAGTTTTT 79
mea cmt3-3.ab1    TTTAATGTAA ATATGTATTT GATGTATTTA ATATTTAGTA TTTAAATAAA TAAAAATAAA AAAAAAAGAA AAAAGTTTTT 80
mea cmt3-4.ab1    TTTAATGTAA ATATGTATTT GATGTATTTA ATATTTAGTA TTTAAATAAA TAAAAATAAA AAAAAA-GAA AAAAGTTTTT 79
mea cmt3-5.ab1    TTTAATGTAA ATATGTATTT GATGTATTTA ATATTTAGTA TTAAAATAAA TAAAAATAAA AAAAAA-GAA AAAAGTTTTT 79
mea cmt3-6.ab1    TTTAATGTAA ATATGTATTT GATGTATTTA ATATTTAGTA TTTAAATAAA TAAAAATAAA AAAAAA-GAA AAAAGTTTTT 79
mea cmt3-7.ab1    TTTAATGTAA ATATGTATTT GATGTATTTA ATATTTAGTA TTTAAATAAA TAAAAATAAA AAAAAA-GAA GAAAGTTTTT 79
mea cmt3-8.ab1    TTTAATGTAA ATATGTATTT GATGTATTTA ATATTTAGTA TTTAAATAAA TAAAAATAAA AAAAAA-GAA AAAAGTTTTT 79
mea cmt3-9.ab1    TTTAATGTAA ATATGTATTT GATGTATTTA ATATTTAGTA TTTAAATAAA TAAAAATAAA AAAAAA-GAA AAAAGTTTTT 79
mea cmt3-10.ab1   TTTAATGTAA ATATGTATTT GATGTATTTA ATATTTAGTA TTTAAATAAA TAAAAATAAA AAAAAA-GAA AAAAGTTTTT 79
mea cmt3-11.ab1   TTTAATGTAA ATATGTATTT GATGTATTTA ATATTTAGTA TTTAAATAAA TAAAAATAAA AAAAAA-GAA AAAAGTTTTT 79
mea cmt3-12.ab1   TTTAATGTAA ATATGTATTT GATGTATTTA ATATTTAGTA TTTAAATAAA TAAAAATAAA AAAAA--GAA AAAAGTTTTT 78
mea cmt3-13.ab1   TTTAATGTAA ATATGTATTT GATGTATTTA ATATTTAGTA TTTAAATAAA TAAAAATAAA AAAAAA-GAA AAAAGTTTTT 79
mea cmt3-14.ab1   TTTAATGTAA ATATGTATTT GATGTATTTA ATATTTAGTA TTTAAATAAA TAAAAATAAA AAAAAA-GAA AAAAGTTTTT 79
mea cmt3-15.ab1   TTTAATGTAA ATATGTATTT GATGTATTTA ATATTTAGTA TTTAAATAAA TAAAAATAAA AAAAAA-GAA AAAAGTTTTT 79
mea cmt3-16.ab1   TTTAATGTAA ATATGTATTT GATGTATTTA ATATTTAGTA TTTAAATAAA TAAAAATAAA AAAAAA-GAA AAAAGTTTTT 79
mea cmt3-17.ab1   TTTAATGTAA ATATGTATTT GATGTATTTA ATATTTAGTA TTTAAATAAA TAAAAATAAA AAAAAA-GAA AAAAGTTTTT 79
mea cmt3-18.ab1   TTTAATGTAA ATATGTATTT GATGTATTTA ATATTTAGTA TTTAAATAAA TAAAAATAAA AAAAAA-GAA AAAAGTTTTT 79
mea cmt3-19.ab1   TTTAATGTAA ATATGTATTT GATGTATTTA ATATTTAGTA TTTAAATAAA TAAAAATAAA AAAAAA-GAA AAAAGTTTTT 79
mea cmt3-20.ab1   TTTAATGTAA ATATGTATTT GATGTATTTA ATATTTAGTA TTTAAATAAA TAAAAATAAA AAAAAA-GAA AAAAGTTTTT 79

                          100        120        140        160
meaisr            TAAAATCCGA AAGTAACTAT TTTCAAAAAA TCTAAATTAT AAACTTAAAT GTTTGGAATC GCGAACGACT ATTGCTAAAT 159
mea cmt3-1.ab1    TAAAACTCGA AAGTAATTAT TTTTAAAAAA TTTAAATTAT AAATTTAAAT GTTTGGAATC GCGAACGATT ATTGTTAAAT 159
mea cmt3-2.ab1    TAAAATTCGA AAGTAATTAT TTTTAAAAAA TCTAAATTAT AAACTTAAAT GTTTGGAATC GCGAACGACT ATTGCTAAAT 159
mea cmt3-3.ab1    -AAAATTTGA AAGTAATTAT TCTTAAAAAA TTTAAATTAT AAATTTAAAT GTTTGGAATC GCGAACGATT ATTGTTAAAT 159
mea cmt3-4.ab1    TAAAATTTGA AAGTAATTAT TTTTAAAAAA TTTAAATTAT AAATTTAAAT GTTTGGAATT GCGAACGATT ATTGTTAAAT 159
mea cmt3-5.ab1    TAAGATTCGA AAGTAATTAT TTTTAAAAAA TTTAAATTAT AAATTTAAAT GTTTGGAATT GCGAACGATT ATTGTTAAAT 159
mea cmt3-6.ab1    TAAAATTTGA AAGTAATTAT TTTTAAAAAA TTTAAATTAT AAATTTAAAT GATTGGAATC GCGAACGACT ATTGTTAAAT 159
mea cmt3-7.ab1    TAAAATTCGA AAGTAATTAT TTTTAAAAAA TTTAAATTAT AAACTTAAAT GTTTGGAATC GCGAACGACT ATTGCTAAAT 159
mea cmt3-8.ab1    TAAAATTTGA AAGTAATTAT TTTTAAAAAA TTTAAATTAT AAATTTAAAT GTTTGGAATC GTGAACGATT ATTGCTAAAT 159
mea cmt3-9.ab1    TAAAATTCGA AAGTAATTAT TTTTAAAAAA TTTAAATTAT AAATTTAAAT GTTTGGAATC GTGAACGATT ATTGTTAAAT 159
mea cmt3-10.ab1   TAAAATTCGA AAGTAATTAT TTTTAAAAAA TTTAAATTAT AAATTTAAAT GTTTGGAATC GCGAATGATT ATTGTTAAAT 159
mea cmt3-11.ab1   TAAAATTCGA AAGTAATTAT TTTTAAAAAA TTTAAATTAT AAATTTAAAT GTTTGGAATC GCGAACGATT ATTGCTAAAT 159
mea cmt3-12.ab1   TAAAATTCGA AAGTAATTAT TTTTAAAAAA TTTAAATTAT AAACTTAAAT GTTTGGAATC GCGAACGATT ATTGTTAAAT 158
mea cmt3-13.ab1   TAAAATTCGA AAGTAATTAT TTTTAAAAAA TTTAAATTAT AAATTTAAAT GTTTGGAGTC GCGAACGATT ATTGTTAAAT 159
mea cmt3-14.ab1   TAAAATTTGA AAGTAATTAT TTTTAAAAAA TTTAAATTAT AAATTTAAAT GTTTGGAATC GCGAACGATT ATTGTTAAAT 159
mea cmt3-15.ab1   TAAAATTTGA AAGTAATTAT TTTTAAAAAA TTTAAATTAT AAATTTAAAT GTTTGGAATC GCGAACGATT ATTGTTAAAT 159
mea cmt3-16.ab1   TAAAATTTGA AAGTAATTAT TTTTAAAAAA TTTAAATTAT AAATTTAAAT GTTTGGAATC GCGAACGATT ATTGTTAAAT 159
mea cmt3-17.ab1   TAAAATTTGA AAGTAATTAT TTTTAAAAAA TTTAAATTAT AAATTTAAAT GTTTGGAATC GCGAACGATT ATTGTTAAAT 159
mea cmt3-18.ab1   TAAAATTTGA AAGTAATTAT TTTTAAAAAA TTTAAATTAT AAATTTAAAT GTTTGGAATC GCGAACGATT ATTGTTAAAT 159
mea cmt3-19.ab1   TAAAATTCGA AAGTAGTTAT TTTTAAAAAA TTTAAATTAT AAATTTAAAT GTTTGGAATC GCGAACGATT ATTGTTAAAT 159
mea cmt3-20.ab1   TAAAATTCGA AAGTAATTAT TTTTAAAAAA TTTAAATTAT AAATTTAAAT GTTTGGAATC GCGAACGATT ATTGTTAAAT 159

                          180        200        220        240
meaisr            ATAAATGCTA AATATACATG AAGATGTGAA AAACATGTTG GATTTGTGGA ATCGTTAATG ACCACGGTTA AATGGCGGGA 239
mea cmt3-1.ab1    ATAAATGTTA AATATATATG AAGATGTGAA AAATATGTTG GATTTGTGGA ATCGTTAATG ATTACGGTTA AATGGCGGGA 239
mea cmt3-2.ab1    ATAAATGCTA AATATACATG AAGATGTGAA AAACATGTTG GATTTGTGGA ATCGTTAATG ATTACGGTTA AATGGCGGGA 239
mea cmt3-3.ab1    ATAAATGTTA AATATATATG AAGATGTGAA AAATATGTTG GATTTGTGGA ATCGTTAATG ATTACGGTTA AATGGCGGGA 239
mea cmt3-4.ab1    ATAAATGTTA AATATATATG AAGATGTGAA AAATATGTTG GATTTGTGGA ATCGTTAATG ATTACGGTTA AATGGCGGGA 239
mea cmt3-5.ab1    ATAAATGTTA AATATATATG AAGATGTGAA AAATATGTTG GATTTGTGGA ATCGTTAATG ATTACGGTTA AATGGCGGGA 239
mea cmt3-6.ab1    ATAAATGCTA AATATACATG AAGATGTGAA AAACATGTTG GATTTGTGGA ATCGTTAATG ACCACGGTTA AATGGCGGGA 239
mea cmt3-7.ab1    ATAAATGTTA AATATATATG AAGATGTGAA AAATATGTTG GATTTGTGGA ATCGTTAATG ATCACGGTTA AATGGCGGGA 239
mea cmt3-8.ab1    ATAAATGTTA AATATATATG AAGATGTGAA AAATATGTTG GATTTGTGGA ATCGTTAATG ATTGCGGTTA AATGGCGGGA 239
mea cmt3-9.ab1    ATAAATGTTA AATATATATG AAGATGTGAA AAATATGTTG GATTTGTGGA ATCGTTAATG ATTACGGTTA AATGGCGGGA 239
mea cmt3-10.ab1   ATAAATGTTA AATATATATG AAGATGTGAA AAATATGTTG GATTTGTGGA ATCGTTAATG ATTACGGTTA AATGGCGGGA 239
mea cmt3-11.ab1   ATAAATGTTA AATATATATG AAGATGTGAA AAATATGTTG GATTTGTGGA GTCGTTAATG ATCACGGTTA AATGGCGGGA 239
mea cmt3-12.ab1   ATAAATGTTA AATGTATATG AAGATGTGAA AAATATGTTG GATTTGTGGA ATCGTTAATG ATTACGGTTA AATGGCGGGA 238
mea cmt3-13.ab1   ATAAATGTTA AATATATATG AAGATGTGAA AAATATGTTG GATTTGTGGA GTCGTTAATG ATTACGGTTA AATGGCGGGA 239
mea cmt3-14.ab1   ATAAATGTTA AATATACATG AAGATGTGAA AAACATGTTG GATTTGTGGA ATCGTTAATG ATTACGGTTA AATGGCGGGA 239
mea cmt3-15.ab1   ATAAATGTTA AATATATATG AAGATGTGAA AAATATGTTG GATTTGTGGA ATCGTTAATG ACCACGGTTA AATGGCGGGA 239
mea cmt3-16.ab1   ATAAATGTTA AATATATATG AAGATGTGAA AAATATGTTG GATTTGTGGA ATCGTTAATG ATTACGGTTA AATGGCGGGA 239
mea cmt3-17.ab1   ATAAATGTTA AATATATATG AAGATGTGAA AAATATGTTG GATTTGTGGA ATCGTTAATG ATTACGGTTA AATGGCGGGA 239
mea cmt3-18.ab1   ATAAATGTTA AATATATATG AAGATGTGAA AAATATGTTG GATTTGTGGA ATCGTTAATG ATCACGGTTA AATGGCGGGA 239
mea cmt3-19.ab1   ATAAATGTTA AATATATATG AAGATGTGAA AAATATGTTG GATTTGTGGA ATCGTTAATG ATTACGGTTA AATGGCGGGA 239
mea cmt3-20.ab1   ATAAATGTTA AATATATATG AAGATGTGAA AAATATGTTG GATTTGTGGA ATCGTTAATG ATTACGGTTA AATGGCGGGA 239
```

		260			
meaisr	TCCAAAATCC	GGTTAGATTT	CACAATGTCA	TATTACG	276
mea cmt3-1.ab1	TTTAAAATTC	GGTTAGATTT	TATAATGTTA	TATTACG	276
mea cmt3-2.ab1	TCCAAAATCC	GGTTAGATTT	CACAATGTTA	TATTACG	276
mea cmt3-3.ab1	TTCAAAATCT	GGTTAGATTT	CACAATGTCA	TATTACG	276
mea cmt3-4.ab1	TTTAAAATTC	GGTTAGATTT	TATAATGTTA	TATTACG	276
mea cmt3-5.ab1	TTCAAAATTC	GGTTAGATTT	TATAATGTTA	TATTACG	276
mea cmt3-6.ab1	TCCAAAATCC	GGTTAGATTT	CACAATGTCA	TATTACG	276
mea cmt3-7.ab1	TCCAAAATCC	GGTTAGATTT	CACAATGTCA	TATTACG	276
mea cmt3-8.ab1	TTCAAAATCC	GGTTAGATTT	CACAATGTCA	TATTACG	276
mea cmt3-9.ab1	TTTAAAATCC	GGTTAGATTT	CACAATGTTA	TATTACG	276
mea cmt3-10.ab1	TTTAAAATTC	GGTTAGATTT	TATAATGTTA	TATTACG	276
mea cmt3-11.ab1	TCCAAAATCC	GGTTAGATTT	CACAATGTCA	TATTACG	276
mea cmt3-12.ab1	TTTAAAATTC	GGTTAGATTT	TATAATGTTA	TATTACG	275
mea cmt3-13.ab1	TTTAAAATTC	GGTTAGATTT	TATAATGTTA	TATTACG	276
mea cmt3-14.ab1	TTTAAAATTT	GGTTAGATTT	TATAATGTTA	TATTACG	276
mea cmt3-15.ab1	TCCAAAATTC	GGTTAGATTT	CACAATGTCA	TATTACG	276
mea cmt3-16.ab1	TCCAAAATTC	GGTTAGATTT	CACAATGTTA	TATTACG	276
mea cmt3-17.ab1	TTCAAAATTC	GGTTAGATTT	TATAATGTTA	TATTACG	276
mea cmt3-18.ab1	TTTAAAATTC	GGTTAGATTT	TATAATGTTA	TATTACG	276
mea cmt3-19.ab1	TCCAAAATCC	GGTTAGATTT	CACAATGTCA	TATTACG	276
mea cmt3-20.ab1	TTTAAAATTC	GGTTAGATTT	TATAATGTTA	TATTACG	276

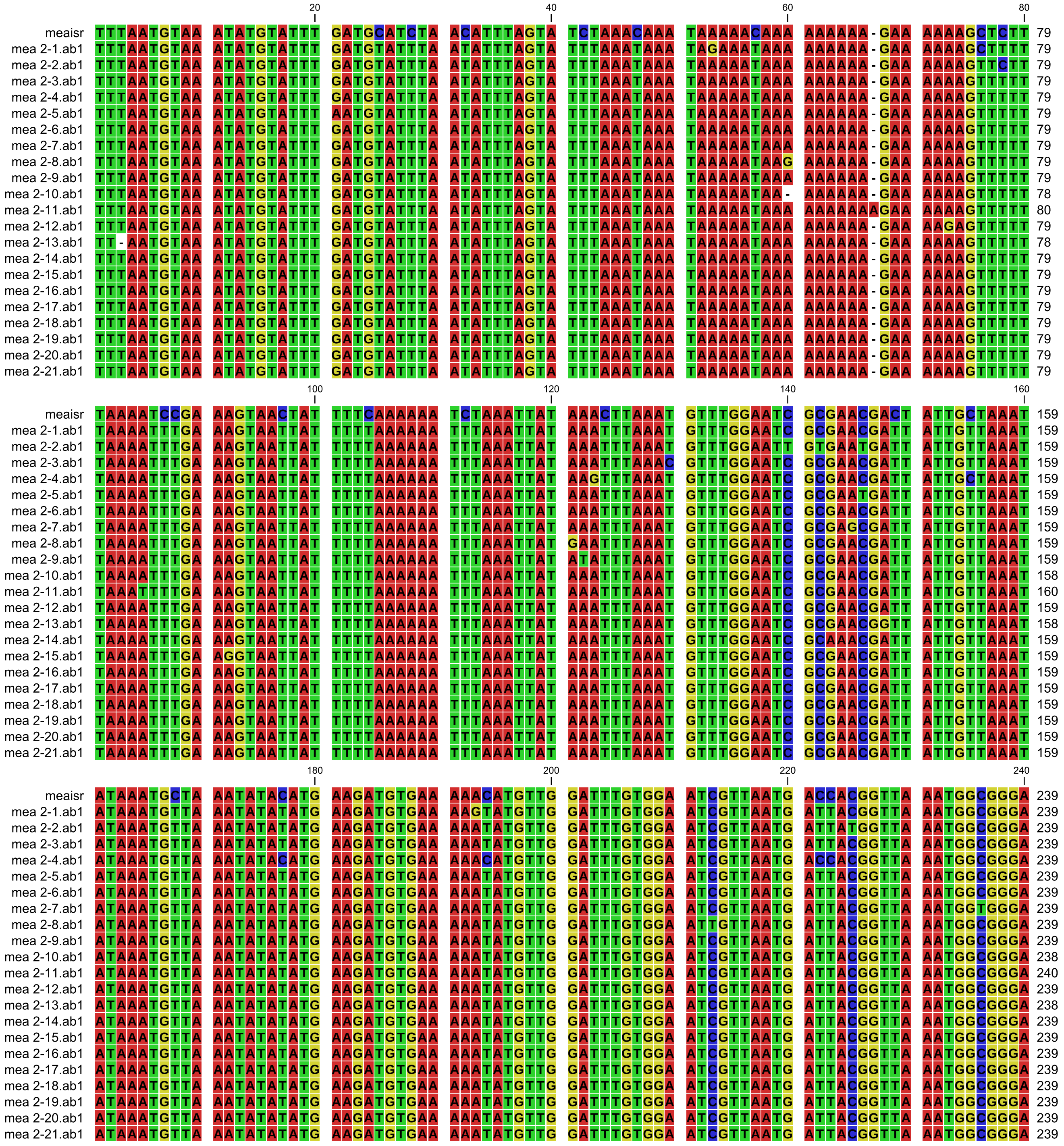
20 40 60 80
meaisr TTTAATGTAA ATATGTATTT GATGCATCTA ACATTTAGTA TCTAAACAAA TAAAAACAAA AAAAAA-GAA AAAAGCTCTT 79
mea 2-1.ab1 TTTAATGTAA ATATGTATTT GATGTATTTA ATATTTAGTA TTTAAATAAA TAGAAATAAA AAAAAA-GAA AAAAGCTTTT 79
mea 2-2.ab1 TTTAATGTAA ATATGTATTT GATGTATTTA ATATTTAGTA TTTAAATAAA TAAAAATAAA AAAAAA-GAA AAAAGTTCTT 79
mea 2-3.ab1 TTTAATGTAA ATATGTATTT GATGTATTTA ATATTTAGTA TTTAAATAAA TAAAAATAAA AAAAAA-GAA AAAAGTTTTT 79
mea 2-4.ab1 TTTAATGTAA ATATGTATTT GATGTATTTA ATATTTAGTA TTTAAATAAA TAAAAATAAA AAAAAA-GAA AAAAGTTTTT 79
mea 2-5.ab1 TTTAATGTAA ATATGTATTT AATGTATTTA ATATTTAGTA TTTAAATAAA TAAAAATAAA AAAAAA-GAA AAAAGTTTTT 79
mea 2-6.ab1 TTTAATGTAA ATATGTATTT GATGTATTTA ATATTTAGTA TTTAAATAAA TAAAAATAAA AAAAAA-GAA AAAAGTTTTT 79
mea 2-7.ab1 TTTAATGTAA ATATGTATTT GATGTATTTA ATATTTAGTA TTTAAATAAA TAAAAATAAA AAAAAA-GAA AAAAGTTTTT 79
mea 2-8.ab1 TTTAATGTAA ATATGTATTT GATGTATTTA ATATTTAGTA TTTAAATAAA TAAAAATAAG AAAAAA-GAA AAAAGTTTTT 79
mea 2-9.ab1 TTTAATGTAA ATATGTATTT GATGTATTTA ATATTTAGTA TTTAAATAAA TAAAAATAAA AAAAAA-GAA AAAAGTTTTT 79
mea 2-10.ab1 TTTAATGTAA ATATGTATTT GATGTATTTA ATATTTAGTA TTTAAATAAA TAAAAATAA- AAAAAA-GAA AAAAGTTTTT 78
mea 2-11.ab1 TTTAATGTAA ATATGTATTT GATGTATTTA ATATTTAGTA TTTAAATAAA TAAAAATAAA AAAAAAAGAA AAAAGTTTTT 80
mea 2-12.ab1 TTTAATGTAA ATATGTATTT GATGTATTTA ATATTTAGTA TTTAAATAAA TAAAAATAAA AAAAAA-GAA AAGAGTTTTT 79
mea 2-13.ab1 TT-AATGTAA ATATGTATTT GATGTATTTA ATATTTAGTA TTTAAATAAA TAAAAATAAA AAAAAA-GAA AAAAGTTTTT 78
mea 2-14.ab1 TTTAATGTAA ATATGTATTT GATGTATTTA ATATTTAGTA TTTAAATAAA TAAAAATAAA AAAAAA-GAA AAAAGTTTTT 79
mea 2-15.ab1 TTTAATGTAA ATATGTATTT GATGTATTTA ATATTTAGTA TTTAAATAAA TAAAAATAAA AAAAAA-GAA AAAAGTTTTT 79
mea 2-16.ab1 TTTAATGTAA ATATGTATTT GATGTATTTA ATATTTAGTA TTTAAATAAA TAAAAATAAA AAAAAA-GAA AAAAGTTTTT 79
mea 2-17.ab1 TTTAATGTAA ATATGTATTT GATGTATTTA ATATTTAGTA TTTAAATAAA TAAAAATAAA AAAAAA-GAA AAAAGTTTTT 79
mea 2-18.ab1 TTTAATGTAA ATATGTATTT GATGTATTTA ATATTTAGTA TTTAAATAAA TAAAAATAAA AAAAAA-GAA AAAAGTTTTT 79
mea 2-19.ab1 TTTAATGTAA ATATGTATTT GATGTATTTA ATATTTAGTA TTTAAATAAA TAAAAATAAA AAAAAA-GAA AAAAGTTTTT 79
mea 2-20.ab1 TTTAATGTAA ATATGTATTT GATGTATTTA ATATTTAGTA TTTAAATAAA TAAAAATAAA AAAAAA-GAA AAAAGTTTTT 79
mea 2-21.ab1 TTTAATGTAA ATATGTATTT GATGTATTTA ATATTTAGTA TTTAAATAAA TAAAAATAAA AAAAAA-GAA AAAAGTTTTT 79
100 120 140 160
meaisr TAAAATCCGA AAGTAACTAT TTTCAAAAAA TCTAAATTAT AAACTTAAAT GTTTGGAATC GCGAACGACT ATTGCTAAAT 159
mea 2-1.ab1 TAAAATTTGA AAGTAATTAT TTTTAAAAAA TTTAAATTAT AAATTTAAAT GTTTGGAATC GCGAACGATT ATTGTTAAAT 159
mea 2-2.ab1 TAAAATTTGA AAGTAATTAT TTTTAAAAAA TTTAAATTAT AAATTTAAAT GTTTGGAATT GTGAATGATT ATTGTTAAAT 159
mea 2-3.ab1 TAAAATTTGA AAGTAATTAT TTTTAAAAAA TTTAAATTAT AAATTTAAAC GTTTGGAATC GCGAACGATT ATTGTTAAAT 159
mea 2-4.ab1 TAAAATTTGA AAGTAATTAT TTTTAAAAAA TTTAAATTAT AAGTTTAAAT GTTTGGAATC GCGAACGATT ATTGCTAAAT 159
mea 2-5.ab1 TAAAATTTGA AAGTAATTAT TTTTAAAAAA TTTAAATTAT AAATTTAAAT GTTTGGAATC GCGAATGATT ATTGTTAAAT 159
mea 2-6.ab1 TAAAATTTGA AAGTAATTAT TTTTAAAAAA TTTAAATTAT AAATTTAAAT GTTTGGAATC GCGAACGATT ATTGTTAAAT 159
mea 2-7.ab1 TAAAATTTGA AAGTAATTAT TTTTAAAAAA TTTAAATTAT AAATTTAAAT GTTTGGAATC GCGAGCGATT ATTGTTAAAT 159
mea 2-8.ab1 TAAAATTTGA AAGTAATTAT TTTTAAAAAA TTTAAATTAT GAATTTAAAT GTTTGGAATC GCGAACGATT ATTGTTAAAT 159
mea 2-9.ab1 TAAAATTTGA AAGTAATTAT TTTTAAAAAA TTTAAATTAT ATATTTAAAT GTTTGGAATC GCGAACGATT ATTGTTAAAT 159
mea 2-10.ab1 TAAAATTTGA AAGTAATTAT TTTTAAAAAA TTTAAATTAT AAATTTAAAT GTTTGGAATC GCGAACGATT ATTGTTAAAT 158
mea 2-11.ab1 TAAATTTTGA AAGTAATTAT TTTTAAAAAA TTTAAATTAT AAATTTAAAT GTTTGGAATC GCGAACGATT ATTGTTAAAT 160
mea 2-12.ab1 TAAAATTTGA AAGTAATTAT TTTTAAAAAA TTTAAATTAT AAATTTAAAT GTTTGGAATC GCGAACGATT ATTGTTAAAT 159
mea 2-13.ab1 TAAAATTTGA AAGTAATTAT TTTTAAAAAA TTTAAATTAT AAATTTAAAT GTTTGGAATC GCGAACGGTT ATTGTTAAAT 158
mea 2-14.ab1 TAAAATTTGA AAGTAATTAT TTTTAAAAAA TTTAAATTAT AAATTTAAAT GTTTGGAATC GCAAACGATT ATTGTTAAAT 159
mea 2-15.ab1 TAAAATTTGA AGGTAATTAT TTTTAAAAAA TTTAAATTAT AAATTTAAAT GTTTGGAATC GCGAACGATT ATTGTTAAAT 159
mea 2-16.ab1 TAAAATTTGA AAGTAATTAT TTTTAAAAAA TTTAAATTAT AAATTTAAAT GTTTGGAATC GCGAACGATT ATTGTTAAAT 159
mea 2-17.ab1 TAAAATTTGA AAGTAATTAT TTTTAAAAAA TTTAAATTAT AAATTTAAAT GTTTGGAATC GCGAACGATT ATTGTTAAAT 159
mea 2-18.ab1 TAAAATTTGA AAGTAATTAT TTTTAAAAAA TTTAAATTAT AAATTTAAAT GTTTGGAATC GCGAACGATT ATTGTTAAAT 159
mea 2-19.ab1 TAAAATTTGA AAGTAATTAT TTTTAAAAAA TTTAAATTAT AAATTTAAAT GTTTGGAATC GCGAACGATT ATTGTTAAAT 159
mea 2-20.ab1 TAAAATTTGA AAGTAATTAT TTTTAAAAAA TTTAAATTAT AAATTTAAAT GTTTGGAATC GCGAACGATT ATTGTTAAAT 159
mea 2-21.ab1 TAAAATTTGA AAGTAATTAT TTTTAAAAAA TTTAAATTAT AAATTTAAAT GTTTGGAATC GCGAACGATT ATTGTTAAAT 159
180 200 220 240
meaisr ATAAATGCTA AATATACATG AAGATGTGAA AAACATGTTG GATTTGTGGA ATCGTTAATG ACCACGGTTA AATGGCGGGA 239
mea 2-1.ab1 ATAAATGTTA AATATATATG AAGATGTGAA AAGTATGTTG GATTTGTGGA ATCGTTAATG ATTACGGTTA AATGGCGGGA 239
mea 2-2.ab1 ATAAATGTTA AATATATATG AAGATGTGAA AAATATGTTG GATTTGTGGA ATCGTTAATG ATTATGGTTA AATGGCGGGA 239
mea 2-3.ab1 ATAAATGTTA AATATATATG AAGATGTGAA AAATATGTTG GATTTGTGGA ATCGTTAATG ATTACGGTTA AATGGCGGGA 239
mea 2-4.ab1 ATAAATGTTA AATATACATG AAGATGTGAA AAACATGTTG GATTTGTGGA ATCGTTAATG ACCACGGTTA AATGGCGGGA 239
mea 2-5.ab1 ATAAATGTTA AATATATATG AAGATGTGAA AAATATGTTG GATTTGTGGA ATCGTTAATG ATTACGGTTA AATGGCGGGA 239
mea 2-6.ab1 ATAAATGTTA AATATATATG AAGATGTGAA AAATATGTTG GATTTGTGGA ATCGTTAATG ATTACGGTTA AATGGCGGGA 239
mea 2-7.ab1 ATAAATGTTA AATATATATG AAGATGTGAA AAATATGTTG GATTTGTGGA ATCGTTAATG ATTACGGTTA AATGGTGGGA 239
mea 2-8.ab1 ATAAATGTTA AATATATATG AAGATGTGAA AAATATGTTG GATTTGTGGA ATTGTTAATG ATTACGGTTA AATGGCGGGA 239
mea 2-9.ab1 ATAAATGTTA AATATATATG AAGATGTGAA AAATATGTTG GATTTGTGGA ATCGTTAATG ATTACGGTTA AATGGCGGGA 239
mea 2-10.ab1 ATAAATGTTA AATATATATG AAGATGTGAA AAATATGTTG GATTTGTGGA ATCGTTAATG ATTACGGTTA AATGGCGGGA 238
mea 2-11.ab1 ATAAATGTTA AATATATATG AAGATGTGAA AAATATGTTG GATTTGTGGA ATCGTTAATG ATTACGGTTA AATGGCGGGA 240
mea 2-12.ab1 ATAAATGTTA AATATATATG AAGATGTGAA AAATATGTTG GATTTGTGGA ATCGTTAATG ATTACGGTTA AATGGCGGGA 239
mea 2-13.ab1 ATAAATGTTA AATATATATG AAGATGTGAA AAATATGTTG GATTTGTGGA ATCGTTAATG ATTACGGTTA AATGGCGGGA 238
mea 2-14.ab1 ATAAATGTTA AATATATATG AAGATGTGAA AAATATGTTG GATTTGTGGA ATCGTTAATG ATTACGGTTA AATGGCGGGA 239
mea 2-15.ab1 ATAAATGTTA AATATATATG AAGATGTGAA AAATATGTTG GATTTGTGGA ATCGTTAATG ATTACGGTTA AATGGCGGGA 239
mea 2-16.ab1 ATAAATGTTA AATATATATG AAGATGTGAA AAATATGTTG GATTTGTGGA ATCGTTAATG ATTACGGTTA AATGGCGGGA 239
mea 2-17.ab1 ATAAATGTTA AATATATATG AAGATGTGAA AAATATGTTG GATTTGTGGA ATCGTTAATG ATTACGGTTA AATGGCGGGA 239
mea 2-18.ab1 ATAAATGTTA AATATATATG AAGATGTGAA AAATATGTTG GATTTGTGGA ATCGTTAATG ATTACGGTTA AATGGCGGGA 239
mea 2-19.ab1 ATAAATGTTA AATATATATG AAGATGTGAA AAATATGTTG GATTTGTGGA ATCGTTAATG ATTACGGTTA AATGGCGGGA 239
mea 2-20.ab1 ATAAATGTTA AATATATATG AAGATGTGAA AAATATGTTG GATTTGTGGA ATCGTTAATG ATTACGGTTA AATGGCGGGA 239
mea 2-21.ab1 ATAAATGTTA AATATATATG AAGATGTGAA AAATATGTTG GATTTGTGGA ATCGTTAATG ATTACGGTTA AATGGCGGGA 239

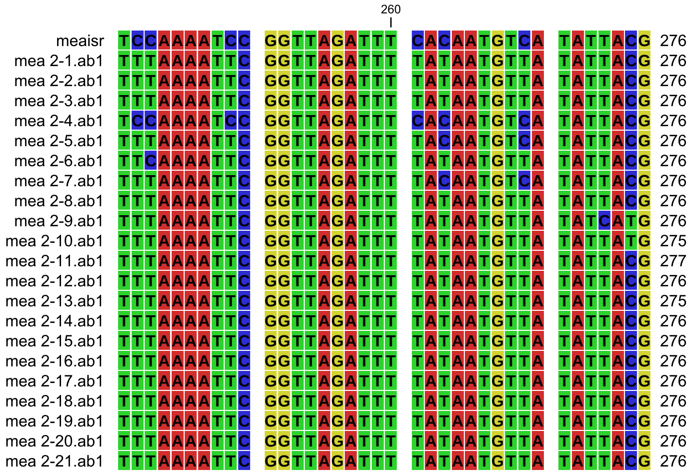
260
meaisr TCCAAAATCC GGTTAGATTT CACAATGTCA TATTACG 276
mea 2-1.ab1 TTTAAAATTC GGTTAGATTT TATAATGTTA TATTACG 276
mea 2-2.ab1 TTTAAAATTC GGTTAGATTT TATAATGTTA TATTACG 276
mea 2-3.ab1 TTTAAAATTC GGTTAGATTT TATAATGTTA TATTACG 276
mea 2-4.ab1 TCCAAAATCC GGTTAGATTT CACAATGTCA TATTACG 276
mea 2-5.ab1 TTTAAAATTC GGTTAGATTT TACAATGTCA TATTACG 276
mea 2-6.ab1 TTCAAAATTC GGTTAGATTT TATAATGTTA TATTACG 276
mea 2-7.ab1 TTTAAAATTC GGTTAGATTT TACAATGTCA TATTACG 276
mea 2-8.ab1 TTTAAAATTC GGTTAGATTT TATAATGTTA TATTACG 276
mea 2-9.ab1 TTTAAAATTC GGTTAGATTT TATAATGTTA TATCATG 276
mea 2-10.ab1 TTTAAAATTC GGTTAGATTT TATAATGTTA TATTATG 275
mea 2-11.ab1 TTTAAAATTC GGTTAGATTT TATAATGTTA TATTACG 277
mea 2-12.ab1 TTTAAAATTC GGTTAGATTT TATAATGTTA TATTACG 276
mea 2-13.ab1 TTTAAAATTC GGTTAGATTT TATAATGTTA TATTACG 275
mea 2-14.ab1 TTTAAAATTC GGTTAGATTT TATAATGTTA TATTACG 276
mea 2-15.ab1 TTTAAAATTC GGTTAGATTT TATAATGTTA TATTACG 276
mea 2-16.ab1 TTTAAAATTC GGTTAGATTT TATAATGTTA TATTACG 276
mea 2-17.ab1 TTTAAAATTC GGTTAGATTT TATAATGTTA TATTACG 276
mea 2-18.ab1 TTTAAAATTC GGTTAGATTT TATAATGTTA TATTACG 276
mea 2-19.ab1 TTTAAAATTC GGTTAGATTT TATAATGTTA TATTACG 276
mea 2-20.ab1 TTTAAAATTC GGTTAGATTT TATAATGTTA TATTACG 276
mea 2-21.ab1 TTTAAAATTC GGTTAGATTT TATAATGTTA TATTACG 276

```
                    20                    40                    60                    80
meaisr       TTTAATGTAA ATATGTATTT GATGCATCTA ACATTTAGTA TCTAAACAAA TAAAAACAAA AAAAAAGAAA AAAGCTCTTT 80
mea 9-1.ab1  TTTAATGTAA ATATGTATTT GATGCATTTA ATATTTAGTA TTTAAATAAA TAAAAATAAA AAAAAAGAAA AAAGTTTTTT 80
mea 9-2.ab1  TTTAATGTAA ATATGTATTT GATGTATTTA ATATTTAGTA TTTAAATAAA TAAAAATAAA AAAAAAGAAA AAAGTTTTTT 80
mea 9-3.ab1  TTTAATGTAA ATATGTATTT GATGTATTTA ATATTTAGTA TTTAAATAAA TAAAAATAAA AAAAAAGAAA AAAGTTTTTT 80
mea 9-4.ab1  TTTAATGTAA ATATGTATTT GATGTATTTA ATATTTAGTA TTTAAATAAA TAAAAATAAA AAAAAAGAAA AAAGTTTTTT 80
mea 9-5.ab1  TTTAATGTAA ATATGTATTT GATGTATTTA ATATTTAGTA TTTAAATAAA TAAAAATAAA AAAAAAGAAA AAAGTTTTTT 80
mea 9-6.ab1  TTTAATGTAA ATATGTATTT GATGTATTTA ATATTTAGTA TTTAAATAAA TAAAGATAAA AAAAAAGAAA AAAGTTTTTT 80
mea 9-7.ab1  TTTAATGTAA ATATGTATTT GATGTATTTA ATATTTAGTA TTTAAATAAA TAAAAATAAA AAAAAAGAAA AAAGTTTTTT 80
mea 9-8.ab1  TTTAATGTAA ATATGTATTT GATGTATTTA ATATTTAGTA TTTAAATAAA TAAAAATAAA AAAAAAGAAA AAAGTTTTTT 80
mea 9-9.ab1  TTTAATGTAA ATATGTATTT GATGTATTTA ATATTTAGTA TTTAAATAAA TAAAAATAAA AAAAAAGAAA AAAGTTTTTT 80
mea 9-10.ab1 TTTAATGTAA ATATGTATTT GATGTATTTA ATATTTAGTA TTTAAATAAA TAAAAATAAA AAAAAAGAGA AAAGTTTTTT 80
mea 9-11.ab1 TTTAATGTAA ATATGTATTT GATGTATTTA ATATTTAGTA TTTAAATAAA TAAAAATAAA AAAAAAGAAA AAAGTTTTTT 80
mea 9-12.ab1 TTTAATGTAA ATATGTATTT GATGTATTTA ATATTTAGTA TTTAAATAAA TAAAAATAAA AAAAAAGAAA AAAGTTTTTT 80
mea 9-13.ab1 TTTAATGTAA ATATGTATTT GATGTATTTA ATATTTAGTA TTTAAATAAA TAAAAATAAA AAAAAAGAAA AAAGTTTTTT 80
mea 9-14.ab1 TTTAATGTAA ATATGTATTT GATGTATTTA ATATTTAGTA TTTAAATAAA TAAAAATAAA AAAAAAGAAA AAAGTTTTTT 80
mea 9-15.ab1 TTTAATGTAA ATATGTATTT GATGTATTTA ATATTTAGTA TTTAAATAAA TAAAAATAAA AAAAAAGAAA AAAGTTTTTT 80
mea 9-16.ab1 TTTAATGTAA ATATGTATTT GATGTATTTA ATATTTAGTA TTTAAATAAA TAAAAATAAA AAAAAAGAAA AAAGTTTTTT 80

                   100                   120                   140                   160
meaisr       AAAATCCGAA AGTAACTATT TTCAAAAAAT CTAAATTATA AACTTAAATG TTTGGAATCG CGAACGACTA TTGCTAAATA 160
mea 9-1.ab1  AAGATTTGAA AGTAATTATT CTTAAAAAAT TTAAATTATA AATTTAAATG TTTGGAATCG CGAACGACTA TTGTTAAATA 160
mea 9-2.ab1  AAAATTCGAA GGTAACTATT TTTAAAAAAT TTAAATTATA AATTTAAATG TTTGGAATTG CGAACGATTA TTGATAAATA 160
mea 9-3.ab1  AAAATTCGAA AGTAATTATT TTTAAAAAAT CTAAATTATA AATTTAAATG TTTGGAATCG CGAACGACTA TTGCTAAATA 160
mea 9-4.ab1  AAATTTCGAA AGTAATTATT TTCAAAAAAT TTAAATTATA AATTTAAATG TTTGGAATCG CGAACGATTA TTGTTAAATA 160
mea 9-5.ab1  AAAATTCGAA AGTAATTATT TTTAAAAAAT TTAAATTATA AATTTAAATG TTTGGAATCG CGAATGATTA TTGTTAAATA 160
mea 9-6.ab1  AAAATTTGAA AGTAATTATT TTTAAAAAAT TTAAATTATA AATTTAAATG TTTGGAATCG CGAATGATTA TTGTTAAATA 160
mea 9-7.ab1  AAAATTTGAA AGTAATTATT TTTAAAAAAT TTAAATTATA AATTTAAATG TTTGGAATCG CGAACGATTA TTGTTAAATA 160
mea 9-8.ab1  AAAATTTGAA AGTAATTATT TTTAAAAAAT TTAAATTATA AATTTAAATG TTTGGAATCG CGAACGATTA TTGTTAAATA 160
mea 9-9.ab1  AAAATTCGAA AGTAATTGTT TTTAAAAAAT TTAAATTATA AATTTAAATG TTTGGAATCG CGAGCGATTA TTGTTAAATA 160
mea 9-10.ab1 AAAATTTGAA AGTAATTATT TTTAAAAAAT TTAAATTATA AATTTAAATG TTTGGAATCG CGAACGATTA TTGTTAAATA 160
mea 9-11.ab1 AAAATTCGAA AGTAATTATT TTTAAAAAAT TTAAATTATA AATTTAAATG TTTGGAATCG CGAACGATTA TTGTTAAATA 160
mea 9-12.ab1 AAAATTCGAA AGTAATTATT TTTGAAAAAT TTAAATTATA AATTTAAATG TTTGGAATCG CGAACGATTA TTGTTAAATA 160
mea 9-13.ab1 AAAATTCGAA AGTAATTATT TTTAAAAAAT TTAAATTATA AATTTAAATG TTTGGAATCG CGAACGATTA TTGTTAAATA 160
mea 9-14.ab1 AAAATTCGAA AGTAATTATT TTTAAAAAAT TTAAATTATA AATTTAAATG TTTGGAATCG CGAACGATTA TTGTTAAATA 160
mea 9-15.ab1 AAAATTTGAA AGTAATTATT TTTAAAAAAT TTAAATTATA AATTTAAATG TTTGGAATCG CGAACGATTA TTGTTAAATA 160
mea 9-16.ab1 AAAATTCGAA AGTAATTATT TTTAAAAAAT TTAAATTATA AATTTAAATG TTTGGAATCG CGAACGATTA TTGTTAAATA 160

                   180                   200                   220                   240
meaisr       TAAATGCTAA ATATACATGA AGATGTGAAA AACATGTTGG ATTTGTGGAA TCGTTAATGA CCACGGTTAA ATGGCGGGAT 240
mea 9-1.ab1  TAAATGTTAA ATATATATGA AGATGTGAAA AATATGTTGG ATTTGTGGAA TCGTTAATGA TTACGGTTAA ATGGCGGGAT 240
mea 9-2.ab1  TAAATGCTAA ATATACATGA AGATGTGAAA AATATGTTGG GTTTGTGGAA TCGTTAATGA CCACGGTTAA ATGGCGGGAT 240
mea 9-3.ab1  TAAATGCTAA ATATACATGA AGATGTGAAA AATATGTTGG ATTTGTGGAA TCGTTAATGA TTACGGTTAA ATGGCGGGAT 240
mea 9-4.ab1  TAAATGTTAA ATATATATGA AGATGTGAAA AATATGTTGG ATTTGTGGAA TCGTTAATGA TTACGGTTAA ATGGCGGGAT 240
mea 9-5.ab1  TAAATGTTAA ATATATATGA AGATGTGAAA AATATGTTGG ATTTGTGGAA TCGTTAATGA TTACGGTTAA ATGGCGGGAT 240
mea 9-6.ab1  TAAATGTTAA ATATATATGA AGATGTGAAA AATATGTTGG ATTTGTGGAA TCGTTAATGA TTACGGTTAA ATGGCGGGAT 240
mea 9-7.ab1  TAAATGTTAA ATATATATGA AGATGTGAAA AACATGTTGG ATTTGTGGAA TCGTTAATGA TCACGGTTAA ATGGCGGGAT 240
mea 9-8.ab1  TAAATGTTAA ATATATATGA AGATGTGAAA AATATGTTGG ATTTGTGGAA TTGTTAATGA TTATGGTTAA ATGGCGGGAT 240
mea 9-9.ab1  TAAATGTTAA ATATATATGA AGATGTGAAA AATATGTTGG ATTTGTGGAA TCGTTAACGA TTACGGTTAA ATGGCGGGAT 240
mea 9-10.ab1 TAAATGTTAA ATATATATGA AGATGTGAAG AATATGTTGG ATTTGTGGAA TCGTTAATGA TTACGGTTAA ATGGCGGGAT 240
mea 9-11.ab1 TAAATGTTAA ATATATATGA AGATGTGAAA AATATGTTGG ATTTGTGGAA TCGTTAATGA TTACGGTTAA ATGGCGGGAT 240
mea 9-12.ab1 TAAATGTTAA ATATATATGA AGATGTGAAA AATATGTTGG ATTTGTGGAA TCGTTAATGA TTACGGTTAA ATGGCGGGAT 240
mea 9-13.ab1 TAAATGTTAA ATATATATGA AGATGTGAAA AATATGTTGG ATTTGTGGAA TCGTT-ATGA TTACGGTTAA ATGGCGGGAT 239
mea 9-14.ab1 TAAATGTTAA ATATATATGA AGATGTGAAA AATATGTTGG ATTTGTGGAA TCGTTAATGA TTACGGTTAA ATGGCGGGAT 240
mea 9-15.ab1 TAAATGTTAA ATATATATGA AGATGTGAAA AATATGTTGG ATTTGTGGAA TCGTTGATGA TTACGGTTAA ATGGCGGGAT 240
mea 9-16.ab1 TAAATGTTAA ATATATATGA AGATGTGAAA AATATGTTGG ATTTGTGGAA TCGTTAATGA TTACGGTTAA ATGGCGGGAT 240

                   260
meaisr       CCAAAATCCG GTTAGATTTC ACAATGTCAT ATTACG 276
mea 9-1.ab1  CTAAAATTCG GTTAGATTTT ATAATGTTAT ATTATG 276
mea 9-2.ab1  TTAAAATTCG GTTAGATTTT ACAATGTCGT ATTACG 276
mea 9-3.ab1  TTAAAATTCG GTTAGATTTT ATAATGTTAT ATTACG 276
mea 9-4.ab1  CCAAAATCCG GTTAGATTTC ACAATGTCAT ATTACG 276
mea 9-5.ab1  TTAAAATTCG GTTAGATTTT ATAATGTTAT ATTACG 276
mea 9-6.ab1  TTAAAATTCG GTTAGATTTT ATAATGTTAT ATTACG 276
mea 9-7.ab1  CCAAAATCCG GTTAGATTTC ACAATGTCAT ATTACG 276
mea 9-8.ab1  TTAAAATTCG GTAAGATTTT ATAATGTTAT ATTATG 276
mea 9-9.ab1  TTAAAATTCG GTTAGATTTT ATAATGTTAT ATTACG 276
mea 9-10.ab1 CCAAAATCCG GTTAGATTTC ACAATGTCAT ATTACG 276
mea 9-11.ab1 TCAAAATTCG GTTAGATTTT ATAATGTTAT ATTACG 276
mea 9-12.ab1 TTAAAATTCG GTTAGATTTT ATAATGTTAT ATTATG 276
mea 9-13.ab1 TTAAAATTCG GTTAGATTTC ATAATGTTAT ATTACG 275
mea 9-14.ab1 TCAAAATCCG GTTAGATTTC ACAATGTCAT ATTACG 276
mea 9-15.ab1 TTAAAATTCG GTTAGATTTT ATAATGTTAT ATTACG 276
mea 9-16.ab1 TTAAAATTCG GTTAGATTTT ATAATGTTAT ATTACG 276
```

```
                                    20                    40                    60                    80
meaisr         -TTTAATGTA AATATGTATT TGATGCATCT AACATTTAGT ATCTAAACAA ATAAAAACAA AAAAAAAGAA AAAAGCTCTT  79
mea 92-1.ab1   TTTTAATGTA AATATGTATT TGATGTATTT AATATTTAGT ATTTAAATAA ATAAAAATAA AAAAAAAGAA AAAAGTTTTT  80
mea 92-2.ab1   TTTTAATGTA AATATGTATT TGATGTATTT AATATTTAGT ATTTAAATAA ATAAAAATAA AAAAAAAGAA AAAAGTTTTT  80
mea 92-3.ab1   TTTTAATGTA AATATGTATT TGATGTATTT AATATTTAGT ATTTAAATAA ATAAAAATAA AAAAAAAGAA AAAAGTTTTT  80
mea 92-4.ab1   TTTTAATGTA AATATGTATT TGATGTATTT AATATTTAGT ATTTAAATAA ATAAAAATAA AAAAAAAGAA AAAAGTTTTT  80
mea 92-5.ab1   TTTTAATGTA AATATGTATT TGATGTATTT AATATTTAGT ATTTAAATAA ATAAAAATAA AAAAAAAGAA AAAAGTTTTT  80
mea 92-6.ab1   TTTTAATGTA AATATGTATT TGATGTATTT AATATTTAGT ATTTAAATAA ATAAAAATAA AAAAAAAGAA AAAAGTTTTT  80
mea 92-7.ab1   TTTTAATGTA AATATGTATT TGATGTATTT AATATTTAGT ATTTAAATAA ATAAAAATAA AAAAAAAGAA AAAAGTTTTT  80
mea 92-8.ab1   TTTTAATGTA AATATGTATT TGATGTATTT AATATTTAGT ATTTAAATAA ATAAAAATAA AAAAAAAGAA AAAAGTTTTT  80
mea 92-9.ab1   TTTTAATGTA AATATGTATT TGATGTATTT AATATTTAGT ATTTAAATAA ATAAAAATAA AAAAAAAGAA AAAAGTTTTT  80
mea 92-10.ab1  TTTTAATGTA AATATGTATT TGATGTATTT AATATTTAGT ATTTAAATAA ATAAAAATAA AAAAAAAGAA GAAAGTTTTT  80
mea 92-11.ab1  TTTTAATGTA AATATGTATT TGATGTATTT AATATTTAGT ATTTAAATAA ATAAAAATAA AAAAAAAGAA AAAAGTTTTT  80
mea 92-12.ab1  TTTTAATGTA AATATGTATT TGATGTATTT AATATTTAGT ATTTAAATAA ATAAAAATAA AAAAAAAGAA AAAAGTTTTT  80
mea 92-13.ab1  TTTTAATGTA AATATGTATT TGATGTATTT AATATTTAGT ATTTAAATAA ATAAAAATAA AAAAAAAGAA AAAAGTTTTT  80
mea 92-14.ab1  TTTTAATGTA AATATGTATT TGATGTATTT AATATTTAGT ATTTAAATAA ATAAAAATAA AAAAAAAGAA AAAAGTTTTT  80
mea 92-15.ab1  TTTTAATGTA AATATGTATT TGATGTATTT AATATTTAGT ATTTAAATAA ATAAAAATAA AAAAAAAGAA AAAAGTTTTT  80
mea 92-16.ab1  TTTTAATGTA AATATGTATT TGATGTATTT AATATTTAGT ATTTAAATAA ATAAAAATAA AAAAAAAGAA AAAAGTTTTT  80
mea 92-17.ab1  TTTTAATGTA AATATGTATT TGATGTATTT AATATTTAGT ATTTAAATAA ATAAAAATAA AAAAAAAGAA AAAAGTTTTT  80
mea 92-18.ab1  TTTTAATGTA AATATGTATT TGATGTATTT AATATTTAGT ATTTAAATAA ATAAAAATAA AAAAAAAGAA AAAAGTTTTT  80
mea 92-19.ab1  TTTTAATGTA AATATGTATT TGATGTATTT AATATTTAGT ATTTAAATAA ATAAAAATAA AAAAAAAGAA AAAAGTTTTT  80
mea 92-20.ab1  TTTTAATGTA AATATGTATT TGATGTATTT AATATTTAGT ATTTAAATAA ATAAAAATAA AAAAAAAGAA AAAAGTTTTT  80
mea 92-21.ab1  TTTTAATGTA AATATGTATT TGATGTATTT AATATTTAGT ATTTAAATAA ATAAAAATAA AAAAAAAGAA AAAAGTTTTT  80
mea 92-22.ab1  TTTTAATGTA AATATGTATT TGATGTATTT AATATTTAGT ATTTAAATAA ATAAAAATAA AAAAAAAGAA AAAAGTTTTT  80
mea 92-23.ab1  TTTTAATGTA AATATGTATT TGATGTATTT AATATTTAGT ATTTAAATAA ATAAAAATAA AAAAAAAGAA AAAAGTTTTT  80
mea 92-24.ab1  TTTTAATGTA AATATGTATT TGATGTATTT AATATTTAGT ATTTAAATAA ATAAAAATAA AAAAAAAGAA AAAAGTTTTT  80
mea 92-25.ab1  TTTTAATGTA AATATGTATT TGATGTATTT AATATTTAGT ATTTAAATAA ATAAAAATAA AAAAAAAGAA AAAAGTTTTT  80

                                   100                   120                   140                   160
meaisr         TAAAATCCGA AAGTAACTAT TTTCAAAAAA TCTAAATTAT AAACTTAAAT GTTTGGAATC GCGAACGACT ATTGCTAAAT  159
mea 92-1.ab1   TAAAATTTGA AAGTAATTAT TTTTAAAAAA TTTAAATTAT AAATTTAAAT GTTTGGAATC GCGAACGATC ATTGTTAAAT  160
mea 92-2.ab1   TAAAATTTGA AAGTAATTAT TTTTAAAAAA TTTAAATTAT AAATTTAAAT GTTTGGAATC GCGAACGATT ATTGTTAAAT  160
mea 92-3.ab1   CAAAATTTGA AAGTAATTAT TTTTAAAAAA TTTAAATTAT AAATTTAAAT GTTTGGAATC GCGAACGATT ATTGTTAAAT  160
mea 92-4.ab1   TAAAATTTGA AAGTAATTAT TTTTAAAAAA TTTAAATTAT AAATTTAAAT GTTTGGAATC GCGAACGATT ATTGTTAAAT  160
mea 92-5.ab1   TAAAATTTGA AAGTAATTAT TTTTAAAAAA TTTAAATTAT AAATTTAAAT GTTTGGAATC GCGAACGATT ATTGTTAAAT  160
mea 92-6.ab1   TAAAATTTGA AAGTAATTAT TTTTAAAAAA TTTAAATTAT AAATTTAAAT GTTTGGAATC GCGAACGATT ATTGTTAAAT  160
mea 92-7.ab1   TAAAATTTGA AAGTAATTAT TTTTAAAAAA TTTAAATTAT AAATTTAAAT GTTTGGAATC GCGAACGATT ATTGTTAAAT  160
mea 92-8.ab1   TAAAATTTGA AAGTAATTAT TTTTAAAAAA TTTAAATTAT AAATTTAAAT GTTTGGAATT GCGAATGATT ATTGTTAAAT  160
mea 92-9.ab1   TAAAATTTGA AAGTAATTAT TTTTAAAAAA TTTAAATTAT AGATTTAAAT GTTTGGAATC GCGAACGATT ATTGTTAAAT  160
mea 92-10.ab1  TAAAATTTGA AAGTAATTAT TTTTAAAAAA TTTAAATTAT AAATTTAAAT GTTTGGAATC GCGAACGATT ATTGTTAAAT  160
mea 92-11.ab1  TAAAATTTGA AAGTAATTAT TTTTAAAAAA TTTAAATTAT AAATTTAAAT GTTTGGAATC GCGAACGATT ATTGTTAAAT  160
mea 92-12.ab1  TAAAATTTGA AAGTAATTAT TTTTAAAAAA TTTAAATTAT AAATTTAAAT GTTTGGAATC GCGAACGATT ATTGTTAAAT  160
mea 92-13.ab1  TAAAATTTGA AAGTAATTAT TTTTAAAAAA TTTAAATTAT AAATTTAAAT GTTTGGAATT GCGAACGATT ATTGTTAAAT  160
mea 92-14.ab1  TGAAATTTGA AAGTAATTAT TTTTAAAAAA TTTAAATTAT AAATTTAAAT GTTTGGAATC GCGAACGATT ATTGTTAAAT  160
mea 92-15.ab1  TAAAATTTGA AAGTAATTAT TTTTAAAAAA TTTAAATTAT AAATTTAAAT GTTTGGAATC GCGAACGATT ATTGTTAAAT  160
mea 92-16.ab1  TAAAATTTGA AAGTAATTAT TTTTAAAAAA TTTAAATTAT AAATTTAAAT GTTTGGAATC GCGAACGGTT ATTGTTAAAT  160
mea 92-17.ab1  TAAAATTTGA AAGTAATTAT TTTTAAAAAA TTTAAATTAT AAATTTAAAT GTTCGGAATC GCGAACGATT ATTGTTAAAT  160
mea 92-18.ab1  TAAAATTTGA AAGTAATTAT TTTTAAAAAA TTTAAATTAT AAATTTAAAT GTTTGGAATC GCGAACGATT ATTGTTAAAT  160
mea 92-19.ab1  TAAAATTTGA AAGTAATTAT TTTTAAAAAA TTTAAATTAT AAATTTAAAT GTTTGGAATC GCGAACGATT ATTGTTAAAT  160
mea 92-20.ab1  TAAAATTTGA AAGTAATTAT TTTTAAAAAA TTTAAATTAT AAATTTAAAT GTTTGGAATC GCGAACGATT ATTGTTAAAT  160
mea 92-21.ab1  TAAAATTTGA AAGTAATTAT TTTTAAAAAA TTTAAATTAT AAATTTAAAT GTTTGGAATC GTGAATGATT ATTGTTAAAT  160
mea 92-22.ab1  TAAAATTTGA AAGTAATTAT TTTTAAAAAA TTTAAATTGT AAATTTAAAT GTTTGGAATC GCGAACGATT ATTGTTAAAT  160
mea 92-23.ab1  TAAAATTTGA AAGTAATTAT TTTTAAAAAA TTTAAATTAT AAATTTAAAT GTTTGGAATC GCGAACGATT ATTGTTAAAT  160
mea 92-24.ab1  TAAAATTTGA AAGTAATTAT TTTTAAAAAA TTTAAATTAT AAATTTAAAT GTTTGGAATT GCGAACGATT ATTGTTAAAT  160
mea 92-25.ab1  TAAAATTTGA AAGTAATTAT TTTTAAAAAA TTTAAATTAT AAATTTAAAT GTTTGGAATC GCGAACGATT ATTGTTAAAT  160

                                   180                   200                   220                   240
meaisr         ATAAATGCTA AATATACATG AAGATGTGAA AAACATGTTG GATTTGTGGA ATCGTTAATG ACCACGGTTA AATGGCGGGA  239
mea 92-1.ab1   ATAAATGTTA AATATATATG AAGATGTGAA AAATATGTTG GATTTGTGGA ATCGTTAATG ATTACGGTTA AATGGCGGGA  240
mea 92-2.ab1   ATAAATGTTA AATATATATG AAGATGTGAA AAATATGTTG GATTTGTGGA ATCGTTAATG ATTACGGTTA AATGGCGGGA  240
mea 92-3.ab1   ATAAATGTTA AATATATATG AAGATGTGAA AAATATGTTG GATTTGTGGA ATCGTTAATG ATTACGGTTA AATGGCGGGA  240
mea 92-4.ab1   ATAAATGTTA AATATATATG AAGATGTGAA AAATATGTTG GATTTGTGGA ATCGTTAATG ATTACGGTTA GATGGCGGGA  240
mea 92-5.ab1   ATAAATGTTA AATATATATG AAGATGTGAA AAATATGTTG GATTTGTGGA ATTGTTAATG ATTACGGTTA AATGGCGGGA  240
mea 92-6.ab1   ATAAATGTTA AATATATATG AAGATGTGAA AAATATGTTG GATTTGTGGA ATCGTTAATG ATTACGGTTA AATGGCGGGA  240
mea 92-7.ab1   ATAAATGTTA AATATATATG AAGATGTGAA AAATATGTTG GATTTGTGGA ATCGTTAATG ATTACGGTTA AATGGCGGGA  240
mea 92-8.ab1   ATAAATGTTA AATATATATG AAGATGTGAA AAATATGTTG GATTTGTGGA ATCGTTAATG ATTACGGTTA AATGGCGGGA  240
mea 92-9.ab1   ATAAATGTTA AATATATATG AAGATGTGAA AAATATGTTG GATTTGTGGA ATCGTTAATG ATTACGGTTA AATGGCGGGA  240
mea 92-10.ab1  ATAAATGTTA AATATATATG AAGATGTGAA AAATATGTTG GATTTGTGGA ATTGTTAATG ATTATGGTTA AATGGTGGGA  240
mea 92-11.ab1  ATAAATGTTA AATATATATG AAGATGTGAA AAATATGTTG GATTTGTGGA ATTGTTAATG ATTACGGTTA AATGGCGGGA  240
mea 92-12.ab1  ATAAATGTTA AATATATATG AAGATGTGAA AAATGTGTTG GATTTGTGGA ATTGTTAATG ATTACGGTTA AATGGCGGGA  240
mea 92-13.ab1  ATAAATGTTA AATATATATG AAGATGTGAA AAATATGTTG GATTTGTGGA ATCGTTAATG ATTACGGTTA AATGGCGGGA  240
mea 92-14.ab1  ATAAATGTTA AATATATATG AAGATGTGAA AAATATGTTG GATTTGTGGA ATCGTTAATG ATTATGGTTA AATGGCGGGA  240
mea 92-15.ab1  ATAAATGTTA AATATATATG AAGATGTGAA AAATATGTTG GATTTGTGGA ATCGTTAATG ATTACGGTTA AATGGCGGGA  240
mea 92-16.ab1  ATAAATGTTA AATATATATG AAGATGTGAA AAATATGTTG GATTTGTGGA ATCGTTAATG ATTACGGTTA AATGGCGGGA  240
mea 92-17.ab1  ATAAATGTTA AATATATATG AAGATGTGAA AAATATGTTG GATTTGTGGA ATCGTTAATG ATTACGGTTA AATGGCGGGA  240
mea 92-18.ab1  ATAAATGTTA AATATATATG AAGATGTGAA AAATATGTTG GATTTGTGGA ATCGTTAATG ATTACGGTTA AATGGCGGGA  240
mea 92-19.ab1  ATAAATGTTA AATATATATG AAGATGTGAA AAATATGTTG GATTTGTGGA ATCGTTAATG ATTACGGTTA AATGGCGGGA  240
mea 92-20.ab1  ATAAATGTTA AATATATATG AAGATGTGAA AAATATGTTG GATTTGTGGA ATTGTTAATG ATTACGGTTA AATGGCGGGA  240
mea 92-21.ab1  ATAAATGTTA AATATATATG AAGATGTGAA AAATATGTTG GATTTGTGGA ATCGTTAATG ATTACGGTTA AATGGCGGGA  240
mea 92-22.ab1  ATAAATGTTA AATATATATG AAGATGTGAA AAATATGTTG GATTTGTGGA ATCGTTAATG ATTACGGTTA AATGGCGGGA  240
mea 92-23.ab1  ATAAATGTTA AATATATATG AAGATGTGAA AAATATGTTG GATTTGTGGA ATCGTTAATG ATTACGGTTA AATGGCGGGA  240
mea 92-24.ab1  ATAAATGTTA AATATATATG AAGATGTGAA AAATATGTTG GATTTGTGGA ATCGTTAATG ATTACGGTTA AATGGCGGGA  240
mea 92-25.ab1  ATAAATGTTA AATATATATG AAGATGTGAA AAATATGTTG GATTTGTGGA ATCGTTAATG ATTACGGTTA AATGGCGGGA  240
```

		260			
meaisr	TCCAAAATCC	GGTTAGATTT	CACAATGTCA	TATTACG	276
mea 92-1.ab1	TTTAAAATTT	GGTTAGATTT	TATAATGTTA	TATTATG	277
mea 92-2.ab1	TTTAAAATTC	GGTTAGATTT	TATAATGTTA	TATTATG	277
mea 92-3.ab1	TTTAAAATTC	GGTTAGATTT	TATAATGTTA	TATTACG	277
mea 92-4.ab1	TTTAAAATTC	GGTTAGATTT	TATAATGTTA	TATTACG	277
mea 92-5.ab1	TTTAAAATTC	GGTTAGATTT	TATAATGTTA	TATTACG	277
mea 92-6.ab1	TTTAAAATTT	GGTTAGATTT	TATAATGTTA	TATTACG	277
mea 92-7.ab1	TTTAAAATTC	GGTTAGATTT	TATAATGTTA	TATTACG	277
mea 92-8.ab1	TTTAAAATTC	GGTTAGATTT	TATAATGTTA	TATTACG	277
mea 92-9.ab1	TTTAAAATTC	GGTTAGATTT	TATAATGTTA	TATTACG	277
mea 92-10.ab1	TTTAAAATTT	GGTTAGATTT	TATAATGTTA	TATTATG	277
mea 92-11.ab1	TTTAAAATTC	GGTTAGATTT	TATAATGTTA	TATTACG	277
mea 92-12.ab1	TTTAAAATTC	GGTTAGATTT	TATAATGTTA	TATTACG	277
mea 92-13.ab1	TTTAAAATTC	GGTTAGATTT	TATAATGTTA	TATTACG	277
mea 92-14.ab1	TTTAAAATTC	GGTTAGATTT	TATAATGTTA	TATTACG	277
mea 92-15.ab1	TTTAAAATTT	GGTTAGATTT	TATAATGTTA	TATTACG	277
mea 92-16.ab1	TTTAAAATTC	GGTTAGATTT	TATAATGTTA	TATTACG	277
mea 92-17.ab1	TTCAAAATTC	GGTTAGATTT	TATAATGTTA	TATTACG	277
mea 92-18.ab1	TTTAAAATTC	GGTTAGATTT	TATAATGTTA	TATTACG	277
mea 92-19.ab1	TTTAAAATTC	GGTTAGATTT	TATAATGTTA	TATTACG	277
mea 92-20.ab1	TTTAAAATTC	GGTTAGATTT	TATAATGTTA	TATTACG	277
mea 92-21.ab1	TTTAAAATTC	GGTTAGATTT	TATAATGTTA	TATTACG	277
mea 92-22.ab1	TTTAAAATTC	GGTTAGATTT	TATAATGTTA	TATTACG	277
mea 92-23.ab1	TTTAAAATTC	GGTTAGATTT	TATAATGTTA	TATTACG	277
mea 92-24.ab1	TTTAAAATTC	GGTTAGATTT	TATAATGTTA	TATTACG	277
mea 92-25.ab1	TTTAAAATTC	GGTTAGATTT	TATAATGTTA	TATTACG	277

```
                                          20                    40                    60                    80
mea isr unconverted   TTTAATGTAA ATATGTATTT GATGCATCTA ACATTTAGTA TCTAAACAAA TAAAAACAAA AAAAAAGAAA AAAGCTCTTT  80
mea 492-1.ab1         TTTAATGTAA ATATGTATTT GATGTATTTA ATATTTAGTA TTTAAATAAA TAAAAATAAA AAAAAAGAAA AAAGTTTTTT  80
mea 492-2.ab1         TTTAATGTAA ATATGTATTT GATGTATTTA ATATTTAGTA TTTAAATAAA TAAAAATAAA AAAAAAGAAA AAAGTTTTTT  80
mea 492-3.ab1         TTTAATGTAA ATATGTATTT GATGTATTTA ATATTTAGTA TTTAAATAAA TAAAAATAAA AAAAAAGAAA AAAGTTTTTT  80
mea 492-4.ab1         TTTAATGTAA ATATGTATTT GATGTATTTA ATATTTAGTA TTTAAATAAA TAAAAATAAA AAAAAAGAAA AAAGTTTTTT  80
mea 492-5.ab1         TTTAATGTAA ATATGTATTT GATGTATTTA ATATTTAGTA TTTAAATAAA TAAAAATAAA AAAAAAGAAA AAAGTTTTTT  80
mea 492-6.ab1         TTTAATGTAA ATATGTATTT GATGTATTTA ATATTTAGTA TTTAAATAAA TAAAAATAAA AAAAAAGAAA AAAGTTTTTT  80
mea 492-7.ab1         TTTAATGTAA ATATGTATTT GATGTATTTA ATATTTAGTA TTTAAATAAA TAAAAATAAA AAAAAAGAAA AAAGTTTTTT  80
mea 492-8.ab1         TTTAATGTAA ATATGTATTT GATGTATTTA ATATTTAGTA TTTAAATAAA TAAAAATAAA AAAAAAGAAA AAAGTTTTTT  80
mea 492-9.ab1         TTTAATGTAA ATATGTATTT GATGTATTTA ATGTTTAGTA TTTAAATAAA TAAAAATAAA AAAAAAGAAA AAAGTTTCTT  80
mea 492-10.ab1        TTTAATGTAA ATATGTATTT GATGTATTTA ATATTTAGTA TTTAAATAAA TAAAAATAAA AAAAAAAAAA AAA-TTTTTT  79
mea 492-11.ab1        TTTAATGTAA ATATGTATTT GATGTATTTA ATATTTAGTA TTTAAATAAA TAAAAATAAA AAAAAAGAAA AAAGTTTTTT  80
mea 492-12.ab1        TTTAATGTAA ATATGTATTT GATGTATTTA ATATTTAGTA TTTAAATAAA TAAAAATAAA AAAAAAGAAA AAAGTTTTTT  80
mea 492-13.ab1        TTTAATGTAA ATATGTATTT GATGTATTTA ATATTTAGTA TTTAAATAAA TAAAAATAAA AAAAAAGAAA AAAGTTTTTT  80
mea 492-14.ab1        TTTAATGTAA ATATGTATTT GATGTATTTA ATATTTAGTA TTTAAATAAA TAAAAATAAA AAAAAAGAAA AAAGTTTTTT  80
mea 492-15.ab1        TTTAATGTAA ATATGTATTT GATGTATTTA ATATTTAGTA TTTAAATAAA TAAAAATAAA AAAAAAGAAA AAAGTTTTTT  80
mea 492-16.ab1        TTTAATGTAA ATATGTATTT GATGTATTTA ATATTTAGTA TTTAAATAAA TAAAAATAAA AAAAAAGAAA AAAGTTTTTT  80
mea 492-17.ab1        TTTAATGTAA ATATGTATTT GATGTATTTA ATATTTAGTA TTTAAATAAA TAAAAATAAA AAAAAAGAAA AAAGTTTTTT  80
mea 492-18.ab1        TTTAATGTAA ATATGTATTT GATGTATTTA ATATTTAGTA TTTAAATAAA TAAAAATAAA AAAAAAGAAA AAAGTTTTTT  80
mea 492-19.ab1        TTTAATGTAA ATATGTATTT GATGTATTTA ATATTTAGTA TTTAAATAAA TAAAAATAAA AAAAAAGAAA AAAGTTTTTT  80
mea 492-20.ab1        TTTAATGTAA ATATGTATTT GATGTATTTA ATATTTAGTA TTTAAATAAA TAAAAATAAA AAAAAAGAAA AAAGTTTTTT  80
mea 492-21.ab1        TTTAATGTAA ATATGTATTT GATGTATTTA ATATTTAGTA TTTAAATAAA TAAAAATAAA AAAAAAGAAA AAAGTTTTTT  80
mea 492-22.ab1        TTTAATGTAA ATATGTATTT GATGTATTTA ATATTTAGTA TTTAAATAAA TAAAAATAAA AAAAAAGAAA AAAGTTTTTT  80
mea 492-23.ab1        TTTAATGTAA ATATGTATTT GATGTATTTA ATATTTAGTA TTTAAATAAA TAAAAATAAA AAAAAAGAAA AAAGTTTTTT  80
mea 492-24.ab1        TTTAATGTAA ATATGTATTT GGTGTATTTA ATATTTAGTA TTTAAATAAA TAAAAATAAA GAAAAAGAAA AAAGTTTTTT  80
```

```
                                         100                   120                   140                   160
mea isr unconverted   AAAATCCGAA AGTAACTATT TTCAAAAAAT CTAAATTATA AACTTAAATG TTTGGAATCG CGAACGACTA TTGCTAAATA  160
mea 492-1.ab1         AAAATTTGAA AGTAATTATT TTTAAAAAAT TTAAATTATA AATTTAAATG TTTGGAATCG CGAACGATTA TTGTTAAATA  160
mea 492-2.ab1         AAAATTTGAA AGTAATTATT TTTAAAAAAT TTAAATTATA AATTTAAATG TTTGGAATTG TGAACGATTA TTGTTAAATA  160
mea 492-3.ab1         AAAATTTGAA AGTAATTATT TTTAAAAAAT TTAAATTATA AATTTAAATG TTTGGAATCG CGAACGATTA TTGTTAAATA  160
mea 492-4.ab1         AAAATTTGAA AGTAATTATT TTTAAAAAAT TTGAATTATA GATTTAAATG TTTGGAATCG CGAATGATTA TTGTTAAATA  160
mea 492-5.ab1         AAAATTTGAA AGTAATTATT TTTAAAAAAT TTAAATTATA AATTTAAATG TTTGGAATTG CGAACGATTA TTGTTAAATA  160
mea 492-6.ab1         AAAATTTGAA AGTAATTATT TTTAAAAAAT TTAAATTATA AATTTAAATG TTTGGAATCG CGAACGATTA TTGTTAAATA  160
mea 492-7.ab1         AAAATTTGAA AGTAATTATT TTTAAAAAAT TTAAATTATA AATTTAAATG TTTGGAATCG CGAACGATTA TTGTTAAATA  160
mea 492-8.ab1         AAAATTTGAA AGTAATTATT TTTAAAAAAT TTAAATTATA AATTTAAATG TTTGGAATTG CGAACGATTA TTGTTAAATA  160
mea 492-9.ab1         AAAATTTGAA AGTAATTATT TTTAAAAAAT TTAAATTATA AATTTAAATG TTTGGAATCG CGAACGATTA TTGTTAAATA  160
mea 492-10.ab1        AAAATTTGAA AGTAATTATT TTTAAAAAAT TTTAATTATA AATTTAAATG GTTGGAATCC CGAATGAATA TTGGTAAATA  159
mea 492-11.ab1        AAAATTTGAA AGTAATTATT TTTAAAAAAT TTAAATTATA AATTTAAATG TTTGGAATTG CGAATGATTA TTGTTAAATA  160
mea 492-12.ab1        AAAATTTGAA AGTAATTATT TT-AAAAAAT TTAAATTATA AATTTAAATG TTTGGAATCG CGAACGATTA TTGTTAAATA  159
mea 492-13.ab1        AAAATTTGAA AGTAATTATT TTTAAAAAAT TTAAATTATA AATTTAAATG TTTGGAATCG CGAATGATTA TTGTTAAATA  160
mea 492-14.ab1        AAAATTTGAA AGTAATTATT TTTAAAAAAT TTAAATTATA AATTTAAATG TTTGGAATCG CGAATGATTA TTGTTAAATA  160
mea 492-15.ab1        AAGATTTGAA AGTAATTATT TTTAAAAAAT TTAAATTATA AATTTAAATG TTTGGAATTG TGAATGATTA TTGTTAAATA  160
mea 492-16.ab1        AAAATTTGAA AGTAATTATT TTTAAAAAAT TTAAATTATA AATTTAAATG TTTGGAATTG TGAATGATTA TTGTTAAATA  160
mea 492-17.ab1        AAAATTTGAA AGTAATTATT TTTAAAAAAT TTAAATTATA AATTTAAATG TTTGGAATCG CGAACGATTA TTGTTAAATA  160
mea 492-18.ab1        AAGATTTGAA AGTAATTATT TTTAAAAAAT TTAAATTATA AATTTAAATG TTTGGAATCG CGAATGATTA TTGTTAAATA  160
mea 492-19.ab1        AAAATTTGAA AGTAATTATT TTTAAAAAAT TTAAATTATA AATTTAAATG TTTGGAATTG TGAATGATTA TTGTTAAATA  160
mea 492-20.ab1        ATAATTTGAA AGTAATTATT TTTAAAAAAT TTAAATTATA AATTTAAATG TTTGGAATCG CGAACGATTA TTGTTAAATA  160
mea 492-21.ab1        AAAATTTGAA AGTAATTATT TTTAAAAAAT TTAAATTATA AATTTAAATG TTTGGAATTG TGAATGATTA TTGTTAAATA  160
mea 492-22.ab1        AAAATTTGAA AGTAATTATT TTTAAAAAAT TTAAATTATA AATTTAAATG TTTGGAATCG CGAATGATTA TTGTTAAATA  160
mea 492-23.ab1        AAAATTTGAA AGTAATTATT TTTAAAAAAT TTAAATTATA AATTTAAATG TTTGGAATTG TGAATGATTA TTGTTAAATA  160
mea 492-24.ab1        AAAATTTGAA AGTAATTATT TTTAAAAAAT TTAAATTATA AATTTAAATG TTTGGAATCG CGAATGATTA TTGTTAAATA  160
```

```
                                         180                   200                   220                   240
mea isr unconverted   TAAATGCTAA ATATACATGA AGATGTGAAA AACATGTTGG ATTTGTGGAA TCGTTAATGA CCACGGTTAA ATGGCGGGAT  240
mea 492-1.ab1         TAAATGTTAA ATATATATGA AGATGTGAAA AATATGTTGG ATTTGTGGAA TCGTTAATGA TTACGGTTAA ATGGCGGGAT  240
mea 492-2.ab1         TAAATGTTAA ATATATATGA AGATGTGAAA AATATGTTGG ATTTGTGGAA TCGTTAATGA TTACGGTTAA ATGGCGGGAT  240
mea 492-3.ab1         TAAATGTTAA ATATATATGA AGATGTGAAA AATATGTTGG ATTTGTGGAA TCGTTAATGA TTACGGTTAA ATGGCGGGAT  240
mea 492-4.ab1         TAAATGTTAA ATATATATGA AGATGTGAAA AATATGTTGG ATTTGTGGAA TTGTTAATGA TTACGGTTAA ATGGTGGGAT  240
mea 492-5.ab1         TAAATGTTAA ATATATATGA AGATGTGAAA AATATGTTGG ATTTGTGGAA TCGTTAATGA TTACGGTTAA ATGGCGGGAT  240
mea 492-6.ab1         TAAATGTTAA ATATATATGA AGATGTGAAA AATATGTTGG ATTTGTGGAA TCGTTAATGA TTACGGTTAA ATGGCGGGAT  240
mea 492-7.ab1         TAAATGTTAA ATATATATGA AGATGTGAAA AATATGTTGG ATTTGTGGAA TCGTTAATGA TTACGGTTAA ATGGCGGGAT  240
mea 492-8.ab1         TAAATGTTAA ATATATATGA AGATGTGAAA AATATGTTGG ATTTGTGGAA TCGTTAATGA TTACGGTTAA ATGGCGGGAT  240
mea 492-9.ab1         TAAATGTTAA ATATATGTGA AGATGTGAAA AATATGTTGG ATTTGTGGAA TCGTTAATGA TTACGGTTAA ATGGTGGGAT  240
mea 492-10.ab1        TAAATGGTAA ATATATATGA AGAAGTGAAA AATATGTTGG AATTGTGGAA TCTGTAATGA TTACGGGTAA ATGGCGGGAT  239
mea 492-11.ab1        TAAATGTTAA ATATATATGA AGATGTGAAA AATATGTTGG ATTTGTGGAA TCGTTAATGA TTACGGTTAA ATGGCGGGAT  240
mea 492-12.ab1        TAAATGTTAA ATATATATGA AGATGTGAAA AATATGTTGG ATTTGTGGAA TCGTTAATGA TTACGGTTAA ATGGCGGGAT  239
mea 492-13.ab1        TAAATGTTAA ATATATATGA AGATGTGAAA AATATGTTGG ATTTGTGGAA TCGTTAATGA TTACGGTTAA ATGGCGGGAT  240
mea 492-14.ab1        TAAATGTTAA ATATATATGA AGATGTGAAA AATATGTTGG ATTTGTGGAA TCGTTAATGA TTACGGTTAA ATGGCGGGAT  240
mea 492-15.ab1        TAAATGTTAA ATATATATGA AGATGTGAAA AATATGTTGG ATTTGTGGAA TCGTTAATGA TTACGGTTAA ATGGCGGGAT  240
mea 492-16.ab1        TAAATGTTAA ATATATATGA AGATGTGAAA AATATGTTGG ATTTGTGGAA TCGTTAATGA TTACGGTTAA ATGGCGGGAT  240
mea 492-17.ab1        TAAATGTTAA ATATATATGA AGATGTGAAA AATATGTTGG ATTTGTGGAA TCGTTAATGA TTACGGTTAA ATGGCGGGAT  240
mea 492-18.ab1        TAAATGTTAA ATATATATGA AGATGTGAAA AATATGTTGG ATTTGTGGAA TCGTTAATGA TTACGGTTAA ATGGCGGGAT  240
mea 492-19.ab1        TAAATGTTAA ATATATATGA AGATGTGAAA AATATGTTGG ATTTGTGGAA TCGTTAATGA TTACGGTTAA ATGGCGGGAT  240
mea 492-20.ab1        TAAATGTTAA ATATATATGA AGATGTGAAA AATATGTTGG ATTTGTGGAA TCGTTAATGA TTACGGTTAA ATGGCGGGAT  240
mea 492-21.ab1        TAAATGTTAA ATATATATGA AGATGTGAAA AATATGTTGG ATTTGTGGAA TCGTTAATGA TTACGGTTAA ATGGCGGGAT  240
mea 492-22.ab1        TAAATGTTAA ATATATATGA AGATGTGAAA AATATGTTGG ATTTGTGGAA TCGTTAATGA TTACGGTTAA ATGGCGGGAT  240
mea 492-23.ab1        TAAATGTTAA ATATATATGA AGATGTGAAA AATATGTTGG ATTTGTGGAA TCGTTAATGA TTACGGTTAA ATGGCGGGAT  240
mea 492-24.ab1        TAAATGTTAA ATATATATGA AGATGTGAAA AATATGTTGG ATTTGTGGAA TCGTTAATGA TTACGGTTAA ATGGCGGGAT  240
```

```
                                                260
                                                  |
mea isr unconverted  CCAAAATCCG GTTAGATTTC ACAATGTCAT ATTACG 276
    mea 492-1.ab1    TTAAAATTCG GTTAGATTTT ATAATGTTAT ATTACG 276
    mea 492-2.ab1    TTAAAATTCG GTTAGATTTT ATAATGTTAT ATTATG 276
    mea 492-3.ab1    TTAAAATTCG GTTAGATTTT ATAATGTTAT ATTACG 276
    mea 492-4.ab1    TTAAAATTTG GTTAGATTTT ATAATGTTAT ATTACG 276
    mea 492-5.ab1    TTAAAATCCG GTTAGATTTT ATAATGTTAT GTTATG 276
    mea 492-6.ab1    TTAAAATTCG GTTAGATTTT ATAATGTTAT ATTACG 276
    mea 492-7.ab1    TTAAAATTCG GTTAGATTTT ATAATGTTAT ATTACG 276
    mea 492-8.ab1    TTAAAATTCG GTTAGATTTT ATAATGTTAT ATTACG 276
    mea 492-9.ab1    TTAAAATTCG GTTAGATTTT ATAATGTTAT ATTACG 276
   mea 492-10.ab1    TTAAAATTTG GTTAGATTTT ATAATGGTAT ATTACC 275
   mea 492-11.ab1    TTAAAATTCG GTTAGATTTT ATAATGTTAT ATTACG 276
   mea 492-12.ab1    TTAAAATTCG GTTAGATTTT ATAATGTTAT ATTACG 275
   mea 492-13.ab1    TTAAAATTCG GTTAGATTTT ATAATGTTAT ATTACG 276
   mea 492-14.ab1    TTAAAATTCG GTTAGATTTT ATAATGTTAT ATTACG 276
   mea 492-15.ab1    TTAAAATTCG GTTAGATTTT ATAATGTTAT ATTACG 276
   mea 492-16.ab1    TTAAAATTCG GTTAGATTTT ATAATGTTAT ATTACG 276
   mea 492-17.ab1    TTAAAATTCG GTTAGATTTT ATAATGTTAT ATTACG 276
   mea 492-18.ab1    TTAAAATTTG GTTAGATTTT ATAATGTTAT ATTACG 276
   mea 492-19.ab1    TTAAAATTCG GTTAGATTTT ATAATGTTAT ATTACG 276
   mea 492-20.ab1    TTAAAATTCG GTTAGATTTT ATAATGTTAT ATTACG 276
   mea 492-21.ab1    TTAAAATTCG GTTAGATTTT ATAATGTTAT ATTACG 276
   mea 492-22.ab1    TTAAAATTCG GTTAGATTTT ATAATGTTAT ATTACG 276
   mea 492-23.ab1    TTAAAATTCG GTTAGATTTT ATAATGTTAT ATTACG 276
   mea 492-24.ab1    TTAAAATTCG GTTAGATTTT ATAATGTTAT ATTACG 276
```

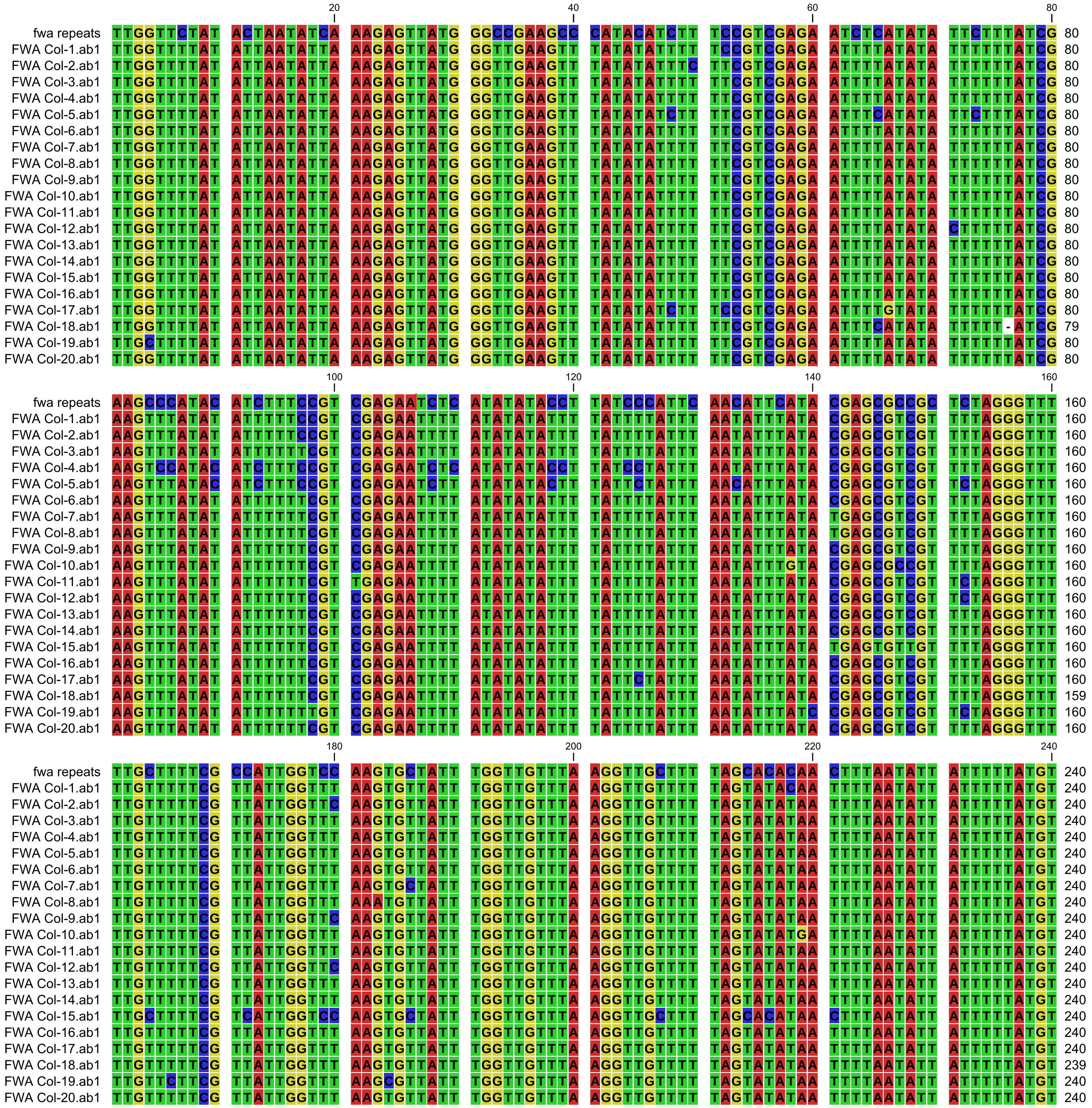
fwa repeats
FWA Col-1.ab1
FWA Col-2.ab1
FWA Col-3.ab1
FWA Col-4.ab1
FWA Col-5.ab1
FWA Col-6.ab1
FWA Col-7.ab1
FWA Col-8.ab1
FWA Col-9.ab1
FWA Col-10.ab1
FWA Col-11.ab1
FWA Col-12.ab1
FWA Col-13.ab1
FWA Col-14.ab1
FWA Col-15.ab1
FWA Col-16.ab1
FWA Col-17.ab1
FWA Col-18.ab1
FWA Col-19.ab1
FWA Col-20.ab1
20
40
60
80
100
120
140
160
180
200
220
240

```
                                                              260                 280                 300                 320
                                                                |                   |                   |                   |
fwa repeats      TTTTTTTCTT ACGATTTATC GATTTGTGGG ATACTGACAA TCAGATTATT GTTGTTTTTT CCAGCCAAAT ATCAGATCTT 320
FWA Col-1.ab1    TTTTTTT--- ACGATTTATC GATTTGTGGG ATATTGATAA TTAGATTATT GTTGTTTTTT TCAGTTAAAT ATTAGATTTT 317
FWA Col-2.ab1    TTTTTTTTTT ACGATTTATC GATTTGTGGG ATATTGATAA TTAGATTATT GTTGTTTTTT TTAGTTAAAT ATTAGATTTT 320
FWA Col-3.ab1    TTTTTTTT-- ACGGTTTATC GATTTGTGGG ATATTGATAA TTAGATTATT GTTGTTTTTT TTAGTTAAAT ATTAGATTTT 318
FWA Col-4.ab1    TTTTTTTTTT ACGATTTATC GATTTGTGGG ATATTGATAA TTAGATTATT GTTGTTTTTT TTAGTTAAAT ATTAGATTTT 320
FWA Col-5.ab1    TTTTTTTTTT ACGATTTATT GATTTGTGGG ATATTGATAA TTAGATTATT GTTGTTTTTT TTAGTTAAAT ATTAGATTTT 320
FWA Col-6.ab1    TTTTTTTTTT ACGATTTATC GATTTGTGGG ATACTGATAA TTAGATTATT GTTGTTTTTT TTAGTTAAAT ATTAGATTTT 320
FWA Col-7.ab1    TTTTTTTTTT ACGATTTATT GATTTGTGGG ATATTGATAA TTAGATTATT GTTGTTTTTT TTAGTTAAAT ATTAGATTTT 320
FWA Col-8.ab1    TTTTTTTTTT ACGATTTATC GATTTGTGGG ATATTGATAA TTAGATTATT GTTGTTTTTT TTAGTTAAAT ATTAGATTTT 320
FWA Col-9.ab1    TTTTTTTTTT ATGATTTATT GATTTGTGGG ATATTGATAA TTAGATTATT GTTGTTTTTT TTAGTTAAAT ATTAGATTTT 320
FWA Col-10.ab1   TTTTTTTTTT ACGATTTATC GATTTGTGGG ATATTGATAA TTAGATTATT GTTGTTTTTT TTAGTTAAAT ATTAGATTTT 320
FWA Col-11.ab1   TTTTTTTTTT ACGATTTATC GATTTGTGGG ATATTGATAA TTAGATTATT GTTGTTTTTT TTAGTTAAAT ATTAGATTTT 320
FWA Col-12.ab1   TTTTTTTTTT ACGATTTATC GATTTGTGGG ATATTGATAA TTAGATTATT GTTGTTTTTT TTAGTTAAAT ATTAGATATT 320
FWA Col-13.ab1   TTTTTTTTTT ACGATTTATC GATTTGTGGG ATACTGATAA TTAGATTATT GTTGTTTTTT TTAGTTAAAT ATTAGATTTT 320
FWA Col-14.ab1   TTTTTTTTT- ACGATTTATC GATTTGTGGG ATATTGATAA TTAGATTATT GTTGTTTTTT TTAGTTAAAT ATTAGATTTT 319
FWA Col-15.ab1   TTTTTTTTTT ACGATTTATT GATTTGTGGG ATATTGATAA TTAGATTATT GTTGTTTTTT TTAGTTAAAT ATTAGATTTT 320
FWA Col-16.ab1   TTTTTTTTTT ATGATTTATC GATTTGTGGG ATATTGATAA TTAGATTATT GTTGTTTTTT TTAGTTAAAT ATCAGATTTT 320
FWA Col-17.ab1   TTTTTTTTTT ACGATTTATC GATTTGTGGG ATATTGATAA TTAGATTATT GTTGTTTTTT TTAGTTAAAT ATTAGATTTT 320
FWA Col-18.ab1   TTTTTTTTTT ACGATTTATC GATTTGTGGG ATATTGATAA TTAGATTATT GTTGTTTTTT TTAGTTAAAT ATTAGATTTT 319
FWA Col-19.ab1   TTTTTTTTT- ACGATTTATC GATTTGTAGG ATATTGATAA TTTAGATTTT GTTGTTTTTT TTAGTCAAAT ATTAGATTGT 319
FWA Col-20.ab1   TTTTTTTTTT ACGATTTATC GATTTGTGGG ATACTGATAA TTAGATTATT GTTGTTTTTT TTAGTTAAAT ATTAGATTTT 320

                                                              340                 360                 380                 400
                                                                |                   |                   |                   |
fwa repeats      GCGCCGCTCT TTATCCCATT CAACATTCAT ACGAGCACCG CTTTACGGTT TTTGCTTTTC GACATTGGTC GAAGTGCTAT 400
FWA Col-1.ab1    GCGTCGTTTT TTATTCTATT TAATATTTAT ATGAGTATCG TTTTACGGTT TTTGTTTTTC GATATTGGTC GAAGTGTTAT 397
FWA Col-2.ab1    GCGTCGTTTT TTATTTTATT TAATATTTAT ACGAGTATCG TTTTACGGTT TTTGTTTTTC GATATTGGTC GAAGTGTTAT 400
FWA Col-3.ab1    GCGTCGTTTT TTATTTTATT TAATATTTAT ACGAGTATCG TTTTACGGTT TTTGTTTTTC GATACTGGTC GAAGTGTTAT 398
FWA Col-4.ab1    GTGTTGTTTT TTATTTTATT TAATATTTAT ATGAGTATTG TTTTATGGTT TTTGTTTTTC GATATTGGTC GAAGTGTTAT 400
FWA Col-5.ab1    GCGTCGTTTT TTATTTTATT TAATATTTAT ACGAGTATCG TTTTACGGTT TTTGTTTTTC GATATTGGTC GAAGTGCTAT 400
FWA Col-6.ab1    GCGTCGTTTT TTATTTTATT TAATATTTAT ACGAGTATCG CTTTACGGTT TTTGTTTTTC GATATTGGTC GAAGTGTTAT 400
FWA Col-7.ab1    GCGTCGTTTT TTATTTTATT TAATATTTAT ACGAGTATCG TTTTACGGTT TTTGTTTTTC GACATCGGTC GAAGTGTTAT 400
FWA Col-8.ab1    GCGTCGTTTT TTATTTTATT TAATATTTAT ACGAGTATCG TTTTACGGTT TTTGTTTTTC GATATTGGTC GAAGTGTTAT 400
FWA Col-9.ab1    GCGTTGTTTT TTATTTTATT TAATATTTAT ACGAGTATCG TTTTACGGTT TTTGTTTTTC GATATTGGTC GAAGTGTTAT 400
FWA Col-10.ab1   GCGTTGTTTT TTATTTTATT TAATATTTAT ACGAGTATCG TTTTACGGTT TTTGTTTTTC GATATTGGTC GAAGTGTTAT 400
FWA Col-11.ab1   GCGTCGTTTT TTATTTTATT TAATATTTAT ACGAGTATCG TTTTACGGTT TTTGTTTTTC GATATTGGTC GAAGTGTTAT 400
FWA Col-12.ab1   GCGTCGTTTT TTATTTTATT TAATATTTAT ACGAGTATCG TTTTACGGTT TTTGTTTTTC GATATTGGTC GAAGTGTTAT 400
FWA Col-13.ab1   GCGTCGTTTT TTATTTTATT TAATATTTAT ACGAGTATCG CTTTACGGTT TTTGTTTTTT GATATTGGTC GAAGTGTTAT 400
FWA Col-14.ab1   GCGTCGTTTT TTATTTTATT TAATATTTAT ACGAGTATCG TTTTACGGTT TTTGTTTTTC GATATTGGTC GAAGTGTTAT 399
FWA Col-15.ab1   GCGTTGTTTT TTATTTTATT TAATATTTAT ACGAGTATCG TTTTACGGTT TTTGTTTTTC GATATTGGTC GAAGTGTTAT 400
FWA Col-16.ab1   GCGTCGTTTT TTATTTTATT TAATATTTAT ACGAGTATCG TTTTACGGTT TTTGTTTTTC GATATTGGTC GAAGTGTTAT 400
FWA Col-17.ab1   GCGTCGTTTT TTATTTTATT TAATATTTAT ACGAGTATCG TTTTACGGTT TTTGTTTTTC GATATTGGTC GAAGTGTTAT 400
FWA Col-18.ab1   GCGTCGTTTT TTATTTTATT TAATATTTAT ATGAGTATCG TTTTACGGTT TTTGTTTTTC GATATTGGTC GAAGTGTTAT 399
FWA Col-19.ab1   GCGTCGTTTT TCATTTTATT TAATATTTAT ACGAGTACCG TTTTACGGTT TTTGTTTTTC GATATTGGTC GAAGTGTTAC 399
FWA Col-20.ab1   GCGTCGTTTT TTATTTTATT TAATATTTAT ACGAGTATCG TTTTACGGTT TTTGTTTTTC GATATTGGTC GAAGTGTTAT 400

                                                              420                 440                 460                 480
                                                                |                   |                   |                   |
fwa repeats      TTGGTTGTTT AAGGTTGCTT TTAGCACACA ACTTTAATAT TATTTTTATG TTTTCTTCTT ACGATTTATC GATTTGTAGG 480
FWA Col-1.ab1    TTGGTTGTTT AAGGTTGCTT TTAGCATACA ACTTTAATAT TATTTTTATG TTTTTTTTTT ACGATTTATC GATTTGTAGG 477
FWA Col-2.ab1    TTGGTTGTTT AAGGTTGTTT TTAGTATATA ATTTTAATAT TATTTTTATG TTTTTTTTTT ATGATTTATC GATTTGTAGG 480
FWA Col-3.ab1    TTGGTTGTTT AAGGTTGTTT TTAGTATATA ATTTTAATAT TATTTTTATG TTTTTTTTTT ACGATTTATC GATTTGTAGG 478
FWA Col-4.ab1    TTGGTTGTTT AAGGTTGTTT TTAGTATATA ATTTTAATAT TATTTTTATG TTTTTTTTTT ACGATTTATC GATTTGTAGG 480
FWA Col-5.ab1    TTGGTTGTTT AAGGTTGCTT TTAGCACACG ACTTTAATAT TATTTTTATG TTTTTTTTTT ACGATTTATC GATTTGTAGG 480
FWA Col-6.ab1    TTGGTTGTTT AAGGTTGTTT TTAGTATATA ATTTTAATAT TATTTTTATG TTTTTTTTT- ACGATTTATC GATTTGTAGG 479
FWA Col-7.ab1    TTGGTTGTTT AAGGTTGTTT TTAGTATATA ATTTTAATAT TATTTTTATG TTTTTTTTTT ACGATTTATT GATTTGTAGG 480
FWA Col-8.ab1    TTGGTTGTTT AAGGTTGTTT TTAGTATATA ATTTTAATAT TATTTTTATG TTTTTTTTTT ACGATTTATC GATTTGTAGG 480
FWA Col-9.ab1    TTGGTTGTTT AAGGTTGTTT TTAGTATATA ATTTTAATAT TATTTTTATG TTTTTTTTTT ACGATTTATC GATTTGTAGG 480
FWA Col-10.ab1   TTGGTTGTTT AAGGTTGTTT TTAGTATATA ATTTTAATAT TATTTTTATG TTTTTTTTTT ACGATTTATC GATTTGTAGG 480
FWA Col-11.ab1   TTGGTTGTTT AAGGTTGTTT TTAGTATATA ATTTTAATAT TATTTTTATG TTTTTTTTTT ACGATTTATC GATTTGTAGG 480
FWA Col-12.ab1   TTGGTTGTTT AAGGTTGTTT TTAGTATATA ATTTTAATAT TTTTTTTATG TTTTTTTTT- ATGATTTATC GATTTGTAGG 479
FWA Col-13.ab1   TTGGTTGTTT AAGGTTGTTT TTAGTATATA ATTTTAATAT TATTTTTATG TTTTTTTTT- ACGATTTATC GATTTGTAGG 479
FWA Col-14.ab1   TTGGTTGTTT AAGGTTGTTT TTAGTATATA ATTTTAATAT TATTTTTATG TTTTTTTTTT ACGATTTATC GATTTGTAGG 479
FWA Col-15.ab1   TTGGTTGTTT AAGGTTGTTT TTAGTATATA ATTTTAATAT TATTTTTATG TTTTTTTTTT ACGATTTATT GATTTGTAGG 480
FWA Col-16.ab1   TTGGTTGTTT AAGGTTGTTT TTAGTATATA ATTTTAATAT TATTTTTATG TTTTTTTTT- ACGATTTATC GATTTGTAGG 479
FWA Col-17.ab1   TTGGTTGCTT AAGGTTGTTT TTAGTATATA ATTTTAATAT TATTTTTATG TTTTTTTTTT ACGATTTATC GATTTGTAGG 480
FWA Col-18.ab1   TTGGTTGTTT AAGGTTGTTT TTAGTATATA ATTTTAATAT TATTTTTATG TTTTTTTTTT ACGATTTATC GATTTGTAGG 479
FWA Col-19.ab1   TTGGTTGTTT AAGTTTGTTT TTAGTATATA ATTTTAATAT TATTTTTATG TTTTTTTTT- ATGATTTATC GATTTGTAGG 478
FWA Col-20.ab1   TTGGTTGTTT AAGGTTGTTT TTAGTATATA ATTTTAATAT TATTTTTATG TTTTTTTTT- ACGATTTATC GATTTGTAGG 479
```

```
                                             500                        520                        540                        560
                                              |                          |                          |                          |
fwa repeats       ATACTGACAA  TCAGATTTTT  GTTGTTTTTT  TCAGCCAAAA  ATCAGATTTT  TTTACACTTT  GTTTAGAGAA  TGATTTTGGT  560
FWA Col-1.ab1     ATATTGATAA  TTAGATTTTT  GTTGTTTTTT  TTAGTTGAAA  ATTAGATTTT  TTTATATTTT  GTTTAGAGAA  TGATTTTGGT  557
FWA Col-2.ab1     ATATTGATAA  TTAGATTTTT  GTTGTTTTTT  TTAGTTAAAA  ATTAGATTTT  TTTATATTTT  GTTTAGAGAA  TGATTTTGGT  560
FWA Col-3.ab1     ATATTGATAA  TTAGATTTTT  GTTGTTTTTT  TTAGTTAAAA  ATTAGATTTT  TTTATATTTT  GTTTAGAGAA  TGATTTTGGT  558
FWA Col-4.ab1     ATATTGATAA  TTAGATTTTT  GTTGTTTTTT  TTAGTTAAAA  ATTAGATTTT  TTTATATTTT  GTTTAGAGAA  TGATTTTGGT  560
FWA Col-5.ab1     ATACTGATAA  TTAGATTTTT  GTTGTTTTTT  TTAGTTAAAA  ATTAGATTTT  TTTATATTTT  GTTTAGAGAA  TGATTTTGGT  560
FWA Col-6.ab1     ATACTGATAA  TTAGATTTTT  GTTGTTTTTT  TTAGTTAAAA  ATTAGATTTT  TTTATATTTT  GTTTAGAGAA  TGATTTTGGT  559
FWA Col-7.ab1     ATATTGATAA  TTAGATTTTT  GTTGTTTTTT  T-AGTTAAAA  ATTAGATTTT  TTTATATTTT  GTTTAGAGAA  TGATTTTGGT  559
FWA Col-8.ab1     ATATTGATAA  TTAGATTTTT  GTTGTTTTTT  TTAGTTAAAA  ATTAGATTTT  TTTATATTTT  GTTTAGAGAA  TGATTTTGGT  560
FWA Col-9.ab1     ATATTGATAA  TTAGATTTTT  GTTGTTTTTT  TTAGTTAAAA  ATTAGATTTT  TTTATATTTT  GTTTAGAGAA  TGATTTTGGT  560
FWA Col-10.ab1    ATACTGATAA  TTAGATTTTT  GTTGTTTTTT  TTAGTTAAAA  ATTAGATTTT  TTTATATTTT  GTTTAGAGAA  TGATTTTGGT  560
FWA Col-11.ab1    ATATTGATAA  TTAGATTTTT  GTTGTTTTTT  TTAGTTAAAA  ATTAGATTTT  TTTATATTTT  GTTTAGAGAA  TGATTTTGGT  560
FWA Col-12.ab1    ATATTGATAA  TTAGATTTTT  GTTGTTTTTT  TTAGTTAAAA  ATTAGATTTT  TTTATATTTT  GTTTAGAGAA  TGATTTTGGT  559
FWA Col-13.ab1    ATATTGATAA  TTAGATTTTT  GTTGTTTTTT  TTAGTTAAAA  ATTAGATTTT  TTTATATTTT  GTTTAGAGAA  TGATTTTGGT  559
FWA Col-14.ab1    ATATTGATAA  TTAGATTTTT  GTTGTTTTTT  TTAGTTAAAA  ATTAGATTTT  TTTATATTTT  GTTTAGAGAA  TGATTTTGGT  559
FWA Col-15.ab1    ATATTGATAA  TTAGATTTTT  GTTGTTTTTT  TTAGTTAAAA  ATTAGATTTT  TTTATATTTT  GTTTAGAGAA  TGATTTTGGT  560
FWA Col-16.ab1    ATATTGATAA  TTAGATTTTT  GTTGTTTTTT  TTAGTTAAAA  ATTAGATTTT  TTTATATTTT  GTTTAGAGAA  TGATTTTGGT  559
FWA Col-17.ab1    ATATTGATAA  TTAGATTTTT  GTTGTTTTTT  TTAGTTAAAA  ATTAGATTTT  TTTATATTTT  GTTTAGAGAA  TGATTTTGGT  560
FWA Col-18.ab1    ATACTGATAA  TTAGATTTTT  GTTGTTTTTT  TTAGTTAAAA  ATTAGATTTT  TTTATATTTT  GTTTAGAGAA  TGATTTTGGT  559
FWA Col-19.ab1    ATATTGATAA  TTAGATTTTT  GTTGTTTTTT  TTAGTTAAAA  ATTAGATTTT  TTTATATTTT  GTTTAGAGAA  TGATTTTGGT  558
FWA Col-20.ab1    ATATTGATAA  TTAGATTTTT  GTTGTTTTTT  TTAGTTAAAA  ATTAGATTTT  TTTATATTTT  GTTTAGAGAA  TGATTTTGGT  559

fwa repeats       TAAGGGCGAT  TTG  573
FWA Col-1.ab1     TAAGGGCGAA  TTC  570
FWA Col-2.ab1     TAAGGGCGAA  TTC  573
FWA Col-3.ab1     TAAGGGCGAA  TTC  571
FWA Col-4.ab1     TAAGGGCGAA  TTC  573
FWA Col-5.ab1     TAAGGGCGAA  TTC  573
FWA Col-6.ab1     TAAGGGCGAA  TTC  572
FWA Col-7.ab1     TAAGGGCGAA  TTC  572
FWA Col-8.ab1     TAAGGGCGAA  TTC  573
FWA Col-9.ab1     TAAGGGCGAA  TTC  573
FWA Col-10.ab1    TAAGGGCGAA  TTC  573
FWA Col-11.ab1    TAAGGGCGAA  TTC  573
FWA Col-12.ab1    TAAGGGCGAA  TTC  572
FWA Col-13.ab1    TAAGGGCGAA  TTC  572
FWA Col-14.ab1    TAAGGGCGAA  TTC  572
FWA Col-15.ab1    TAAGGGCGAA  TTC  573
FWA Col-16.ab1    TAAGGGCGAA  TTC  572
FWA Col-17.ab1    TAAGGGCGAA  TTC  573
FWA Col-18.ab1    TAAGGGCGAA  TTC  572
FWA Col-19.ab1    TAAGGGCGAA  TTC  571
FWA Col-20.ab1    TAAGGGCGAA  TTC  572
```

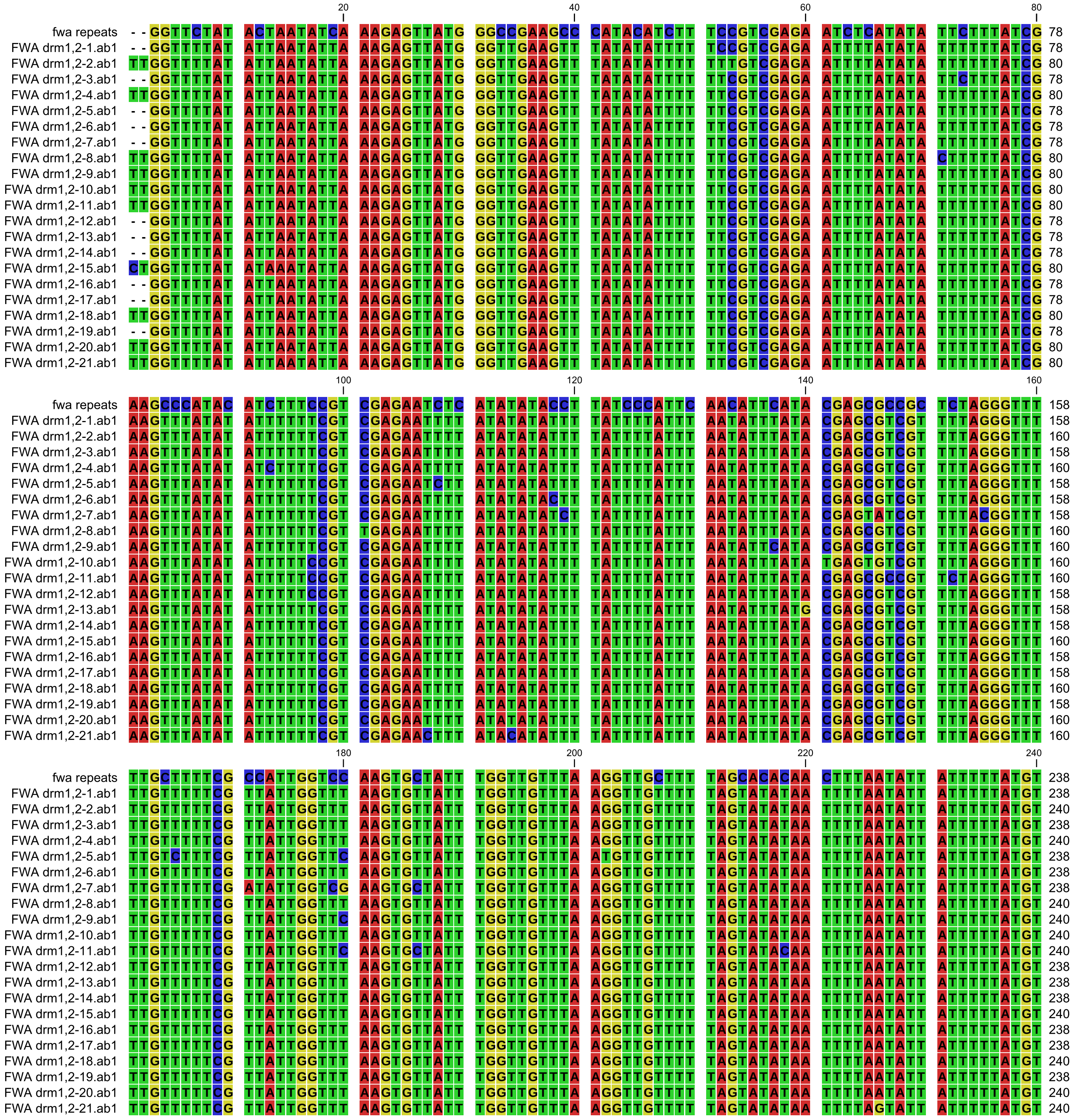
20 40 60 80
fwa repeats --GGTTCTAT ACTAATATCA AAGAGTTATG GGCCGAAGCC CATACATCTT TCCGTCGAGA ATCTCATATA TTCTTTATCG 78
FWA drm1,2-1.ab1 --GGTTTTAT ATTAATATTA AAGAGTTATG GGTTGAAGTT TATATATTTT TCCGTCGAGA ATTTTATATA TTTTTTATCG 78
FWA drm1,2-2.ab1 TTGGTTTTAT ATTAATATTA AAGAGTTATG GGTTGAAGTT TATATATTTT TTTGTCGAGA ATTTTATATA TTTTTTATCG 80
FWA drm1,2-3.ab1 --GGTTTTAT ATTAATATTA AAGAGTTATG GGTTGAAGTT TATATATTTT TTCGTCGAGA ATTTTATATA TTCTTTATCG 78
FWA drm1,2-4.ab1 TTGGTTTTAT ATTAATATTA AAGAGTTATG GGTTGAAGTT TATATATTTT TTCGTCGAGA ATTTTATATA TTTTTTATCG 80
FWA drm1,2-5.ab1 --GGTTTTAT ATTAATATTA AAGAGTTATG GGTTGAAGTT TATATATTTT TTCGTCGAGA ATTTTATATA TTTTTTATCG 78
FWA drm1,2-6.ab1 --GGTTTTAT ATTAATATTA AAGAGTTATG GGTTGAAGTT TATATATTTT TTCGTCGAGA ATTTTATATA TTTTTTATCG 78
FWA drm1,2-7.ab1 --GGTTTTAT ATTAATATTA AAGAGTTATG GGTTGAAGTT TATATATTTT TTCGTCGAGA ATTTTATATA TTTTTTATCG 78
FWA drm1,2-8.ab1 TTGGTTTTAT ATTAATATTA AAGAGTTATG GGTTGAAGTT TATATATTTT TTCGTCGAGA ATTTTATATA CTTTTTATCG 80
FWA drm1,2-9.ab1 TTGGTTTTAT ATTAATATTA AAGAGTTATG GGTTGAAGTT TATATATTTT TTCGTCGAGA ATTTTATATA TTTTTTATCG 80
FWA drm1,2-10.ab1 TTGGTTTTAT ATTAATATTA AAGAGTTATG GGTTGAAGTT TATATATTTT TTCGTCGAGA ATTTTATATA TTTTTTATCG 80
FWA drm1,2-11.ab1 TTGGTTTTAT ATTAATATTA AAGAGTTATG GGTTGAAGTT TATATATTTT TTCGTCGAGA ATTTTATATA TTTTTTATCG 80
FWA drm1,2-12.ab1 --GGTTTTAT ATTAATATTA AAGAGTTATG GGTTGAAGTT TATATATTTT TTCGTCGAGA ATTTTATATA TTTTTTATCG 78
FWA drm1,2-13.ab1 --GGTTTTAT ATTAATATTA AAGAGTTATG GGTTGAAGTT TATATATTTT TTCGTCGAGA ATTTTATATA TTTTTTATCG 78
FWA drm1,2-14.ab1 --GGTTTTAT ATTAATATTA AAGAGTTATG GGTTGAAGTT TATATATTTT TTCGTCGAGA ATTTTATATA TTTTTTATCG 78
FWA drm1,2-15.ab1 CTGGTTTTAT ATAAATATTA AAGAGTTATG GGTTGAAGTT TATATATTTT TTCGTCGAGA ATTTTATATA TTTTTTATCG 80
FWA drm1,2-16.ab1 --GGTTTTAT ATTAATATTA AAGAGTTATG GGTTGAAGTT TATATATTTT TTCGTCGAGA ATTTTATATA TTTTTTATCG 78
FWA drm1,2-17.ab1 --GGTTTTAT ATTAATATTA AAGAGTTATG GGTTGAAGTT TATATATTTT TTCGTCGAGA ATTTTATATA TTTTTTATCG 78
FWA drm1,2-18.ab1 TTGGTTTTAT ATTAATATTA AAGAGTTATG GGTTGAAGTT TATATATTTT TTCGTCGAGA ATTTTATATA TTTTTTATCG 80
FWA drm1,2-19.ab1 --GGTTTTAT ATTAATATTA AAGAGTTATG GGTTGAAGTT TATATATTTT TTCGTCGAGA ATTTTATATA TTTTTTATCG 78
FWA drm1,2-20.ab1 TTGGTTTTAT ATTAATATTA AAGAGTTATG GGTTGAAGTT TATATATTTT TTCGTCGAGA ATTTTATATA TTTTTTATCG 80
FWA drm1,2-21.ab1 TTGGTTTTAT ATTAATATTA AAGAGTTATG GGTTGAAGTT TATATATTTT TTCGTCGAGA ATTTTATATA TTTTTTATCG 80
100 120 140 160
fwa repeats AAGCCCATAC ATCTTTCCGT CGAGAATCTC ATATATACCT TATCCCATTC AACATTCATA CGAGCGCCGC TCTAGGGTTT 158
FWA drm1,2-1.ab1 AAGTTTATAT ATTTTTTCGT CGAGAATTTT ATATATATTT TATTTTATTT AATATTTATA CGAGCGTCGT TTTAGGGTTT 158
FWA drm1,2-2.ab1 AAGTTTATAT ATTTTTTCGT CGAGAATTTT ATATATATTT TATTTTATTT AATATTTATA CGAGCGTCGT TTTAGGGTTT 160
FWA drm1,2-3.ab1 AAGTTTATAT ATTTTTTCGT CGAGAATTTT ATATATATTT TATTTTATTT AATATTTATA CGAGCGTCGT TTTAGGGTTT 158
FWA drm1,2-4.ab1 AAGTTTATAT ATCTTTTCGT CGAGAATTTT ATATATATTT TATTTTATTT AATATTTATA CGAGCGTCGT TTTAGGGTTT 160
FWA drm1,2-5.ab1 AAGTTTATAT ATTTTTTCGT CGAGAATCTT ATATATATTT TATTTTATTT AATATTTATA CGAGCGTCGT TTTAGGGTTT 158
FWA drm1,2-6.ab1 AAGTTTATAT ATTTTTTCGT CGAGAATTTT ATATATACTT TATTTTATTT AATATTTATA CGAGCGTCGT TTTAGGGTTT 158
FWA drm1,2-7.ab1 AAGTTTATAT ATTTTTTCGT CGAGAATTTT ATATATATCT TATTTTATTT AATATTTATA CGAGTATCGT TTTACGGTTT 158
FWA drm1,2-8.ab1 AAGTTTATAT ATTTTTTCGT TGAGAATTTT ATATATATTT TATTTTATTT AATATTTATA CGAGCGTCGT TTTAGGGTTT 160
FWA drm1,2-9.ab1 AAGTTTATAT ATTTTTTCGT CGAGAATTTT ATATATATTT TATTTTATTT AATATTCATA CGAGCGTCGT TTTAGGGTTT 160
FWA drm1,2-10.ab1 AAGTTTATAT ATTTTTCCGT CGAGAATTTT ATATATATTT TATTTTATTT AATATTTATA TGAGTGTCGT TTTAGGGTTT 160
FWA drm1,2-11.ab1 AAGTTTATAT ATTTTTCCGT CGAGAATTTT ATATATATTT TATTTTATTT AATATTTATA CGAGCGCCGT TCTAGGGTTT 160
FWA drm1,2-12.ab1 AAGTTTATAT ATTTTTCCGT CGAGAATTTT ATATATATTT TATTTTATTT AATATTTATA CGAGCGTCGT TTTAGGGTTT 158
FWA drm1,2-13.ab1 AAGTTTATAT ATTTTTTCGT CGAGAATTTT ATATATATTT TATTTTATTT AATATTTATG CGAGCGTCGT TTTAGGGTTT 158
FWA drm1,2-14.ab1 AAGTTTATAT ATTTTTTCGT CGAGAATTTT ATATATATTT TATTTTATTT AATATTTATA CGAGCGTCGT TTTAGGGTTT 158
FWA drm1,2-15.ab1 AAGTTTATAT ATTTTTTCGT CGAGAATTTT ATATATATTT TATTTTATTT AATATTTATA CGAGCGTCGT TTTAGGGTTT 160
FWA drm1,2-16.ab1 AAGTTTATAT ATTTTTTCGT CGAGAATTTT ATATATATTT TATTTTATTT AATATTTATA CGAGCGTCGT TTTAGGGTTT 158
FWA drm1,2-17.ab1 AAGTTTATAT ATTTTTTCGT CGAGAATTTT ATATATATTT TATTTTATTT AATATTTATA CGAGCGTCGT TTTAGGGTTT 158
FWA drm1,2-18.ab1 AAGTTTATAT ATTTTTTCGT CGAGAATTTT ATATATATTT TATTTTATTT AATATTTATA CGAGCGTCGT TTTAGGGTTT 160
FWA drm1,2-19.ab1 AAGTTTATAT ATTTTTTCGT CGAGAATTTT ATATATATTT TATTTTATTT AATATTTATA CGAGCGTCGT TTTAGGGTTT 158
FWA drm1,2-20.ab1 AAGTTTATAT ATTTTTTCGT CGAGAATTTT ATATATATTT TATTTTATTT AATATTTATA CGAGCGTCGT TTTAGGGTTT 160
FWA drm1,2-21.ab1 AAGTTTATAT ATTTTTTCGT CGAGAACTTT ATACATATTT TATTTTATTT AATATTTATA CGAGCGTCGT TTTAGGGTTT 160
180 200 220 240
fwa repeats TTGCTTTTCG CCATTGGTCC AAGTGCTATT TGGTTGTTTA AGGTTGCTTT TAGCACACAA CTTTAATATT ATTTTTATGT 238
FWA drm1,2-1.ab1 TTGTTTTTCG TTATTGGTTT AAGTGTTATT TGGTTGTTTA AGGTTGTTTT TAGTATATAA TTTTAATATT ATTTTTATGT 238
FWA drm1,2-2.ab1 TTGTTTTTCG TTATTGGTTT AAGTGTTATT TGGTTGTTTA AGGTTGTTTT TAGTATATAA TTTTAATATT ATTTTTATGT 240
FWA drm1,2-3.ab1 TTGTTTTTCG TTATTGGTTT AAGTGTTATT TGGTTGTTTA AGGTTGTTTT TAGTATATAA TTTTAATATT ATTTTTATGT 238
FWA drm1,2-4.ab1 TTGTTTTTCG TTATTGGTTT AAGTGTTATT TGGTTGTTTA AGGTTGTTTT TAGTATATAA TTTTAATATT ATTTTTATGT 240
FWA drm1,2-5.ab1 TTGTCTTTCG TTATTGGTTC AAGTGTTATT TGGTTGTTTA ATGTTGTTTT TAGTATATAA TTTTAATATT ATTTTTATGT 238
FWA drm1,2-6.ab1 TTGTTTTTCG TTATTGGTTT AAGTGTTATT TGGTTGTTTA AGGTTGTTTT TAGTATATAA TTTTAATATT ATTTTTATGT 238
FWA drm1,2-7.ab1 TTGTTTTTCG ATATTGGTCG AAGTGCTATT TGGTTGTTTA AGGTTGTTTT TAGTATATAA TTTTAATATT ATTTTTATGT 238
FWA drm1,2-8.ab1 TTGTTTTTCG TTATTGGTTT AAGTGTTATT TGGTTGTTTA AGGTTGTTTT TAGTATATAA TTTTAATATT ATTTTTATGT 240
FWA drm1,2-9.ab1 TTGTTTTTCG TTATTGGTTC AAGTGTTATT TGGTTGTTTA AGGTTGTTTT TAGTATATAA TTTTAATATT ATTTTTATGT 240
FWA drm1,2-10.ab1 TTGTTTTTCG TTATTGGTTT AAGTGTTATT TGGTTGTTTA AGGTTGTTTT TAGTATATAA TTTTAATATT ATTTTTATGT 240
FWA drm1,2-11.ab1 TTGTTTTTCG TTATTGGTTC AAGTGCTATT TGGTTGTTTA AGGTTGTTTT TAGTATACAA TTTTAATATT ATTTTTATGT 240
FWA drm1,2-12.ab1 TTGTTTTTCG TTATTGGTTT AAGTGTTATT TGGTTGTTTA AGGTTGTTTT TAGTATATAA TTTTAATATT ATTTTTATGT 238
FWA drm1,2-13.ab1 TTGTTTTTCG TTATTGGTTT AAGTGTTATT TGGTTGTTTA AGGTTGTTTT TAGTATATAA TTTTAATATT ATTTTTATGT 238
FWA drm1,2-14.ab1 TTGTTTTTCG TTATTGGTTT AAGTGTTATT TGGTTGTTTA AGGTTGTTTT TAGTATATAA TTTTAATATT ATTTTTATGT 238
FWA drm1,2-15.ab1 TTGTTTTTCG TTATTGGTTT AAGTGTTATT TGGTTGTTTA AGGTTGTTTT TAGTATATAA TTTTAATATT ATTTTTATGT 240
FWA drm1,2-16.ab1 TTGTTTTTCG TTATTGGTTT AAGTGTTATT TGGTTGTTTA AGGTTGTTTT TAGTATATAA TTTTAATATT ATTTTTATGT 238
FWA drm1,2-17.ab1 TTGTTTTTCG TTATTGGTTT AAGTGTTATT TGGTTGTTTA AGGTTGTTTT TAGTATATAA TTTTAATATT ATTTTTATGT 238
FWA drm1,2-18.ab1 TTGTTTTTCG TTATTGGTTT AAGTGTTATT TGGTTGTTTA AGGTTGTTTT TAGTATATAA TTTTAATATT ATTTTTATGT 240
FWA drm1,2-19.ab1 TTGTTTTTCG TTATTGGTTT AAGTGTTATT TGGTTGTTTA AGGTTGTTTT TAGTATATAA TTTTAATATT ATTTTTATGT 238
FWA drm1,2-20.ab1 TTGTTTTTCG TTATTGGTTT AAGTGTTATT TGGTTGTTTA AGGTTGTTTT TAGTATATAA TTTTAATATT ATTTTTATGT 240
FWA drm1,2-21.ab1 TTGTTTTTCG TTATTGGTTT AAGTGTTATT TGGTTGTTTA AGGTTGTTTT TAGTATATAA TTTTAGTATT ATTTTTATGT 240

		260		280		300		320	
fwa repeats	TTTTTTTCTT	ACGATTTATC	GATTTGTGGG	ATACTGACAA	TCAGATTATT	GTTGTTTTTT	CCAGCCAAAT	ATCAGATCTT	318
FWA drm1,2-1.ab1	TTTTTTTTTT	ACGATTTATC	GATTTGTGGG	ATATTGATAA	TTAGATTATT	GTTGTTTTTT	TTAGTTAAAT	ATTAGATTTT	318
FWA drm1,2-2.ab1	TTTTTTTTTT	ACGATTTATC	GATTTGTGGG	ATATTGATAA	TTAGATTATT	GTTGTTTTTT	TTAGTTAAAT	ATTAGATTTT	320
FWA drm1,2-3.ab1	TTTTTTTTTT	ACGATTTATC	GATTTGTGGG	ATATTGATAA	TTAGATTATT	GTTGTTTTTT	TTAGTTAAAT	ATTAGATTTT	318
FWA drm1,2-4.ab1	TTTTTTTTTT	ACGATTTATC	GATTTGTGGG	ATATTGATAA	TTAGATTATT	GTTGTTTTTT	TTAGTTAAAT	ATTAGATTTT	320
FWA drm1,2-5.ab1	TTTTTTTTTT	ACGATTTATC	GATTTGTGGG	ATATTGATAA	TTAGATTATT	GTTGTTTTTT	TTAGTTAAAT	ATTAGATTTT	318
FWA drm1,2-6.ab1	TTTTTTTTTT	ACGATTTATC	GATTTGTGGG	ATATTGATAA	TTAGATTATT	GTTGTTTTTT	TTAGTTAAAT	ATTAGATTTT	318
FWA drm1,2-7.ab1	TTTTTTTTT-	ACGATTTATC	GATTTGTAGG	ATATTGATAA	TTAGATTATT	GTTGTTTTTT	TTAGTTAAAT	ATTAGATTTT	317
FWA drm1,2-8.ab1	TTTTTTTTTT	ACGATTTATC	GATTTGTGGG	ATATTGATAA	TTAGATTATT	GTTGTTTTTT	TTAGTTAAAT	ATTAGATTTT	320
FWA drm1,2-9.ab1	TTTTTTTTTT	ACGATTTATC	GATTTGTGGG	ATATTGATAA	TTAGATTATT	GTTGTTTTTT	TTAGTTAAAT	ATTAGATTTT	320
FWA drm1,2-10.ab1	TTTTTTTTTT	ACGATTTATC	GATTTGTGGG	ATATTGATAA	TTAGATTATT	GTTGTTTTTT	TTAGTCAAAT	ATTAGATTTT	320
FWA drm1,2-11.ab1	TTTTTTTTTT	ACGATTTATC	GATTTGTGGG	ATATTGATAA	TTAGATTATT	GTTGTTTTTT	TTAGTTAAAT	ATTAGATTTT	320
FWA drm1,2-12.ab1	TTTTTTTTTT	ACGATTTATC	GATTTGTGGG	ATATTGATAA	TTAGATTATT	GTTGTTTTTT	TTAGTTAAAT	ATTAGATTTT	318
FWA drm1,2-13.ab1	TTTTTTTTT-	ATGATTTATC	GATTTGTGGG	ATATTGATAA	TTAGATTATT	GTTGTTTTTT	TTAGTTAAAT	ATTAGATTTT	317
FWA drm1,2-14.ab1	TTTTTTTTTT	ACGATTTATC	GATTTGTGGG	ATACTGATAA	TTAGATTATT	GTTGTTTTTT	TTAGTTAAAT	ATTAGATTTT	318
FWA drm1,2-15.ab1	TTTTTTTTTT	ACGATTTATC	GATTTGTGGG	ATATTGATAA	TTAGATTATT	GTTGTTTTTT	TTAGTTAAAT	ATTAGATTTT	320
FWA drm1,2-16.ab1	TTTTTTTTTT	ACGATTTACC	GATTTGTGGG	ATATTGATAA	TTAGATTATT	GTTGTTTTTT	TTAGTTAAAT	ATTAGATTTT	318
FWA drm1,2-17.ab1	TTTTTTTTTT	ACGATTTATC	GATTTGTGAG	ATATGGATAA	TTAGATTATT	GTTGTTTTTT	TTAGTTAAAT	ATTAGATTTT	318
FWA drm1,2-18.ab1	TTTTTTTTTT	ACGATTTATC	GATTTGTGGG	ATATTGATAA	TTAGATTATT	GTTGTTTTTT	TTAGTTAAAT	ATTAGATTTT	320
FWA drm1,2-19.ab1	TTTTTTTTTT	ACGATTTATC	GATTTGTGGG	ATATTGATAA	TTAGATTATT	GTTGTTTTTT	TTAGTTAAAT	ATTAGATTTT	318
FWA drm1,2-20.ab1	TTTTTTTTTT	ACGATTTATC	GATTTGTGGG	ATATTGATAA	TTAGATTATT	GTTGTTTTTT	T-AGTTAAAT	ATTAGATTTT	319
FWA drm1,2-21.ab1	TTTTTTTTTT	ACGATTTATC	GATTTGTGGG	ATATTGATAA	TTAGATTATT	GTTGTTTTTT	TTAGTTAAAT	ATTAGATTTT	320

		340		360		380		400	
fwa repeats	GCGCCGCTCT	TTATCCCATT	CAACATTCAT	ACGAGCACCG	CTTTACGGTT	TTTGCTTTTC	GGCATTGGTC	GAAGTGCTAT	398
FWA drm1,2-1.ab1	GCGTCGTTTT	TTATTTTATT	TAATATTTAT	ACGAGTATCG	TTTTACGGTT	TTTGTTTTTC	GATATTGGTC	GAAGTGTTAT	398
FWA drm1,2-2.ab1	GCGTCGTTTT	TTATTTTATT	TAATATTTAT	ACGAGTATCG	TTTTACGGTT	TTTGTTTTTC	GATATTGGTC	GAAGTGTTAT	400
FWA drm1,2-3.ab1	GCGTCGTTTT	TTATTTTATT	TAATATTTAT	ACGAGTATCG	TTTTACGGTT	TTTGTTTTTC	GATATTGGTC	GAAGTGTTAT	398
FWA drm1,2-4.ab1	GCGTCGTTTT	TTATTTTATT	TAATATTTAT	ACGAGTATCG	TTTTACGGTT	TTTGTTTTTC	GATACTGGTC	GAAGTGTTAT	400
FWA drm1,2-5.ab1	GCGTCGTTTT	TTATTTTATT	TAATATTTAT	ACGAGTATCG	TTTTACGGTT	TTTGTTTTTC	GATATTGGTC	GAAGTGTTAT	398
FWA drm1,2-6.ab1	GCGTCGTTTT	TTATTTTATT	TAATATTTAT	ACGAGTATCG	TTTTACGGTT	TTTGTTTTTC	GATATTGGTC	GAAGTGTTAT	398
FWA drm1,2-7.ab1	GCGTCGTTTT	TTATTTTATT	TAATATTTAT	ACGAGTATCG	TTTTACGGTT	TTTGTTTTTC	GACATTGGTC	GAAGTGTTAT	397
FWA drm1,2-8.ab1	GCGTCGTTTT	TTATTTTATT	TAATATTTAT	ACGAGTATCG	TTTTACGGTT	TTTGTTTTTC	GATATTGGTC	GAAGTGTTAT	400
FWA drm1,2-9.ab1	GCGTCGTTTT	TTATTTTATT	TAATATTTAT	ACGAGTACCG	CTTTACGGTT	TTTGTTTTTC	GATATTGGTC	GAAGTGTTAT	400
FWA drm1,2-10.ab1	GCGTCGTTTT	TTATTTTATT	TAATATTTAT	ACGAGTATCG	TTTTACGGTT	TTTGTTTTTC	GATATTGGTC	GAAGTGTTAT	400
FWA drm1,2-11.ab1	GCGTCGTTTT	TTATTTTATT	TAATATTTAT	ACGAGTATCG	TTTTACGGTT	TTTGTTTTTC	GATATTGGTC	GAAGTGTTAT	400
FWA drm1,2-12.ab1	GCGTCGTTTT	TTATTTTATT	TAATATTTAT	ACGAGTATCG	TTTTACGGTT	TTTGTTTTTC	GATATTGGTC	GAAGTGTTAT	398
FWA drm1,2-13.ab1	GCGTCGTTTT	TTATTTTATT	TAATATTTAT	ACGAGTATCG	TTTTACGGTT	TTTGTTTTTC	GATATTGGTC	GAAGTGTTAT	397
FWA drm1,2-14.ab1	GCGTCGTTTT	TTATTTTATT	TAATATTTAT	ACGAGTATCG	TTTTACGGTT	TTTGTTTTTC	GATATTGGTC	GAAGTGTTAT	398
FWA drm1,2-15.ab1	GCGTCGTTTT	TTATTTTATT	TAATATTTAT	ACGAGTATCG	TTTTACGGTT	TTTGTTTTTT	GATATTGGTC	GAAGTGTTAT	400
FWA drm1,2-16.ab1	GCGTCGTTTT	TTATTTTATT	TAATATTTAT	ACGAGTATCG	TTTTACGGTT	TTTGTTTTTC	GATATTGGTC	GAAGTGTTAT	398
FWA drm1,2-17.ab1	GCGTCGTTTT	TTATTTTATT	TAATATTTAT	ACGAGTATCG	TTTTACGGTT	TTTGTTTTTC	GATATTGGTC	GAAGTGTTAT	398
FWA drm1,2-18.ab1	GCGTCGTTTT	TTATTTTATT	TAATATTTAT	ACGAGTATCG	TTTTACGGTT	TTTGTTTTTC	GATATTGGTC	GAAGTGTTAT	400
FWA drm1,2-19.ab1	GCGTCGTTTT	TTATTTTATT	TAATATTTAT	ACGAGTATCG	TTTTACGGTT	TTTGTTTTTC	GATATTGGTC	GAAGTGTTAT	398
FWA drm1,2-20.ab1	GCGTCGTTTT	TTATTTTATT	TAATATTTAT	ACGAGTATCG	TTTTACGGTT	TTTGTTTTTC	GATATTGGTC	GAAGTGTTAT	399
FWA drm1,2-21.ab1	GCGTCGTTTT	TTATTTTATT	TAATATTTAT	ACGAGTATCG	TTTTACGGTT	TTTGTTTTTC	GATATTGGTC	GAAGTGTTAT	400

		420		440		460		480	
fwa repeats	TTGGTTGTTT	AAGGTTGCTT	TTAGCACACA	ACTTTAATAT	TATTTTTATG	TTTTCTTCTT	ACGATTTATC	GATTTGTAGG	478
FWA drm1,2-1.ab1	TCGGTTGTTT	AAGGTTGTTT	TTAGTATATA	ATTTTAATAT	TATTTTTATG	TTTTTTTTTT	ACGATTTATC	GATTTGTAGG	478
FWA drm1,2-2.ab1	TTGGTTGTTT	AAGGTTGTTT	TTAGTATATA	ATTTTAATAT	TATTTTTATG	TTTTTTTTT-	ACGATTTATC	GATTTGTAGG	479
FWA drm1,2-3.ab1	TTGGTTGTTT	AAGGTTGTTT	TTAGTATATA	ATTTTAATAT	TATTTTTATG	TTTTTTTTTT	ACGATTTATC	GATTTGTAGG	478
FWA drm1,2-4.ab1	TTGGTTGTTT	AAGGTTGTTT	TTAGTATATA	ATTTTAATAT	TATTTTTATG	TTTTTTTTTT	ACGATTTATC	GATTTGTAGG	480
FWA drm1,2-5.ab1	TTGGTTGTTT	AAGGTTGTTT	TTAGTATATA	ATTTTAATAT	TATTTTTATG	TTTTTTTTTT	ACGATTTATC	GATTTGTAGG	478
FWA drm1,2-6.ab1	TTGGTTGTTT	AAGGTTGTTT	TTAGTATATA	ATTTTAATAT	TATTTTTATG	TTTTTTTTTT	ACGATTTATC	GATTTGTAGG	478
FWA drm1,2-7.ab1	TTGGTTGTTT	AAGGTTGTTT	TTAGTATATA	ATTTTAATAT	TATTTTTATG	TTTTTTTTTT	ACGATTTATC	GATTTGTAGG	477
FWA drm1,2-8.ab1	TTGGTTGTTT	AAGGTTGTTT	TTAGTATATA	ATTTTAATAT	TATTTTTATG	TTTTTTTTTT	ACGATTTATC	GATTTGCAGG	480
FWA drm1,2-9.ab1	TTGGTTGTTT	AAGGTTGTTT	TTAGTATATA	ATTTTAATAT	TATTTTTATG	TTTTTTTTT-	ACGATTTATC	GATTTGTAGG	479
FWA drm1,2-10.ab1	TTGGTTGTTT	AAGGTTGTTT	T-AGTATATA	ATTTTAATAT	TATTTTTATG	TTTTTTTTTT	ACGATTTATC	GATTTGTAGG	479
FWA drm1,2-11.ab1	TTGGTTGTTT	AAGGTTGTTT	TTAGTATATA	ATTTTAATAT	TATTTTTATG	TTTTTTTTTT	ACGATTTATC	GATTTGTAGG	480
FWA drm1,2-12.ab1	TTGGTTGTTT	AAGGTTGTTT	TTAGTATATA	ATTTTAATAT	TATTTTTATG	TTTTTTTTTT	ACGATTTATC	GATTTGTAGG	478
FWA drm1,2-13.ab1	TTGGTTGTTT	AAGGTTGTTT	TTAGTATATA	ATTTTAATAT	TATTTTTATG	TTTTTTTTTT	ACGATTTATT	GATTTGTAGG	477
FWA drm1,2-14.ab1	TTGGTTGTTT	AAGGTTGTTT	TTAGTATATA	ATTTTAATAT	TATTTTTATG	TTTTTTTTTT	ACGATTTATC	GATTTGTAGG	478
FWA drm1,2-15.ab1	TTGGTTGTTT	AAGGTTGTTT	TTAGTATATA	ATTTCAATAT	TATTTTTATG	TTTTTTTTTT	ATGATTTATC	GATTTGTAGG	480
FWA drm1,2-16.ab1	TTGGTTGTTT	AAGGTTGTTT	TTAGTATATA	ATTTTAATAT	TATTTTTATG	TTTTTTTTTT	ACGATTTATC	GATTTGTAGG	478
FWA drm1,2-17.ab1	TTGGTTGTTT	AAGGTTGTTT	TTAGTATATA	ATTTTAATAT	TATTTTTATG	TTTTTTTTTT	ACGATTTATT	GATTTGTAGG	478
FWA drm1,2-18.ab1	TTGGTTGTTT	AAGGTTGTTT	TTAGTATATA	ATTTTAATAT	-ATTTTTATG	TTTTTTTTTT	ATGATTTATC	GATTTGTAGG	479
FWA drm1,2-19.ab1	TTGGTTGTTT	AAGGTTGTTT	TTAGTATATA	ATTTTAATAT	TATTTTTATG	TTTTTTTTTT	ACGATTTATC	GATTTGTAGG	478
FWA drm1,2-20.ab1	TTGGTTGTTT	AAGGTTGTTT	TTAGTATATA	ATTTTAATAT	TATTTTTATG	TTTTTTTTT-	ACGATTTATC	GATTTGTAGG	479
FWA drm1,2-21.ab1	TTGGTTGTTT	AAGGTTGTTT	TTAGTATATA	ATTTTAATAT	TATTTTTATG	TTTTTTTTTT	ACGATTTATC	GATTTGTAGG	480

500 520 540 560

fwa repeats	ATACTGACAA	TCAGATTTTT	GTTGTTTTTT	TCAGCCAAAA	ATCAGATTTT	TTTACACTTT	GTTTAGAGAA	TGATTTTGGT	558
FWA drm1,2-1.ab1	ATATTGATAA	TTAGATTTTT	GTTGTTTTTT	TTAGTTAAAA	ATTAGATTTT	TTTATATTTT	GTTTAGAGAA	TGATTTTGGT	558
FWA drm1,2-2.ab1	ATATTGATAA	TTAGATTTTT	GTTGTTTTTT	TTAGTTAAAA	ATTAGATTTT	TTTATATTTT	GTTTAGAGAA	TGATTTTGGT	559
FWA drm1,2-3.ab1	ATATTGATAA	TTAGATTTTT	GTTGTTTTTT	TTAGTTAAAA	ATTAGATTTT	TTTATATTTT	GTTTAGAGAA	TGATTTTGGT	558
FWA drm1,2-4.ab1	ATATTGATAA	TTAGATTTTT	GTTGTTTTTT	TTAGTTAAAA	ATTAGATTTT	TTTATATTTT	GTTTAGAGAA	TGATTTTGGT	560
FWA drm1,2-5.ab1	ATATTGATAA	TTAGATTTTT	GTTGTTTTTT	TTAGTTAAAA	ATTAGATTTT	TTTATATTTT	GTTTAGAGAA	TGATTTTGGT	558
FWA drm1,2-6.ab1	ATATTGATAA	TTAGATTTTT	GTTGTTTTTT	TTAGTTAAAA	ATTAGATTTT	TTTATATTTT	GTTTAGAGAA	TGATTTTGGT	558
FWA drm1,2-7.ab1	ATATTGATAA	TTAGATTTTT	GTTGTTTTTT	TTAGTTAAAA	ATTAGATTTT	TTTATATTTT	GTTTAGAGAA	TGATTTTGGT	557
FWA drm1,2-8.ab1	ATATTGATAA	TTAGATTTTT	GTTGTTTTTT	TTAGTTAAAA	ATTAGATTTT	TTTATATTTT	GTTTAGAGAA	TGATTTTGGT	560
FWA drm1,2-9.ab1	ATATTGATAA	TTAGATTTTT	GTTGTTTTTT	TTAGTTAAAA	ATTGGATTTT	TTTATATTTT	GTTTAGAGAA	TGATTTTGGT	559
FWA drm1,2-10.ab1	ATATTGATAA	TTAGATTTTT	----------	----------	----------	-TTATATTTT	GTTTAGAGAA	TGATTTTGGT	528
FWA drm1,2-11.ab1	ATATTGATAA	TTAGATTTTT	GTTGTTTTTT	TTAGTTAAAA	ATTAGATTTT	TTTATATTTT	GTTTAGAGAA	TGATTTTGGT	560
FWA drm1,2-12.ab1	ATATTGATAA	TTAGATTTTT	GTTGTTTTTT	TTAGTTAAAA	ATTAGATTTT	TTTATATTTT	GTTTAGAGAA	TGATTT-GGT	557
FWA drm1,2-13.ab1	ATATTGATAA	TTAGATTTTT	GTTGTTTTTT	TTAGTTAAAA	ATTAGATTTT	TTTATATTTT	GTTTAGAGAA	TGATTTTGGT	557
FWA drm1,2-14.ab1	ATGTTGATAA	TTAGATTTTT	GTTGTTTTTT	TTAGTTAAAA	ATTAGATTTT	TTTATATTTT	GTTTAGAGAA	TGATTTTGGT	558
FWA drm1,2-15.ab1	ATATTGATAA	TTAGATTTTT	GTTGTTTTTT	TTAGTTAAAA	ATTAGATTTT	TTTATATTTT	GTTTAGAGAA	TGATTTTGGT	560
FWA drm1,2-16.ab1	ATATTGATAA	TCAGATTTTT	GTTGTTTTTT	TTAGTTAAAA	ATTAGATTTT	TTTATATTTT	GTTTAGAGAA	TGATTTTGGT	558
FWA drm1,2-17.ab1	ATATTGATAA	TTAGATTTTT	GTTGTTTTTT	TTAGTTAAAA	ATTAGATTTT	TTTATATTTT	GTTTAGAGAA	TGATTTTGGT	558
FWA drm1,2-18.ab1	ATATTGATAA	TTAGATTTTT	GTTGTTTTTT	TTAGTTCCAA	ATCACATTTT	TTTATATTTT	GTTTAGAGAA	TGATTTTGGT	559
FWA drm1,2-19.ab1	ATATTGATAA	TTAGATTTTT	GTTGTTTTTT	TTAGTTAAAA	ATTAGATTTT	TTTATATTTT	GTTTAGAGAA	TGATTTTGGT	558
FWA drm1,2-20.ab1	ATATTGATAA	TTAGATTTTT	GTTGTTCTTT	TTAGTTAAAA	ATTAGATTTT	TTTATATTTT	GTTTAGAGAA	TGATTTTGGT	558
FWA drm1,2-21.ab1	ATATTGATAA	TTAGATTTTT	GTTGTTTTTT	TTAGTTAAAA	ATTAGATTTT	TTTATATTTT	GTTTAGAGAA	TGATTTTGGT	560

fwa repeats	TAAGGGCGAT	T	569
FWA drm1,2-1.ab1	TAAGGGCGAA	T	569
FWA drm1,2-2.ab1	TAAGGGCGAA	T	570
FWA drm1,2-3.ab1	TAAGGGCGAA	T	569
FWA drm1,2-4.ab1	TAAGGGCGAA	T	571
FWA drm1,2-5.ab1	TAAGGGCGAA	T	569
FWA drm1,2-6.ab1	TAAGGGCGAA	T	569
FWA drm1,2-7.ab1	TAAGGGCGAA	T	568
FWA drm1,2-8.ab1	TAAGGGCGAA	T	571
FWA drm1,2-9.ab1	TAAGGGCGAA	T	570
FWA drm1,2-10.ab1	TAAGGGCGAA	T	539
FWA drm1,2-11.ab1	TAAGGGCGAA	T	571
FWA drm1,2-12.ab1	TAAGGGCGAA	T	568
FWA drm1,2-13.ab1	TAAGGGCGAA	T	568
FWA drm1,2-14.ab1	TAAGGGCGAA	T	569
FWA drm1,2-15.ab1	TAAGGGCGAA	T	571
FWA drm1,2-16.ab1	TAAGGGCGAA	T	569
FWA drm1,2-17.ab1	TAAGGGCGAA	T	569
FWA drm1,2-18.ab1	TAAGGGCGAA	C	570
FWA drm1,2-19.ab1	TAAGGGCGAA	T	569
FWA drm1,2-20.ab1	TAAGGGCGAA	T	569
FWA drm1,2-21.ab1	TAAGGGCGAA	T	571

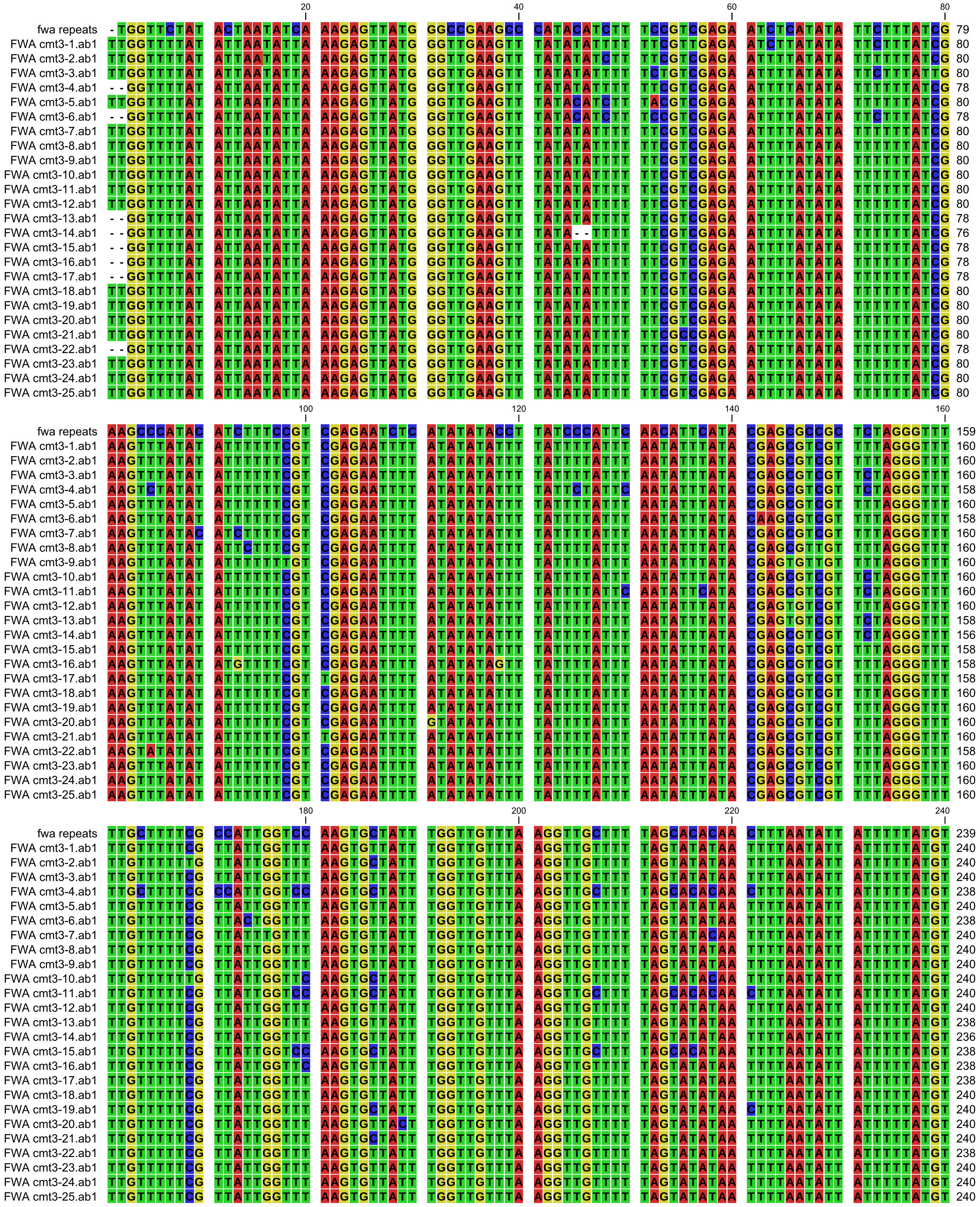
fwa repeats
FWA cmt3-1.ab1
FWA cmt3-2.ab1
FWA cmt3-3.ab1
FWA cmt3-4.ab1
FWA cmt3-5.ab1
FWA cmt3-6.ab1
FWA cmt3-7.ab1
FWA cmt3-8.ab1
FWA cmt3-9.ab1
FWA cmt3-10.ab1
FWA cmt3-11.ab1
FWA cmt3-12.ab1
FWA cmt3-13.ab1
FWA cmt3-14.ab1
FWA cmt3-15.ab1
FWA cmt3-16.ab1
FWA cmt3-17.ab1
FWA cmt3-18.ab1
FWA cmt3-19.ab1
FWA cmt3-20.ab1
FWA cmt3-21.ab1
FWA cmt3-22.ab1
FWA cmt3-23.ab1
FWA cmt3-24.ab1
FWA cmt3-25.ab1

```
                     260                   280                   300                   320
fwa repeats      TTTTTTTCTT ACGATTTATC GATTTGTGGG ATACTGACAA TCAGATTATT GTTGTTTTTT CCAGCCAAAT ATCAGATCTT 319
FWA cmt3-1.ab1   TTTTTTTTTT ACGATTTATC GATTTGTGGG ATATTGATAA TTAGATTATT GTTGTTTTTT TTAGTTAAAT ATTAGATTTT 320
FWA cmt3-2.ab1   TTTTTTTTTT ACGATTTATC GATTTGTGGG ATATTGATAA TTAGATTATT GTTGTTTTTT TTAGTTAAAT ATTAGATTTT 320
FWA cmt3-3.ab1   TTTTTTTTTT ACGATTTATT GATTTGTGGG ATATTGATAA TTAGATTATT GTTGTTTTTT TTAGTTAAAT ATTAGATTTT 320
FWA cmt3-4.ab1   TTTTTTTT-- ACGATTTATT GATTTGTGGG ATATTGATAA TTAGATTATT GTTGTTTTTT TTAGTTAAAT ATTAGATTTT 316
FWA cmt3-5.ab1   TTTTTTTTTT ACGATTTATC GATTTGTGGG ATATTGATAA TTAGATTATT GTTGTTTTTT TTAGTTAAAT ATTAGATTTT 320
FWA cmt3-6.ab1   TTTTTTTTTT ATGATTTATC GATTTGTGGG ATATTGATAA TTAGATTATT GTTGTTTTTT TTAGTTAAAT ATTAGATTTT 318
FWA cmt3-7.ab1   TTTTTTTTTT ACGATTTATC GATTTGTGGG ATATTGATAA TTAGATTATT GTTGTTTTTT TTAGTTAAAT ATTAGATTTT 320
FWA cmt3-8.ab1   TTTTTTTTTT ACGATTTATT GATTTGTGGG ATATTGATAA TTAGATTATT GTTGTTTTTT TTAGTTAAAT ATTAGATTTT 320
FWA cmt3-9.ab1   TTTTTTTTTT ACGATTTATC GATTTGTGGG ATATTGATAA TTAGATTATT GTTGTTTTTT TTAGTTAAAT ATTAGATTTT 320
FWA cmt3-10.ab1  TTTTTTTTTT ACGATTTATC GATTTGTGGG ATATTGACAA TTAGATTATT GTTGTTTTTT TTAGTTAAAT ATTAGATCTT 320
FWA cmt3-11.ab1  TTTTCTTCTT ACGATTTATC GATTTGTGGG ATACTGATAA TTAGATTATT GTTGTTTTTT TTAGTTAAAT ATTAGATTTT 320
FWA cmt3-12.ab1  TTTTCTTCTT ACGATTTATC GATTTGTGGG ATACTGACAA TCAGATTATT GTTGTTTTTT TTAGTTAAAT ATTAGATTTT 320
FWA cmt3-13.ab1  TTTTTTTTTT ACGATTTATT GATTTGTGGG ATATTGATAA TTAGATTATT GTTGTTTTTT TTAGTTAAAT ATTAGATTTT 318
FWA cmt3-14.ab1  TTTTTTTTTT ACGATTTATC GATTTGTGGG ATATTGATAA TTAGATTATT GTTGTTTTTT TTAGTTAAAT ATTAGATTTT 316
FWA cmt3-15.ab1  TTTTTTTTTT ACGATTTATC GATTTGTGGG ATATTGATAA TTAGATTATT GTTGTTTTTT TTAGTTAAAT ATTAGATTTT 318
FWA cmt3-16.ab1  TTTTTTTTTT ACGATTTATC GATTTGTGGG ATATTGATAA TTAGATTATT GTTGTTTTTT TTAGTTAAAT ATTAGATTTT 318
FWA cmt3-17.ab1  TTTTTTTTTT ACGATTTATT GATTTGTGGG ATACTGATAA CTAGATTATT GTTGTTTTTT TTAGTTAAAT ATTAGATTTT 318
FWA cmt3-18.ab1  TTTTTTTTTT ACGATTTATC GATTTGTGGG ATATTGATAA TTAGATTATT GTTGTTTTTT TCAGTCAAAT ATTAGATTTT 320
FWA cmt3-19.ab1  TTTTCTTCTT ACGATTTATT GATTTGTGGG ATATTGATAA TTAGATTATT GTTGTTTTTT TTAGTTAAAT ATTAGATTTT 320
FWA cmt3-20.ab1  TTTTTTTTTT ATGATTTATC GATTTGTGGG ATATTGATAA TTAGATTATT GTTGTTTTTT TTAGTTAAAT GTTAGATTTT 320
FWA cmt3-21.ab1  TTTTTTTTTT ACGATTTATC GATTTGTGGG ATATTGATAA TTAGATTATT GTTGTTTTTT TTAGTTAAAT ATTAGATTTT 320
FWA cmt3-22.ab1  TTTTTTTTTT ACGATTTATC GATTTGTGGG ATATTGATAA TTAGATTATT GTTGTTTTTT TTAGTTAAAT ATTAGATTTT 318
FWA cmt3-23.ab1  TTTTTTTTTT TCGATTTATC GATTTGTGGG ATATTGATAA TTAGATTATT GTTGTTTTTT TTAGTTAAAT ATTAGATTTT 320
FWA cmt3-24.ab1  TTTTTTTTTT ACGATTTGTC GATTTGTGGG ATATTGATAA TTAGATTATT GTTGTTTTTT TTAGTTAAAT ATTAGATTTT 320
FWA cmt3-25.ab1  TTTTTTTTT- ACGATTTATC GATTTGTGGG ATATTGATAA TTAGATTATT GTTGTTTTTT TTAGTTAAAT ATTAGATTTT 319

                     340                   360                   380                   400
fwa repeats      GCGCCGCTCT TTATCCCATT CAACATTCAT ACGAGCACCG CTTTACGGTT TTTGCTTTTC GACATTGGTC GAAGTGCTAT 399
FWA cmt3-1.ab1   GCGTCGTTTT TTAATTTATT TAATATTTAT ACGAGTATCG TTCTACGGTT TTTGTTTTTC GATATTGGTC GAAGTGTTAT 400
FWA cmt3-2.ab1   GCGTCGTTTT TTATTTTATT TAATATTTAT ACGAGTATCG TTTTACGGTT TTTGTTTTTC GATATTGGTC GAAGTGTTAT 400
FWA cmt3-3.ab1   GCGTCGTTTT TTATTTTATT TAATATTTAT ACGAGTATCG TTTTATGGTT TTTGTTTTTC GATATTGGTT GAAGTGTTAT 400
FWA cmt3-4.ab1   GCGTCGTTTT TTATTTTATT TAATATTTAT ACGAGTATCG TTTTACGGTT TTTGTTTTTC GATATTGGTC GAAGTGTTAT 396
FWA cmt3-5.ab1   GCGTCGCTTT TTATTTTATT TAATATTTAT ACGAGTATCG TTTTACGGTT TTTGTTTTTC GATATTGGTC GAAGTGCTAT 400
FWA cmt3-6.ab1   GCGTTGTTTT TTATTTTATT TAATATTTAT ACGAGTATCG TTTTATGGTT TTTGTTTTTC GATATTGGTC GAAGTGTTAT 398
FWA cmt3-7.ab1   GCGTCGTTTT TTATTTTATT TAATATTTAT ACGAGTATCG TTTTACGGTT TTTGTTTTTC GATATTGGTC GAAGTGTTAT 400
FWA cmt3-8.ab1   GCGTCGTTTT TTATTTTATT TAATATTTAT ACGAGTATCG TTTTACGGTT TTTGTTTTTC GATATTGGTC GAAGTGTTAT 400
FWA cmt3-9.ab1   GCGTCGTTTT TTATTTTATT TAATATTTAT ACGAGTATCG TTTTACGGTT TTTGTTTTTC GATATTGGTT GAAGTGTTAT 400
FWA cmt3-10.ab1  GCGTCGTTTT TTATTTTATT TAATATTTAT ACGAGTATCG TTTTACGGTT TTTGTTTTTC GATATTGGTC GAAGTGTTAT 400
FWA cmt3-11.ab1  GCGTCGTTTT TTATCTTATT TAATATTTAT ACGAGTATCG TTTTACGGTT TTTGTTTTTC GATATTGGTC GAAGTGTTAT 400
FWA cmt3-12.ab1  GCGTTGTTTT TTATTTTATT TAATATTTAT ACGAGTATCG TTTTACGGTT TTTGTTTTTC GATATTGGTC GAAGTGTTAT 400
FWA cmt3-13.ab1  GCGTCGTTTT TTATTTTATT TAATATTTAT ACGAGTATCG TTTTACGGTT TTTGTTTTTC GATATTGGTC GAAGTGTTAT 398
FWA cmt3-14.ab1  GCGTTGTTTT TTATTTTATT TAATATTTAT ACGAGTATCG TTTTACGGTT TTTGTTTTTC GATATTGGTC GAAGTGTTAT 396
FWA cmt3-15.ab1  GCGTCGTTCT TTATTTTATT TAATATTTAT ACGAGTATCG TTTTACGGTT TTTGTTTTTC GATATTGGTC GAAGTGTTAT 398
FWA cmt3-16.ab1  GCGTCGTTTT TTATTTTATT TAATATTTAT ACGAGTATCG TTTTACGGTT TTTGTTTTTC GATATTGGTC GAAGTGTTAT 398
FWA cmt3-17.ab1  GCGTCGTTTT TTATTTTATT TAATATTTAT ACGAGTATCG TTTTACGGTT TTTGTTTTTC GATATTGGTC GAAGTGTTAT 398
FWA cmt3-18.ab1  GCGTCGTTTT TTATTTTATT TAATATTTAT ACGAGTATCG TTTTACGGTT TTTGTTTTTC GATATTGGTC GAAGTGTTAT 400
FWA cmt3-19.ab1  GCGTCGTTTT TTATTTTATT TAATATTTAT ACGAGTATTG TTTTACGGTT TTTGCTTTTC GATATTGGTC GAAGTGCTAT 400
FWA cmt3-20.ab1  GCGTCGTTTT TTATTTTATT TAATATTTAT ACGAGTATCG TTTTACGGTT TTTGTTTTTC GATATTGGTC GAAGTGTTAT 400
FWA cmt3-21.ab1  GCGTCGTTTT TTATTTTATT TAATATTTAT ACGAGTATCG TTTTACGGTT TTTGTTTTTC GATATTGGTC GAAGTGTTAT 400
FWA cmt3-22.ab1  GCGTCGTTTT TTATTTTATT TAATATTTAT ACGAGTATCG TTTTACGGTT TTTGTTTTTC GATATTGGTC GAAGTGTTAT 398
FWA cmt3-23.ab1  GCGTCGTTTT TTATTTTATT TAATATTTAT ACGAGTATCG TTTTACGGTT TTTGTTTTTC GATATTGGTC GAAGTGTTAT 400
FWA cmt3-24.ab1  GCGTCGTTTT TTATTTTATT TAATATTTAT ACGAGTATCG TTTTACGGTT TTTGTTTTTC GATATTGGTC GAAGTGTTAT 400
FWA cmt3-25.ab1  GCGTCGTTTT TTATTTTATT TAATATTTAT ACGAGTATCG TTTTACGGTT TTTGTTTTTC GATATTGGTC GAAGTGTTAT 399

                     420                   440                   460                   480
fwa repeats      TTGGTTGTTT AAGGTTGCTT TTAGCACACA ACTTTAATAT TATTTTTATG TTTTCTTCTT ACGATTTATC GATTTGTAGG 479
FWA cmt3-1.ab1   TTGGTTGTTT AAGGTTGTTT TTAGTATACA ATTTTAATAT TATTTTTATG TTTTCTTTTT ACGATTTATC GATTTGTAGG 480
FWA cmt3-2.ab1   TTGGTTGTTT AAGGTTGTTT TTAGTATATA ATATTAATAT TATTTTTATG TTTTTTTTTT ACGATTTATC GATTTGTGGG 480
FWA cmt3-3.ab1   TTGGTTGTTT AAGGTTGTTT TTAGTATACA ACTTTAATAT TATTTTTATG TTTTCTTCTT ATGATTTATC GATTTGTAGG 480
FWA cmt3-4.ab1   TTGGTTGTTT AAGGTTGTTT TTAGTATATA ATTTTAATAT TATTTTTATG TTTTTTTTTT ACGATTTATC GATTTGTAGG 476
FWA cmt3-5.ab1   TTGGTTGTTT AAGGTTGTTT TTAGTATATA ATTTTAATAT TATTTTTATG TTTTTTTTTT ATGATTTATC GAATTGTAGG 480
FWA cmt3-6.ab1   TTGGTTGTTT AAGGTTGTTT TTAGTATATA ATTTTAATAT TATTTTTATG TTTTTTTTTT ACGATTTATC GATTTGTAGG 478
FWA cmt3-7.ab1   TTGGTTGTTT AAGGTTGTTT TTAGTATATA ATTTTAATAT TATTTTTATG TTTTTTTTTT ACGATTTATC GATTTGTAGG 480
FWA cmt3-8.ab1   TTGGTTGTTT AAGGTTGTTT TTAGTATATA ATTTTAATAT TATTTTTATG TTTTTTTTTT ATGATTTATC GATTTGTAGG 480
FWA cmt3-9.ab1   TTGGTTGTTT AAGGTTGTTT TTAGTATATA ATTTTAATAT TATTTTTATG TTTTTTTTTT ACGATTTATC GATTTGTAGG 480
FWA cmt3-10.ab1  TTGGTTGTTT AAGGTTGCTT TTAGCACACA ACTTTAATAT TATTTTTATG TTTTCTTCTT ATGATTTATT GATTTGTAGG 480
FWA cmt3-11.ab1  TTGGTTGTTT AAGGTTGTTT TTAGTATATA ATTTTAATAT TATTTTTATG TTTTTTTTTT ACGATTTATC GATTTGTAGG 480
FWA cmt3-12.ab1  TTGGTTGTTT AAGGTTGTTT TTAGTATATA ATTTTAATAT TATTTTTATG TTTTCTTTTT ACGATTTATC GATTTGTAGG 480
FWA cmt3-13.ab1  TTGGTTGTTT AAGGTTGTTT TTAGTATATA ATTTTAATAT TATTTTTATG TTTTTTTTTT ATGATTTATC GATTTGTAGG 478
FWA cmt3-14.ab1  TTGGTTGTTT AAGGTTGTTT TTAGTATATA ATTTTAATAT TATTTTTATG TTTTTTTTTT ACGATTTATC GATTTGTAGG 476
FWA cmt3-15.ab1  TTGGTTGTTT AAGGTTGTTT TTAGCACACA ACTTTAATAT TATTTTTATG TTTTTTTTTT ACGATTTATC GATTTGTAGG 478
FWA cmt3-16.ab1  TTGGTTGTTT AAGGTTGTTT TTAGTATATA ATTTTAATAT TATTTTTATG TTTTTTTTTT ACGATTTATC GATTTGTAGG 478
FWA cmt3-17.ab1  TTGGTTGTTT AAGGTTGTTT TTAGTATATA ATATTAATAT TATTTTTATG TTTTTTTTTT ACGATTTATC GATTTGTAGG 478
FWA cmt3-18.ab1  TTGGTTGTTT AAGGTTGTTT TTAGTATATA ATTTTAATAT TATTTTTATG TTTTTTTTTT ACGATTTATC GATTTGTAGG 480
FWA cmt3-19.ab1  TTGGTTGTTT AAGGTTGCTT TTAGCACACA ATTTTAATAT TATTTTTATG TTTTTTTTTT ATGATTTATT GATTTGTAGG 480
FWA cmt3-20.ab1  TTGGTTGTTT AAGGTTGTTT TTAGTATATA ATTTTAATAT TATTTTTATG TTTTTTTTT- ATGATTTATC GATTTGTAGG 479
FWA cmt3-21.ab1  TTGGTTGTTT AAGGTTGTTT TTAGTATATA ATTTTAATAT TATTTTTATG TTTTTTTTTT ACGATTTATC GATTTGTAGG 480
FWA cmt3-22.ab1  TTGGTTGTTT AAGGTTGTTT TTAGTATATA ATTTTAATAT TATTTTTATG TTTTTTTTTT ACGATTTATC GATTTGTAGG 478
FWA cmt3-23.ab1  TTGGTTGTTT AAGGTTGTTT TTAGTATATA ATTTTAATAT TATTTTTATG TTTTTTTTTT ACGATTTATC GATTTGTAGG 480
FWA cmt3-24.ab1  TTGGTTGTTT AAGGTTGTTT TTAGTATATA ATTTTAATAT TATTTTTATG TTTTTTTTTT ACGATTTATC GATTTGTAGG 480
FWA cmt3-25.ab1  TTGGTTGTTT AAGGTTGTTT TTAGTGTATA ATTTTAATAT TATTTTTATG TTTTTTTTTT ACGATTTATC GATTTGTAGG 479
```

```
                                                           500                                520                                540                                560
                                                             |                                  |                                  |                                  |
fwa repeats      ATACTGACAA  TCAGATTTTT  GTTGTTTTTT  TCAGCCAAAA  ATCAGATTTT  TTTACACTTT  GTTTAGAGAA  TGATTTTGGT  559
FWA cmt3-1.ab1   ATATTGATAA  TTAGATTTTT  GTTGTTTTTT  TTAGTTAAAA  ATTAGATTTT  TTTATATTTT  GTTTAGAGAA  TGATTTTGGT  560
FWA cmt3-2.ab1   ATATTGATAA  TTAGATTTTT  GTTGTTTTTT  TTAGTTAAAA  ATTAGATTTT  TTTATATTTT  GTTTAGAGAA  TGATTTTGGT  560
FWA cmt3-3.ab1   ATATTGATAA  TTAGATTTTT  GTTGTTTTTT  TTAGTTAAAA  ATTAGATTTT  TTTATATTTT  GTTTAGAGAA  TGATTTTGGT  560
FWA cmt3-4.ab1   ATATTGATAA  TTAGATTTTT  GTTGTTTTTT  TTAGTTAAAA  ATTAGATTTT  TTTATATTTT  GTTTAGAGAA  TGATTTTGGT  556
FWA cmt3-5.ab1   ATATTGATAA  TTAGATTTTT  GTTGTTTTTT  T-AGTCAAAA  ATTAGATTTT  TTTATATTTT  GTTTAGAGAA  TGATTTTGGT  559
FWA cmt3-6.ab1   ATATTGATAA  TTAGATTTTT  GTTGTTTTTT  TTAGTTAAAA  ATTAGATTTT  TTTATATTTT  GTTTAGAGAA  TGATTTTGGT  558
FWA cmt3-7.ab1   ATATTGATAA  TTAGATTTTT  GTTGTTTTTT  TTAGTTAAAA  ATTAGATTTT  TTTATATTTT  GTTTAGAGAA  TGATTTTGGT  560
FWA cmt3-8.ab1   ATATTGATAA  TTAGATTTTT  GTTGTTTTTT  TTAGTTAAAA  ATTAGATTTT  TTTATATTTT  GTTTAGAGAA  TGATTTTGGT  560
FWA cmt3-9.ab1   ATATTGATAA  TTAGATTTTT  GTTGTTTTTT  TTAGTTAAAA  ATTAGATTTT  TTTATATTTT  GTTTAGAGAA  TGATTTTGGT  560
FWA cmt3-10.ab1  ATATTGATAA  TTAGATTTTT  GTTGTTTTTT  TTAGTTAAAA  ATTAGATTTT  TTTATATTTT  GTTTAGAGAA  TGATTTTGGT  560
FWA cmt3-11.ab1  ATATTGATAA  TTAGATTTTT  GTTGTTTTTT  TTAGTTAAAA  ATTAGATTTT  TTTATATTTT  GTTTAGAGAA  TGATTTTGGT  560
FWA cmt3-12.ab1  ATATTGATAA  TTAGATTTTT  GTTGTTTTTT  TTAGTTAAAA  ATTAGATTTT  TTTATATTTT  GTTTAGAGAA  TGATTTTGGT  560
FWA cmt3-13.ab1  ATATTGATAA  TTAGATTTTT  GTTGTTTTTT  TTAGTTAAAA  ATTAGATTTT  TTTATATTTT  GTTTAGAGAA  TGATTTTGGT  558
FWA cmt3-14.ab1  ATATTGATAA  TTAGATTTTT  GTTGTTTTTT  TTAGTTAAAA  ATTAGATTTT  TTTATATTTT  GTTTAGAGAA  TGATTTTGGT  556
FWA cmt3-15.ab1  ATATTGATAA  TTAGATTTTT  GTTGTTTTTT  TTAGTTAAAA  ATTAGATTTT  TTTATATTTT  GTTTAGAGAA  TGATTTTGGT  558
FWA cmt3-16.ab1  ATATTGATAA  TTAGATTTTT  GTTGTTTTTT  TTAGTTAAAA  ATTAGATTTT  TTTATATTTT  GTTTAGAGAA  TGATTTTGGT  558
FWA cmt3-17.ab1  ATATTGATAA  TTAGATTTTT  GTTGTTTTTT  TTAGTTAAAA  ATTAGATTTT  TTTATATTTT  GTTTAGAGAA  TGATTTTGGT  558
FWA cmt3-18.ab1  ATATTGATAA  TTAGATTTTT  GTTGTTTTTT  TTAGTTAAAA  ATTAGATTTT  TTTATATTTT  GTTTAGAGAA  TGATTTTGGT  560
FWA cmt3-19.ab1  ATATTGATAA  TTAGATTTTT  GTAGTTTTTT  TTAGTTAAAA  ATTAGATTTT  TTTATATTTT  GTTTAGAGAA  TGATTTTGGT  560
FWA cmt3-20.ab1  ATATTGATAA  TTAGATTTTT  GTTGTTTTTT  TTAGTTAAAA  ATTAGATTTT  TTTATATTTT  GTTTAGAGAA  TGATTTTGGT  559
FWA cmt3-21.ab1  ATATTGATAA  TTAGATTTTT  GTTGTTTTTT  TTAGTTAAAA  ATTAGATTTT  TTTATATTTT  GTTTAGAGAA  TGATTTTGGT  560
FWA cmt3-22.ab1  ATATTGATAA  TTAGATTTTT  GTTGTTTTAT  TTAGTTAAAA  ATTAGATTTT  TTTATATTTT  GTTTAGAGAA  TGATTTTGGT  558
FWA cmt3-23.ab1  ATATTGATAA  TTAGATTTTT  GTTGTTTTTT  TTAGTTAAAA  ATTAGATTTT  TTTATATTTT  GTTTAGAGAA  TGATTTTGGT  560
FWA cmt3-24.ab1  ATATTGATAA  TTAGATTTTT  GTTGTTTTTT  TTAGTTAAAA  ATTAGATTTT  TTTATATTTT  GTTTAGAGAA  TGATTTTGGT  560
FWA cmt3-25.ab1  ATATTGATAA  TTAGATTTTT  GTTGTTTTTT  TTAGTTAAAA  ATTAGATTTT  TTTATATTTT  GTTTAGAGAA  TGATTTTGGT  559

fwa repeats      TAAGGGCGAT  TTG  572
FWA cmt3-1.ab1   TAAGGGCGAA  TTC  573
FWA cmt3-2.ab1   TAAGGGCGAA  TTC  573
FWA cmt3-3.ab1   TAAGGGCGAA  TTC  573
FWA cmt3-4.ab1   TAAGGGCGAA  TTC  569
FWA cmt3-5.ab1   TAAGGGCGAA  TTC  572
FWA cmt3-6.ab1   TAAGGGCGAA  TTC  571
FWA cmt3-7.ab1   TAAGGGCGAA  TTC  573
FWA cmt3-8.ab1   TAAGGGCGAA  TTC  573
FWA cmt3-9.ab1   TAAGGGCGAA  TTC  573
FWA cmt3-10.ab1  TAAGGGCGAA  TTC  573
FWA cmt3-11.ab1  TAAGGGCGAA  TTC  573
FWA cmt3-12.ab1  TAAGGGCGAA  TTC  573
FWA cmt3-13.ab1  TAAGGGCGAA  TTC  571
FWA cmt3-14.ab1  TAAGGGCGAA  TTC  569
FWA cmt3-15.ab1  TAAGGGCGAA  TTC  571
FWA cmt3-16.ab1  TAAGGGCGAA  TTC  571
FWA cmt3-17.ab1  TAAGGGCGAA  TTC  571
FWA cmt3-18.ab1  TAAGGGCGAA  TTC  573
FWA cmt3-19.ab1  TAAGGGCGAA  TTC  573
FWA cmt3-20.ab1  TAAGGGCGAA  TTC  572
FWA cmt3-21.ab1  TAAGGGCGAA  TTC  573
FWA cmt3-22.ab1  TAAGGGCGAA  TTC  571
FWA cmt3-23.ab1  TAAGGGCGAA  TTC  573
FWA cmt3-24.ab1  TAAGGGCGAA  TTC  573
FWA cmt3-25.ab1  TAAGGGCGAA  TTC  572
```

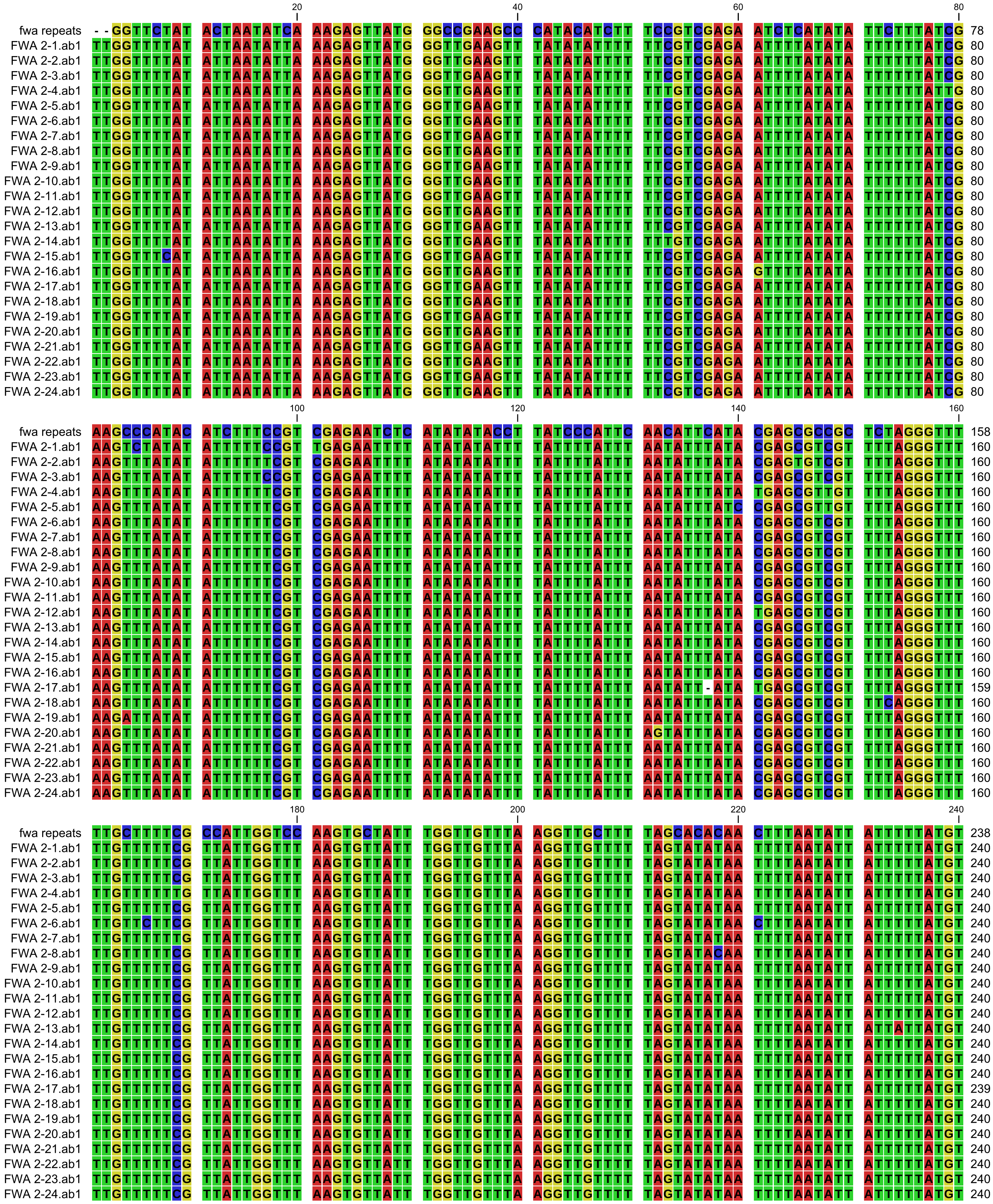
20 40 60 80
fwa repeats --GGTTCTAT ACTAATATCA AAGAGTTATG GGCCGAAGCC CATACATCTT TCCGTCGAGA ATCTCATATA TTCTTTATCG 78
FWA 2-1.ab1 TTGGTTTTAT ATTAATATTA AAGAGTTATG GGTTGAAGTT TATATATTTT TTCGTCGAGA ATTTTATATA TTTTTTATCG 80
FWA 2-2.ab1 TTGGTTTTAT ATTAATATTA AAGAGTTATG GGTTGAAGTT TATATATTTT TTCGTCGAGA ATTTTATATA TTTTTTATCG 80
FWA 2-3.ab1 TTGGTTTTAT ATTAATATTA AAGAGTTATG GGTTGAAGTT TATATATTTT TTCGTCGAGA ATTTTATATA TTTTTTATCG 80
FWA 2-4.ab1 TTGGTTTTAT ATTAATATTA AAGAGTTATG GGTTGAAGTT TATATATTTT TTTGTCGAGA ATTTTATATA TTTTTTATTG 80
FWA 2-5.ab1 TTGGTTTTAT ATTAATATTA AAGAGTTATG GGTTGAAGTT TATATATTTT TTCGTCGAGA ATTTTATATA TTTTTTATCG 80
FWA 2-6.ab1 TTGGTTTTAT ATTAATATTA AAGAGTTATG GGTTGAAGTT TATATATTTT TTCGTCGAGA ATTTTATATA TTTTTTATCG 80
FWA 2-7.ab1 TTGGTTTTAT ATTAATATTA AAGAGTTATG GGTTGAAGTT TATATATTTT TTCGTCGAGA ATTTTATATA TTTTTTATCG 80
FWA 2-8.ab1 TTGGTTTTAT ATTAATATTA AAGAGTTATG GGTTGAAGTT TATATATTTT TTCGTCGAGA ATTTTATATA TTTTTTATCG 80
FWA 2-9.ab1 TTGGTTTTAT ATTAATATTA AAGAGTTATG GGTTGAAGTT TATATATTTT TTCGTCGAGA ATTTTATATA TTTTTTATCG 80
FWA 2-10.ab1 TTGGTTTTAT ATTAATATTA AAGAGTTATG GGTTGAAGTT TATATATTTT TTCGTCGAGA ATTTTATATA TTTTTTATCG 80
FWA 2-11.ab1 TTGGTTTTAT ATTAATATTA AAGAGTTATG GGTTGAAGTT TATATATTTT TTCGTCGAGA ATTTTATATA TTTTTTATCG 80
FWA 2-12.ab1 TTGGTTTTAT ATTAATATTA AAGAGTTATG GGTTGAAGTT TATATATTTT TTCGTCGAGA ATTTTATATA TTTTTTATCG 80
FWA 2-13.ab1 TTGGTTTTAT ATTAATATTA AAGAGTTATG GGTTGAAGTT TATATATTTT TTCGTCGAGA ATTTTATATA TTTTTTATCG 80
FWA 2-14.ab1 TTGGTTTTAT ATTAATATTA AAGAGTTATG GGTTGAAGTT TATATATTTT TTTGTCGAGA ATTTTATATA TTTTTTATCG 80
FWA 2-15.ab1 TTGGTTTCAT ATTAATATTA AAGAGTTATG GGTTGAAGTT TATATATTTT TTCGTCGAGA ATTTTATATA TTTTTTATCG 80
FWA 2-16.ab1 TTGGTTTTAT ATTAATATTA AAGAGTTATG GGTTGAAGTT TATATATTTT TTCGTCGAGA GTTTTATATA TTTTTTATCG 80
FWA 2-17.ab1 TTGGTTTTAT ATTAATATTA AAGAGTTATG GGTTGAAGTT TATATATTTT TTCGTCGAGA ATTTTATATA TTTTTTATCG 80
FWA 2-18.ab1 TTGGTTTTAT ATTAATATTA AAGAGTTATG GGTTGAAGTT TATATATTTT TTCGTCGAGA ATTTTATATA TTTTTTATCG 80
FWA 2-19.ab1 TTGGTTTTAT ATTAATATTA AAGAGTTATG GGTTGAAGTT TATATATTTT TTCGTCGAGA ATTTTATATA TTTTTTATCG 80
FWA 2-20.ab1 TTGGTTTTAT ATTAATATTA AAGAGTTATG GGTTGAAGTT TATATATTTT TTCGTCGAGA ATTTTATATA TTTTTTATCG 80
FWA 2-21.ab1 TTGGTTTTAT ATTAATATTA AAGAGTTATG GGTTGAAGTT TATATATTTT TTCGTCGAGA ATTTTATATA TTTTTTATCG 80
FWA 2-22.ab1 TTGGTTTTAT ATTAATATTA AAGAGTTATG GGTTGAAGTT TATATATTTT TTCGTCGAGA ATTTTATATA TTTTTTATCG 80
FWA 2-23.ab1 TTGGTTTTAT ATTAATATTA AAGAGTTATG GGTTGAAGTT TATATATTTT TTCGTCGAGA ATTTTATATA TTTTTTATCG 80
FWA 2-24.ab1 TTGGTTTTAT ATTAATATTA AAGAGTTATG GGTTGAAGTT TATATATTTT TTCGTCGAGA ATTTTATATA TTTTTTATCG 80
100 120 140 160
fwa repeats AAGCCCATAC ATCTTTCCGT CGAGAATCTC ATATATACCT TATCCCATTC AACATTCATA CGAGCGCCGC TCTAGGGTTT 158
FWA 2-1.ab1 AAGTCTATAT ATTTTTCCGT TGAGAATTTT ATATATATTT TATTTTATTT AATATTTATA CGAGCGTCGT TTTAGGGTTT 160
FWA 2-2.ab1 AAGTTTATAT ATTTTTTCGT CGAGAATTTT ATATATATTT TATTTTATTT AATATTTATA CGAGTGTCGT TTTAGGGTTT 160
FWA 2-3.ab1 AAGTTTATAT ATTTTTCCGT CGAGAATTTT ATATATATTT TATTTTATTT AATATTTATA CGAGCGTCGT TTTAGGGTTT 160
FWA 2-4.ab1 AAGTTTATAT ATTTTTTCGT CGAGAATTTT ATATATATTT TATTTTATTT AATATTTATA TGAGCGTTGT TTTAGGGTTT 160
FWA 2-5.ab1 AAGTTTATAT ATTTTTTCGT CGAGAATTTT ATATATATTT TATTTTATTT AATATTTATC CGAGCGTTGT TTTAGGGTTT 160
FWA 2-6.ab1 AAGTTTATAT ATTTTTTCGT CGAGAATTTT ATATATATTT TATTTTATTT AATATTTATA CGAGCGTCGT TTTAGGGTTT 160
FWA 2-7.ab1 AAGTTTATAT ATTTTTTCGT CGAGAATTTT ATATATATTT TATTTTATTT AATATTTATA CGAGCGTCGT TTTAGGGTTT 160
FWA 2-8.ab1 AAGTTTATAT ATTTTTTCGT CGAGAATTTT ATATATATTT TATTTTATTT AATATTTATA CGAGCGTCGT TTTAGGGTTT 160
FWA 2-9.ab1 AAGTTTATAT ATTTTTTCGT CGAGAATTTT ATATATATTT TATTTTATTT AATATTTATA CGAGCGTCGT TTTAGGGTTT 160
FWA 2-10.ab1 AAGTTTATAT ATTTTTTCGT CGAGAATTTT ATATATATTT TATTTTATTT AATATTTATA CGAGCGTCGT TTTAGGGTTT 160
FWA 2-11.ab1 AAGTTTATAT ATTTTTTCGT CGAGAATTTT ATATATATTT TATTTTATTT AATATTTATA CGAGCGTCGT TTTAGGGTTT 160
FWA 2-12.ab1 AAGTTTATAT ATTTTTTCGT CGAGAATTTT ATATATATTT TATTTTATTT AATATTTATA TGAGCGTCGT TTTAGGGTTT 160
FWA 2-13.ab1 AAGTTTATAT ATTTTTTCGT CGAGAATTTT ATATATATTT TATTTTATTT AATATTTATA CGAGCGTCGT TTTAGGGTTT 160
FWA 2-14.ab1 AAGTTTATAT ATTTTTTCGT CGAGAATTTT ATATATATTT TATTTTATTT AATATTTATA CGAGCGTCGT TTTAGGGTTT 160
FWA 2-15.ab1 AAGTTTATAT ATTTTTTCGT CGAGAATTTT ATATATATTT TATTTTATTT AATATTTATA CGAGCGTCGT TTTAGGGTTT 160
FWA 2-16.ab1 AAGTTTATAT ATTTTTTCGT CGAGAATTTT ATATATATTT TATTTTATTT AATATTTATA CGAGCGTCGT TTTAGGGTTT 160
FWA 2-17.ab1 AAGTTTATAT ATTTTTTCGT CGAGAATTTT ATATATATTT TATTTTATTT AATATT-ATA TGAGCGTCGT TTTAGGGTTT 159
FWA 2-18.ab1 AAGTTTATAT ATTTTTTCGT CGAGAATTTT ATATATATTT TATTTTATTT AATATTTATA CGAGCGTCGT TTCAGGGTTT 160
FWA 2-19.ab1 AAGATTATAT ATTTTTTCGT CGAGAATTTT ATATATATTT TATTTTATTT AATATTTATA CGAGCGTCGT TTTAGGGTTT 160
FWA 2-20.ab1 AAGTTTATAT ATTTTTTCGT CGAGAATTTT ATATATATTT TATTTTATTT AGTATTTATA CGAGCGTCGT TTTAGGGTTT 160
FWA 2-21.ab1 AAGTTTATAT ATTTTTTCGT CGAGAATTTT ATATATATTT TATTTTATTT AATATTTATA CGAGCGTCGT TTTAGGGTTT 160
FWA 2-22.ab1 AAGTTTATAT ATTTTTTCGT CGAGAATTTT ATATATATTT TATTTTATTT AATATTTATA CGAGCGTCGT TTTAGGGTTT 160
FWA 2-23.ab1 AAGTTTATAT ATTTTTTCGT CGAGAATTTT ATATATATTT TATTTTATTT AATATTTATA CGAGCGTCGT TTTAGGGTTT 160
FWA 2-24.ab1 AAGTTTATAT ATTTTTTCGT CGAGAATTTT ATATATATTT TATTTTATTT AATATTTATA CGAGCGTCGT TTTAGGGTTT 160
180 200 220 240
fwa repeats TTGCTTTTCG CCATTGGTCC AAGTGCTATT TGGTTGTTTA AGGTTGCTTT TAGCACACAA CTTTAATATT ATTTTTATGT 238
FWA 2-1.ab1 TTGTTTTTCG TTATTGGTTT AAGTGTTATT TGGTTGTTTA AGGTTGTTTT TAGTATATAA TTTTAATATT ATTTTTATGT 240
FWA 2-2.ab1 TTGTTTTTCG TTATTGGTTT AAGTGTTATT TGGTTGTTTA AGGTTGTTTT TAGTATATAA TTTTAATATT ATTTTTATGT 240
FWA 2-3.ab1 TTGTTTTTCG TTATTGGTTT AAGTGTTATT TGGTTGTTTA AGGTTGTTTT TAGTATATAA TTTTAATATT ATTTTTATGT 240
FWA 2-4.ab1 TTGTTTTTTG TTATTGGTTT AAGTGTTATT TGGTTGTTTA AGGTTGTTTT TAGTATATAA TTTTAATATT ATTTTTATGT 240
FWA 2-5.ab1 TTGTTTTTCG TTATTGGTTT AAGTGTTATT TGGTTGTTTA AGGTTGTTTT TAGTATATAA TTTTAATATT ATTTTTATGT 240
FWA 2-6.ab1 TTGTTCTTCG TTATTGGTTT AAGTGTTATT TGGTTGTTTA AGGTTGTTTT TAGTATATAA CTTTAATATT ATTTTTATGT 240
FWA 2-7.ab1 TTGTTTTTTG TTATTGGTTT AAGTGTTATT TGGTTGTTTA AGGTTGTTTT TAGTATATAA TTTTAATATT ATTTTTATGT 240
FWA 2-8.ab1 TTGTTTTTCG TTATTGGTTT AAGTGTTATT TGGTTGTTTA AGGTTGTTTT TAGTATACAA TTTTAATATT ATTTTTATGT 240
FWA 2-9.ab1 TTGTTTTTCG TTATTGGTTT AAGTGTTATT TGGTTGTTTA AGGTTGTTTT TAGTATATAA TTTTAATATT ATTTTTATGT 240
FWA 2-10.ab1 TTGTTTTTCG TTATTGGTTT AAGTGTTATT TGGTTGTTTA AGGTTGTTTT TAGTATATAA TTTTAATATT ATTTTTATGT 240
FWA 2-11.ab1 TTGTTTTTCG TTATTGGTTT AAGTGTTATT TGGTTGTTTA AGGTTGTTTT TAGTATATAA TTTTAATATT ATTTTTATGT 240
FWA 2-12.ab1 TTGTTTTTCG TTATTGGTTT AAGTGTTATT TGGTTGTTTA AGGTTGTTTT TAGTATATAA TTTTAATATT ATTTTTATGT 240
FWA 2-13.ab1 TTGTTTTTCG TTATTGGTTT AAGTGTTATT TGGTTGTTTA AGGTTGTTTT TAGTATATAA TTTTAATATT ATTATTATGT 240
FWA 2-14.ab1 TTGTTTTTCG TTATTGGTTT AAGTGTTATT TGGTTGTTTA AGGTTGTTTT TAGTATATAA TTTTAATATT ATTTTTATGT 240
FWA 2-15.ab1 TTGTTTTTCG TTATTGGTTT AAGTGTTATT TGGTTGTTTA AGGTTGTTTT TAGTATATAA TTTTAATATT ATTTTTATGT 240
FWA 2-16.ab1 TTGTTTTTCG TTATTGGTTT AAGTGTTATT TGGTTGTTTA AGGTTGTTTT TAGTATATAA TTTTAATATT ATTTTTATGT 240
FWA 2-17.ab1 TTGTTTTTCG TTATTGGTTT AAGTGTTATT TGGTTGTTTA AGGTTGTTTT TAGTATATAA TTTTAATATT ATTTTTATGT 239
FWA 2-18.ab1 TTGTTTTTCG TTATTGGTTT AAGTGTTATT TGGTTGTTTA AGGTTGTTTT TAGTATATAA TTTTAATATT ATTTTTATGT 240
FWA 2-19.ab1 TTGTTTTTCG TTATTGGTTT AAGTGTTATT TGGTTGTTTA AGGTTGTTTT TAGTATATAA TTTTAATATT ATTTTTATGT 240
FWA 2-20.ab1 TTGTTTTTCG TTATTGGTTT AAGTGTTATT TGGTTGTTTA AGGTTGTTTT TAGTATATAA TTTTAATATT ATTTTTATGT 240
FWA 2-21.ab1 TTGTTTTTCG TTATTGGTTT AAGTGTTATT TGGTTGTTTA AGGTTGTTTT TAGTATATAA TTTTAATATT ATTTTTATGT 240
FWA 2-22.ab1 TTGTTTTTCG TTATTGGTTT AAGTGTTATT TGGTTGTTTA AGGTTGTTTT TAGTATATAA TTTTAATATT ATTTTTATGT 240
FWA 2-23.ab1 TTGTTTTTCG TTATTGGTTT AAGTGTTATT TGGTTGTTTA AGGTTGTTTT TAGTATATAA TTTTAATATT ATTTTTATGT 240
FWA 2-24.ab1 TTGTTTTTCG TTATTGGTTT AAGTGTTATT TGGTTGTTTA AGGTTGTTTT TAGTATATAA TTTTAATATT ATTTTTATGT 240

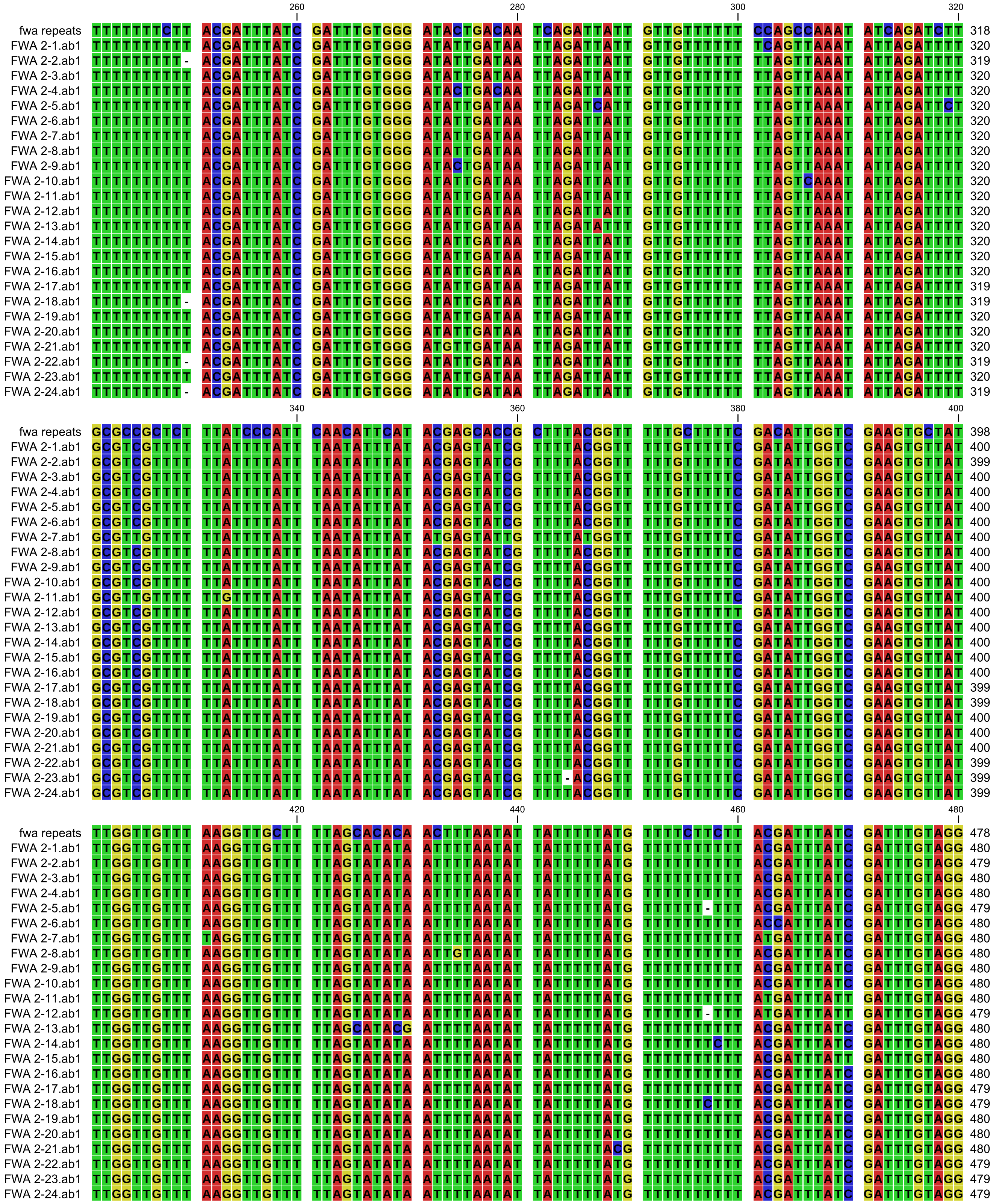
260
280
300
320
340
360
380
400
420
440
460
480
fwa repeats
FWA 2-1.ab1
FWA 2-2.ab1
FWA 2-3.ab1
FWA 2-4.ab1
FWA 2-5.ab1
FWA 2-6.ab1
FWA 2-7.ab1
FWA 2-8.ab1
FWA 2-9.ab1
FWA 2-10.ab1
FWA 2-11.ab1
FWA 2-12.ab1
FWA 2-13.ab1
FWA 2-14.ab1
FWA 2-15.ab1
FWA 2-16.ab1
FWA 2-17.ab1
FWA 2-18.ab1
FWA 2-19.ab1
FWA 2-20.ab1
FWA 2-21.ab1
FWA 2-22.ab1
FWA 2-23.ab1
FWA 2-24.ab1

```
                                          500                                 520                                 540                                 560
                                            |                                   |                                   |                                   |
fwa repeats     ATACTGACAA  TCAGATTTTT  GTTGTTTTTT  TCAGCCAAAA  ATCAGATTTT  TTTACACTTT  GTTTAGAGAA  TGATTTTGGT  558
FWA 2-1.ab1     ATATTGATAA  TTAGATTTTT  GTTGTTTTTT  TTAGTTAAAA  ATTAGATTTT  TTTATATTTT  GTTTAGAGAA  TGATTTTGGT  560
FWA 2-2.ab1     ATACTGATAA  TTAGATTTTT  GTTGTTTTTT  TTAGTTAAAA  ATTAGATTTT  TTTATATTTT  GTTTAGAGAA  TGATTTTGGT  559
FWA 2-3.ab1     ATATTGATAA  TTAGATTTTT  GTTGTTTTTT  TTAGTTAAAA  ATTAGATTTT  TTTATATTTT  GTTTAGAGAA  TGATTTTGGT  560
FWA 2-4.ab1     ATATTGATAA  TTAGATTTTT  GTTGTTTTTT  TTAGTTAAAA  ATTAGATTTT  TTTATATTTT  GTTTAGAGAA  TGATTTTGGT  560
FWA 2-5.ab1     ATATTGATAA  TTAGATTTTT  GTTGGTTTTT  TTAGTTAAAA  ATTAGATTTT  TTTATATTTT  GTTTAGAGAA  TGATTTTGGT  559
FWA 2-6.ab1     ATATTGATAA  TTAGATTTTT  GTTGTTTTTT  TTAGTTAAAA  ATTAGATTTT  TTTATATTTT  GTTTAGAGAC  TGATTTTGGT  560
FWA 2-7.ab1     ATATTGATAA  TCAGATTTTT  GTTGTTTTTT  TTAGTTAAAA  ATTAGATTTT  TTTATATTTT  GTTTAGAGAA  TGATTTTGGT  560
FWA 2-8.ab1     ATATTGATAA  TTAGATTTTT  GTTGTTTTTT  TTAGTTAAAA  ATTAGATTTT  TTTATATTTT  GTTTAGAGAA  TGATTTTGGT  560
FWA 2-9.ab1     ATATTGATAA  TTAGATTTTT  GTTGTTTTTT  TTAGTTATAA  ATTAGATTTT  TTTATATTTT  GTTTAGAGAA  TGATTTTGGT  560
FWA 2-10.ab1    ATATTGATAA  TTAGATTTTT  GTTGTTTTTT  TTAGTTAAAA  ATTAGATTTT  TTTATATTTT  GTTTAGAGAA  TGATTTTGGT  560
FWA 2-11.ab1    ATATTGATAA  TTAGATTTTT  GTTGTTTTTT  TTAGTTAAAA  ATTAGATTTT  TTTATATTTT  GTTTAGAGAA  TGATTTTGGT  560
FWA 2-12.ab1    ATATTGATAA  TTAGATTTTT  GTTGTTTTTT  TTAGTTAAAA  ATCAGATTTT  TTTATATTTT  GTTTAGAGAA  TGATTTTGGT  559
FWA 2-13.ab1    ATATTGATAA  TCAGATTTTT  GTTGTTTTTT  TTAGTTAAAA  ATTAGATTTT  TTTATATTTT  GTTTAGAGAA  TGATTTTGGT  560
FWA 2-14.ab1    ATACTGATAA  TTAGATTTCT  GTTGTTTTTT  TTAGTTAAAA  ATTAGATTTT  TTTATATTTT  GTTTAGAGAA  TGATTTTGGT  560
FWA 2-15.ab1    ATATTGATAA  TTAGATTTTT  GTTGTTTTTT  TTAGTTAAAA  ATTAGATTTT  TTTATATTTT  GTTTAGAGAA  TGATTTTGGT  560
FWA 2-16.ab1    ATACTGATAA  TTAGATTTTT  GTTGTTTTTT  TTAGTCAAAA  ATTAGATTTT  TTTATATTTT  GTTTAGAGAA  TGATTTTGGT  560
FWA 2-17.ab1    ATACTGATAA  TTAGATTTTT  GTTGTTTTTT  TTAGTTAAAA  ATTAGATTTT  TTTATATTTT  GTTTAGAGAA  TGATTTTGGT  559
FWA 2-18.ab1    ATATTGATAA  TTAGATTTTT  GTTGTTTTTT  TTAGTTAAAA  ATTAGATTTT  TTTATATTTT  GTTTAGAGAA  TGATTTTGGT  559
FWA 2-19.ab1    ATATTGATAA  TTAGATTTTT  GTTGTTTTTT  T-AGTTAAAA  ATTAGATTTT  TTTATATTTT  GTTTAGAGAA  TGATTTTGGT  559
FWA 2-20.ab1    ATATTGATAA  TTAGATTTTT  GTTGTTTTTT  TTAGTTAAAA  ATTAGATTTT  TTTATATTTT  GTTTAGAGAA  TGATTTTGGT  560
FWA 2-21.ab1    ATATTGATAA  TTAGATTTTT  GTTATTTCTT  TTAGTTAAGA  ATTAGATTTT  TTTATATTTT  GTTTAGAGAA  TGATTTTGGT  560
FWA 2-22.ab1    ATACTGATAA  TTAGATTTTT  GTTGTTTTTT  TTAGTTAAAA  ATTAGATTTT  TTTATATTTT  GTTTAGAGAA  TGATTTTGGT  559
FWA 2-23.ab1    ATACTGATAA  TTAGATTTTT  GTTGTTTTTT  TTAGTTAAAA  ATTAGATTTT  TTTATATTTT  GTTTAGAGAA  TGATTTTGGT  559
FWA 2-24.ab1    ATATTGATAA  TTAGATTTTT  GTTGTTTTTT  T-AGTTAAAA  ATTAGATTTT  TTTATATTTT  GTTTAGAGAA  TGATTTTGGT  558

fwa repeats     TAAGGGCGAT  T--  569
FWA 2-1.ab1     TAAGGGCGAA  TTC  573
FWA 2-2.ab1     TAAGGGCGAA  TTC  572
FWA 2-3.ab1     TAAGGGCGAA  TTC  573
FWA 2-4.ab1     TAAGGGCGAA  TTC  573
FWA 2-5.ab1     TAAGGGCGAA  TTC  572
FWA 2-6.ab1     TAAGGGCGAA  TTC  573
FWA 2-7.ab1     TAAGGGCGAA  TTC  573
FWA 2-8.ab1     TAAGGGCGAA  TTC  573
FWA 2-9.ab1     TAAGGGCGAA  TTC  573
FWA 2-10.ab1    TAAGGGCGAA  TTC  573
FWA 2-11.ab1    TAAGGGCGAA  TTC  573
FWA 2-12.ab1    TAAGGGCGAA  TTC  572
FWA 2-13.ab1    TAAGGGCGAA  TTC  573
FWA 2-14.ab1    TAAGGGCGAA  TTC  573
FWA 2-15.ab1    TAAGGGCGAA  TTC  573
FWA 2-16.ab1    TAAGGGCGAA  TTC  573
FWA 2-17.ab1    TAAGGGCGAA  TTC  572
FWA 2-18.ab1    TAAGGGCGAA  TTC  572
FWA 2-19.ab1    TAAGGGCGAA  TTC  572
FWA 2-20.ab1    TAAGGGCGAA  TTC  573
FWA 2-21.ab1    TAAGGGCGAA  TTC  573
FWA 2-22.ab1    TAAGGGCGAA  TTC  572
FWA 2-23.ab1    TAAGGGCGAA  TTC  572
FWA 2-24.ab1    TAAGGGCGAA  TTC  571
```

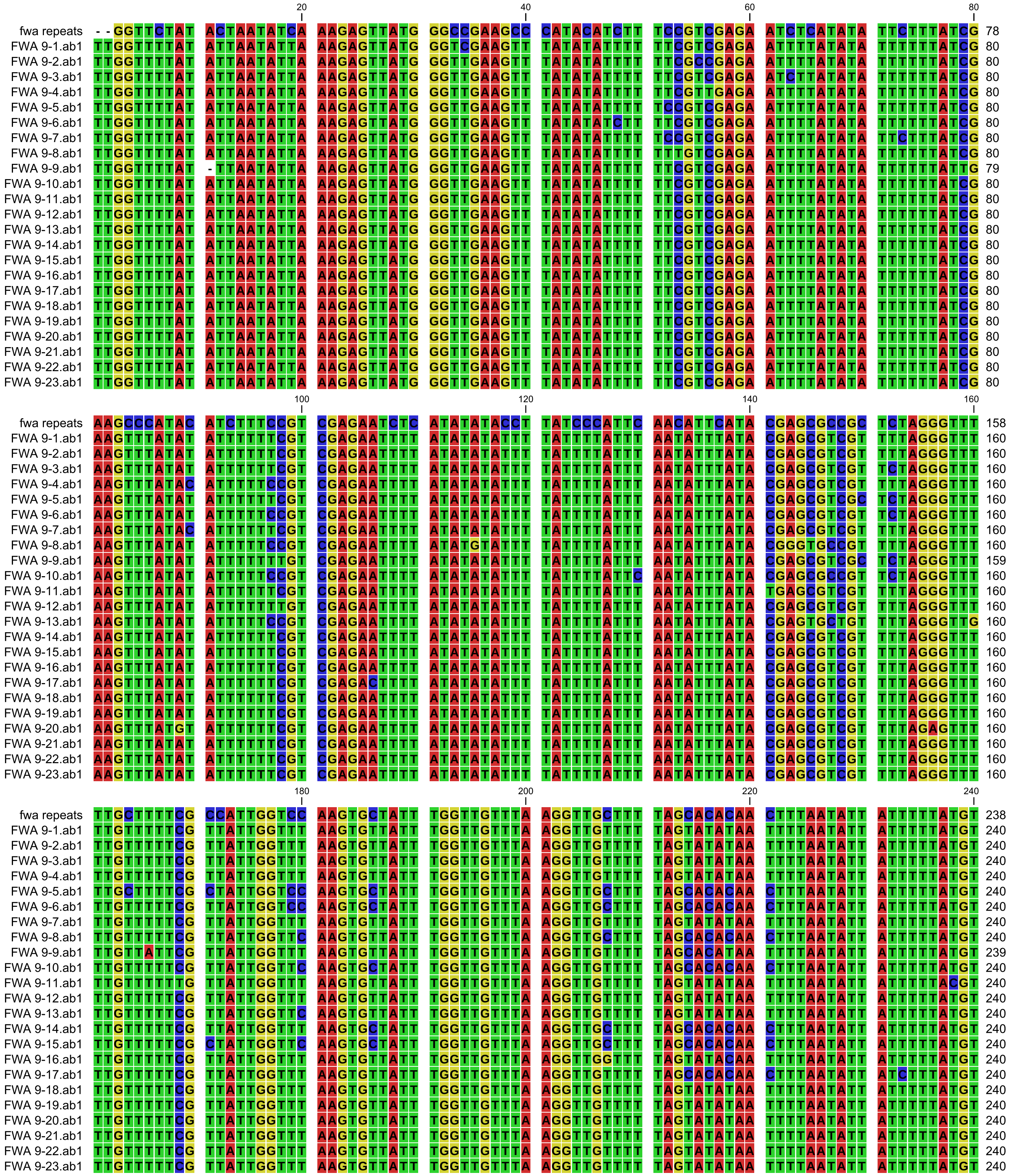
20
40
60
80
100
120
140
160
180
200
220
240
fwa repeats
FWA 9-1.ab1
FWA 9-2.ab1
FWA 9-3.ab1
FWA 9-4.ab1
FWA 9-5.ab1
FWA 9-6.ab1
FWA 9-7.ab1
FWA 9-8.ab1
FWA 9-9.ab1
FWA 9-10.ab1
FWA 9-11.ab1
FWA 9-12.ab1
FWA 9-13.ab1
FWA 9-14.ab1
FWA 9-15.ab1
FWA 9-16.ab1
FWA 9-17.ab1
FWA 9-18.ab1
FWA 9-19.ab1
FWA 9-20.ab1
FWA 9-21.ab1
FWA 9-22.ab1
FWA 9-23.ab1

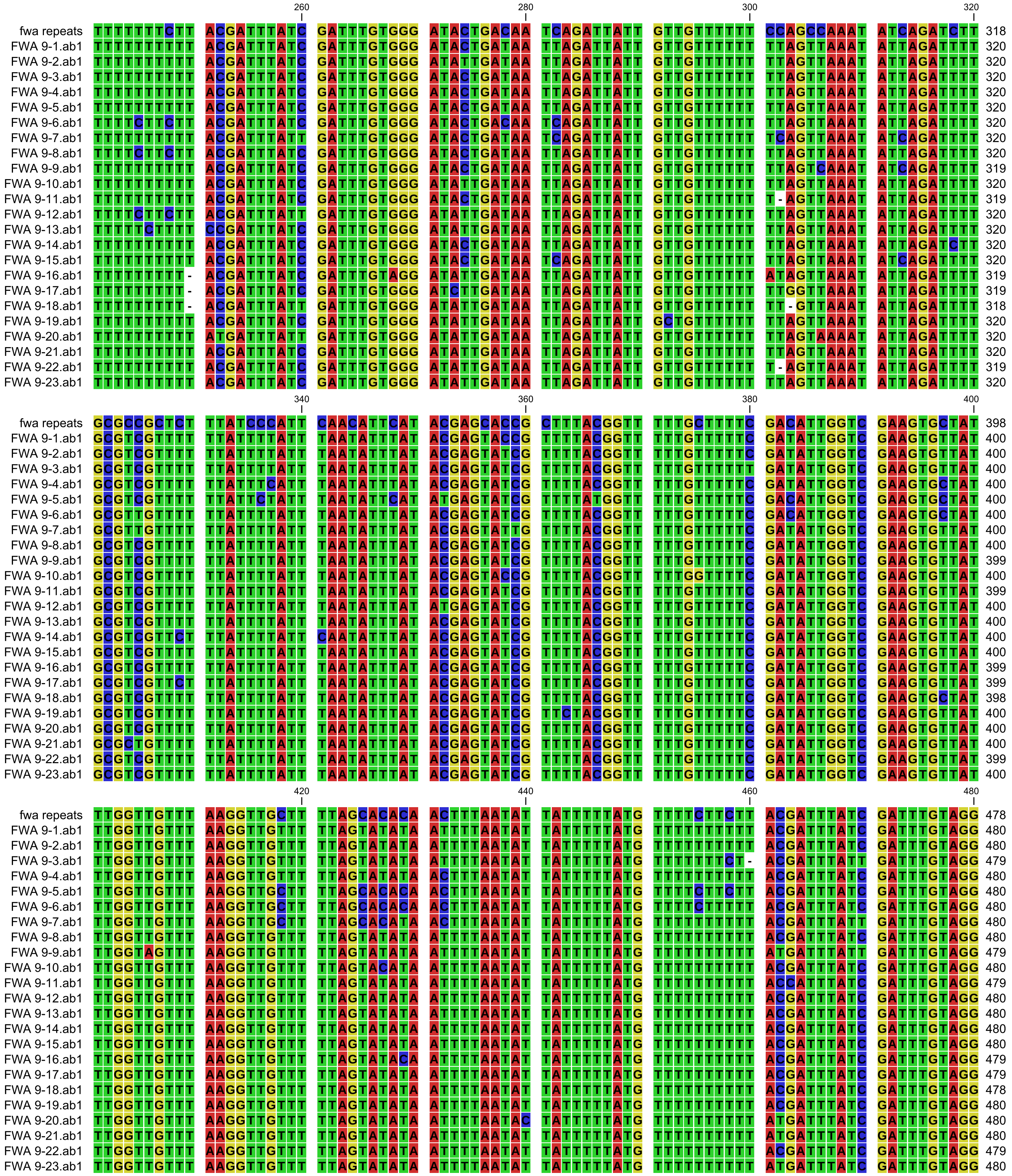

```
                                       500                 520                 540                 560
                                         |                   |                   |                   |
fwa repeats     ATACTGACAA TCAGATTTTT GTTGTTTTTT TCAGCCAAAA ATCAGATTTT TTTACACTTT GTTTAGAGAA TGATTTTGGT 558
FWA 9-1.ab1     ATACTGATAA TTAGATTTTT GTTGTTTTTT TTAGTTAAAA ATTAGATTTT TTTATATTTT GTTTAGAGAA TGATTTTGGT 560
FWA 9-2.ab1     ATATTGATAA TTAGATTTTT GTTGTTTTTT TTAGTTAAAA ATTAGATTTT TTTATATTTT GTTTAGAGAA TGATTTTGGT 560
FWA 9-3.ab1     ATACTGATAA TTAGATTTTT GTTGTTTTTT TTAGTTAAAA ATTAGATTTT TTTATATTTT GTTTAGAGAA TGATTTTGGT 559
FWA 9-4.ab1     ATATTGATAA TTAGATTTTT GTTGTTTTTT TTAGTTAAAA ATTAGATTTT TTTATATTTT GTTTAGAGAA TGATTTTGGT 560
FWA 9-5.ab1     ATACTGATAA TCAGATTTTT GTTGTTTTTT TTAGTTAAAA ATTAGATTTT TTTATATTTT GTTTAGAGAA TGATTTTGGT 560
FWA 9-6.ab1     ATACTGACAA TCAGATTTTT GTTGTTTTTT TTAGTTAAAA ATTAGATTTT TTTATATTTT GTTTAGAGAA TGATTTTGGT 560
FWA 9-7.ab1     ATATTGATAA TTAGATTTTT GTTGTTTTTT TTAGTTAAAA ATTAGATTTT TTTATATTTT GTTTAGAGAA TGATTTTGGT 560
FWA 9-8.ab1     ATATTGATAA TTAGATTTTT GTTGTTTTTT TTAGTTAAAA ATTAGATTTT TTTATATTTT GTTTAGAGAA TGATTTTGGT 560
FWA 9-9.ab1     ATATTGATAA TTAGATTTTT GTTGTTTTTT TTAGTTAAAA ATTAGATTTT TTTATATTTT GTTTAGAGAA TGATTTTGGT 559
FWA 9-10.ab1    ATATTGATAA TTAGATTTTT GTTGTTTTTT TCAGTTAAAA ATTAGATTTT TTTATATTTT GTTTAGAGAA TGATTTTGGT 560
FWA 9-11.ab1    ATACTGATAA TTAGATTTTT GTTGTTTTTT TTAGTTAAAA ATTAGATTTT TTTATATTTT GTTTAGAGAA TGATTTTGGT 559
FWA 9-12.ab1    ATATTGATAA TTAGATTTTT GTTGTTTTTT TTAGTTAAAA ATTAGATTTT TTTATATTTT GTTTAGAGAA TGATTTTGGT 560
FWA 9-13.ab1    ATATTGATAA TTAGATTTTT GTTGTTTTTT TCAGTTAAAA ATTAGATTTT TTTATATTTT GTTTAGAGAA TGATTTTGGT 560
FWA 9-14.ab1    ATACTGATAA TTAGATTTTT GTTGTTTTTT TTAGTTAAAA ATTAGATTTT TTTATATTTT GTTTAGAGAA TGATTTTGGT 560
FWA 9-15.ab1    ATACTGATAA TCAGATTTTT GTTGTTTTTT TTAGTTAAAA ATTAGATTTT TTTATATTTT GTTTAGAGAA TGATTTTGGT 560
FWA 9-16.ab1    ATATTGATAA TTAGATTTTT GTTGTTTTTT TTAGTTAAAA ATTAGATTTT TTTATATTTT GTTTAGAGAA TGATTTTGGT 559
FWA 9-17.ab1    ATATTGATAA TTAGATTTTT GTTGTTTTTT TTAGTTAAAA ATTAGATTTT TTTATATTTT GTTTAGAGAA TGATTTTGGT 559
FWA 9-18.ab1    ATATTGATAA TTAGATTTTT GTTGTTTTTT TTAGTTAAAA ATTAGATTTT TTTATATTTT GTTTAGAGAA TGATTTTGGT 558
FWA 9-19.ab1    ATACTGATAA TTAGATTTTT GTTGTTTTTT TTAGTTAAAA ATTAGATTTT TTTATATTTT GTTTAGAGAA TGATTTTGGT 560
FWA 9-20.ab1    ATATTGATAA TTAGATTTTT GTTGTTTTTT TTAGTTAAAA ATTAGATTTT TTTATATTTT GTTTAGGTTA TGATTTTGGT 560
FWA 9-21.ab1    ATATTGATAA TTAGATTTTT GTTGTTTTTT TTAGTTAAAA ATTAGATTTT TTTATATTTT GTTTAGAGAA TGATTTTGGT 560
FWA 9-22.ab1    ATACTGATAA TTAGATTTTT GTTGTTTTTT --AGTTAAAA ATTAGATTTT TTTATATTTT GTTTAGAGAA TGATTTTGGT 557
FWA 9-23.ab1    ATATTGATAA TTAGATTTTT GTTGTTTTTT TTAGTTAAAA ATTAGATTTT TTTATATTTT GTTTAGAGAA TGATTTTGGT 560

fwa repeats     TAAGGGCGAT T 569
FWA 9-1.ab1     TAAGGGCGAA T 571
FWA 9-2.ab1     TAAGGGCGAA T 571
FWA 9-3.ab1     TAAGGGCGAA T 570
FWA 9-4.ab1     TAAGGGCGAA T 571
FWA 9-5.ab1     TAAGGGCGAA T 571
FWA 9-6.ab1     TAAGGGCGAA T 571
FWA 9-7.ab1     TAAGGGCGAA T 571
FWA 9-8.ab1     TAAGGGCGAA T 571
FWA 9-9.ab1     TAAGGGCGAA T 570
FWA 9-10.ab1    TAAGGGCGAA T 571
FWA 9-11.ab1    TAAGGGCGAA T 570
FWA 9-12.ab1    TAAGGGCGAA T 571
FWA 9-13.ab1    TAAGGGCGAA T 571
FWA 9-14.ab1    TAAGGGCGAA T 571
FWA 9-15.ab1    TAAGGGCGAA T 571
FWA 9-16.ab1    TAAGGGCGAA T 570
FWA 9-17.ab1    TAAGGGCGAA T 570
FWA 9-18.ab1    TAAGGGCGAA T 569
FWA 9-19.ab1    TAAGGGCGAA T 571
FWA 9-20.ab1    TAAGGGCGAA T 571
FWA 9-21.ab1    TAAGGGCGAA T 571
FWA 9-22.ab1    TAAGGGCGAA T 568
FWA 9-23.ab1    TAAGGGCGAA T 571
```

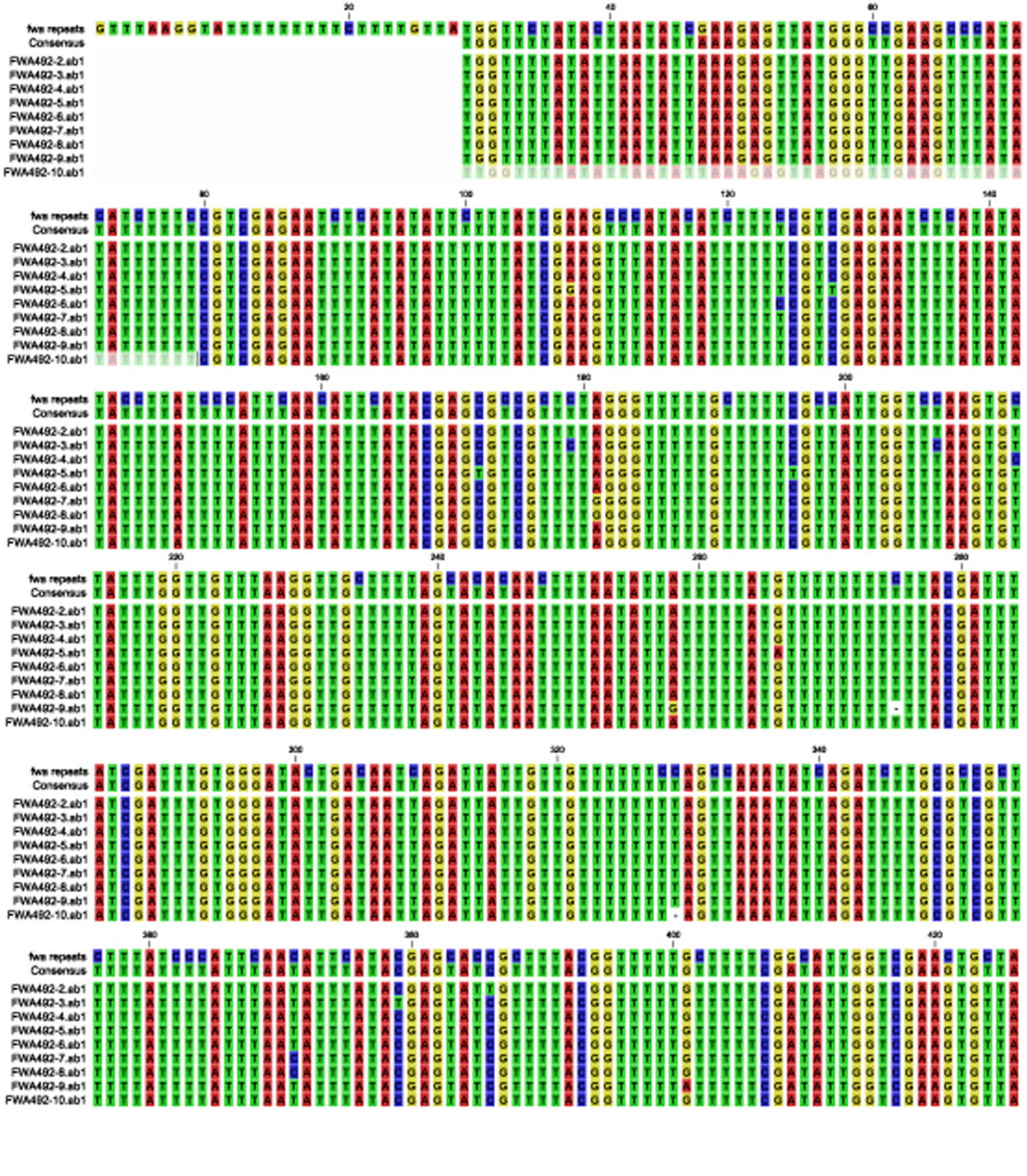
fwa repeats
Consensus
FWA492-2.ab1
FWA492-3.ab1
FWA492-4.ab1
FWA492-5.ab1
FWA492-6.ab1
FWA492-7.ab1
FWA492-8.ab1
FWA492-9.ab1
FWA492-10.ab1

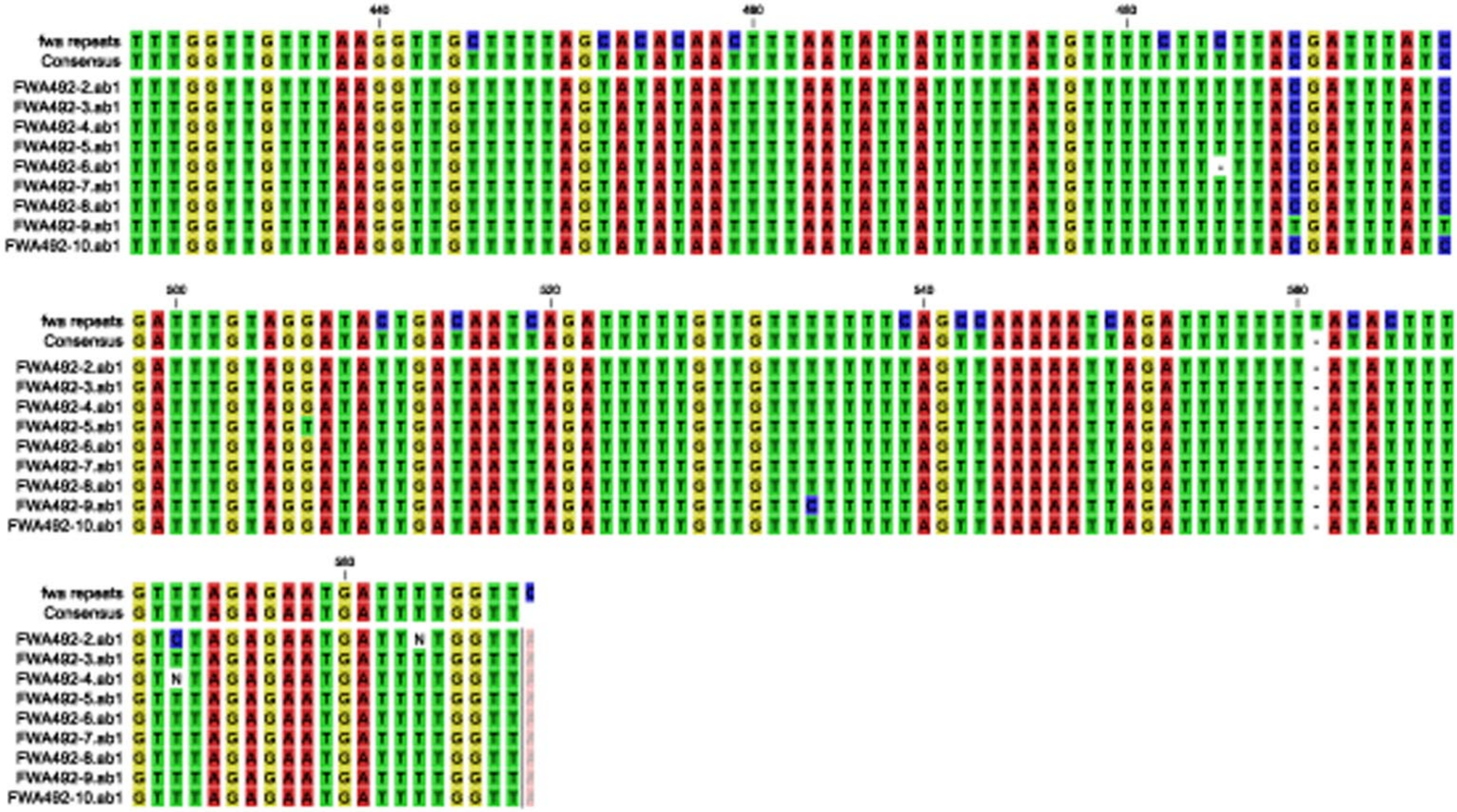
fws repeats
Consensus
FWA492-2.ab1
FWA492-3.ab1
FWA492-4.ab1
FWA492-5.ab1
FWA492-6.ab1
FWA492-7.ab1
FWA492-8.ab1
FWA492-9.ab1
FWA492-10.ab1

		20		40		60		80	
fwa repeats	T-GGTTCTAT	ACTAATATCG	AAGAGTTATG	GGCCGAAGCC	CATACATCTT	TCCGTCGAGA	ATCTCATATA	TTCTTTATCG	79
FWA 2-1L.ab1	TTGGTTTTAT	ATTAATATTA	AAGAGTTATG	GGTTGAAGTT	TATATATTTT	TTTGTTGAGA	ATTTTATATA	TTTTTTATTG	80
FWA 2-2L.ab1	TTGGTTTTAT	ATTAATATTA	AAGAGTTATG	GGTTGAAGTT	TATATATTTT	TTTGTTGAGA	ATTTTATATA	TTTTTTATTG	80
FWA 2-3L.ab1	TTGGTTTTAT	ATTAATATTA	AAGAGTTATG	GGTTGAAGTT	TATATATTTT	TTTGTTGAGA	ATTTTATATA	TTTTTTATTG	80
FWA 2-4L.ab1	TTGGTTTTAT	ATTAATATTA	AAGAGTTATG	GGTTGAAGTT	TATATATTTT	TTTGTTGAGA	ATTTTATATA	TTTTTTATTG	80
FWA 2-5L.ab1	TTGGTTTTAT	ATTAATATTA	AAGAGTTATG	GGTTGAAGTT	TATATATTTT	TTTGTTGAGA	ATTTTATATA	TTTTTTATTG	80
FWA 2-6L.ab1	TTGGTTTTAT	ATTAATATTA	AAGAGTTATG	GGTTGAAGTT	TATATATTTT	TTTGTCGAGA	ATTTTATATA	TTTTTTATCG	80
FWA 2-7L.ab1	TTGGTTTTAT	ATTAATATTA	AAGAGTTATG	GGTTGAAGTT	TATATGTTTT	TTTGTTGAGA	ATTTTATATA	TTTTTTATTG	80
FWA 2-8L.ab1	TTGGTTTTAT	ATTAATATTA	AAGAGTTATG	GGTTGAAGTT	TATATATTTT	TTTGTTGAGA	ATTTTATATA	TTTTTTATTG	80
FWA 2-9L.ab1	TTGGTTTTAT	ATTAATATTA	AAGAGTTATG	GGTTGAAGTT	TATATATTTT	TTTGTTGAGA	ATTTTATATA	TTTTTTATTG	80
FWA 2-10L.ab1	TTGGTTTTAT	ATTAATATTA	AAGAGTTATG	GGTTGAAGTT	TATATATTTT	TTCGTCGAGA	ATTTTATATA	TTTTTTATTG	80
FWA 2-11L.ab1	TTGGTTTTAT	ATTAATATTA	AAGAGTTATG	GGTTGAAGTT	TATATATTTT	TTTGTTGAGA	ATTTTATATA	TTTTTTATTG	80
FWA 2-12L.ab1	TTGGTTTTAT	ATTAATATTA	AAGAGTTATG	GGTTGAAGTT	TATATATTTT	TTTGTTGAGA	ATTTTATATA	TTTTTTATTG	80

		100		120		140		160	
fwa repeats	AAGCCCATAC	ATCTTTCCGT	CGAGAATCTC	ATATATACCT	TATCCCATTC	AACATTCATA	CGAGCGCCGC	TCTAGGGTTT	159
FWA 2-1L.ab1	AAGCTTATAT	ATTTTTTTGT	TGAGAATTTT	ATATATATTT	TATTTTATTT	AATATTTATA	TGAGTGTTGT	TTTAGGGTTT	160
FWA 2-2L.ab1	AAGTTTATAT	ATTTTTTTGT	TGAGAATTTT	ATATATATTT	TATTTTATTT	AATATTTATA	TGAGTGTTGT	TTTAGGGTTT	160
FWA 2-3L.ab1	AAGTTTATAT	ATTTTTTTGT	TGAGAATTTT	ATATATATTT	TATTTTATTT	AATATTTATA	TGAGTGTTGT	TTTAGGGTTT	160
FWA 2-4L.ab1	AAGTTTATAT	ATTTTTTTGT	TGAGAATTTT	ATATATATTT	TATTTTATTT	AATATTTATA	TGAGTGTTGT	TTTAGGGTTT	160
FWA 2-5L.ab1	AAGTTTATAT	ATTTTTTTGT	TGAGAATTTT	ATATATATTT	TATTTTATTT	AATATTTATA	TGAGTGTTGT	TTTAGGGTTT	160
FWA 2-6L.ab1	AAGTTTATAT	ATTTTTTCGT	TGAGAATTTT	ATATATATTT	TATTTTATTT	AATATTTATA	CGAGTGTCGT	TTTAGGGTTT	160
FWA 2-7L.ab1	AAGTTTATAT	ATTTTTTTGT	TGAGAATTTT	ATATATATTT	TATTTTATTT	AATATTTATA	TGAGTGTTGT	TTTAGGGTTT	160
FWA 2-8L.ab1	AAGTTTATAT	ATTTTTTTGT	TGAGAATTTT	ATATATATTT	TATTTTATTT	AATATTTATA	TGAGTGTTGT	TTTAGGGTTT	160
FWA 2-9L.ab1	AAGTTTATAT	ATTTTTTTGT	TGAGAATTTT	ATATATATTT	TATTTTATTT	AATATTTATA	TGAGTGTTGT	TTTAGGGTTT	160
FWA 2-10L.ab1	AAGTTTATAT	ATTTTTTCGT	TGAGAATTTT	ATATATATTT	TATTTTATTT	AATATTTATA	TGAGTGTTGT	TTTAGGGTTT	160
FWA 2-11L.ab1	AAGTTTATAT	ATTTTTTTGT	TGAGAATTTT	ATATATATTT	TATTTTATTT	AATATTTATA	TGAGTGTTGT	TTTAGGGTTT	160
FWA 2-12L.ab1	AAGTTTATAT	ATTTTTTCGT	CGAGAATTTT	ATATATATTT	TATTTTATTT	AATATTTATA	CGAGCGTCGT	TTTAGGGTTT	160

		180		200		220		240	
fwa repeats	TTGCTTTTCG	CCATTGGTCC	--AAGTGCTA	TTTGGTTGTT	TAAGGTTGCT	TTTAGCACAC	AACTTTAATA	TTATTTTTAT	237
FWA 2-1L.ab1	TTGTTTTTTG	TTATTGGTTT	--AAGTGTTA	TTTGGTTGTT	TAAGGTTGTT	TTTAGTATAT	AATTTTAATA	TTATTTTTAT	238
FWA 2-2L.ab1	TTGTTTTTTG	TTATTGGTTT	--AAGTGTTA	TTTGGTTGTA	TAAGGTTGTT	TTTAGTATAT	AATTTTAATA	TTATTTTTAT	238
FWA 2-3L.ab1	TTGTTTTTTG	TTATTGGTTT	--AAGTGTTA	TTTGGTTGTT	TAAGGTTGTT	TTTAGTATAT	AATTTTAATA	TTATTTTTAT	238
FWA 2-4L.ab1	TTGTTTTTTG	TTATTGGTTT	--AGGTGTTA	TTTGGTTGTT	TAAGGTTGTT	TTTAGTATAT	AATTTTAATA	TTATTTTTAT	238
FWA 2-5L.ab1	TTGTTTTTTG	TTATTGGTTT	--AAGTGTTA	TTTGGTTGTT	TAAGGTTGTT	TTTAGTATGT	AATTTTAATA	TTATTTTTAT	238
FWA 2-6L.ab1	TTGTTTTTCG	TTATTGGTTT	--AAGTGTTA	TTTGGTTGTT	TAAGGTTGTT	TTTAGTATAT	AATTTTAATA	TTATATTTAT	238
FWA 2-7L.ab1	TTGTTTTTTG	TTATTGGTTT	--AAGTGTTA	TTTGGTTGTT	TAAGGTTGTT	TTTAGTATAT	AATTTTAATA	TTATTTTTAT	238
FWA 2-8L.ab1	TTGTTTTTTG	TTATTGGTTT	--AAGTGTTA	TTTGGTTGTT	TAAGGTTGTT	TTTAGTATAT	AATTTTAATA	TTATTTTTAT	238
FWA 2-9L.ab1	TTGTTTTTTG	TTATTGGTTT	--AAGTGTTA	TTTGGTTGTT	TAAGGTTGTT	TTTAGTATAT	AACTTTAATA	TTATTTTTAT	238
FWA 2-10L.ab1	TTGTTTTTTG	TTATTGGTTT	--AAGTGTTA	TTTGGTTGTT	TAAGGTTGTT	TTTAGTATAT	AATTTTAATA	TTATTTTTAT	238
FWA 2-11L.ab1	TTGTTTTTTG	TTATTGGTTT	--AAGTGTTA	TTTGGTTGTT	TAAGGTTGTT	TTTAGTATAT	AATTTTAATA	TTATTTTTAT	238
FWA 2-12L.ab1	TTGTTTTTTG	TTATTGGTTT	--AAGTGTTA	TTTGGTTGTT	TAAGGTTGTT	TTTAGTATAT	AATTTTAATA	TTATTTTTAT	238

		260		280		300		320	
fwa repeats	GTTTTTTTTC	TTACGATTTA	TCGATTTGTG	GGATACTGAC	AATCAGATTA	TTGTTGTTTT	TTCCAGCCAA	ATATCAGA--	315
FWA 2-1L.ab1	GTTTTTTTTT	TTATGATTTA	TTGATTTGTG	GGATATTGAT	AATTAGATTA	TTGTTGTTTT	TTTTAGTTAA	ATATTAGAAT	318
FWA 2-2L.ab1	GTTTTTTTTT	TTATGATTTA	TTGATTTGTG	GGATATTGAT	AATTAGATTA	TTGTTGTTTT	TTTTAGTTAA	ATATTAGAAT	318
FWA 2-3L.ab1	GTTTTTTTTT	TTATGACTTA	TTGATTTGTG	GGATATTGAT	AATTAGATTA	TTGTTGTTTT	TTTTAGTTAA	ATATTAGAAT	318
FWA 2-4L.ab1	GTTTTTTTTT	TTATGATTTA	TTGATTTGTG	GGATATTGAT	AATTAGATTA	TTGTTGTTTT	TTTTAGTTAA	ATATTAGAAT	318
FWA 2-5L.ab1	GTTTTTTTTT	TTATGATTTA	TTGATTTGTG	GGATATTGAT	AATTAGATTA	TTGTTGTTTT	TTTTAGTTAA	ATGTTAGAAT	318
FWA 2-6L.ab1	GTTTTTTTTT	TTATGATTTA	TTGATTTGTG	GGATATTGAT	AATTAGATTA	TTGTTGTTTT	TTTTAGTTAA	ATATTAGAAT	318
FWA 2-7L.ab1	GTTTTTTTTT	TTATGATTTA	TTGATTTGTG	GGATATTGAT	AATTAGATTA	TTGTTGTTTT	TTTTAGTTAA	ATATTAGAAT	318
FWA 2-8L.ab1	GTTTTTTTTT	TTATGATTTA	TTGATTTGTG	GGATATTGAT	AATTAGATTA	TTGTTGTTTT	TTTTAGTTAA	ATATTAGAAT	318
FWA 2-9L.ab1	GTTTTTTTTT	T-ATGATTTA	TTGATTTGTG	GGATATTGAT	AATTAGATTA	TTGTTGTTTT	TTTTAGTTAA	ATATTAGAAT	317
FWA 2-10L.ab1	GTTTTTTTTT	TTACGATTTA	TTGATTTGTG	GGATATTGAT	AATTAGATTA	TTGTTGTTTT	TTTTAGTTAA	ATATTAGAAT	318
FWA 2-11L.ab1	GTTTTTTTTT	T-ATGATTTA	TTGATTTGTG	GGATATTGAT	AATTAGATTA	TTGTTGTTTT	TTTTAGTTAA	ATATTAGAAT	317
FWA 2-12L.ab1	GTTTTTTTTT	TTATGATTTA	TTGATTTGTG	GGATATTGAT	AATTAGATTA	TTGTTGTTTT	TTTTAGTTAA	ATATTAGAAT	318

		340		360		380		400	
fwa repeats	TCTTGCGCCG	CTCTTTATCC	CATTCAACAT	TCATACGAGC	ACCGCTTTAC	GGTTTTTGCT	TTTCGGCATT	GGTCGAACTG	395
FWA 2-1L.ab1	TTTTGTGTTG	TTTTTTATTT	TATTTAATAT	TTATATGAGT	ATTGTTTTAT	GGTTTTTGTT	TTTTGGTATT	GGTTGAATTG	398
FWA 2-2L.ab1	TTTTGTGTTG	TTTTTTATTT	TATTTAATAT	TTATATGAGT	ATTGTTTTGT	GGTTTTTGTT	TTTTGGTATT	GGTTGAATTG	398
FWA 2-3L.ab1	TTTTGTGTTG	TTTTTTATTT	TATTTAATAT	TTATATGAGT	ATTGTTTTAT	GGTTTTTGTT	TTTTGGTATT	GGTTGAATTG	398
FWA 2-4L.ab1	TTTTGTGTTG	TTTTTTATTT	TATTTAATAT	TTATATGAGT	ATTGTTTTAT	GGTTTTTGTT	TTTTGGTATT	GGTTGAATTG	398
FWA 2-5L.ab1	TTTTGTGTTG	TTTTTTATTT	TATTTAATAT	TTATATGAGT	ATTGTTTTAT	GGTTTTTGTT	TTTTGGTATT	GGTTGAATTG	398
FWA 2-6L.ab1	TTTTGTGTTG	TTTTTTATTT	TATTTAATAT	TTATATGAGT	ATTGTTTTAT	GGTTTTTGTT	TTTTGGTATT	GGTTGAATTG	398
FWA 2-7L.ab1	TTTTGTGTTG	TTTTTTATTT	TATTTAATAT	TTATATGAGT	ATTGTTTTAT	GGTTTTTGTT	TTTTGGTATT	GGTTGAATTG	398
FWA 2-8L.ab1	TTTTGTGTTG	TTTTTTATTT	TATTTAATAT	TTATATGAGT	ATTGTTTTAT	GGTTTTTGTT	TTTTGGTATT	GGTTGAATTG	398
FWA 2-9L.ab1	TTTTGTGTTG	TTTTTTATTT	TATTTAATAT	TTATATGAGT	ATTGTTTTAT	GGTTTTTGTT	TTTTGGTATT	GGTTGAATTG	397
FWA 2-10L.ab1	TTTTGTGTTG	TTTTTTATTT	TATTTAATAT	TTATATGAGT	ATTGTTTTAT	GGTTTTTGTT	TTTTGGTATT	GGTCGAATTG	398
FWA 2-11L.ab1	TTTTGTGTTG	TTTTTTATTT	TATTTAATAT	TTATATGAGT	ATTGTTTTAT	GGTTTTTGTT	TTTTGGTATT	GGTTGAATTG	397
FWA 2-12L.ab1	TTTTGTGTTG	TTTTTTATTT	TATTTAATAT	TTATATGAGT	ATTGTTTTAT	GGTTTTTGTT	TTTTGGTATT	GGTTGAATTG	398

```
                                420                   440                   460                   480
fwa repeats    CTATTTGGTT GTTTAAGGTT GCTTTTAGCA CACAACTTTA ATATTATTTT TATGTTTTCT TCTTACGATT TATCGATTTG 475
FWA 2-1L.ab1   TTATTTGGTT GTTTAAGGTT GTTTTTAGTA TATAATTTTA ATATTATTTT TATGTTTTTT TTTTATGATT TATTGATTTG 478
FWA 2-2L.ab1   TTATTTGGTT GTTTAAGGTT GTTTTTAGTA TATAATTTTA ATATTATTTT TATGTTTTTT TTTTATGATT TATTGATTTG 478
FWA 2-3L.ab1   TTATTTGGTT GTTTAAGGTT GTTTTTAGTA TATAATTTTA ATATTATTTT TATGTTTTTT TTTTATGATT TATTGATTTG 478
FWA 2-4L.ab1   TTATTTGGTT GTTTAAGGTT GTTTTTAGTA TATAATCTTA ATATTATTTT TATGTTTTTT TTT-ATGATT TATTGATTTG 477
FWA 2-5L.ab1   TTATTTGGTT GTTTAAGGTT GTTTTTAGTA TATAATTTTA ATATTATTTT TATGTTTTTT TTTTATGATT TATTGATTTG 478
FWA 2-6L.ab1   TTATTTGGTT GTTTAAGGTT GTTTTTAGTA TATAATTTTA ATATTATTTT TATGTTTTTT TTTTATGATT TATTGATTTG 478
FWA 2-7L.ab1   TTATTTGGTT GTTTAAGGTT GTTTTTAGTA TATAATTTTA ATATTATTTT TATGTTTTTT TTTTATGATT TATTGATTTG 478
FWA 2-8L.ab1   TTATTTGGTT GTTTAAGGTT GTTTTTAGTG TATAATTTTA ATATTATTTT TATGTTTTTT TTTTATGATT TATTGATTTG 478
FWA 2-9L.ab1   TTATTTGGTT GTTTAAGGTT GTTTTTAGTA TATAATTTTA ATATTATTTT TATGTTTTTT TTTTATGATT TATTGATTTG 477
FWA 2-10L.ab1  TTATTTGGTT GTTTAAGGTT GTTTTTAGTA TATAATTTTA ATATTATTTT TATGTTTTTT TTTTATGATT TATCGATTTG 478
FWA 2-11L.ab1  TTATTTGGTT GTTTAAGGTT GGTTTTAGTA TATAGTTTTA ATATTATTTT TATGTTTTTT TTTTATGATT TATTGATTTG 477
FWA 2-12L.ab1  TTATTTGGTT GTTTAAGGTT GTTTTTAGTA TATAATTTTA ATATTATTTT TATGTTTTTT TTTTATGATT TATTGATTTG 478

                                500                   520                   540                   560
fwa repeats    TAGGATACTG ACAATCAGAT TTTTGTTGTT TTTTTCAGCC AAAAATCAGA TTTTTTTTAC ACTTTGTTTA GAGAATGATT 555
FWA 2-1L.ab1   TAGGATATTG ATAATTAGAT TTTTGTTGTT TTTTTTAGTT AAAAATTAGA TTTTTTT-AT ATTTTGTTTA GAGAATGATT 557
FWA 2-2L.ab1   TAGGATATTG ATAATTAGAT TTTTGTTGTT TTTTTTAGTT AAAAATTAGA TTTTTTT-AT ATTTTGTTTA GAGAATGATT 557
FWA 2-3L.ab1   TAGGATATTG ATAATTAGAT TTTTGTTGTT TTTTTTAGTT AAAAATTAGA TTTTTTT-AT ATTTTGTTTA GAGAATGATT 557
FWA 2-4L.ab1   TAGGATATTG ATAATTAGAT CTTTGTTGTT TTTTTTAGTT AAAAATTAGA TTTTTTT-AT ATTTTGTTTA GAGAATGATT 556
FWA 2-5L.ab1   TAGGATATTG ATAATTGGAT TTTTGTTGTT TTTTTTAGTT AAAAATTAGA TTTTTTT-AT ATTTTGTTTA GAGAATGATT 557
FWA 2-6L.ab1   TAGGATATTG ATAATTAGAT TTTTGTTGTT TTTTTTAGTT AAAAATTAGA TTTTTTT-AT ATTTTGTTTA GAGAATGATT 557
FWA 2-7L.ab1   TAGGATATTG ATAATTAGAT TTTTGTTGTT TTTTTTAGTT AAAAATTAGA TTTTTTT-AT ATTTTGTTTA GAGAATGATT 557
FWA 2-8L.ab1   TAGGATATTG ATAATTAGAT TTTTGTTGTT TTTTTTAGTT AAAAATTAGA TTTTTTT-AT ATTTTGTTTA GAGAATGATT 557
FWA 2-9L.ab1   TAGGATATTG ATAATTAGAT TTTTGTTGTT TTTTTTAGTT AAAAATTAGA TTTTTTT-AT ATTTTGTTTA GAGAATGATT 556
FWA 2-10L.ab1  TAGGATATTG ATAATTAGAT TTTTGTTGTT TTTTTTAGTT AAAAATTAGA TTTTTTT-AT ATTTTGTTTA GAGAATGATT 557
FWA 2-11L.ab1  TAGGATATTG ATAATTAGAT TTTTGTTGTT TTTTTTAGTT AAAAATTAGA TTTTTTT-AT ATTTTGTTTA GAGAATGATT 556
FWA 2-12L.ab1  TAGGATATTG ATAATTAGAT TTTTGTTGTT TTTTTTAGTT AAAAATTAGA TTTTTTT-AT ATTTTGTTTA GAGAATGATT 557

                                580
fwa repeats    TTGGTTCC-- -CGATTTGTC 572
FWA 2-1L.ab1   T-GGTTAAGG GCGAATTCCA 576
FWA 2-2L.ab1   TTGGTTAAGG GCGAATTCCA 577
FWA 2-3L.ab1   TTGGTTAAGG GCGAATTCCA 577
FWA 2-4L.ab1   TTGGTTAAGG GCGAATTCCA 576
FWA 2-5L.ab1   TTGGTTAAGG GCGAATTCCA 577
FWA 2-6L.ab1   TTGGTTAAGG GCGAATTCCA 577
FWA 2-7L.ab1   TTGGTTAAGG GCGAATTCCA 577
FWA 2-8L.ab1   TTGGTTAAGG GCGAATTCCA 577
FWA 2-9L.ab1   TTGGTTAAGG GCGAATTCCA 576
FWA 2-10L.ab1  TTGGTTAAGG GCGAATTCCA 577
FWA 2-11L.ab1  TTGGTTAAGG GCGAATTCCA 576
FWA 2-12L.ab1  TTGGTTAAGG GCGAATTCCA 577
```

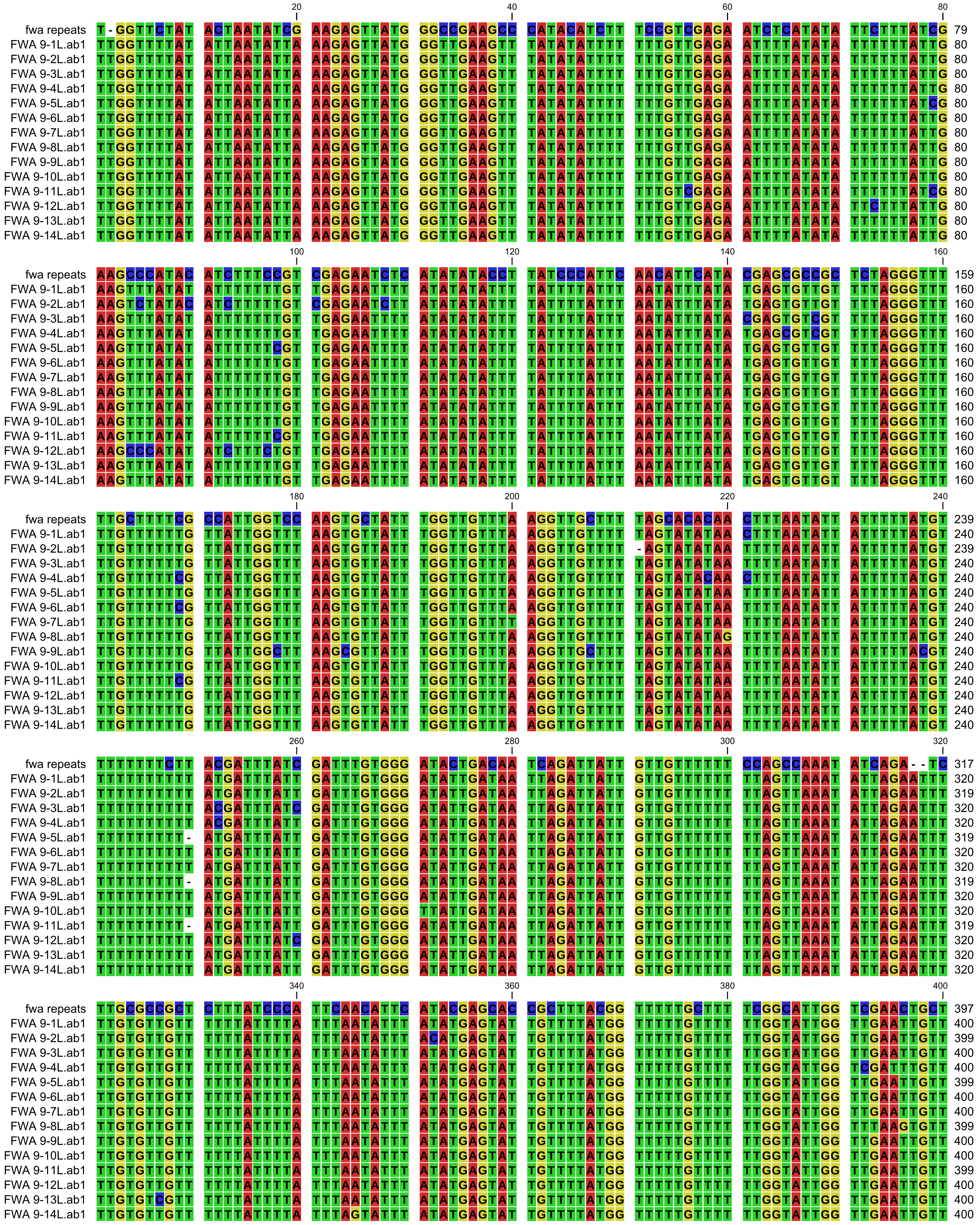
fwa repeats
FWA 9-1L.ab1
FWA 9-2L.ab1
FWA 9-3L.ab1
FWA 9-4L.ab1
FWA 9-5L.ab1
FWA 9-6L.ab1
FWA 9-7L.ab1
FWA 9-8L.ab1
FWA 9-9L.ab1
FWA 9-10L.ab1
FWA 9-11L.ab1
FWA 9-12L.ab1
FWA 9-13L.ab1
FWA 9-14L.ab1

```
                                     420                                   440                                   460                                   480
                                       |                                     |                                     |                                     |
fwa repeats      ATTTGGTTGT TTAAGGTTGC TTTTAGCACA CAACTTTAAT ATTATTTTTA TGTTTTCTTC TTACGATTTA TCGATTTGTA 477
FWA 9-1L.ab1     ATTTGGTTGT TTAAGGTTGT TTTTAGTATA TAATTTTAAT ATTATTTTTA TGTTTTTTTT TTATGATTTA TTGATTTGTA 480
FWA 9-2L.ab1     ATTTGGTTGT TTAAGGTTGT TTTTAGTAAA TAATTTTAAT ATTATTTTTA TGTTTTTTTT TTATGATTTA TTGATTTGTA 479
FWA 9-3L.ab1     ATTTGGTTGT TTAAGGTTGC TTTTAGTATA CAATTTTAAT ATTATTTTTA TGTTTTCTTT TTATGATTTA TTGATTTGTA 480
FWA 9-4L.ab1     ATTTGGTTGT TTAAGGTTGT TTTTAGTATA TAATTTTAAT ATTATTTTTA TGTTTTTTTT TTATGATTTA TTGATTTGTA 480
FWA 9-5L.ab1     ATTTGGTTGT TTAAGGTTGT TTTTAGTATA TAATTTTAAT ATTATTTTTA TGTTTTTTTT TTATGATTTA TTGATTTGTA 479
FWA 9-6L.ab1     ATTTGGTTGT TTAAGGTTGT TTTTAGTATA TAATTTTAAT ATTATTTTTA TGTTTTTTTT TTATGATTTA TTGATTTGTA 480
FWA 9-7L.ab1     ATTTGGTTGT TTAAGGTTGT TTTTAGTATA TAATTTTAAT ATTATTTTTA TGTTTTTTTT TTATGATTTA TTGATTTGTA 480
FWA 9-8L.ab1     ATTTGGTTGT TTAAGGTTGT TTTTAGTATA TAATTTTAAT ATTATTTTTA TGTTTTTTTT TTGTGATTTA TTGATTTGTA 479
FWA 9-9L.ab1     ATTTGGTTGT TTAAGGTTGT TTTTAGTATA TAATTTTAAT ATTATTTTTA TGTTTTTTTT TTATGATTTA TTGATTTGTA 480
FWA 9-10L.ab1    ATTTGGTTGT TTAAGGTTGT TTTTAGTATA TAATTTTAAT ATTATTTTTA TGTTTTTTTT T-ATGATTTA TTGATTTGTA 479
FWA 9-11L.ab1    ATTTGGTTGT TTAAGGTTGT TTTTAGTATA TAATTTTAAT ATTATTTTTA TGTTTTTTTT TTATGATTTA TTGATTTGTA 479
FWA 9-12L.ab1    ATTTGGTTGT TTAAGGTTGT TTTTAGTATA TAATTTTAAT ATTATTTTTA TGTTTTTTTT TTATGATTTA TTGATTTGTA 480
FWA 9-13L.ab1    ATTTGGTTGT TTAAGGTTGT TTTTAGTATA TAATTTTAAT ATTATTTTTA TGTTTTTTTT TTATGATTTA TTGATTTGTA 480
FWA 9-14L.ab1    ATTTGGTTGT TTAAGGTTGT TTTTAGTATA TAATTTTAAT ATTATTTTTA TGTTTTTTTT T-ATGATTTA TTGATTTGTA 479

                                     500                                   520                                   540                                   560
                                       |                                     |                                     |                                     |
fwa repeats      GGATACTGAC AATCAGATTT TTGTTGTTTT TTTCAGCCAA AAATCAGATT TTTTTTACAC TTTGTTTAGA GAATGATTTT 557
FWA 9-1L.ab1     GGATATTGAT AATTAGATTT TTGTTGTTTT TTTTATTTAA AAATTAGATT TTTTT-ATAT TTTGTTTAGA GAATGATTTT 559
FWA 9-2L.ab1     GGATATTGAT AATTAGATTT TTGTTGTTTT TTTTAGTTAA AAATTAGATT TTTTT-ATAT TTTGTTTAGA GAATGATTTT 558
FWA 9-3L.ab1     GGATATTGAT AATTAGATTT TTGTTGTTTT TTT-AGTTAA AAATTAGATT TTTTT-ATAT TTTGTTTAGA GAATGATTTT 558
FWA 9-4L.ab1     GGATATTGAT AATTAGATTT TTGTTGTTTT TTT-AGTTAA AAATTAGATT TTTTT-ATAT TTTGTTTAGA GAATGATTTT 558
FWA 9-5L.ab1     GGATATTGAT AATTAGATTT TTGTTGTTTT TTTTAGTTAA AAATTAGATT TTTTT-ATAT TTTGTTTAGA GAATGATTTT 558
FWA 9-6L.ab1     GGATATTGAT AATTAGATTT TTGTTGTTTT TTTTAGTTAA AGATTAGATT TTTTT-ATAT TTTGTTTAGA GAATGATTTT 559
FWA 9-7L.ab1     GGATATTGAT AATTAGATTT TTGTTGTTTT TTTTAGTTAA AAATTAGATT TTTTT-ATAT TTTGTTTAGA GAATGATTTT 559
FWA 9-8L.ab1     GGATATTGAT AATTAGATTT TTGTTGTTTT TTTTAGTTAA AAATTAGATT TTTTT-ATAT TTTGTTTAGA GAATGATTTT 558
FWA 9-9L.ab1     GGATATTGAT AATTAGATTT TTGTTGTTTT TTTTAGTTAA AAATTAGATT TTTTT-ATAT TTTGTTTAGA GAATGATTTT 559
FWA 9-10L.ab1    GGATATTGAT AATTAGATTT TTGTTGTTTT TTTTAGTTAA AAATTAGATT TTTTT-ATAT TTTGTTTAGA GAATGATTTT 558
FWA 9-11L.ab1    GGATATTGAT AATTAGATTT TTGTTGTTTT TTTTAGTTAA AAATTAGATT TTTTT-ATAT TTTGTTTAGA GAATGATTTT 558
FWA 9-12L.ab1    GGATATTGAT AATTAGATTT TTGTTGTTTT TTTTAGTTAA AAATTAGATT TTTTT-ATAT TTTGTTTAGA GAATGATTTT 559
FWA 9-13L.ab1    GGATATTGAT AATTAGATTT TTGTTGTTTT TTTTAGTTAA AAATTAGATT TTTTT-ATAT TTTGTTTAGA GAATGATTTT 559
FWA 9-14L.ab1    GGATATTGAT AATTAGATTT TTGTTGTTTT TTTTAGTTAA AAATTAGATT TTTTT-ATAT TTTGTTTAGA GAATGATTTT 558

fwa repeats      GGTTCC--- 563
FWA 9-1L.ab1     GGTTAAGGG 568
FWA 9-2L.ab1     GGTTAAGGG 567
FWA 9-3L.ab1     GGTTAAGGG 567
FWA 9-4L.ab1     GGTTAAGGG 567
FWA 9-5L.ab1     GGTTAAGGG 567
FWA 9-6L.ab1     GGTTAAGGG 568
FWA 9-7L.ab1     GGTTAAGGG 568
FWA 9-8L.ab1     GGTTAAGGG 567
FWA 9-9L.ab1     GGTTAAGGG 568
FWA 9-10L.ab1    GGTTAAGGG 567
FWA 9-11L.ab1    GGTTAAGGG 567
FWA 9-12L.ab1    GGTTAAGGG 568
FWA 9-13L.ab1    GGTTAAGGG 568
FWA 9-14L.ab1    GGTTAAGGG 567
```

```
                          20                   40                   60                   80
fwa repeats      T-GGTTCTAT ACTAATATCG AAGAGTTATG GGCCGAAGCC CATACATCTT TCCGTCGAGA ATCTCATATA TTCTTTATCG 79
FWA 92-1a.ab1    TTGGTTTTAT ATTAATATTA AAGAGTTATG GGTTGAAGTT TATATATTTT TTTGTTGAGA ATTTTATATA TTTTTTATTG 80
FWA 92-2a.ab1    TTGGTTTTAT ATTAATATTA AAGAGTTATG GGTTGAAGTT TATATATTTT TTTGTTGAGA ATTTTATATA TTTTTTATTG 80
FWA 92-3a.ab1    TTGGTTTTAT ATTAATATTA AAGAGTTATG GGTTGAAGTT TATATATTTT TTTGTTGAGA ATTTTATATA TTTTTTATTG 80
FWA 92-4a.ab1    TTGGTTTTAT ATTAATATTA AAGAGTTATG GGTTGAAGTT TATATATTTT TTTGTTGAGA ATTTTATATA TTTTTTATTG 80
FWA 92-5a.ab1    TTGGTTTTAT ATTAATATTA AAGAGTTATG GGTTGAAGTT TATATATTTT TTTGTTGAGA ACTTTATATA TTTTTTATTG 80
FWA 92-6a.ab1    TTGGTTTTAT ATTAATATTA AAGAGTTATG GGTTGAAGTT TGTATATCTT TTTGTTGAGA ATTTTATATA TTTTTTATTG 80
FWA 92-7a.ab1    TTGGTTTTAT ATTAATATTA AAGAGTTATG GGTTGAAGTT TATATATTTT TTTGTTGAGA ATTTTATATA TTTTTTATTG 80
FWA 92-8a.ab1    TTGGTTTTAT ATTAATATTA AAGAGTTATG GGTTGAAGTT TATATATTTT TTTGTTGAGA ATTTTATATA TTTTTTATTG 80
FWA 92-10a.ab1   TTGGTTTTAT ATTAATATTA AAGAGTTATG GGTTGAAGTT TATATATTTT TTTGTTGAGA ATTTTATATA TTTTTTATTG 80
FWA 92-11a.ab1   TTGGTTTTAT ATTAATATTA AAGAGTTATG GGTTGAAGTT TATATATTTT TTTGTTGAGA ATTTTATATA TTTTTTATTG 80
FWA 92-12a.ab1   TTGGTTTTAT ATTAATATTA AAGAGTTATG GGTTGAAGTT TATATATTTT TTTGTTGAGA ATTTTATATA TTTTTTATTG 80
FWA 92-13a.ab1   TTGGTTTTAT ATTAATATTA AAGAGTTATG GGTTGAAGTT TATATATTTT TTTGTTGAGA ATTTTATATA TTTTTTATTG 80
FWA 92-14a.ab1   TTGGTTTTAT ATTAATATTA AAGAGTTATG GGTTGAAGTT TATATATTTT TTTGTTGAGA ATTTTATATA TTTTTTATTG 80
FWA 92-15a.ab1   TTGGTTTTAT ATTAATATTA AAGAGTTATG GGTTGAAGTT TATATATTTT TTTGTTGAGA ATTTTATATA TTTTTTATTG 80
FWA 92-16a.ab1   TTGGTTTTAT ATTAATATTA AAGAGTTATG GGTTGAAGTT TATATATTTT TTTGTTGAGA ATTTTATATA TTTTTTATTG 80
FWA 92-17a.ab1   TTGGTTTTAT ATTAATATTA AAGAGTTATG GGTTGAAGTT TATATATTTT TTTGTTGAGA ATTTTATATA TTTTTTATTG 80

                         100                  120                  140                  160
fwa repeats      AAGCCCATAC ATCTTTCCGT CGAGAATCTC ATATATACCT TATCCCATTC AACATTCATA CGAGCGCCGC TCTAGGGTTT 159
FWA 92-1a.ab1    AAGTTTATAT ATTTTTTTGT TGAGAATTTT ATATATATTT TATTTTATTT AATATTTATA TGAGTGTTGT TTTAGGGTTT 160
FWA 92-2a.ab1    AAGTTTATAT ATTTTTTTGT TGAGAATTTT ATATATATTT TATTTTATTT AATATTTATA TGAGTGTTGT TTTAGGGTTT 160
FWA 92-3a.ab1    AAGTTTATAT ATTTTTTTGT TGAGAATTTT ATATATATTT TATTTTATTT AATATTTATA TGAGTGTTGT TTTAGGGTTT 160
FWA 92-4a.ab1    AAGTTTATAT ATTTTTTTGT TGAGAATTTT ATATATATTT TATTTTATTT AATATTTATA TGAGTGTTGT TTTAGGGTTT 160
FWA 92-5a.ab1    AAGTTTATAT ATTTTTTTGT TGAGAATTTT ATATATATTT TATTTTATTT AATATTTATA TGAGTGTTGT TTTAGGGTTT 160
FWA 92-6a.ab1    AAGTTTACAT ATTTTTTTGT TGAGAATTTT ATATATATTT TATTTTATTT AATATTTATA TGAGTGTTGT TTTAGGGTTT 160
FWA 92-7a.ab1    AAGTTTATAT ATTTTTTTGT TGAGAATTTT ATATATATTT TATTTTATTT AATATTTATA TGAGTGTTGT TTTAGGGTTT 160
FWA 92-8a.ab1    AAGTTTATAT ATTTTTTTGT TGAGAATTTT ATATATATTT TATTTTATTT AATATTTATA TGAGTGTTGT TTTAGGGTTT 160
FWA 92-10a.ab1   AAGTTTATAT ATTTTTTTGT TGAGAATTTT ATATATATTT TATTTTATTT AATATTTATA TGAGTGTTGT TTTAGGGTTT 160
FWA 92-11a.ab1   AAGTTTATAT ATTTTTTTGT TGAGAATTTT ATATATATTT TATTTTATTT AATATTTATA TGAGTGTTGT TTTAGGGTTT 160
FWA 92-12a.ab1   AAGTTTATAT ATTTTTTTGT TGAGAATTTT ATATATATTT TATTTTATTT AATATTTATA TGAGTGTTGT TTTAGGGTTT 160
FWA 92-13a.ab1   AAGTTTATAT ATTTTTTTGT TGAGAATTTT ATATATATTT TATTTTATTT AATATTTATA TGAGTGTTGT TTTAGGGTTT 160
FWA 92-14a.ab1   AAGTTTATAT ATTTTTTTGT TGAGAATTTT ATATATATTT TATTTTATTT AATATTTATA TGAGTGTTGT TTTAGGGTTT 160
FWA 92-15a.ab1   AAGTTTATAT ATTTTTTTGT TGAGAATTTT ATATATATTT TATTTTATTT AATATTTATA TGAGTGTTGT TTTAGGGTTT 160
FWA 92-16a.ab1   AAGTTTATAT ATTCTTTTGT TGAGAATTTT ATATATATTT TATTTTATTT AATATTTATA TGAGTGTTGT TTTAGGGTTT 160
FWA 92-17a.ab1   AAGTTTATAT ATTTTTTTGT TGAGAATTTT ATATATATTT TATTTTATTT AATATTTATA TGAGTGTTGT TTTAGGGTTT 160

                         180                  200                  220                  240
fwa repeats      TTGCTTTTCG CCATTGGTCC AAGTGCTATT TGGTTGTTTA AGGTTGCTTT TAGCACACAA CTTTAATATT ATTTTTATGT 239
FWA 92-1a.ab1    TTGTTTTTTG TTATTGGTTT AAGTGTTATT TGGTTGTTTA AGGTTGTTTT TAGTATATAA TTTTAATATT ATTTTTATGT 240
FWA 92-2a.ab1    TTGTTTTTTG TTATTGGTTT AAGTGTTATT TGGTTGTTTA AGGTTGTTTT TAGTATATAA TTTTAATATT ATTTTTATGT 240
FWA 92-3a.ab1    TTGTTTTTTG TTATTGGTTT AAGTGTTATT TGGTTGTTTA AGGTTGTTTT TAGTATATAA TTTTAATATT ATTTTTATGT 240
FWA 92-4a.ab1    TTGTTTTTTG TTATTGGTTT AAGTGTTATT TGGTTGTTTA AGGTTGTTTT TAGTATATAA TTTTAATATT ATTTTTATGT 240
FWA 92-5a.ab1    TTGTTTTTTG TTATTGGTTT AAGTGTTATT TGGTTGTTTA AGGTTGTTTT TAGTATATAA TTTTAATATT ATTTTTATGT 240
FWA 92-6a.ab1    TTGTTTTTTG TTATTGGTTT AAGTGTTATT TGGTTGTTTA AGGTTGTTTT TAGTATATAA TTTTAATATT ATTTTTATGT 240
FWA 92-7a.ab1    TTGTTTTTTG TTATTGGTTT AAGTGTTATT TGGTTGTTTA AGGTTGTTTT TAGTATATAA TTTTAATATT ATTTTTATGT 240
FWA 92-8a.ab1    TTGTTTTTTG TTACTGGTTT AAGTGTTATT TGGTTGTTTA AGGTTGTTTT TAGTATATAA TTTTAATATT ATTTTTATGT 240
FWA 92-10a.ab1   TTGTTTTTTG TTATTGGTTT AAGTGTTATT TGGTTGTTTA AGGTTGTTTT TAGTATATAA TTTTAATATT ATTTTTATGT 240
FWA 92-11a.ab1   TTGTTTTTTG TTATTGGTTT AAGTGTTATT TGGTTGTTTA AGGTTGTTTT TAGTATATAA TTTTAATATT ATTTTTATGT 240
FWA 92-12a.ab1   TTGTTTTTTG TTATTGGTTT AAGTGTTATT TGGTTGTTTA AGGTTGTTTT TAGTATATAA TTTTAATATT ATTTTTATGT 240
FWA 92-13a.ab1   TTGTTTTTTG TTATTGGTTT AAGTGTTATT TGGTTGTTTA AGGTTGTTTT TAGTATATAA TTTTAATATT ATTTTTATGT 240
FWA 92-14a.ab1   TTGTTTTTTG TTATTGGTTT AAGTGTTATT TGGTTGTTTA AGGTTGTTTT TAGTATATAA TTTTAATATT ATTTTTATGT 240
FWA 92-15a.ab1   TTGTTTTTTG TTATTGGTTT AAGTGTTATT TGGTTGTTTA AGGTTGTTTT TAGTATATAA TTTTAATATT ATTTTTATGT 240
FWA 92-16a.ab1   TTGTTTTTTG TTATTGGTTT AAGTGTTATT TGGTTGTTTA AGGTTGTTTT TAGTATATAA TTTTAATATT ATTTTTATGT 240
FWA 92-17a.ab1   TTGTTTTTTG TTATTGGTTT AAGTGTTATT TGGTTGTTTA AGGTTGTTTT TAGTATATAA TTTTAATATT ATTTTTATGT 240

                         260                  280                  300                  320
fwa repeats      TTTTTTTCTT ACGATTTATC GATTTGTGGG ATACTGACAA TCAGATTATT GTTGTTTTTT CCAGCCAAAT ATCAGA--TC 317
FWA 92-1a.ab1    TTTTTTTTTT ATGATTTATT GATTTGTGGG ATATTGATAA TTAGATTATT GTTGTTTTTT TTAGTTAAAT ATTAGAATTT 320
FWA 92-2a.ab1    TTTTTTTTTT ATGATTTATT GATTTGTGGG ATATTGATAA TTAGATTATT GTTGTTTTTT TTAGTTAAAT ATTAGAATTT 320
FWA 92-3a.ab1    TTTTTTTTTT ATGATTTATT GATTTGTGGG ATATTGATAA TTAGATTATT GTTGTTTTTT TTAGTTAAAT ATTAGAATTT 320
FWA 92-4a.ab1    TTTTTTTTTT ATGATTTATT GATTTGTGGG ATATTGATAA TTAGATTATT GTTGTTTTTT TTAGTTAAAT ATTAGAATTT 320
FWA 92-5a.ab1    TTTTTTTTTT ATGATTTATT GATTTGTGGG ATATTGATAA TTAGATTATT GTTGTTTTTT TTAGTTAAAT ATTAGAATTT 320
FWA 92-6a.ab1    TTTTTTTTTT ATGATTTATT GATTTGTGGG ATATTGATAA TTAGATTATT GTTGTTTTTT TTAGTTAAAT ATTAGAATTT 320
FWA 92-7a.ab1    TTTTTTTTTT ATGATTTATT GATTTGTGGG ATATTGATAA TTAGATTATT GTTGTTTTTT TTAGTTAAAT ATTAGAATTT 320
FWA 92-8a.ab1    TTTTTTTTT- ATGATTTATT GATTTGTGGG ACATTGATAA TTAGATTATT GTTGTTTTTT TTAGTTACAT ATTAGAATTT 319
FWA 92-10a.ab1   TTTTTTTTTT ATGATTTATT GATTTGTGGG ATATTGATAA TTAGATTATT GTTGTTTTTT TTAGTTAAAT ATTAGAATTT 320
FWA 92-11a.ab1   TTTTTTTTTT ATGATTTATT GATTTGTGGG ATATTGATAA TTAGATTATT GTTGTTTTTT TTAGTTAAAT ATTAGAATTT 320
FWA 92-12a.ab1   TTTTTTTTTT ATGATTTATT GATTTGTGGG ATATTGATAA TTAGATTATT GTTGTTTTTT TTAGTTAAAT ATTAGAATTT 320
FWA 92-13a.ab1   TTTTTTTTTT ATGATTTATT GATTTGTGGG ATATTGATAA TTAGATTATT GTTGTTTTTT TTAGTTAAAT ATTAGAATTT 320
FWA 92-14a.ab1   TTTTTTTTTT ATGATTTATT GATTTGTGGG ATATTGATAA TTAGATTATT GTTGTTTTTT TTAGTTAAAT ATTAGAATTT 320
FWA 92-15a.ab1   TTTTTTTTT- ATGATTTATT GATTTGTGGG ATATTGATAA TTAGATTATT GTTGTTTTTT TTAGTTAAAT ATTGGAATTT 319
FWA 92-16a.ab1   TTTTTTTTT- ATGATTTATT GATTTGTGGG ATATTGATAA TTAGATTATT GTTGTTTTTT TTAGTTAAAT ATTAGAATTT 319
FWA 92-17a.ab1   TTTTTTTTTT ATGATTTATT GATTTGTGGG ATATTGATAA TTAGATTATT GTTGTTTTTT TTAGTTAAAT ATTAGAATTT 320
```

```
                                    340                     360                     380                     400
fwa repeats      TTGCGCCGCT CTTTATCCCA TTCAACATTC ATACGAGCAC CGCTTTACGG TTTTTGCTTT TCGGCATTGG TCGAACTGCT 397
FWA 92-1a.ab1    TTGTGTTGTT TTTTATTTTA TTTAATATTT ATATGAGTAT TGTTTTATGG TTTTTGTTTT TTGGTATTGG TTGAATTGTT 400
FWA 92-2a.ab1    TTGTGTTGTT TTTTATTTTA TTTAATGTTT ATATGAGTAT TGTTTTATGG TTTTTGTTTT TTGGTATTGG TTGAATTGTT 400
FWA 92-3a.ab1    TTGTGTTGTT TTTTATTTTA TTTAATATTT ATATGAGTAT TGTTTTATGG TTTTTGTTTT TTGGTATTGG TTGAATTGTT 400
FWA 92-4a.ab1    TTGTGTTGTT TTTTATTTTA TTTAATATTT ATATGAGTAT TGTTTTATGG TTTTTGTTTT TTGGTATTGG TTGAATTGTT 400
FWA 92-5a.ab1    TTGTGTTGTT TTTTATTTTA TTTAATATTT ATATGAGTAT TGTTTTATGG TTTTTGTTTT TTGGTATTGG TTGAATTGTT 400
FWA 92-6a.ab1    TTGTGTTGTT TTTTATTTTA TTTAATATTT ATATGAGTAT TGTTTTATGG TTTTTGTTTT TTGGTATTGG TTGAATTGTT 400
FWA 92-7a.ab1    TTGTGTTGTT TTTTATTTTA TTTAATATTT ATATGAGTAT TGTTTTATGG TTTTTGTTTT TTGGTATTGG TTGAATTGTT 400
FWA 92-8a.ab1    TTGTGTTGTT TTTTATTTTA TTTAATATTT ATATGAGTAT TGTTTTATGG TTTTTGTTTT TTGGTATTGG TTGAATTGTT 399
FWA 92-10a.ab1   TTGTGTTGTT TTTTATTATA TTTAATATTT ATATGAGTAT TGTTTTATGG TTTTTGTTTT TTGGTATTGG TTGAATTGTT 400
FWA 92-11a.ab1   TTGTGTTGTT TTTTATTTTA TTTAATATTT ATATGAGTAT TGTTTTATGG TTTTTGTTTT TTGGTATTGG TTGAATTGTT 400
FWA 92-12a.ab1   TTGTGTTGTT TTTTATTTTA TTTAATATTT ATATGAGTAT TGTTTTATGG TTTTTGTTTT TTGGTATTGG TTGAATTGTT 400
FWA 92-13a.ab1   TTGTGTTGTT TTTTATTTTA TTTAATATTT ATATGAGTAT TGTTTTATGG TTTTTGTTTT TTGGTATTGG TTGAATTGTT 400
FWA 92-14a.ab1   TTGTGTTGTT TTTTATTTTA TTTAATATTT ATATGAGTAT TGTTTTATGG TTTTTGTTTT TTGGTATTGG TTGAATTGTT 400
FWA 92-15a.ab1   TTGTGTTGTT TTTCATTTTA TTTAATATTT ATATGAGTAT TGTTTTATGG TTTTTGTTTT TTGGTATTGG TTGAATTGTT 399
FWA 92-16a.ab1   TTGTGTTGTT TTTTATTTTA TTTAATATTT ATATGAGTAT TGTTTTATGG TTTTTGTTTT TTGGTATTGG TTGAATTGTT 399
FWA 92-17a.ab1   TTGTGTTGTT TTTTATTTTA TTTAATATTT ATATGAGTAT TGTTTTATGG CTTTTGTTTT TTGGTATTGG TTGAATTGTT 400

                                    420                     440                     460                     480
fwa repeats      ATTTGGTTGT TTAAGGTTGC TTTTAGCACA CAACTTTAAT ATTATTTTTA TGTTTTCTTC TTACGATTTA TCGATTTGTA 477
FWA 92-1a.ab1    ATTTGGTTGT TTAAGGTTGT TTTTAGTATA TAATTTTAAT ATTATTTTTA TGTTTTTTTT TTATGATTTA TTGATTTGTA 480
FWA 92-2a.ab1    ATTTGGTTGT TTAAGGTTGT TTTTAGTATA TAATTTTAAT ATTATTTTTA TGTTTTTTTT TTATGATTTA TTGATTTGTA 480
FWA 92-3a.ab1    ATTTGGTTGT TTAAGGTTGT TTTTAGTATA TAATTTTAAT ATTATTTTTA TGTTTTTTTT TTATGATTTA TTGATTTGTA 480
FWA 92-4a.ab1    ATTTGGTTGT TTAAGGTTGT TTTTAGTATA TAATTTTAAT ATTATTTTTA TGTTTTTTTT TTATGATTTA TTGATTTGTA 480
FWA 92-5a.ab1    ATTTGGTTGT TTAAGGTTGT TTTTAGTATA TAATTTTAAT ATTATTTTTA TGTTTTTTTT TTATGATTTA TTGATTTGTA 480
FWA 92-6a.ab1    ATTTGGTTGT TTAAGGTAGT TTTTAGTATA TAATTTTAAT ATTATTTTTA TGTTTTTTTT TTATGATTTA TTGATTTGTA 480
FWA 92-7a.ab1    ATTTGGTTGT TTAAGGTTGT TTTTAGTATA TAATTTTAAT ATTATTTTTA TGTTTTTTTT TTATGATTTA TTGATTTGTA 480
FWA 92-8a.ab1    ATTTGGTTGT TTAAGGTTGT TTTTAGTATA TAATTTTAAT ATTATTTTTA TGTTTTTTTT TTATGATTTA TTGATTTGTA 479
FWA 92-10a.ab1   ATTTGGTTGT TTAAGGTTGT TTTTAGTATA TAATTTTAAT ATTATTTTTA TGTTTTTTTT TTATGATTTA TTGATTTGTA 480
FWA 92-11a.ab1   ATTTGGTTGT TTAAGGTTGT TTTTAGTATA TAATTTTAAT ATTATTTTTA TGTTTTTTTT TTATGATTTA TTGATTTGTA 480
FWA 92-12a.ab1   ATTTGGTTGT TTAAGGTTGT TTTTAGTATA TAATTTTAAT ATTATTTTTA TGTTTTTTTT TTATGATTTA TTGATTTGTA 480
FWA 92-13a.ab1   ATTTGGTTGT TTAAGGTTGT TTTTAGTATA TAATTTTAAT ATTATTTTTA TGTTTTTTTT TTATGATTTA TTGATTTGTA 480
FWA 92-14a.ab1   ATTTGGTTGT TTAAGGTTGT TTTTAGTATA TAATTTTAAT ATTATTTTTA TGTTTTTTTT TTATGATTTA TTGATTTGTA 480
FWA 92-15a.ab1   ATTTGGTTGT TTAAGGTTGT TTTTAGTATG TAATTTTAAT ATTATTTTTA TGTTTTTTTT TTATGATTTA TTGATTTGTA 479
FWA 92-16a.ab1   ATTTGGTTGT TTAAGGTTGT TTTTAGTATA TAATTTTAAT ATTATTTTTA TGTTTTTTTT TTATGATTTA TTGATTTGTA 479
FWA 92-17a.ab1   ATTTGGTTGT TTAAGGTTGT TTTTAGTATA TAATTTTAAT ATTATTTTTA TGTTTTTTTT TTATGATTTG TTGATTTGTA 480

                                    500                     520                     540                     560
fwa repeats      GGATACTGAC AATCAGATTT TTGTTGTTTT TTTCAGCCAA AAATCAGATT TTTTTTACAC TTTGTTTAGA GAATGATTTT 557
FWA 92-1a.ab1    GGATATTGAT AATTAGATTT TTGTTGTTTT TTTTAGTTAA AAATTAGATT TTTTT-ATAT TTTGTTTAGA GAATGATTTT 559
FWA 92-2a.ab1    GGATATTGAT AATTAGATTT TTGTTGTTTT TTTTAGTTAA AAATTAGATT TTTTT-ATAT TTTGTTTAGA GAATGATTTT 559
FWA 92-3a.ab1    GGATATTGAT AATTAGATTT TTGTTGTTTT TTTTAGTTGA AAATTAGATT TTTTT-ATAT TTTGTTTAGA GAATGATTTT 559
FWA 92-4a.ab1    GGATATTGAT AATTAGATTT TTGTTGTTTT TTTTAGTTAA AAATTAGATT TTTTT-ATAT TTTGTTTAGA GAATGATTTT 559
FWA 92-5a.ab1    GGATATTGAT AATTAGATTT TTGTTGTTTT TTTTAGTTAA AAATTAGATT TTTTT-ATAT TTTGTTTAGA GAATGATTTT 559
FWA 92-6a.ab1    GGATATTGAT AATTAGATTT TTGTTGTTTT TTTTAGTTAA AAATTAGATT TTTTT-ATAT TTTGTTTAGA GAATGATTTT 559
FWA 92-7a.ab1    GGATATTGAT AATTAGATTT TTGTTGTTTT TTTTAGTTAA AA-TTAGATT TTTTT-ATAT TTTGTTTAGA GAATGATTTT 558
FWA 92-8a.ab1    GGATATTGAT AATTAGATTT TTGTTGTTTT TTTTAGTTAA AAATTAGATT TTTTT-ATAT TTTGTTTAGA GAATGATTTT 558
FWA 92-10a.ab1   GGATATTGAT AATTAGATTT TTGTTGTTTT TTTTAGTTAA AAATTAGATT TTTTT-ATAT TTTGTTTAGA GAATGATTTT 559
FWA 92-11a.ab1   GGATATTGAT AATTAGATTT TCGTTGTTTT TTTTAGTTAA AAATTAGATT TTTTT-ATAT TTTGTTTAGA GAATGATTTT 559
FWA 92-12a.ab1   GGATATTGAT AATTAGATTT TTGTTGTTTT TTTTAGTTAA AAATTAGATT TTTTT-ATAT TTTGTTTAGA GAATGATTTT 559
FWA 92-13a.ab1   GGATATTGAT AATTAGATTT TTGTTGTTTT TTTTAGTTAA AAATTAGATT TTTTT-ATAT TTTGTTTAGA GAATGATTTT 559
FWA 92-14a.ab1   GGGTATTGAT AATTAGATTT TTGTTGTTTT TTTTAGTTAA AAATTAGATT TTTTT-ATAT TTTGTTTAGA GAATGATTTT 559
FWA 92-15a.ab1   GGATATTGAT AATTAGATTT TTGTTGTTTT TTTTAGTTAA AAATTAGATT TTTTT-ATAT TTTGTTTAGA GAATGATTTT 558
FWA 92-16a.ab1   GGATATTGAT AATTAGATTT TTGTTGTTTT TTTTAGTTAA AAATTAGATT TTTTT-ATAT TTTGTTTAGA GAATGATTTT 558
FWA 92-17a.ab1   GGATATTGAT AATTAGATTT TTGTTGTTTT TTTTAGTTAA AAATTAGATT TTTTT-ATAT TTTGTTTAGA GAATGATTTT 559

fwa repeats      GGTTCC--- 563
FWA 92-1a.ab1    GGTTAAGGG 568
FWA 92-2a.ab1    GGTTAAGGG 568
FWA 92-3a.ab1    GGTTAAGGG 568
FWA 92-4a.ab1    GGTTAAGGG 568
FWA 92-5a.ab1    GGTTAAGGG 568
FWA 92-6a.ab1    GGTTAAGGG 568
FWA 92-7a.ab1    GGTTAAGGG 567
FWA 92-8a.ab1    GGTTAAGGG 567
FWA 92-10a.ab1   GGTTAAGGG 568
FWA 92-11a.ab1   GGTTAAGGG 568
FWA 92-12a.ab1   GGTTAAGGG 568
FWA 92-13a.ab1   GGTTAAGGG 568
FWA 92-14a.ab1   GGTTAAGGG 568
FWA 92-15a.ab1   GGTTAAGGG 567
FWA 92-16a.ab1   GGTTAAGGG 567
FWA 92-17a.ab1   GGTTAAGGG 568
```

```
                                       20                                      40                                      60                                      80
SDC repeats.AB1  GCTGACGTGT ATTGTGAAAT CTTATCTTTA TAACTAACGT GTACAATGAA ATCTTATCTT TATAACTGAC GTGTACTGTG  80
SDC 9-1.AB1      GCTGACGTGT ATTGTGAAAT TTTATTTTTA TAATTAACGT GTATAATGAA ATTTTATTTT TATAACTGAC GTGTACTGTG  80
SDC 9-2.AB1      GCTGACGTGT ATTGTGAAAT TTTATTTTTA TAATTAACGT GTATAATGAA ATTTTATTTT TATAACTGAC GTGTACTGTG  80
SDC 9-3.AB1      GCTGACGTGT ATTGTGAAAT TTTATTTTTA TAATTAACGT GTATAATGAA ATTTTATTTT TATAACTGAC GTGTACTGTG  80
SDC 9-4.AB1      GCTGACGTGT ATTGTGAAAT TTTATTTTTA TAACTAACGT GTACAATGAA ATTTTATTTT TATAACTGAC GTGTACTGTG  80
SDC 9-5.AB1      GCTGACGTGT ATTGTGAAAT TTTATTTTTA TAATTAACGT GTATAATGAA ATTTTATTTT TATAACTGAC GTGTACTGTG  80
SDC 9-6.AB1      GCTGACGTGT ATTGTGAAAT TTTATTTTTA TAATTAACGT GTATAATGAA ATTTTATTTT TATAATTGAC GTGTACTGTG  80
SDC 9-7.AB1      GCTGACGTGT ATTGTGAAAT TTTATTTTTA TAACTAACGT GTACAATGAA ATTTTATTTT TATAACTGAC GTGTACTGTG  80
SDC 9-8.AB1      GCTGACGTGT ATTGTGAAAT TTTATTTTTA TAATTAACGT GTATAATGAA ATTTTATTTT TATAACTGAC GTGTACTGTG  80
SDC 9-9.AB1      GCTGACGTGT ATTGTGAAAT TTTATTTTTA TAACTAACGT GTACAATGAA ATTTTATTTT TATAACTGAC GTGTACTGTG  80
SDC 9-10.AB1     GCTGACGTGT ATTGTGTAAT TTTATTTTTA TAATTAACGT GTATAATGAA ATTTTATTTT TATAACTGAC GTGTACTGTG  80
SDC 9-11.AB1     GCTGACGTGT ATTGTGAAAT TTTATTTTTA TAATTAACGT GTATAATGAA ATTTTATTTT TATAATTGAT GTGTATTGTG  80
SDC 9-12.AB1     GTTGACGTGT ATTGTGAAAT TTTATTTTTA TAATTAACGT GTATAATGAA ATTTTATTTT TATAACTGAT GTGTATTGTG  80
SDC 9-13.AB1     GTTGACGTGT ATCGTGAAAT TTTATTTTTA TAATTAACGT GTATAATGAA ATTTTATTTT TATAACTGAT GTGTATTGTG  80

                                      100                                     120                                     140                                     160
SDC repeats.AB1  AAATCTTATC TTTATAACTG ATGTGTACTG TGAAATCTTA TCTTTATAAC TGACGTGTAC TGTGAAATTT TATCTAAATA  160
SDC 9-1.AB1      AAATTTTATT TTTATAACTG ATGTGTACTG TGAAATTTTA TTTTTATAAT TGACGTGTAC TGTGAAATTT TATTTAAATA  160
SDC 9-2.AB1      AAATTTTATT TTTATAACTG ATGTGTACTG TGAAATTTTA TTTTTATAAT TGACGTGTAC TGTGAAATTT TATTTAAATA  160
SDC 9-3.AB1      AAATTTTATT TTTATAACTG ATGTGTACTG TGAAATTTTA TTTTTATAAT TGACGTGTAC TGTGAAATTT TATTTAAATA  160
SDC 9-4.AB1      AAATTTTATT TTTATAACTG GTGTGTACTG TGAAATTTTA TTTTTATAAC AGACGTGTAC TGTGAAATTT TATTTAAATA  160
SDC 9-5.AB1      AAATTTTATT TTTATAACTG ATGTGTACTG TGAAATTTTA TTTTTATAAT TGACGTGTAC TGTGAAATTT TATTTAAATA  160
SDC 9-6.AB1      AAATTTTATT TTTATAATTG ATGTGTACTG TGAAATTTTA TTTTTATAAT TGACGTGTAC TGTGAAATTT TATTTAAATA  160
SDC 9-7.AB1      AAATTTTATT TTTATAACTG GTGTGTACTG TGAAATTTTA TTTTTATAAC AGACGTGTAC TGTGAAATTT TATTTAAATA  160
SDC 9-8.AB1      AAATTTTATT TTTATAACTG ATGTGTACTG TGAAATTTTA TTTTTATAAT TGACGTGTAC TGTGAAATTT TATTTAAATA  160
SDC 9-9.AB1      AAATTTTATT TTTATAACTG GTGTGTACTG TGAAATTTTA TTTTTATAAC AGACGTGTAC TGTGAAATTT TATTTAAATA  160
SDC 9-10.AB1     AAATTTTATT TCTATAACTG ATGTGTACTG TGAAATTTTA TTTTTATAAT TGACGTGTAC TGTGAAATTT TATTTAAATA  160
SDC 9-11.AB1     AAATTTTATT TTTATAATTG ATGTGTATTG TGAAATTTTA TTTTTATAAT TGATGTGTAC TGTGAAATTT TATTTAAATA  160
SDC 9-12.AB1     AAATTTTATT TTTATAATTG ATGTGTATTG TGAAATTTTA TTTTTATAAT TGACGTGTAT TGTGAAATTT TATTTAAATA  160
SDC 9-13.AB1     AAATTTTATT TTTATAATTG ATGTGTATTG TGAAATTATA TTTTTATAAT TGACGTGTAT TGTGAAATTT TATTTAAATA  160

                                      180                                     200                                     220
SDC repeats.AB1  ACTGACGTGT GCAGCGAAAT CTCATCTTTA TATCAACGTG TACTGTAAAA TCTTATCTTC ATAA  224
SDC 9-1.AB1      ACTGACGTGT ACAGCGAAAT TTTATTTTTA TATTAACGTG TACTGTAAAA TTTTATTTTT ATAA  224
SDC 9-2.AB1      ACTGACGTGT ACGGCGAAAT TTTATTTTTA TATTAACGTG TACTGTAAAA TTTTATTTTT ATAA  224
SDC 9-3.AB1      ACTGACGTGT ACAGCGAAAT TTTATTTTTA TATTAACGTG TACTGTAAAA TTTTATTTTT ATAA  224
SDC 9-4.AB1      ACTGACGTGT ACAGCGAAAT TTTATTTTTA TATTAACGTG TACTGTAAAA TTTTATTTTT ATAA  224
SDC 9-5.AB1      ACTGACGTGT ACAGCGAAAT TTTATTTTTA TATTAACGTG TACTGTAAAA TTTTATTTTT ATAA  224
SDC 9-6.AB1      ACTGACGTGT ACAGCGAAAT TTTATTTTTA TATTAACGTG TACTGTAAAA TTTTATTTTT ATAA  224
SDC 9-7.AB1      ACTGACGTGT ACAGCGAAAT TTTATTTTTA TATTAACGTG TACTGTAAAA TTTTATTTTT ATAA  224
SDC 9-8.AB1      ACTGACGTGT ACAGCGAAAT TTTATTTTTA TATTAACGTG TACTGTAAAA TTTTATTTTT ATAA  224
SDC 9-9.AB1      ACTGACGTGT ACAGCGAAAT TTTATTTTTA TATTAACGTG TACTGTAAAA TTTTATTTTT ATAA  224
SDC 9-10.AB1     ACTGACGTGT ATAGCGAAAT TTTATTTTTA TATTAACGTG TATTGTAAAA TTTTATTTTT ATAA  224
SDC 9-11.AB1     ACTGATGTGT ACGGCTAAAT TTTATTTTTA TATTAACGTG TACTGTAAAA TTTTATTTTT ATAA  224
SDC 9-12.AB1     ATTGACGTGT ACAGCGAAAT TTTATTTTTA TATTAACGTG TATTGTAAAA TTTTATTTTT ATAA  224
SDC 9-13.AB1     ACTGATGTGT ACAGCGAAAT TTTATTTTTA TATTAACGTG TATTGTAAAA TTTTATTTTT ATAA  224
```

```
                                      20                                      40                                      60                                      80
SDC repeats   TTATGAAGAT AAGATTTTAC AGTACACGTT GATATAAAGA TGAGATTTCG CTGTACACGT CAGTTATTTA GATAAAATTT 80
SDC 2-1.ab1   TTATGAAGAT AAGATTTTAC AGTATACGTT GATATAAAGA TGAGATTTCG CTGTATACGT TAGTTATTTA GATAAA-TTT 79
SDC 2-2.ab1   TTATGAAGAT AAGATTTTAC AGTATATGTT GATATAAAGA TGAGATTTCG CTGTATGCGT CAGTTATTTG GATAAAATTT 80
SDC 2-3.ab1   TTATGAAGAT AAGATTTTAT AGTATACGTT GATATAAAGA TGAGATTTCG TTGTATACGT TAGTTATTTA GATAAAATTT 80
SDC 2-4.ab1   TTATGAAGAT AAGATTTTAT AGTATACGTT GATATAAAGA TGAGATTTCG TTGTATATGT TAGTTATTTA GATAAAATTT 80
SDC 2-5.ab1   TTATGAAGAT AAGATTTTAC AGTATATGTT GATATAAAGA TGAGATTTTG TTGTATATGT TAGTTATTTA GATAAAATTT 80
SDC 2-6.ab1   TTATGAAGAT AAGATTTTAT AGTATACGTT GATATAAAGA TGAGATTTCG CTGTATATGT TAGTTATTTA GATAAAATTT 80
SDC 2-7.ab1   TTATGAAGAT AAGATTTTAT AGTATACGTT GATATAAAGA TGAGATTTCG CTGTATACGT CAGTTATTTA GATAAAATTT 80
SDC 2-8.ab1   TTATGAAGAT AAGATTTTAT AGTATACGTT GATATAAAGA TGAGATTTCG CTGTATACGT TAGTTATTTA GATAAAATTT 80
SDC 2-9.ab1   TTATGAAGAT AAGATTTTAT AGTATACGTT GATATAAAGA TGAGATTTCG CTGTATACGT TAGTTATTTA GATAAAATTT 80
SDC 2-10.ab1  TTATGAAGAT AAGATTTTAT AGTATACGTT GATATAAAGA TGAGATTTCG TTGTATATGT TAGTTATTTA GATAAAATTT 80
SDC 2-11.ab1  TTATGAAGAT AAGATTTTAT AGTATACGTT GATATAAAGA TGAGATTTCG CTGTATACGT CAGTTATTTA GATAAAATTT 80
SDC 2-12.ab1  TTATGAAGAT AAGATTTTGT AGTATACGTT GATATAAAGA TGAGATTTCG TTGTATATGT TAGTTATTTA GATAAAATTT 80
SDC 2-13.ab1  TTATGAAGAT AAGATTTTAT AGTATACGTT GATATAAAGA TGAGATTTCG TTGTATACGT CAGTCATTTA GATAAAATTT 80
SDC 2-14.ab1  TTATGAAGGT AAGATTTTAT AGTATATGTT GATATAAGGA TGAGATTTTG TTGTATACGT TAGTTATTTA GATAAAATTT 80
SDC 2-15.ab1  TTATGAAGAT AAGATTTTAT AGTATATGTT GATATAAAGA TGAGATTTTG TTGTATATGT TAGTTATTTA GATAAAATTT 80
SDC 2-16.ab1  TTATGAAGAT AAGATTTTAT GGTATACGTT GATATAAAGA TGAGATTTCG TTGTATACGT TAGTTATTTA GATAAAATTT 80
SDC 2-17.ab1  TTATGAAGAT AAGATTTTAC AGTATACGTT GATATAAAGA TGAGATTTTG CTGTATACGT CAGTTATTTA GATAAAATTT 80
SDC 2-18.ab1  TTATGAAGAT AAGATTTTAT AGTATACGTT GATATAAAGA TGAGATTTCG TTGTATACGT CAGTTATTTA GATAAAATTT 80

                                     100                                     120                                     140                                     160
SDC repeats   CACAGTACAC GTCAGTTATA AAGATAAGAT TTCACAGTAC ACATCAGTTA TAAAGATAAG ATTTCACAGT ACACGTCAGT 160
SDC 2-1.ab1   TACAGTATAC GTCAGTTATA AAGATAAGAT TTTACAGTAT ATATCAGTTA TAAAGATAAG ATTTTATAGT GTACGTTAGT 159
SDC 2-2.ab1   TACAGTATAC GTTAGTTATA AAGATAAGAT TTTACAGTAT ATATCAGTTA TAAAGATAGG ATTTTACAGT ATACGTCAGT 160
SDC 2-3.ab1   TACAGTATAC GTCAGTTATA AAGATAAGAT TTTATAGTAT ATATCAGTTA TAAAGATAAG ATTTTACAGT ATACGTCAGT 160
SDC 2-4.ab1   TACAGTATAC GTCAGTTATA AAGATAAGAT TTTATAGTAT ATATTAGTTA TAAAGATAAG ATTTTATAGT ATACGTTAGT 160
SDC 2-5.ab1   TACAGTATAC GTCAGTTATA AAGATAAGAT TTTACAGTAT ATATCAGTTA TAAAGATAAG ATTTCACAGT ACATGTCAGT 160
SDC 2-6.ab1   TACAGTATAC GTCAGTTATA AAGATAAGAT TTTACAGTAT ATATTAGTTA TAAAGATAAG ATTTTATAGT ATACGTTAGT 160
SDC 2-7.ab1   TACAGTATAT GTCAGTTATA AAGATAAGAT TTTACAGTAT ATATCAGTTA TAAAGATAAG ATTTTACAGT ATACGTTAGT 160
SDC 2-8.ab1   TACAGTATAC GTTAGTTATA AAGATAAGAT TTTACAGTAT ATATTAGTTA TAAGGATAAG ATTTTACAGT ATACGTTAGT 160
SDC 2-9.ab1   TACAGTATAC GTTAGTTATA AAGATAAGAT TTTACAGTAT ATATTAGTTA TAAGGATAAG ATTTTACAGT ATACGTTAGT 160
SDC 2-10.ab1  TACAGTATAC GTCAGTTATA AAGATAAGAT TTTACAGTAT GTATCAGTTG TAAAGATAAG ATTTTGTAGT ATACGTCAGT 160
SDC 2-11.ab1  TACAGTATAT GTCAGTTATA AAGATAAGAT TTTACAGTAT ATATCAGTTA TAAAGATAAG ATTTTACAGT ATACGTCAGT 160
SDC 2-12.ab1  TATAGTATAT GTTAGTTATA AAGATAAGAT TTTACAGTAT ATATCAGTTA TAAAGATAAG ATTTTACAGT ATACGTCAGT 160
SDC 2-13.ab1  TATAGTATAC GTTAGTTATA AAGATAAGAT TTTATAGTAT GTATTAGTTA TAAAGATAAG ATTTTATAGT ACACGTTAGT 160
SDC 2-14.ab1  TATAGTATAC GTCGGTTATA AAGATAAGAT TTTACAGTAC ATATCAGTTA TAAAGATAAG ATTTTACAGT ATACGTCAGT 160
SDC 2-15.ab1  TATAGTATAT GTTAGTTATA AAGATAAGAT TTTATAGTAT ATATTAGTTA TAAAGATAAG ATTTTATAGT ATATGTTAGT 160
SDC 2-16.ab1  TATAGTATAC GTCAGTTATA AAGATAAGAT TTTACAGTAT ATATTAGTTA TAAAGATAAG ATTTTATAGT ATACGTCAGT 160
SDC 2-17.ab1  TATAGTATAC GTTAGTTATA AAGATAAGAT TTTATAGTAT ATATCAGTTA TAAAGATAAG ATTTTATAGT GTACGTCAGT 160
SDC 2-18.ab1  TATAGTATAC GTTAGTTATA AAGATAAGAT TTTATAGTAT GTATTAGTTA TAAAGATAAG ATTTTATAGT ACACGTTAGT 160

                                     180                                     200                                     220
SDC repeats   TATAAAGATA AGATTTCATT GTACACGTTA GTTATAAAGA TAAGATTTCA CAATACACGT CAGC 224
SDC 2-1.ab1   TATAAAGATA AGATTTTATT GTATACGTTA GTTATAAAGA TAAGATTTTA TAATATGCGT CAGT 223
SDC 2-2.ab1   TATAAAGATA AGATTTTATT GTATACGTTA GTTATAAAGA TAAGATTTTA TAATATACGT CAGT 224
SDC 2-3.ab1   TATAAAGATA AGATTTTATT GTATACGTTA GTTATAAAGA TAAGATTTTA TAATATATGT CAGT 224
SDC 2-4.ab1   TATAAAGATA AGATTTTATT GTATACGTTA GTTATAAAGA TAAGATTTTA TAATATACGT TAGT 224
SDC 2-5.ab1   TATAAAGATA AGATTTTATT GTATATGTTA GTTATAAAGA TAGGATTTCA CAATACATGT TAGT 224
SDC 2-6.ab1   TATAAAGATA AGATTTTATT GTACATGTTA GTTATAAAGA TAAGATTTCA TAATATACGT CAGT 224
SDC 2-7.ab1   TATAAAGATA AGATTTTATT GTATACGTTA GTTATAAAGA TAAGATTTTA TAATATACGT CAGT 224
SDC 2-8.ab1   TATAAAGATA AGACTTTATT GTATATGTTA GTTATAAAGA TAAGATTTTA TAATATGTGT CAGT 224
SDC 2-9.ab1   TATAAAGATA AGATTTTATT GTATATGTTA GCTATAAAGA TAAGATTTTA TAATATGTGT CAGT 224
SDC 2-10.ab1  TATAAAGATA AGATTTTATT GTATACGTTA GTTATAAAGA TGAGATTTTA TAATATACGT TAGT 224
SDC 2-11.ab1  TATAAAGATA AGATTTTATT GTATACGTTA GTTATAAAGA TAAGATTTTA TAATATACGT CAGT 224
SDC 2-12.ab1  TATAAAGATA AGATTTTATT GTATACGTTA GTTATAAAGA TAAGATTTTA TAATATATGT TAGT 224
SDC 2-13.ab1  TATAAAGATA GGATTTCATT GTACACGTTA GTTATAAAGA TAAGATTTTA TAATATACGT TAGT 224
SDC 2-14.ab1  TATAAAGATA AGATTTTATT GTATACGTTA GTTATAAAGA TAAGATTTTA CGGTACACGT CAGT 224
SDC 2-15.ab1  TATAAGGATA AGATTTTATT GTATATGTTA GTTATAAAGA TAAGATTTTA TAATATATGT TAGT 224
SDC 2-16.ab1  TATAAAGATA AGATTTTATT GTATACGTTA GTTATAAAGA TAAGATTTTA TAATATACTT TAGT 224
SDC 2-17.ab1  TATAAAGATA AGATTTTATT GTATACGTTA GTTATAAAGA TAAGATTTTA TAATATACGT CAGT 224
SDC 2-18.ab1  TATAAAGATA GGATTTCATT GTACACGTTA GTTATAAAGA TAAGATTTTA TAATATACGT TAGT 224
```

```
                                       20                    40                    60                    80
SDC repeats     TTATGAAGAT AAGATTTTAC AGTACACGTT GATATAAAGA TGAGATTTCG CTGTACACGT CAGTTATTTA GATAAAATTT  80
SDC 92-1.ab1    TTATGAAGAT AAGATTTTAT AGTATACGTT GATATAAAGA TGAGATTTCG TTGTATACGT TAGTTATTTA GGTAAAATTT  80
SDC 92-2.ab1    TTATGAAGAT AAGATTTTAC AGTATACGTT GATATAAAGA TGAGATTTCG TTGTATACGT CAGTTATTTA GATAAGATTT  80
SDC 92-5.ab1    TTATGAAGAT AAGATTTTAC AGTATACGTT GATGTAAAGA TGAGATTTCG TTGTATACGT TAGTTATTTA GATAAAATTT  80
SDC 92-6.ab1    TTATGAAGAT AAGATTTTAC AGTATACGTT GATATAAAGA TGAGATTTCG TTGTATACGT CAGTTATTTA GATAAAATTT  80
SDC 92-7.ab1    TTATGAAGAT AAGATTTTAT AGTATACGTT GATATAAAGA TGAGATTTTG TTGTATATGT TAGTTATTTA GATAAAATTT  80
SDC 92-8.ab1    TTATGAAGAT AAGATTTTAC AGTATACGTT GATATAAAGA TGAGATTTCG TTGTATACGT CAGTTATTTA GATAAAATTT  80
SDC 92-9.ab1    TTATGAAGAT AAGATTTTAC AGTATACGTT GATATAAAGA TGAGATTTCG CTGTATACGT CATTTATTTA GATAAAATTT  80
SDC 92-10.ab1   TTATGAAGAT AAGATTTTAT AGTATATGTT GATATAAAGA TGAGATTTTG CTGTATACGT CAGTTATTTA GATAAAATTT  80
SDC 92-11.ab1   TTATGAAGAT AAGATTTTAT AGTATACGTT GACATAAAGA TGAGATTTCG TTGTATACGT CAGTTATTTA GATAAAATTT  80
SDC 92-12.ab1   TTATGAAGAT AAGATTTTAT AGTATACGTT GATATAAAGA TGAGATTTTG TTGTATATGT TAGTTATTTA GATAAAATTT  80
SDC 92-13.ab1   TGATGAAAAG AAAATTTTAC AGTACACGTT GATATAAAGA TGAGATTTCG TTGTATACGT TAGTTATTTA GAAAAAATTT  80
SDC 92-14.ab1   TTATGAAGAT AAGATTTTAC AGTATACGTT GATATAAAGA TGAGATTTCG CTGTATACGT CAGTTATTTA GATAAAATTT  80
SDC 92-15.ab1   TTATGAAGAT AAGATTTTAT AGTATACGTT GATATAAAGA TGAGATTTCG TTGTATACGT TAGTTATTTA GGTAAAATTT  80
SDC 92-16.ab1   TTATGAAGAT AAGATTTTAC AGTATACGTT GATATAAAGA TGAGATTTCG CTGTATACGT CAGTTATTTA GATAAAATTT  80
SDC 92-17.ab1   TTATGAAGAT AAGATTTTAT AGTATATGTT GATATAAAGA TGAGATTTCG CTGTATACGT TAGTTATTTA GATAAAATTT  80
SDC 92-18.ab1   TTATGAAGAT AAGATTTTAC AGTATACGTT GATATAAAGA TGAGATTTCG TTGTATACGT CAGTTATTTA GATAAAATTT  80
SDC 92-19.ab1   TTATGAAGAT AAGATTTTAT AGTATACGTT GATATAAAGA TGAGATTTCG CTGTATACGT TAGTTGTTTA GATAAAGTTT  80
SDC 92-20.ab1   TTATGAAGAT AAGATTTTAC AGTATACGTT GATGTAAAGA TGAGATTTCG TTGTATACGT TAGTTATTTA GATAAAATTT  80
SDC 92-21.ab1   TTATGAAGAT AAGATTTTAC AGTATACGTT GATGTAAAGA TGAGATTTCG CTGTATATGT TAGTTATTTA GATAAAATTT  80

                                      100                   120                   140                   160
SDC repeats     CACAGTACAC GTCAGTTATA AAGATAAGAT TTCACAGTAC ACATCAGTTA TAAAGATAAG ATTTCACAGT ACACGTCAGT  160
SDC 92-1.ab1    TATAGTATAC GTCAGTTATA AAGATAAGAT TTTACAGTAT ATATTAGTTA TAAAGATAAG ATTTTATAGT ATACGTCAGT  160
SDC 92-2.ab1    TATAGTATAT GTTAGTTATA AAGATAAGAT TTTATAGTAT ATATCAGTTA TAAAGATAAG ATTTTACAGT ATACGTCAGT  160
SDC 92-5.ab1    TATAGTATAC GTCAGTTATA AAGATAAGAT TTTACAGTGT ATATTAGTTA TGAAGATAAG ATTTTACAGT ATACGTTAGT  160
SDC 92-6.ab1    TACAGTATAC GTCAGTTATA AAGATAAGAT TTTACAGTAT ATATTAGTTA TAAAGATAAG ATTTTACAGT ATACGTTAGT  160
SDC 92-7.ab1    TATAGTATAT GTTAGTTATA AAGATAAGAT TTTATAGTAT ATATTAGTTA TAAAGATAAG ATTTTATAGT ATATGTTAGT  160
SDC 92-8.ab1    TATAGTATAT GTTAGTTATA AAGATAAGAT TTTATAGTAT ATATTAGTTA TAAAGATAAG ATTTTATAGT ATATGTTAGT  160
SDC 92-9.ab1    TATAGTATAC GTCAGTTATA AAGATAAGAT TTTATAGTAT ATATCAGTTA TAAAGATAAG ATTTTACAGT ATACGTCAGT  160
SDC 92-10.ab1   TACAGTATAC GTCAGTTATA AAGATAAGAT TTTACAGTAT ATATTAGTTA TAAAGATAAG ATTTTATAGT ATATGTTAGT  160
SDC 92-11.ab1   TATAGTATAC GTCAGTTATA AAGATAAGAT TTTACGGTAT ATATTAGTTA TAAAGATAAG ATTTTACAGT ATACGTCAGT  160
SDC 92-12.ab1   TATAGTATAT GTTAGTTATA AAGATAAGAT TTTATAGTAT ATATTAGTTA TAAAGATAAG ATTTTATAGT ATATGTTAGT  160
SDC 92-13.ab1   TATAGTATAC GTCAGTTATA AAGATAAGAT TTTACAGTGT ATATTAGTTA TGAAGATAAG ATTTTACAGT ATACGTTAGT  160
SDC 92-14.ab1   TACAGTATAC GTCAGTTATA AAGATAAGAT TTTACAGTAT ATATCAGTTA TAAAGATAAG ATTTTACAGT ATACGTCAGT  160
SDC 92-15.ab1   TATAGTATAC GTCAGTTATA AAGATAAGAT TTTACAGTAT ATATTAGTTA TAAAGATAAG ATTTTATAGT ATACGCCAGT  160
SDC 92-16.ab1   TACAGTATAC GTCAGTTATA AAGATAAGAT TTTACAGTAT ATATCAGTTA TAAAGATAAG ATTTTACAGT ATACGTCAGT  160
SDC 92-17.ab1   TACAGTATAC GTCAGTTATA AAGATAAGAT CTTACAGTAT ATATCAGTTA TAAAGATAAG ATTTTATAGT ATACGTTAGT  160
SDC 92-18.ab1   TACAGTATAC GTCAGTTATA AAGATAAGAT TTTACAGTAT ATATTAGTTA TAAAGATAAG ATTTTATAGT ATACGTCAGT  160
SDC 92-19.ab1   TATAGTATAC GTTAGTTATA AAGATAAGAT TTTATGGTAT ATATTAGTTA TAAAGATAAG ATTTTACAGT ATATGTTAGT  160
SDC 92-20.ab1   TATAGTATAC GTCAGTTATA AAGATAAGAT TTTACAGTGT ATATTAGTTA TGAAGATAAG ATTTTACAGT ATACGTTAGT  160
SDC 92-21.ab1   TATAGTATAC GTCAGTTATA AAGATAAGAT TTTATAGTAT ATATTAGTTA TAAAGATAAG ATTTTACAGT ATACGTTAGT  160

                                      180                   200                   220
SDC repeats     TATAAAGATA AGATTTCATT GTACACGTTA GTTATAAAGA TAAGATTTCA CAATACACGT CAGC  224
SDC 92-1.ab1    TATAAAGATA AGATTTTATT GTATACGTTA GTTATAAAGA TAAGATTTTG TAATATACGT TAGT  224
SDC 92-2.ab1    TATAAAGATA AGATTTTATT GTATACGTTA GTTGTAAAGA TAAGATTTTA TAATATACGT TAGT  224
SDC 92-5.ab1    TATAAAGATA AGATTTTATT GTATACGTTA GTTATAAAGA TAAGATTTTA TAATATACGT TAGT  224
SDC 92-6.ab1    TATAAAGATA AGATTTTATT GTATACGTTA GTTATAAAGA TAAGATTTTA TAATATACGT CAGT  224
SDC 92-7.ab1    TATAAAGATA AGATTTTATT GTATATGTTA GTTATAAAGA TAAGATTTTA TAATATATGT TAGC  224
SDC 92-8.ab1    TATAAAGATA AGATTTTATT GTATACGTTA GTTATAAAGA TAAGATTTTA TAATATACGT CAGT  224
SDC 92-9.ab1    TATAAAGATA AGATTTTATT GTATACGTTA GTTATAAAGA TAAGATTTTA TAATATACGT TAGT  224
SDC 92-10.ab1   TATAAAGATA AGATTTTATT GTATATGTTA GTTATAAGGA TAAGATTTTA TAATATATTT CAGT  224
SDC 92-11.ab1   TATAAAGATA AGATTTTATT GTATACGTTA GTTATAAAGA TAAGATTTTA TAATATATGT TAGT  224
SDC 92-12.ab1   TATAAAGATA AGATTTTATT GTATATGTTA GTTATAAAGA TAAGATTTTA TAATATATGT TAGT  224
SDC 92-13.ab1   TATAAAGATA AGATTTTATT GTATACGTTA GTTATAAAGA TAAGATTTTA TAATATACGT TAGT  224
SDC 92-14.ab1   TATAAAGATA AGATTTTATT GTATACGTTA GTTATAAAGA TAAGATTTTA TAATATACGT TAGT  224
SDC 92-15.ab1   TATAAAGATA AGATTTTATT GTATACGTTA GTTATAAAGA TAAGATTTTG TAATATACGT TAGT  224
SDC 92-16.ab1   TATAAAGATA AGATTTTATT GTATACGTTA GTTATAAAGA TAAGATTTTA TAATATACGT TAGT  224
SDC 92-17.ab1   TATAAAGATA AGATTTTATT GTATATGTTA GTTATAAAGA TAAGATTTTA TAATATACGT TAGT  224
SDC 92-18.ab1   TATAAAGATA AGATTTTATT GTATACGTTA GTTATAAAGA TAAGATTTTG TAATATACGT TAGT  224
SDC 92-19.ab1   TATAAAGATA AGATTTTATT GTATACGTTA GTTATAAAGA TAAGATTTTA TAATATACGT CAGT  224
SDC 92-20.ab1   TATAAAGATA AGATTTTATT GTATACGTTA GTTATAAAGA TAAGATTTTA TAATATACGT TAGT  224
SDC 92-21.ab1   TATAAAGATA AGATTTTATT GTATATGTTA GTTATAAAGA TAAGATTTTA TAATATACGT CAGT  224
```

```
SDC repeats     TTATGAAGAT AAGATTTTAC AGTACACGTT GATATAAAGA TGAGATTTCG CTGTACACGT CAGTTATTTA GATAAAATTT 80
SDC 492-1.ab1   TTATGAAGAT AAGATTTTAT AGTATACGTT GACATAAAGA TGAGATTTCG TTGTATACGT TAGTTATTTA GATAAAATTT 80
SDC 492-2.ab1   TTATGAAGAT AAGATTTTAT AGTATATGTT GATATAAAGA TGAGATTTTG TTGTATATGT TAGTTATTTA GATAAAATTT 80
SDC 492-3.ab1   TTATGAAGAT AAGATTTTAT AGTATATGTT GATATAAAGA TGAGATTTTG TTGTATATGT TAGTTATTTA GATAAAATTT 80
SDC 492-4.ab1   TTATGAAGAT AAGATTTTAT AGTATACGTT GATATAAAGA TGAGATTTCG TTGTATACGT TAGTTATTTA GATAAAATTT 80
SDC 492-5.ab1   TTATGAAGAT AAGATTTTAT AGTATACGTT GATATAAAGA TGAGATTTCG TTGTATACGT CAGTTATTTA GATAAAATTT 80
SDC 492-6.ab1   TTATGAAGAT AAGATTTTAT AGTATACGTT GATATAAAGA TGAGATTTCG TTGTATACGT TAGTTATTTA GATAAAATTT 80
SDC 492-7.ab1   TTATGAAGAT AAGATTTTAT AGTATACGTT GATATAAAGA TGAGATTTCG TTGTATACGT TAGTTATTTA GATAAAATTT 80
SDC 492-8.ab1   TTATGAAGAT AAGATTTTAT AGTATATGTT GATATAAAGA TGAGATTTTG TTGTATATGT TAGTTATTTA GATAAAATTT 80
SDC 492-9.ab1   TTATGAAGAT AAGATTTTAT AGTATATGTT GATATAAAGA TGAGATTTTG TTGTATATGT TAGTTATTTA GATAAAATTT 80
SDC 492-10.ab1  TTATGAAGAT AAGATTTTAT AGTATACGTT GATATAAAGA TGAGATTTCG TTGTATACGT CAGTTATTTA GATAAAATTT 80
SDC 492-11.ab1  TTATGAAGAT AAGATTTTAT AGTATACGTT GATATAAAGA TGAGATTTCG TTGTATACGT TAGTTATTTA GATAAAATTT 80
SDC 492-12.ab1  TTATGAAGAT AAGATTTTAT AGTATACGTT GATATAAAGA TGAGATTTCG TTGTATACGT TAGTTATTTA GATAAAATTT 80
SDC 492-13.ab1  TTATGAAGAT AAGATTTTAT AGTATATGTT GATATAAAGA TGAGATTTTG TTGTATATGT TAGTTATTTA GATAAAATTT 80
SDC 492-14.ab1  TTATGAAGAT AAGATTTTAT AGTATACGTT GATATAAAGA TGAGATTTCG TTGTATACGT TAGTTATTTA GATAAAATTT 80
SDC 492-15.ab1  TTATGAAGAT AAGATTTTAT AGTATATGTT GATATAAAGA TGAGGTTTTG TTGTATATGT TAGTTATTTA GATAAAATTT 80
SDC 492-16.ab1  TTATGAAGAT AAGATTTTAT AGTATATGTT GATATAAAGA TGAGATTTTG TTGTATATGT TAGTTATTTA GATAAAATTT 80

SDC repeats     CACAGTACAC GTCAGTTATA AAGATAAGAT TTCACAGTAC ACATCAGTTA TAAAGATAAG ATTTCACAGT ACACGTCAGT 160
SDC 492-1.ab1   TATAGTATAC GTTAGTTATA AAGATAAGAT TTTATAGTAT ATATTAGTTA TAAAGATAAG ATTTTATAGT ATACGTTAGT 160
SDC 492-2.ab1   TATAGTATAT GTTAGTTATA AAGATAAGAT TTTATAGTAT ATATTAGTTA TAAAGATAAG ATTTTATAGT ATATGTTAGT 160
SDC 492-3.ab1   TATAGTATAT GTTAGTTATA AAGATAAGAT TTTATAGTAT ATATTAGTTA TAGAGATAAG ATTTTATAGT ATATGTTAGT 160
SDC 492-4.ab1   TATAGTATAC GTTAGTTATA AAGATAAGAT TTTATAGTAT ATATTAGTTA TAAAGATAAG ATTTTATAGT ATACGTTAGT 160
SDC 492-5.ab1   TATAGTATAC GTCAGTTATA AAGATAAGAT TTTATAGTAT ATATTAGTTA TAAAGATAAG ATTTTATAGT ATACGTTAGT 160
SDC 492-6.ab1   TATAGTATAC GTTAGTTATA AAGATAAGAT TTTATAGTAT ATATTAGTTA TAAAGATAAG ATTTTATAGT ATACGTTAGT 160
SDC 492-7.ab1   TATAGTATAC GTTAGTTATA AAGATAAGAT TTTATAGTAT ATATTAGTTA TAAAGATAAG ATTTTATAGT ATACGTTAGT 160
SDC 492-8.ab1   TATAGTATAT GTTAGTTATA AAGATAAGAT TTTATAGTAT ATATTAGTTA TAAAGATAAG ATTTTATAGT ATATGTTAGT 160
SDC 492-9.ab1   TATAGTATAT GTTAGTTATA AAGATAAGAT TTTATAGTAT ATATTAGTTA TAAAGATAAG ATTTTATAGT ATATGTTAGT 160
SDC 492-10.ab1  TATAGTATAC GTCAGTTATA AAGATAAGAT TTTATAGTAT ATATTAGTTA TAAAGATAAG ATTTTATAGT ATACGTTAGT 160
SDC 492-11.ab1  TATAGTATAC GTTAGTTATA AAGATAAGAT TTTATAGTAT ATATTAGTTA TAAAGATAAG ATTTTATAGT ATACGTTAGT 160
SDC 492-12.ab1  TATAGTATAC GTTAGTTATA AAGATAAGAT TTTATAGTAT ATATTAGTTA TAAAGATAAG ATTTTATAGT ATACGTTAGT 160
SDC 492-13.ab1  TATAGTATAT GTTAGTTATA AAGATAAGAT TTTATAGTAT ATATTAGTTA TAAAGATAAG ATTTTATAGT ATATGTTAGT 160
SDC 492-14.ab1  TATAGTATAC GTTAGTTATA AAGATAAGAT TTTATAGTAT ATATTAGTTA TAAAGATAAG ATTTTATAGT ATACGTTAGT 160
SDC 492-15.ab1  TATAGTATAT GTTAGTTATA AGGATAAGAT TTTATAGTAT ATATTAGTTG TAAAGATAAG ATTTTATAGT ATATGTTGGT 160
SDC 492-16.ab1  TATAGTATAT GTTAGTTATA AAGATAAGAT TTTATAGTAT ATATTAGTTA TAAAGATAAG ATTTTATAAT ATATGTTAGT 160

SDC repeats     TATAAAGATA AGATTTCATT GTACACGTTA GTTATAAAGA TAAGATTTCA CAATACACGT CAGC 224
SDC 492-1.ab1   TATAAAGATA AGATTTTATT GTATACGTTA GTTATAAGGA TAAGATTTTA TAATATACGT TAGT 224
SDC 492-2.ab1   TATAAAGATA AGATTTTATT GTATATGTTA GTTATAAAGA TAAGATTTTA TAATATATGT TAGT 224
SDC 492-3.ab1   TATAAAGATA GGATTTTATT GTGTATGTTA GTTATAAAGA TAAGATTTTA TAATATATGT TAGT 224
SDC 492-4.ab1   TATAAAGATA AGATTTTATT GTATACGTTA GTTATAAGGA TAAGATTTTA TAATATACGT TAGT 224
SDC 492-5.ab1   TATAAAGATA AGATTTTATT GTATACGTTA GTTATAAAGA TAAGATTTTA TAATATACGT TAGT 224
SDC 492-6.ab1   TATAAAGATA AGATTTTATT GTATACGTTA GTTATAAGGA TAAGATTTTA TAATATACGT TAGT 224
SDC 492-7.ab1   TATAAAGATA AGATTTTATT GTATACGTTA GTTATAAGGA TAAGATTTTA TAATATACGT TAGT 224
SDC 492-8.ab1   TATAAAGATA AGATTTTATT GTATATGTTA GTTATAAAGA TAAGATTTTA TAATATATGT TAGT 224
SDC 492-9.ab1   TATAAAGATA AGATTTTATT GTATATGTTA GTTATAAAGA TAAGATTTTA TAATATATGT TAGT 224
SDC 492-10.ab1  TATAAAGATA AGATTTTATT GTATACGTTA GTTATAAAGA TAAGATTTTA TAATATACGT TAGT 224
SDC 492-11.ab1  TATAAAGATA AGATTTTATT GTATACGTTA GTTATAAGGA TAAGATTTTA TAATATACGT TAGT 224
SDC 492-12.ab1  TATAAAGATA AGATTTTATT GTATACGTTA GTTATAAGGA TAAGATTTTA TAATATACGT TAGT 224
SDC 492-13.ab1  TATAAAGATA AGATTTTATT GTATATGTTA GTTATAAAGA TAAGATTTCA CAATACATGT TAGT 224
SDC 492-14.ab1  TATAAAGATA AGATTTTATT GTATACGTTA GTTATAAGGA TAAGATTTTA TAATATACGT TAGT 224
SDC 492-15.ab1  TATAAAGATA AGATTTTATT GTATATGTTA GTTATAAAGA TAAGATTTTA TAATACATGT TAGT 224
SDC 492-16.ab1  TTTA-ATATA AAATATATTA GGGTTTAT-- GTTAATAAGT ATAGAGTTTA AAGTAGAA-T GAG- 219
```
