# Supplementary material for: SRA-Domain Proteins Required for DRM2-Mediated De Novo DNA Methylation
Source: PLoS Genet. 2008 Nov 28;4(11):e1000280. doi: 10.1371/journal.pgen.1000280 (PMC2582956; doi:10.1371/journal.pgen.1000280)
Supplement: Table S1 — Primer sequences. (0.03 MB DOC) [file pgen.1000280.s006.doc]

**Table S1. Primer Sequences**

| Primer Number | Sequence |
| --- | --- |
| JP2452 | TCGTGGTGGTGAGTTTGTTAC |
| JP2453 | CAGCATCATCACAAGCATCC |
| JP3395 | AATGTAAGTTGTAAACCATTTGAACGTGACC |
| JP3396 | CAGGCATCCGTAGAACTCATGAGC |
| JP5393 | ATTTTCTTAAAAAATTTTCAACTCATTTTTTTTAAAAAA |
| JP5392 | GAGTAATATATGGAAGAATTATTAATAAAGTGGYTGTAGT |
| JP1821 | CAATATACRATCCAAAAAACARTTATTAAAATAATATCTTAA |
| JP1822 | GTTGTATAAGTTTAGTTTTAATTTTAYGGATYAGTATTAATTT |
| JP4039 | TYATYGTAYAAAAYATTYAAGATATATGATGAATTATTTGATTATTATT |
| JP4045 | CCAAATCTTARARATCTCTTRAAATRTTTRRAATTCTTCCAARCRRCRTAAA |
| JP2004 | GGTTTTATATTAATATTAAAGAGTTATGGG TYGAAGTTT |
| JP4423 | AACCAAAATCATTCTCTAAACAAAATATAA AAAAATC |
| M17 | TTTTCCCTAGTTGAGATGGGAATT |
| JP4225 | CAAAGACGATGACGACAAATGACGTGAGTCTAGAGAGTTAATTAAG |
| JP4226 | CTTAATTAACTCTCTAGACTCACGTCATTTGTCGTCATCGTCTTTG |
